# How do income changes impact on mental health and wellbeing for working-age adults? A systematic review and meta-analysis

**DOI:** 10.1016/S2468-2667(22)00058-5

**Published:** 2022-06-01

**Authors:** Rachel M Thomson, Erik Igelström, Amrit Kaur Purba, Michal Shimonovich, Hilary Thomson, Gerry McCartney, Aaron Reeves, Alastair Leyland, Anna Pearce, S Vittal Katikireddi

**Affiliations:** MRC/CSO Social and Public Health Sciences Unit, University of Glasgow, Glasgow, UK; School of Social and Political Sciences, University of Glasgow, Glasgow, UK; Public Health Scotland, Edinburgh, UK; Department of Social Policy and Intervention, University of Oxford, Oxford, UK; MRC/CSO Social and Public Health Sciences Unit, University of Glasgow, Glasgow, UK; MRC/CSO Social and Public Health Sciences Unit, University of Glasgow, Glasgow, UK; Public Health Scotland, Edinburgh, UK

## Abstract

**Background:**

Lower incomes are associated with poorer mental health and wellbeing, but the extent to which income has a causal effect is debated. We aimed to synthesise evidence from studies measuring the impact of changes in individual and household income on mental health and wellbeing outcomes in working-age adults (aged 16–64 years).

**Methods:**

For this systematic review and meta-analysis, we searched MEDLINE, Embase, Web of Science, PsycINFO, ASSIA, EconLit, and RePEc on Feb 5, 2020, for randomised controlled trials (RCTs) and quantitative non-randomised studies. We had no date limits for our search. We included English-language studies measuring effects of individual or household income change on any mental health or wellbeing outcome. We used Cochrane risk of bias (RoB) tools. We conducted three-level random-effects meta-analyses, and explored heterogeneity using meta-regression and stratified analyses. Synthesis without meta-analysis was based on effect direction. Critical RoB studies were excluded from primary analyses. Certainty of evidence was assessed using Grading of Recommendations Assessment, Development and Evaluation (GRADE). This study is registered with PROSPERO, CRD42020168379.

**Findings:**

Of 16 521 citations screened, 136 were narratively synthesised (12·5% RCTs) and 86 meta-analysed. RoB was high: 30·1% were rated critical and 47·1% serious or high. A binary income increase lifting individuals out of poverty was associated with 0·13 SD improvement in mental health measures (95% CI 0·07 to 0·20; n=42 128; 18 studies), considerably larger than other income increases (0·01 SD improvement, 0·002 to 0·019; n=216 509, 14 studies). For wellbeing, increases out of poverty were associated with 0·38 SD improvement (0·09 to 0·66; n=101 350, 8 studies) versus 0·16 for other income increases (0·07 to 0·25; n=62 619, 11 studies). Income decreases from any source were associated with 0·21 SD worsening of mental health measures (–0·30 to –0·13; n=227 804, 11 studies). Effect sizes were larger in low-income and middle-income settings and in higher RoB studies. Heterogeneity was high (*I*^2^=79-87%). GRADE certainty was low or very low.

**Interpretation:**

Income changes probably impact mental health, particularly where they move individuals out of poverty, although effect sizes are modest and certainty low. Effects are larger for wellbeing outcomes, and potentially for income losses. To best support population mental health, welfare policies need to reach the most socioeconomically disadvantaged.

**Funding:**

Wellcome Trust, Medical Research Council, Chief Scientist Office, and European Research Council.

## Introduction

Poor mental health is a leading global cause of disability linked with worsened social and physical health outcomes.^1–4^ Social conditions are thought to be important in driving mental health burden and increasing health inequalities.^5^ Income could be particularly important, with those on lower incomes less able to access health-promoting goods and services and maintain a feeling of control or security over their lives.^6–8^ Crucially, income is also amenable to policy intervention.^9,10^

Low income is demonstrably correlated with poor mental health, but it is less clear whether changing someone’s income will improve their mental health.^11,12^ Those with poor mental health are more likely to experience subsequent income losses, indicating potential for reverse causation or health selection.^13^ Additionally, poor mental health and income changes have common causes (eg, job loss) that introduce potential for confounding.^14^ There is evidence that income source and setting might influence any potential causal relationship, with more convincing evidence of effects on mental health for those on low incomes, in low-income countries, or where money is from a policy intervention rather than one-off events like lottery wins.^15–19^

Governments have the ability to redistribute incomes through taxation or welfare policies, potentially influencing mental health and health inequalities, especially in the working-age population which is particularly influenced by such policy levers (eg, via income taxation or unemployment benefits).^20–22^ There have been narrative reviews considering the impact of income changes on adult mental health,^23^ but previous quantitative evidence syntheses have focused on specific exposures or are restricted to low-income or middle-income settings.^24,25^ Using tools designed to aid in the interpretation of potential threats to causal inference, we aimed to quantify the impact of any changes in individual or household incomes on mental health outcomes for working-age adults, considering whether the source or size of income change was important, and whether any beneficial or detrimental impacts varied by socioeconomic background or setting.

## Methods

### Overview

**Figure 1:**
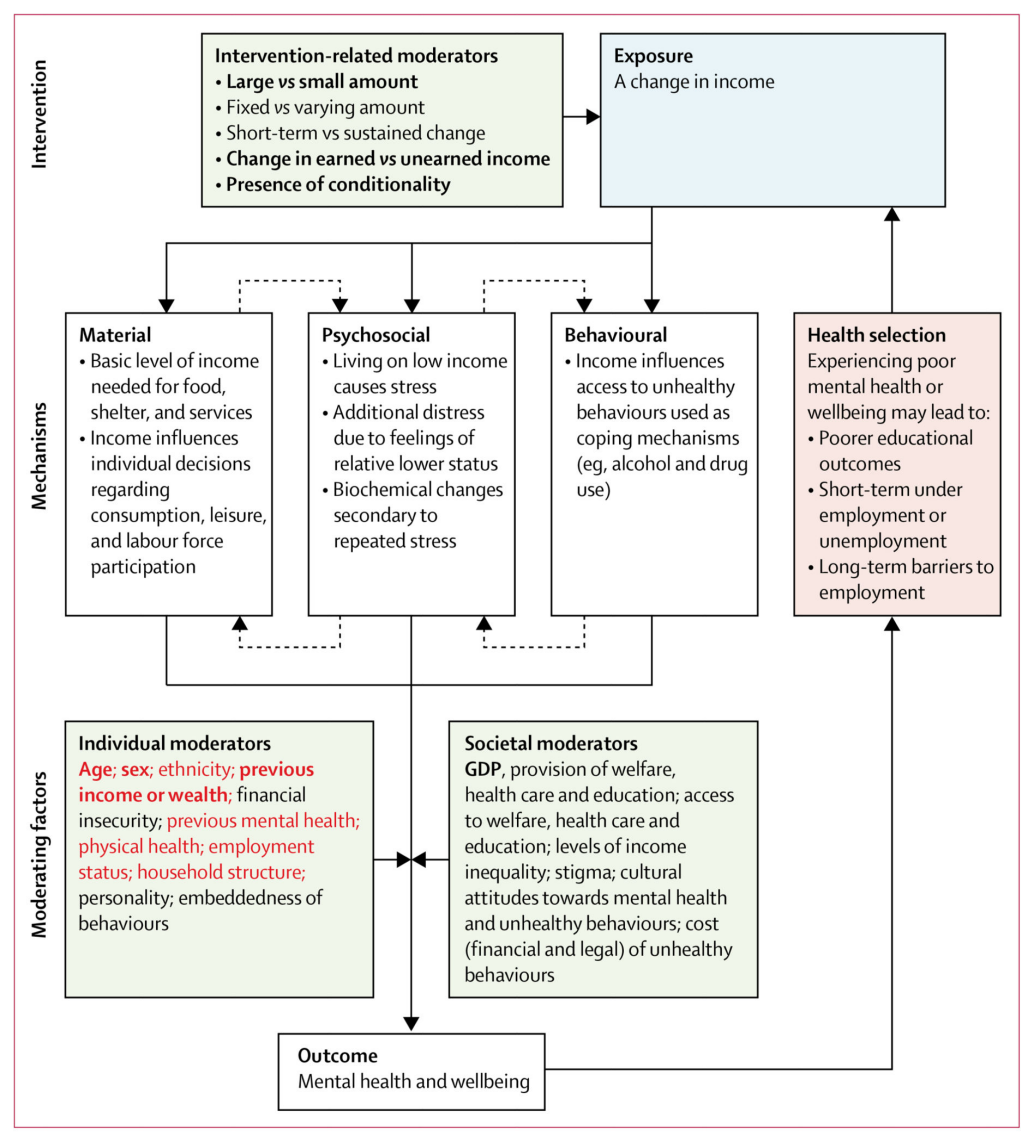
Logic model indicating theory of change for income and mental health Variables considered as important potential confounders in this study are indicated in red and were selected a priori by the researchers on the basis of variables that were viewed as key confounders in the literature. Variables considered important potential effect modifiers for exploration in this study are indicated in bold; these were selected based on assumptions regarding their likely importance and anticipated data availability. GDP=gross domestic product.

We did a systematic review and meta-analysis as per Preferred Reporting Items for Systematic review and Meta-Analysis (PRISMA) and Synthesis Without Meta-analysis (SWiM) in systematic reviews: reporting guidance.^26,27^ This study is registered with PROSPERO, CRD42020168379.^28^ A logic model was developed drawing on a systematic review of theory by Benzeval and colleagues^13^ and advice from subject experts (figure 1; further background in protocol^28^), which was used to identify important confounders and effect modifiers to explore in subgroup analysis. Protocol deviations are reported in the appendix (p 2).

### Literature search

Searches of peer-reviewed and relevant grey literature (eg, economics working papers and academic theses) were done using MEDLINE, Embase, Web of Science, PsycINFO, ASSIA, EconLIT, and RePEc on Feb 5, 2020, using a strategy developed with an information specialist (appendix p 14). No date limits were applied to the search. Search terms included “mental health”, depression, anxiety, “anxiety disorder”, wellbeing, “quality of life”, “life satisfaction”, “psychological distress” AND income [or synonym] NEAR change [or synonym]. See the appendix for full details on the search terms (appendix p 14). Our population of interest was working-age adults (aged 16–64 years). Exposure or intervention of interest was change in household or individual income from any source, compared to no equivalent income change. All mental health (eg, depression or anxiety) or wellbeing (eg, life satisfaction or happiness) outcomes were eligible for inclusion (details on classification are in the appendix p 2). We included randomised and non-randomised quantitative studies, with the latter category including any non-randomised study design (eg, case-control, cohort, or natural experiment studies) provided comparison was made between an exposed and unexposed group. References were de-duplicated in Endnote (version X9) and imported to Covidence for screening.

### Study selection

All titles or abstracts and full-text papers were independently screened by RMT and a second reviewer (EI, AKP, MS, AL, AP, or SVK), with conflicts resolved by consensus or discussion with a third reviewer (SVK or HT). Non-English-language studies were excluded at the full-text stage. Reference lists of relevant systematic reviews and included studies were screened for additional studies. Where eligible studies contained overlapping or duplicate data, a set of decision rules considered alignment with our PECO (ie, population, exposure, comparator, and outcome) and risk of bias (RoB; appendix p 2).

### Data extraction and RoB assessment

Data were extracted in Excel (version 2202) by RMT and checked by a second reviewer (EI, AKP, MS, AL, AP, or SVK). RoB assessment was done independently at the datapoint or outcome level by RMT and the same second reviewer using the Cochrane Collaboration RoB-2 tool for randomised studies^29^ and Risk of Bias in Non-randomised Studies of Interventions (ROBINS-I) for non-randomised studies.^30^ A low ROBINS-I rating is considered comparable to a well-conducted randomised controlled trial (RCT) in assessing intervention effect; omitting any key confounder automatically merits at least a serious rating. Conflicts were resolved by consensus. Non-randomised studies rated critical were excluded from primary synthesis as per ROBINS-I guidance but explored in a sensitivity analysis. Further detail on extracted items, decision rules, and RoB assessment, including selection of prespecified key confounders, is available in the appendix (pp 3, 6).

### Data synthesis

#### Synthesis without meta-analysis (SWiM)

We synthesised findings from all studies within the SWiM. Direction of effect was coded as beneficial or harmful for both outcome domains separately at study level, with findings categorised as inconsistent if less than 70% of extracted datapoints reported one effect direction.^31^ As per Cochrane guidance, statistical significance was not taken into account during this classification.^32^ Modified effect direction plots displayed findings stratified by RoB and are presented in the appendix (p 96). Sign tests assessed evidence of effect (excluding studies at critical RoB or with inconsistent findings) and χ^2^ tests assessed differences in effect by RoB, study design (RCT *vs* non-randomised studies), or income change source.

#### Primary meta-analyses

We did random-effects meta-analyses, with mental health and wellbeing measures analysed separately. Heterogeneity was explored using the *I*^2^ statistic. Where two or more datapoints from one study were included in the same analysis (eg, sex-stratified results), three-level meta-analyses accounted for dependence of effect sizes.^33^ We used the restricted maximum likelihood estimator to estimate between-study variance.^34^ In our primary meta-analyses, we combined estimates from RCTs and non-randomised studies, as they provide contrasting strengths and weaknesses for our research question; however, we also did a subgroup analysis analysing them separately.

Continuous exposures of log (income) were analysed separately from binary exposures of income increases or decreases because the income–health relationship is thought to be curvilinear, with decreasing marginal returns at higher levels.^35^ Binary exposure datapoints were transformed to standardised mean difference (SMD) for synthesis, combining continuous and dichotomous outcomes as per Cochrane guidelines.^36^ Continuous exposure datapoints were transformed to standardised beta coefficients or odds ratios (ORs) for continuous and binary outcomes, respectively (appendix p 4).

Where more than ten studies were included in the meta-analysis, meta-regression explored heterogeneity by: characteristics of income change (increases *vs* decreases, small *vs* large changes, or movement across a poverty threshold), type of income change (source of transfer, earned *vs* unearned, if accompanied by conditionality), type of outcome measure (self-report *vs* validated tools or administrative data), and individual or setting (socioeconomic position [SEP], high-income country [HIC] *vs* low-income and middle-income country [LMIC]). Statistical analysis was done using the meta package (version 4·19-0) in RStudio.^37^

#### Subgroup analyses

Additionally, we stratified meta-analyses by sex, study design (RCT *vs* non-randomised studies), RoB (low or moderate *vs* serious *vs* critical), movement across a poverty threshold, participant SEP (as defined within the study), HIC versus LMIC, and whether non-working-age participants were included. Analyses stratified by poverty movements and SEP generated similar results due to data overlap; only the former are discussed in the Results section (all plots are available in the appendix p 97). Publication bias or small study effect was assessed using funnel plots and Egger’s test.

#### Certainty assessment

Certainty of evidence was assessed using the Grading of Recommendations Assessment, Development and Evaluation (GRADE) system.^38^ This combines information on five domains: risk of bias, imprecision, inconsistency (including statistical heterogeneity), indirectness (assessing how closely available data reflect the research question), and publication bias. Key outcomes reported were: the impact of income changes on grouped mental health outcomes and the impact of income changes on grouped wellbeing outcomes. A planned key outcome on the impact of changes in unearned income was not reported (appendix p 2). A condensed GRADE summary of findings (table) is presented, with additional details provided in the appendix (p 93).

No ethics approval was requested, as the research solely extracted non-disclosive data from previously published studies in which informed consent was obtained by the primary investigators.^28^

### Role of the funding source

The funders of the study had no role in study design, data collection, data analysis, data interpretation, or writing of the report.

## Results

Of 16 521 articles screened, 136 studies were eligible for inclusion (PRISMA flowchart, appendix p 18).^18,19,39–172^ 90 studies reported mental health outcomes (230 datapoints) and 65 reported wellbeing outcomes (146 datapoints). Studies excluded at the full-text stage are detailed in the appendix (p 69). Only 17 included studies were RCTs (12·5%; appendix p 28), all of which examined the effect of a binary income increase. Most included studies were from HICs (72·1%), with 26·5% from the USA.

Most studies (61·0%) reported on income changes without specifying their size, but 30·1% specifically reported effects of large income changes (>20% change, or described as large by study authors), and 8·8% small income changes. A sizeable minority (37·5%) focused on income changes in low SEP populations, with 20·6% measuring the effect of moves across a meaningful poverty or subsistence threshold (either explicitly reported as such or representing a large change in an exclusively low SEP population; appendix p 6). Income change source was not reported in 46·3% of studies, typically where income fluctuations were measured over time in panel data. Known sources were: welfare policies influencing income alone such as cash transfers (16·9%); disasters (7·4%); taxation or wage policies (6·6%); welfare policies with additional components beyond income (5·9%); lottery wins (5·1%); and income changes due to illness or caring responsibilities, salary changes, benefit advice services, or cash transfers from other sources (all <5%). Time horizons ranged from 0 to 288 months, with 12 months the most common interval between intervention and outcome (mean 34·3; median 14·5 [IQR 12–36]).

Risk of bias of the 136 included studies was high: 4·8% were rated low, 18·4% moderate or some concerns, 47·1% serious or high, and 30·1% critical (appendix p 63). 68 non-critical studies were included in primary meta-analyses, with an additional 18 critical RoB studies explored in sensitivity meta-analyses.

The effect direction in all 90 studies considering mental health outcomes is shown in an effect direction plot in the appendix (p 96). After excluding studies with inconsistent findings and at critical RoB, 88·9% reported a beneficial effect of income, where either an income increase was associated with improvement in mental health or an income decrease was associated with worsening of mental health (95% CI 77·4–95·8, n=54, p<0·0001). There was no evidence of difference in this percentage by RoB (p=0·55), study design (p=0·77), or income source (p=0·84).

**Figure 2:**
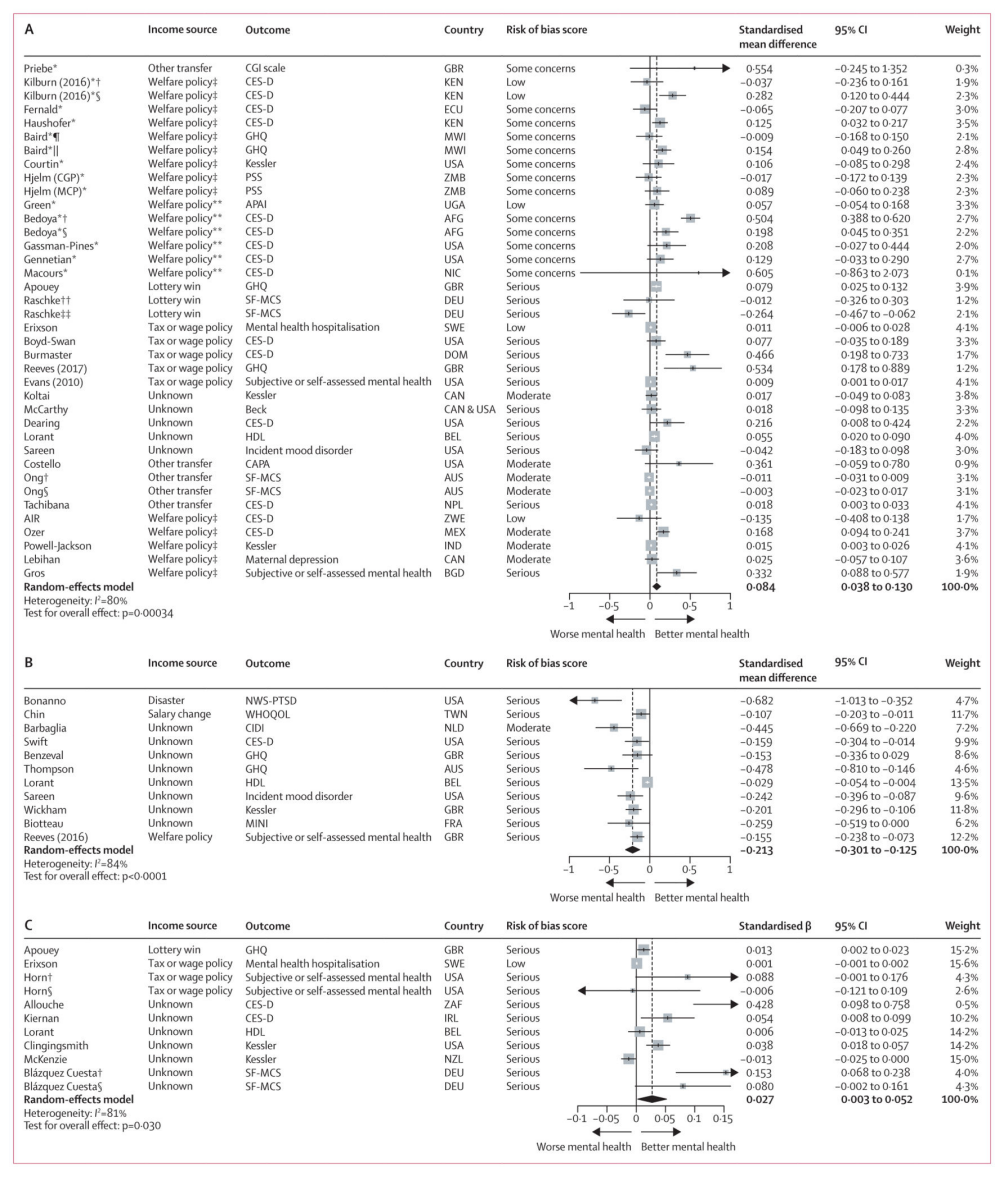
Forest plots for meta-analyses of studies reporting the effect of a binary income increase (A; n=32) and decrease (B; n=11) on a mental health outcome and of a continuous log (income) change on a mental health outcome (C; n=9) In A, number of people=258 637 and number of observations=1 756 078. In B, number of people=227 804 and number of observations=281 728. In C, number of people=1 510 221 and number of observations=3 036 715. In all panels, studies are sorted in order of trial status (randomised controlled trial or non-randomised study), income source, outcome, and risk of bias score. AFG=Afghanistan. APAI=Acholi Psychosocial Assessment Instrument. AUS=Australia. Beck=Beck depression inventory. BEL=Belgium. BGD=Bangladesh. CAN=Canada. CAPA=Child and Adolescent Psychiatric Assessment. CES-D=Center for Epidemiological Studies-Depression scale. CGI=Clinical Global Impression. CGP=Zambia child grant programme. CIDI=Composite International Diagnostic Interview. DEU=Germany. DOM=Dominican Republic. ECU=Ecuador. FRA=France. GBR=Great Britain. GHQ=General Health Questionnaire. HDL=Health and Daily Living Form. IND=India. IRL=Ireland. KEN=Kenya. Kessler=Kessler Psychological Distress scales. MCP=Zambia multiple category cash transfer programme. MEX=Mexico. MINI=Mini International Neuropsychiatric Interview. MWI=Malawi. NLD=Netherlands. NIC=Nicaragua. NPL=Nepal. NWS-PTSD=National Women’s Study-Post-traumatic Stress Disorder module. NZL=New Zealand. PSS=Perceived Stress Scale. SF-MCS=Mental Component Summary of Short Form Survey. SWE=Sweden. TWN=Taiwan. UGA=Uganda. WHOQOL=psychological domain of abbreviated WHO Quality of Life tool. ZAF=South Africa. ZMB=Zambia. ZWE=Zimbabwe. *Study was a randomised controlled trial. †Sex-stratified results for women. ‡Welfare policy only affected income. §Sex-stratified results for men. ¶Stratified results for school dropouts. ||Stratified results for school attenders. **Welfare policy influenced income and other factors. ††Stratified results for those of high educational attainment. ‡‡Stratified results for those of low educational attainment.

On meta-analysis, a binary income increase was associated with a 0·084 SD improvement in mental health measures (95% CI 0·038 to 0·130, *I*^2^=80%, indicating high heterogeneity). This was based on 32 studies analysing 258 637 people (figure 2A), with most exposures influencing unearned income and 40·6% of studies (n=13) rated serious RoB. Eight RCTs and five non-randomised studies (40·6% of all studies) included at least one important co-intervention for those exposed that could influence mental health, such as education, training, or work incentives (appendix p 28). Meta-regression suggested effect sizes were larger where income changes moved individuals across a poverty threshold (β 0·11, 95% CI 0·03 to 0·19) or targeted those of low SEP (0·08, –0·01 to 0·17]), or in LMICs (0·07, −0·02 to 0·15; appendix p 91). Considering income source, only welfare policies not solely targeting income had differential, slightly larger effects (β 0·18, 95% CI 0·02 to 0·34). In stratified analyses (appendix pp 97–104), effect sizes were strikingly larger for poverty transitions (SMD 0·134, 95% CI 0·070 to 0·198 *vs* 0·011, 0·002 to 0·019 for other income changes; p<0·00017 for test of differences), as well as in LMICs (SMD 0·122, 95% CI 0·041 to 0·203 *vs* 0·030, 0·007–0·053 in HICs; p=0·033). Critical RoB studies reported effect sizes six times larger than other studies: SMD 0·507 (95% CI 0·163 to 0·851) versus 0·087 for both low or moderate RoB (0·030 to 0·144) and serious RoB (–0·001 to 0·175; p=0·061; appendix p 99). Effect sizes also appeared larger in RCTs than in non-randomised studies (SMD 0·130, 95% CI 0·047 to 0·213 *vs* 0·043, 0·012 to 0·074; p=0·054) and for studies that only included those of working age (0·118, 0·056 to 0·181 *vs* 0·035, –0·016 to 0·087; p=0·044). There were no important sex differences (appendix p 97).

A binary income decrease was associated with a 0·213 SD worsening of mental health measures (95% CI –0·301 to –0·125; *I*^2^=84%), two and a half times the magnitude for income increases. This was based on eleven non-randomised studies analysing 227 804 people (figure 2B), with income source mostly unknown, and all but one rated serious RoB. Meta-regression suggested larger effect sizes where the income change was due to a disaster (β –0·48, 95% CI –0·88 to –0·07), although only one study reported this exposure;^61^ no other differences were seen by income source. In stratified analyses (appendix pp 105–107) effect sizes were again larger in critical (SMD –0·346, 95% CI –0·449 to –0·244]) than in serious RoB studies (–0·185, –0·264 to –0·107; p=0·012). There was no evidence of important sex or age differences.

The pooled standardised β for a log (income) change on continuous mental health outcomes was 0·027 (95% CI 0·003 to 0·052; *I*^2^=81%), indicating a 10% income increase would be associated with a 0·003 SD improvement in mental health. This was based on nine non-randomised studies analysing 1 510 221 people (figure 2C), with income source again mostly unknown and all but one study rated serious RoB. Two low RoB Swedish lottery studies unfortunately could not be included in the synthesis as income was not log-transformed within their modelling, but they also reported a small beneficial effect of winning on mental health.^18,19^ On stratification (appendix p 107–109) there was some evidence effects were larger in women (SMD 0·090, 95% CI 0·031 to 0·149) than men (0·047, –0·035 to 0·129), although this was based on few studies. There was no clear evidence of important SEP or age differences.

The pooled effect for five non-randomised studies considering the effect of log (income) change on binary mental health measures was OR 0·967 (95% CI 0·919–1·016; *I*^2^=53%), with sex-stratified analysis finding no clear differences (appendix p 119).

For the wellbeing outcome domain, the effect direction in all 64 studies considering wellbeing outcomes is shown in an effect direction plot in the appendix (p 96). After excluding those with inconsistent findings and at critical RoB, 95·0% reported a beneficial effect (95% CI 83·1–99·4, n=40, sign test p<0·0001). On χ^2^ testing there was no evidence of differences by RoB (p=0·63), study design (p=0·68), or income source (p=0·92).

**Figure 3:**
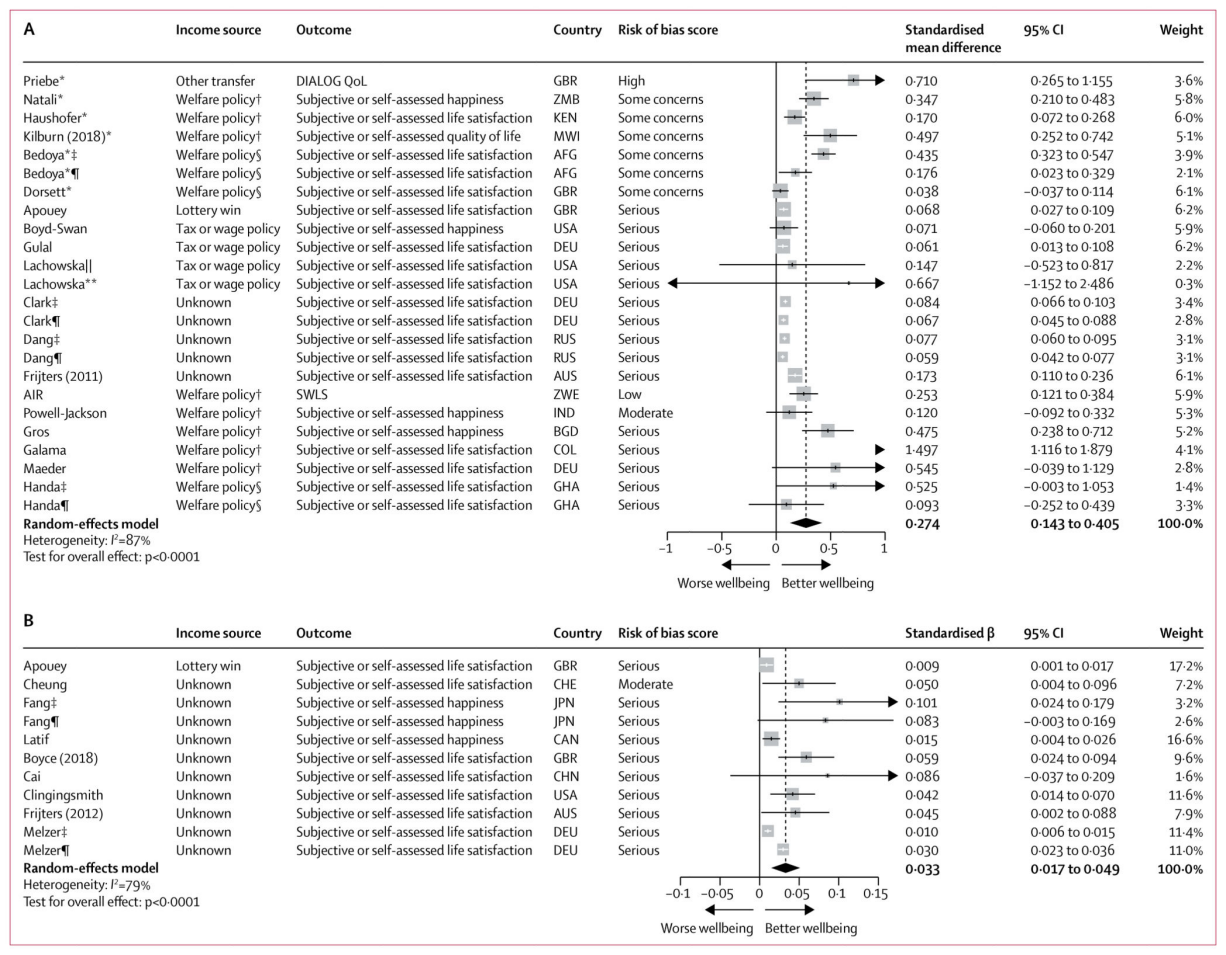
Forest plots for meta-analyses of studies reporting the effect of a binary income increase on a wellbeing outcome (A; n=19) and a continuous log (income) change on a wellbeing outcome (B; n=9) In A, number of people=163 969 and number of observations=885 981. In B, number of people=105 326 and number of observations=567 356. In both panels, studies are sorted in order of trial status (randomised controlled trial or non-randomised study), income source, outcome, and risk of bias score. AFG=Afghanistan. AIR=American Institutes for Research. AUS=Australia. BGD=Bangladesh. CHE=Switzerland. CHN=China. COL=Columbia. DEU=Germany. DIALOG QoL=DIALOG quality of life scale. GBR=Great Britain. GHA=Ghana. IND=India. JPN=Japan. KEN=Kenya. MWI=Malawi. RUS=Russia. SWLS=Diener’s Satisfaction with Life Scale. ZMB=Zambia. ZWE=Zimbabwe. *Study was a randomised controlled trial. †Welfare policy only affected income. ‡Sex-stratified results for women. §Welfare policy influenced by income and other factors. ¶Sex-stratified results for men. ||Stratified results for those on medium income. **Stratified results for those on low income.

On meta-analysis, a binary income increase was associated with a 0·274 SD improvement in wellbeing measures (95% CI 0·143 to 0·405; *I*^2^=87%), three times that seen for mental health. This was based on 19 studies analysing 163 969 people (figure 3A), with most exposures originating from unearned income as in figure 2A, and 63·2% of studies (n=12) rated serious RoB. Three RCTs and three non-randomised studies (31·6% of all studies) had important co-interventions (appendix p 28). On meta-regression, only welfare policies affecting solely income potentially had larger effects (β 0·33, 95% CI –0·01 to 0·68). In stratified analyses (appendix p 110–116), effect sizes were larger for poverty transitions (SMD 0·377, 95% CI 0·093 to 0·661 v*s* 0·160, 0·069 to 0·252]; p=0·16) and in LMICs (0·371, 0·153 to 0·589 *vs* 0·087, 0·052 to 0·122; p=0·012). Studies at serious rather than low or moderate RoB reported slightly larger effects (0·315, 0·089 to 0·540 *vs* 0·245, 0·137 to 0·353; p=0·83) but estimates were imprecise. Effect sizes for women were larger than for men (0·195, 0·080 to 0·309 *vs* 0·063, 0·050 to 0·077; p<0·0046). There was no evidence of important differences when stratifying by study design or age.

There were insufficient non-critical RoB studies considering the impact of a binary income decrease on wellbeing outcomes to perform meta-analysis (n=3). However, in a sensitivity analysis including critical RoB studies (appendix p 117), the effect size was slightly larger in critical RoB studies (SMD –0·278, 95% CI –0·378 to –0·178 *vs* –0·195, –0·317 to –0·073; p=0·30) with wide CIs.

The pooled standardised β for a log (income) change on continuous wellbeing outcomes was 0·033 (95% CI 0·017–0·049; *I*^2^=79%), indicating a 10% income increase would be associated with a 0·003 SD improvement in wellbeing, a similar effect magnitude as for mental health. This was based on nine non-randomised studies analysing 105 326 people (figure 3B), with income source again unknown in most cases and all but one study rated serious RoB (88·9%). Again, a low RoB lottery study could not be included in meta-analysis but also reported a small significant effect of lottery wins on life satisfaction.^19^ There was no clear evidence of important age or sex differences (appendix pp 117–118).

For common outcome measures, we used variance or SDs reported in other included studies to facilitate data transformations where these were not reported for the study sample (n=7;^46,47,82,92,97,100,139^ appendix p 5). To ensure this did not introduce bias we re-ran meta-analyses excluding these studies, and there was no change to the overall pattern of the findings (appendix pp 120–123).

Funnel plots and Egger’s test results indicated some evidence of publication bias or small study effect in our primary meta-analyses (appendix pp 123–125). This was particularly striking for studies considering income decreases and mental health outcomes (p<0·0001), and continuous income changes and wellbeing outcomes (p=0·027).

Overall, the evidence base showed substantial limitations when assessed according to the GRADE criteria (table and appendix p 93).^38^ We report with low certainty that income changes have beneficial effects on mental health and wellbeing (ie, an income increase is associated with improvement while an income decrease is associated with worsening). Our estimates of effect size for key outcomes were subject to low or very low certainty.

## Discussion

Our comprehensive systematic review and meta-analysis, covering a broad range of income change sources, contexts, and settings, found that income has beneficial effects on mental health and wellbeing: income increases resulted in a 0·08 SD (95% CI 0·04 to 0·13) improvement in mental health measures and 0·27 SD (0·14 to 0·41) improvement in wellbeing measures. In stratified analyses, effects were up to 13 times larger where increases moved individuals out of poverty, and up to four times larger for those in LMICs. There was no consistent evidence that effect size was influenced by income source. Effects from studies reporting continuous income were smaller than those considering binary income changes, possibly because these could not incorporate any threshold effect of poverty. Effects might be larger for income decreases, which were associated with a 0·21 SD worsening of mental health measures (95% CI –0·30 to –0·13). Certainty for all key outcomes was low or very low due to high heterogeneity, considerable risk of bias, unmeasured confounding, and inclusion of cointerventions such as conditionality or non-monetary benefits.

The relationship between income and mental health has been subject to detailed study over many decades. However, previous evidence syntheses have been subject to limitations. Cooper and Stewart^23^ published a narrative review in 2015 concluding income has a positive effect on mental health, but did not attempt to quantify this relationship or explore why effects diverge across the literature. A more recent non-systematic review by Ridley and colleagues^25^ examined the effect of anti-poverty interventions on mental health, reporting a similar effect size to our own (SMD 0·09) despite excluding non-randomised studies. McGuire and colleagues^24^ in their meta-analysis focus on cash transfers in LMICs and do not assess certainty, but also report similar effect sizes. The added value of our study is in summarising evidence from a broader range of exposures, considering how effects differ by exposure characteristics and between groups, and robustly assessing RoB and certainty of the evidence base to aid in causal interpretation, providing meta-analysed effect estimates showing a small if uncertain effect of income change on mental health.

The larger effect sizes found in our study for income increases targeting the most socioeconomically disadvantaged were not unexpected.^15,23^ It is difficult to determine which of the three most consistent effect modifiers we identified (poverty transitions, lower SEP, or LMIC setting) is most important, due to considerable overlap of studies falling into two or three of these categories. Our finding of a larger effect size for wellbeing outcomes was in keeping with those of McGuire and colleagues;^24^ we do note that most studies reported single subjective wellbeing items (eg, life satisfaction or happiness), which are arguably more biased than multi-dimensional wellbeing measures.^173,174^

Existing literature on loss aversion suggests financial losses could have greater effects on mental health than financial gains,^63^ although evidence is inconsistent.^175^ The very low certainty we report in this finding due to potential publication bias does not resolve this debate, nor will it be easy to settle since reducing incomes cannot be ethically tested in trials. Here, future research might need to rely on natural experiment approaches, ideally with pre-registered analysis plans to ensure dissemination of negative findings.^176^ Triangulation across methodologies could help overcome the limitations and trade-offs required by each individual approach to determining causal inference, in particular where this draws on cross-disciplinary learning.^177^

Finally, to contextualise our results within wider literature on common mental health interventions, in comparable meta-analyses on treatments for mental ill health pooled effect sizes for antidepressant usage and cognitive behavioural therapy (CBT) were SMD 0·30 and SMD 0·53, respectively.^178,179^ This implies that, based on our findings, income-based interventions that move people above the poverty line might be roughly half as effective in improving mental health as antidepressants and a quarter as effective as CBT. Given that, in contrast with our included studies, these treatments are primarily studied in individuals at high-risk rather than general population samples, the potential impacts of anti-poverty interventions at a population mental health level could be substantial.

Our study has important strengths. To our knowledge, this is the first review to quantitatively summarise the totality of the evidence base on income changes and mental health or wellbeing. We closely followed gold standard Cochrane guidance on conducting and reporting systematic reviews of interventions. Using ROBINS-I we were able to include non-randomised studies and rigorously assess these against the same standard for threats to causal interpretation as RCTs. We pre-registered our study protocol and made only minimal changes (appendix p 2). Our decisions about key confounding variables and stratified analyses were informed by a comprehensive literature review and preparation of a logic model (figure 1), ensuring our assumptions about proposed causal mechanisms were clear.

However, some important limitations remain. First, some relevant economics working papers might have been missed by our search methods despite efforts to avoid this. Second, the heterogeneity of included studies is very high. Combining RCTs and non-randomised studies in the same meta-analyses (which is not standard practice) could have contributed to this, although we did not find marked differences in heterogeneity when stratifying by study design. We feel on balance the benefits of including non-randomised studies (which often evaluate real-world, large-scale natural policy experiments) probably outweighed potential drawbacks. Third, although ideally synthesis of income change exposures would be expressed in terms of absolute or relative change, data availability and reporting meant we were unable to do this with our binary exposures. Our sensitivity analyses considering effects of small versus large changes and poverty transitions explored some heterogeneity by intervention size, but possibly not all. Excluding non-English language texts is also a limitation, although we note only 12 records were excluded solely for this reason (PRISMA flowchart; appendix p 18). Finally, although not unanticipated, the relatively high RoB of included studies means caution should be taken in interpretation, as shown by our GRADE certainty assessments.

In terms of policy implications, our findings add to the evidence base supporting income supplementation as a means to improve mental health and wellbeing, especially when targeted at those in poverty or in LMICs. Although effect sizes are small, they are comparable with common individual-level interventions such as antidepressants or CBT. Our findings also suggest a particularly detrimental effect of income decreases, indicating policy makers wishing to protect population mental health should mitigate against societal or economic changes that could erode income. Decision makers and policy modellers might benefit from use of our results when calculating potential mental health impacts of future policies that will change incomes.

Future intervention studies of cash transfers should consider including more robust, multi-dimensional measures of wellbeing. There is a need for studies reporting the impact of income change on a continuous scale that consider the question in a causal or counterfactual way, as well as studies considering longitudinal effects of living on a low income. The low certainty of our GRADE assessments highlights both that non-randomised studies considering this topic area frequently do not appropriately adjust for known key confounding variables (particularly past mental health), and that RCTs often include cointerventions which create difficulties in measuring the effect of income alone. To improve certainty in future estimates, researchers should consider how best to reduce these threats to causal inference during study planning.

Given our focus on individual-level exposures and outcomes, future systematic reviews considering the quantitative impact of relative income or community-level outcomes would be welcome, as would further analyses considering whether and how the benefits of income are augmented when accompanied by co-interventions. Finally, in our logic model (figure 1) we identified some potential effect modifiers (eg, ethnicity and income inequality) we were unable to explore in detail in our synthesis due to lack of data; future research on this topic could benefit from use of our logic model when planning analyses to prespecify potentially important variables for stratification.

Our findings suggest that income probably does have a causal effect on mental health and wellbeing despite weaknesses in the evidence base, and this relationship is stronger for those in poverty or in LMICs. To be most supportive of population mental health and reduce inequalities, policy makers should design income and welfare policies that provide an adequate financial safety net for the most socioeconomically disadvantaged. There would be considerable value in future research investigating the mechanisms linking income and health, and the incorporation of these findings into intervention planning and evaluation.

## Supplementary Material

Online supplement

## Figures and Tables

**Table:** Condensed summary of findings and certainty of evidence (as per GRADE)

	Number of participants (number of studies)	Effect estimate (95% CI)	Certainty of evidence
**Income changes and grouped mental health outcomes**			
Any income change (effect direction)	922 428 (54)	88·9% (77·4 to 95·8) of studies report beneficial effect of income on mental health	Low*
10% income increase	1510 666 (9)	Standardised β 0·003 (0·0003 to 0·005)	Low*
Crossing poverty or subsistence threshold	42 128 (18)	SMD 0·13 (0·07 to 0·20)	Low†
Income decrease (mixed amounts)	227 804 (11)	SMD –0·21 (–0·30 to – 0·13)	Very low‡
**Income changes and grouped wellbeing outcomes**			
Any income change (effect direction)	311 219 (40)	95·0% (83·1 to 99·4) of studies report beneficial effect of income on wellbeing	Low*
10% income increase	105 326 (9)	Standardised β 0·003 (0·002 to 0·005)	Very low‡
Crossing poverty or subsistence threshold	101 350 (8)	SMD 0·38 (0·09 to 0·66)	Low*
Income decrease (mixed amounts)	Insufficient evidence§		
Changes in unearned income and all outcomes	Insufficient evidence§		

GRADE=Grading of Recommendations Assessment, Development and Evaluation. SMD=standardised mean difference. For full GRADE results, see the appendix (p 93).

* Downgraded by 2 levels for inconsistency and risk of bias of included studies.

† Downgraded by 2 levels for inconsistency and indirectness of evidence.

‡ Downgraded by 3 levels for inconsistency, risk of bias of included studies, and publication bias.

§ Insufficient numbers of studies identified to investigate this planned GRADE outcome.

## Data Availability

The study protocol is published on PROSPERO. All extracted data used for analysis and analytic code are available in a public GitHub repository. Template data forms and copies of the totality of data extracted from included studies are available from the corresponding author on reasonable request.

## Research in context

### Evidence before this study

Those on low incomes are known to have poorer mental health and wellbeing than those who are wealthier. However, there is debate in the literature around the extent to which this represents a causal relationship, rather than being solely the result of confounding or reverse causation. Before conducting this study, we searched MEDLINE, Google Scholar, and PROSPERO to identify existing or planned English-language systematic and non-systematic reviews of the relationship between income and health outcomes in adults, with no date limits; we repeated these scoping searches on Nov 24, 2021. Search terms included health, “mental health”, depression, anxiety, wellbeing AND income (or synonym). Existing reviews have either suggested a potential association between income changes and mental health without attempting to quantify this potential relationship or considering risk of bias within the literature, or have focused on narrow definitions of exposures in restricted settings.

### Added value of this study

In this systematic review and meta-analysis, integrating Cochrane risk of bias tools designed to assist in interpretating threats to causality, income changes were associated with small but consistent effects on subsequent mental health and wellbeing outcomes in working-age adults (aged 16–64 years). Effect sizes were largest where increases crossed a poverty threshold. To our knowledge, this is the first comprehensive systematic review to quantify effect sizes through meta-analysis and rigorously assess causality and certainty of evidence across the breadth of literature on this topic.

### Implication of all the available evidence

Income changes probably have a causal effect on the mental health of working-age adults, although certainty in the effect size is low due to heterogeneity and risk of bias. Welfare policy makers wishing to improve public mental health should prioritise lifting individuals out of poverty.
