# Supplementary material for: How do income changes impact on mental health and wellbeing for working-age adults? A systematic review and meta-analysis
Source: Lancet Public Health. Author manuscript; Available in PMC 2023 Aug 4. (PMC7614874; doi:10.1016/S2468-2667(22)00058-5)

THE LANCET
Public Health

Supplementary appendix

This appendix formed part of the original submission and has been peer reviewed. We post it as supplied by the authors.

Supplement to: Thomson RM, Igelström E, Purba AK, et al. How do income changes impact on mental health and wellbeing for working-age adults? A systematic review and meta-analysis. *Lancet Public Health* 2022; **7:** 515–28.

How do income changes impact on mental health and wellbeing for working-age adults? A systematic review and meta-analysis

Rachel M Thomson, Erik Igelström, Amrit Kaur Purba, Michal Shimonovich, Hilary Thomson, Gerry McCartney, Aaron Reeves, Alastair Leyland, Anna Pearce, S Vittal Katikireddi

Supplementary Appendices

Appendix A: Additional information on protocol and process 2

Table A1: Data items extracted from included studies........ 6

Appendix B: Details of search strategy 14

Appendix C: PRISMA flowchart 18

Appendix D: Details of included studies 19

List of 136 included studies........ 19

Table D1: Included studies and their characteristics. 28

Table D2: Risk of bias (RoB) domain scores for included non-randomised studies, measured using ROBINS-I........ 63

Table D3: Risk of bias (RoB) domain scores for included randomised controlled trials, measured using ROB-2........ 68

Appendix E: List of studies excluded at full text 69

Appendix F: Additional tables 91

Table F1: Results of meta-regression for all main analyses. 91

Table F2: GRADE summary of findings and certainty of evidence for each outcome domain 93

Appendix G: Additional graphs 96

Effect direction plots........ 96

Stratified forest plots for main subgroup analysis........ 97

Additional analysis using binary outcomes........ 119

Sensitivity analysis excluding calculations which used an external standard deviation 120

Funnel plots and Egger's test investigating publication bias/small study effect........ 123

Appendix A: Additional information on protocol and process

Clarifications to and deviations from protocol as published

1. Clarified that books and book chapters were excluded
2. Clarified that exposures of interest should represent changes in *absolute* rather than relative income (the only exception to this was movements into/out of poverty, due to research interest in the threshold effect of this transition)
3. Clarified that changes in subjective financial strain/stress alone were not considered appropriate exposures where it was unclear whether these were accompanied by absolute income change
4. Clarified that studies of universal basic income/minimum income guarantee interventions were eligible for inclusion when they included a comparison between those who did and did not experience an income change following the introduction of the intervention
5. Clarified that health-related quality of life measures were excluded
6. Clarified that psychological variables (e.g. self-efficacy) were excluded
7. Clarified that area-level outcomes (e.g. suicide rates) were excluded
8. As more studies than expected considered binary exposures we were able to meta-analyse these, and therefore took the decision (based on existing literature) to consider increases and decreases separately, and to consider transitions into/out of poverty within subgroup analysis
9. Meta-regression rather than subgroup analysis was used as the main approach to investigate effect modification after separating out continuous/binary increase/binary decrease exposures – it had been intended a priori that the analysis would include some element of meta-regression if possible, but it was not anticipated there would be sufficient datapoints to do this to the extent to which we were able
10. GRADE certainty assessments were not reported for two pre-specified critical outcomes (mental health/wellbeing outcomes secondary to changes in unearned income) due to the structure of extracted data, with most analyses including *only* changes in either earned or unearned income

Assignment of outcomes to mental health vs wellbeing domain

Outcomes which were indicators of serious mental illness (e.g. psychosis), common mental health problems (e.g. depression, anxiety) or general mental health (e.g. self-harm, composite symptom scores such as SF-12) were grouped into the mental health domain. Mental health outcomes measured at area-level (e.g. suicide rates) and outcomes related to substance use were not eligible for inclusion. Outcomes which were indicators of life satisfaction, happiness, or quality of life were grouped into the wellbeing domain.

Primary allocation of outcomes to domains occurred at data extraction and was subsequently independently checked by a second reviewer, with any unclear decisions resolved by consensus. In one study (Chin 2018) an outcome nominally described as a quality of life measure (the Taiwanese version of WHOQOL-BREF) was categorised as a mental health measure, as the outcome extracted was the psychological domain score only, rather than the overall quality of life score.

Decision rules for selecting between studies with overlapping data

These questions were designed to assess both the closeness to our study PECOS and also a crude assessment of risk of bias.

Most important criteria (given equal weight)

- How large is the sample size?
- Does the study report longitudinal rather than cross-sectional data?

- Do the research question(s) and sample correspond well to our own:
    - In terms of the included population?
    - In terms of the exposure/outcome combination?
- How well does the study address confounding in relation to our research question in its design and analysis?
- How well would the data reported facilitate inclusion in a meta-analysis?
- Is the study peer-reviewed?

<u>Remaining criteria to be used if no clear decision after applying the above (in order of importance)</u>

- How recent are the data used?
- How many years of data does the study use, if longitudinal?

Decision rules for selecting which outcome measure to include from a study with multiple options

Where studies report more than one mental health outcome:

1. Use validated rather than subjective/unvalidated measure if available
2. Use measures of depression (most common) where more than one option

Where studies report more than one wellbeing measure:

1. Use validated rather than subjective/unvalidated measure if available
2. Use measures of life satisfaction (most common) where more than one option

Where multiple measures of an outcome are reported:

1. Use last occasion measured UNLESS this occurs after the intervention could conceivably have exerted its main effect on our outcome of interest
2. In the latter case, opt for the measurement closest to the end of the intervention

Further detail on process for assessing risk of bias

Key confounders for ROBINS-I were determined a priori: age, sex, ethnicity, other measures of socioeconomic position excluding income (e.g. educational attainment, area-level deprivation, benefit status, housing tenure), employment status, household composition, physical health, and past mental health. Where multiple adjusted effect estimates were reported in a study, the estimate which most closely matched our ideal confounding strategy was selected. As some of our identified key confounders could also function as mediators if they occurred after the exposure of interest, the timing of measurement was also taken into account in ROBINS assessment under the confounding domain.

Anticipated common co-interventions which could differ between intervention groups, and therefore impact on outcomes, were also listed a priori. Examples included: any direct protective effects of secure employment vs insecure employment vs unemployment on mental health; any mental health effects of conditions attached to a policy e.g. conditional cash transfers; increased or decreased access to healthcare e.g. through work-related insurance; contemporaneous policy changes which did not impact on income but may have impacted on mental health outcomes e.g. housing quality standards.

Each non-randomised study (NRS) was scored across the seven ROBINS-I domains (confounding, selection, classification, deviations, missingness, outcome measurement, and reporting) in

accordance with the ROBINS-I detailed guidance.[1] The overall score for the study was taken from the highest/most biased domain-level RoB rating. In interpreting ROBINS-I ratings, a 'low' overall rating is considered comparable to a well-conducted RCT in assessing the true effect of the intervention, whereas a 'moderate' rating indicates that the study is sound for an NRS, but cannot be considered comparable to an RCT. A 'serious' rating indicates that the study has some important problems in a domain (for example, the omission of any pre-specified key confounder from analysis), but may still provide useful information for synthesis. In contrast, a 'critical' rating indicates that a study is too problematic in a domain to provide any useful evidence on the effect of interest (for example, a judgement that due to the study design or analytical choices confounding was 'inherently not controllable').

When stratifying analysis by RoB, RCTs rated 'some concerns' on RoB-2 were grouped with 'moderate' NRSs and 'high' RCTs were grouped with 'serious' NRSs. The percentage agreement for RoB assessment was 60.3%.

Process for data transformations for meta-analysis

All data transformations were conducted according to guidance within the Cochrane Handbook[2] and, where necessary, using an online effect size calculator by The Campbell Collaboration.[3] For binary/dichotomous exposure measurements, continuous and dichotomous outcome data were combined. We opted to report these as standardised mean differences (SMDs) as most outcomes were originally reported on continuous scales, and therefore fewer assumptions were made when completing the conversions. This meant that where studies reported on the same outcome measure both continuously and using a binary 'cut-off', we utilised the former estimate in our synthesis. For continuous exposure measurements – which are rarely reported in systematic reviews, meaning best practice recommendations are not available within the Cochrane Handbook – we opted *not* to combine continuous and dichotomous outcome data. This decision was taken (after discussion with systematic review and statistical experts) because the common statistical methods available to combine these are based on the ability to make direct comparisons between two distinct exposed and unexposed groups, and we therefore did not feel it was appropriate or interpretable to utilise these for continuous exposures. Instead, we pooled these data separately as either standardised beta coefficients or odds ratios.

Where studies reported that a p-value was <0.05 without reporting the exact value and this was required to determine the standard error of an estimate, the p-value was conservatively assumed to be 0.05 as per Cochrane to facilitate inclusion in meta-analysis. Where studies reported probit rather than logit coefficients, these were multiplied by 1.81 prior to conversion to SMD.[4] Where studies reported the output of a linear probability model (i.e., the percentage point difference in prevalence of a binary outcome between exposure groups), where possible SMDs were approximated by applying this absolute difference to an estimate of prevalence in the study control group if this was reported. Where studies using longitudinal data reported only the number of person-year

---

[1] Sterne JAC, Higgins JPT, Elbers RG, Reeves BC and the development group for ROBINS-I. Risk Of Bias In Non-randomized Studies of Interventions (ROBINS-I): detailed guidance, updated 12 October 2016

[2] Higgins JPT, Thomas J, Chandler J, Cumpston M, Li T, Page MJ, Welch VA (editors). Cochrane Handbook for Systematic Reviews of Interventions version 6.2 (updated February 2021). Cochrane, 2021. Available from www.training.cochrane.org/handbook.

[3] https://www.campbellcollaboration.org/escalc/html/EffectSizeCalculator-Home.php

[4] https://aniruhil.github.io/avsr/teaching/mpa6020/LogitandProbitModels2.html

observations without including the number of individuals, this was conservatively estimated by dividing the number of observations by the number of waves of data.

Study investigators were contacted by email where data were insufficiently reported for standardisation or transformation, and in a small number of cases (n=7) we utilised variance/standard deviations reported in other included studies for common outcome measures where these were not reported for the study sample. We performed sensitivity analysis excluding these studies to ensure this did not bias our findings.

Planned meta-analyses which could not be completed due to insufficient data

1. For binary income decreases and mental health outcomes (Figure 3B), could not stratify by study design, socioeconomic position, poverty transitions or setting
2. For continuous income changes and continuous mental health outcomes (Figure 3C), could not stratify by risk of bias, study design, poverty transitions or setting
3. For continuous income changes and binary mental health outcomes (Figure G22), could not stratify by risk of bias, study design, socioeconomic position, poverty transitions, setting or age
4. For binary income decreases and wellbeing outcomes there were insufficient studies to complete any primary meta-analysis
5. For continuous income changes and continuous wellbeing outcomes (Figure 4B), could not stratify by risk of bias, study design, socioeconomic position, poverty transitions or setting
6. For continuous income changes and binary wellbeing outcomes there were insufficient studies to complete any meta-analysis
7. Meta-regression could not be completed for any analysis of continuous income changes as these included fewer than 10 studies

Table A1: Data items extracted from included studies

STUDY LEVEL VARIABLES			
**Field**	**Brief description**	**Guidance**	**Required format/permissible entries**
**source**	Where did we find the study?	If this is a relevant study screened in Covidence	Cov
		If this is a relevant study identified in a Systematic Review	SR
		If this is a relevant study identified from another included study	INT
		If this is a relevant study identified from another source	EXT
**source2**	If relevant, which other paper was the source of the study?	Source = Covidence i.e. initial searches	n/a
		Source = Systematic Review	First author surname and year of publication
		Source = another included study	Internal reference name of that paper ('study' column)
		Source = other e.g. communication with expert	Correspondent surname and other descriptor e.g .PhD
**id**	Internal reference number	Source = Covidence i.e. initial searches	Covidence ID number
		Source = Systematic Review	SR# (# = original number)
		Source = another included study	INT#
		Source = other e.g. communication with expert	EXT#
**study**	Internal reference name		First author's surname and year of publication
**first_author**	Surname of first author		
**year_pub**	Year of publication		
**title**	Title of study		
**where_published**	Where was the study published?	If study published in a journal	Name of journal
		If study published by organisation e.g. University, World Bank	Name of organisation

**publication_cat**	What type of publication is this?		Journal
			Working paper
			Report
			PhD thesis
			Other peer-reviewed
**randomised**	Is the study randomised?		Randomised
			Not randomised
**length**	Does the study include repeated measures on the same individuals?	No repeated measures	Cross-sectional
		At least two waves of data collection on same individuals	Longitudinal
**int_assignment**	What determines whether an individual receives our intervention of interest? (income change)	Determined by a researcher or trial design e.g. randomisation	Researcher/trial
		Determined directly by a policymaker	Policymaker
		Determined entirely by chance	Chance
		Determined by the person's location	Location
		Determined by a choice made by the person	Individual choice
		Determined by some characteristics of the person e.g. SES, health	Individual characteristics
		Any other clear factor not on the list	Other
		It is not possible to determine why the person's income changed	Unclear/unknown
**objective**	What is the aim of the study, as stated by authors?		
**country**	In which country/countries did the study take place?		
**setting**	What World Bank category was this country in at the time of the study?		High income
			Middle income
			Low income
**study_years**	What years did the study take place?	Can be expressed as either a range or list (if exclude certain years)	

**fu_length**	How many years of follow up were there (for our question of interest)?	If cross-sectional study	n/a
		If longitudinal study	Number of years
**data_source**	Brief description of data source		
**data_source_cat**	Are the data primary or secondary?		Primary data
			Secondary data
**study_design**	What is the design of the study in relation to our research question?	Longitudinal data, not a trial, no natural experiment (NE) elements	Basic longitudinal
		Cross-sectional data, not a trial, no NE elements	Basic cross-sectional
		Repeat cross-sectional data, not a trial, no NE elements	Repeat cross-sectional
		Pre-specified trial of an intervention, with randomisation	Randomised trial of intervention
		Pre-specified trial of an intervention, without randomisation	Non-randomised trial of intervention
		Pre/post intervention study with control group e.g. NE using DiD or longitudinal data on same individuals	Before and after (controlled)
		Pre/post intervention study without control group	Before and after (no control)
		Any other natural experiment not described here	Other natural experiment
**sampling_frame**	What was the population that was eligible for initial recruitment into **the whole sample**?	Brief description covering inclusion criteria for initial data collection e.g. trial, cohort study	
**excl_criteria**	What excluded someone from being part of **our sample of interest?**	List of reasons someone would be excluded from the sample in which we are interested (may be a subset of the whole sample)	
**total_participants**	Number of participants in our sample of interest		
**average_age**	Mean age in our sample of interest	**If not possible to extract for our sample of interest, extract for whole sample. If reported by subgroup e.g. by sex, extract overview of information where possible unless this would entail large amounts of data (applies to all demographic columns)**	Report mean and SD if available
**age_range**	Age range in our sample of interest	If not reported, indicate whether likely to be working age (16-65) based on sampling frame	
**percent_male**	Percentage of our sample of interest which is male		

**sep**	Socioeconomic breakdown of our sample of interest	If possible extract education or other relevant individual indicator of SEP.	
**ethnicity**	Ethnic breakdown of our sample of interest	Nationality/country of origin (e.g. immigrant status) not sufficient	
**int_type**	What is the reason for/source of the income change?<br><br>NOTE: this is our judgement based on the identification strategy/ exposure measurement e.g. an income change simply occurring during a time of recession should be noted 'HH income change (reason unknown)' unless people were explicitly asked if their income fell *due to* the recession	Cannot definitively identify cause of income change	HH income change (reason unknown)
		Income received from lottery win	Lottery win
		Income received from benefits after using advice service	Income maximisation/benefits advice service
		Change resulting from welfare policy only affecting cash	Cash transfer only
		Change resulting from other welfare policy	Other welfare policy
		Change resulting from taxation/wage policy	Taxation/wage policy
		Change explicitly described as change in salary (not due to policy)	Salary change
		Change explicitly described as due to natural or unnatural disaster e.g. flood, terrorist attack	Natural/Unnatural disaster
		Change explicitly described as due to illness or caring responsibilities	Illness/caring
		Change explicitly described as due to marriage breakdown	Marriage breakdown
		Change explicitly described as due to macroeconomic event	Macroeconomic event
		Income received from cash transfer from any another source	Cash transfer (other source)
**int_def**	Description of the intervention/income change		
**income_def**	How is income defined within the study?	e.g. individual/household, gross/net, if equivalised, what currency	
**int_meas_cat**	How is the income change measured?	If reported by respondent or proxy	Self-report
		If deduced from administrative data	Admin data
		If self-evident from trial data e.g. randomisation status	Trial data
		If estimated or modelled from other available data	Estimated/modelled
**int_duration**	How long did the intervention last for?	Also mention here if there was a delay between intervention ceasing and data collection	
**int_mean**	Mean value of the intervention measure (if applicable)		
**int_sd**	SD of the intervention measure (if applicable)		

**comparator**	Who/what is the comparator?	e.g. self, control group	
**outcome_domain**	Which of our two broad outcome domains does the study report on?		Mental health
			Wellbeing
			Both
**outcome_type**	Which subcategory does the outcome report on? (Should be based on our view of measure, not what study calls it)	If uses measure of mental health e.g. depression, anxiety, stress (NOT substance use)	Mental health
		If asks about positive/negative affect, general wellbeing scales	Wellbeing
		If asks about satisfaction with life (NOT individual aspects e.g. work)	Life satisfaction
		If asks about happiness (NOT affect)	Happiness
		If uses formal quality of life scale (NOT health-related QoL)	Quality of Life
**outcome_def**	How is the outcome defined within the study?	Can include information on how scores are calculated here, particularly if different from standard use of measure	
**outcome_meas_cat**	How is the outcome measured?	If simple survey question rather than tool	Self-report - Survey Q
		If a validated tool is used as intended (or alterations justified)	Self-report - validated tool
		If ascertained from administrative data	Admin data
		If ascertained from clinical interview	Clinical interview
**outcome_duration**	How long did the outcome last for/how long is it measure over?	If cross-sectional measurement rather than other time = n/a	
**outcome_mean**	Mean of the outcome measure(s) (if applicable)		
**outcome_sd**	SD of the outcome measure(s) (if applicable)		
**analysis_type**	What type of analysis was performed?	Simple regression modelling +/- adjustment for confounders	Standard regression
			Fixed/Random effects model
			Interrupted time series
			Difference in difference
			Instrumental variable analysis
			Propensity score matching
			Structural equation modelling

		Any other NE method aimed at assessing causality not listed	Other causal method
			Correlation
			Chi squared
			t-test
		e.g. ANOVA	Other
**analysis_desc**	Description of the analysis performed	Include type of regression e.g. linear, probit	
**conf_controlled**	What confounding variables were adjusted for?		
**mediators_or_ modifiers**	Were any mediators or moderators investigated?	Focus on those relevant to our question	

ADDITIONAL DATAPOINT LEVEL VARIABLES

Field	Brief description	Guidance	Permissible entries
**gender**	Sex/gender of participants for this datapoint	Only men	Male
		Only women	Female
		Includes men and women	Both
**age**	Does this sample only include working age population?		Working age only
			Includes <16s or >64s
**study_design**	What is the study design?	If randomised controlled trial	RCT
		If non-randomised study	NRS
**SG1_earned**	For subgroup analysis: is the change to earned or unearned income?	If **likely** includes earned income as main component	Earned income
		If **only** includes unearned income e.g. welfare	Unearned income
**SG2_size**	For subgroup analysis: what size is the income change? For one-off payment, consider annual context	If <20% increase to annual income	Small change
		If >20% increase to annual income OR described in study as large OR described as 'subsistence amount' in low-income setting	Large change
		If includes small and large changes	Includes both
**SG3_SEP**	For subgroup analysis: what is the socioeconomic position of the sample? (not just country/setting)	If only includes those with low baseline SEP	Low SEP
		If only includes those on high baseline SEP	High SEP
		If includes a mix of low and high baseline SEP	Mixed

**poverty**	Does the income change likely cross a meaningful poverty or subsistence threshold?	If income change is explicitly described as crossing a poverty threshold OR represents a “large change” (as per our definition) in a population of solely low SEP individuals	Yes
		All other situations	No
**conditionality?**	Was the income change associated with conditionality? i.e. did the person have to do anything in return	If clear conditions attached to receipt e.g. conditional cash transfer	Yes
		If no clear conditions associated with income change	No
**int_meas_cat1**	What type of measurement is the intervention?		Continuous
			Binary
			Ordinal
**int_meas_cat2**	Is the income change only in one direction?		Increase only
			Decrease only
		If study does not separate the two	No distinction
**out_meas_cat**	What type of measurement is the outcome?		Continuous
			Binary
			Ordinal
**outcome_measure**	What type of datapoint is this?		Reg coefficient - unadjusted
			Reg coefficient - adjusted
			Odds ratio - unadjusted
			Odds ratio - adjusted
			Risk ratio - unadjusted
			Risk ratio - adjusted
			Rate ratio - unadjusted
			Rate ratio - adjusted
			Change/diff. in score - unadjusted
			Change/diff. in score - adjusted
			Correlation coefficient

**standardised_beta?**	If this is a regression coefficient, is it a standardised beta? i.e. the interpretation is the change in outcome in SDs resulting from 1 SD change in exposure		Yes
			No
		If unsure or unclear from study, note this	n/a
**point_est**	The main datapoint estimate of interest	Should be adjusted estimate if available	
**n_numerator**	Relevant numerator (if appropriate)	Can use this to record n of exposed/unexposed	
**n_denominator**	Relevant denominator	Should be *observations* for longitudinal data	
**other_denom**	Any other relevant denominator (if appropriate)	Can use this to record n of individuals for longitudinal data	
**lower_ci**	Lower 95% confidence interval (if appropriate)		
**upper_ci**	Upper 95% confidence interval (if appropriate)		
**se**	Standard error (if appropriate)		
**sd**	Standard deviation (if appropriate)		
**stat_sig?**	Is the point estimate of interest significant at 5% level?		Yes
			No
**comp_point_est**	The main comparator datapoint of interest	May be either outcome in comparator group OR unadjusted regression coefficient	
**comp_numerator**	Relevant numerator (if appropriate)		
**comp_denominator**	Relevant denominator		
**comp_otherdenom**	Any other relevant denominator (if appropriate)		
**comp_lower_ci**	Lower 95% confidence interval (if appropriate)		
**comp_upper_ci**	Upper 95% confidence interval (if appropriate)		
**comp_sd_or_se**	Standard deviation OR standard error (whichever appropriate)	Should be same option as main datapoint, but if not this should be indicated in notes cell	
**comp2_pointest**	Secondary comparator datapoint of interest	If used, should be described in notes cell	
**comp2_numerator**	Relevant numerator (if appropriate)		
**comp2_denominator**	Relevant denominator		
**comp2_lower_ci**	Lower 95% confidence interval (if appropriate)		
**comp2_upper_ci**	Upper 95% confidence interval (if appropriate)		
**comp2_sd_or_se**	Standard deviation OR standard error (whichever appropriate)	Should be same option as main datapoint, but if not this should be indicated in notes cell	

Appendix B: Details of search strategy

Date of searches: 5th February 2020

Total number of hits across all databases: 23,822

Number of hits once duplicates removed in EndNote: 17,823

Database	Initial	Deduplicated
Medline	1431	1413
Embase	2800	1666
Web of Science	6843	5382
PsycINFO	3650	2101
EconLit	6367	5451
ASSIA	1218	383
RePEc	1513	1427
**Total**	23,822	17,823

Number of hits once additional duplicates removed in Covidence: 16,521

Ovid MEDLINE(R) 1946 to January Week 4 2020

1	"Mental Health"/	36294
2	Depression/	114576
3	Anxiety/ or "Anxiety Disorder"/	106108
4	Well-being.ab,ti.	58627
5	Wellbeing.ab,ti.	10960
6	"Quality of Life"/	187275
7	"Life satisfaction".ab,ti.	5908
8	"Psychological distress".ab,ti.	15125
9	((income* or "social security" or earning* or salar* or wage* or money or financ* or loan* or debt* or payment* or lottery or poverty or monies or wealth or cash or welfare) adj3 (effect* or change* or increas* or decreas* or variation* or alter* or transfer* or shock* or win* or won or award* or transition or additional)).ab,ti.	19121
10	1 or 2 or 3 or 4 or 5 or 6 or 7 or 8	449982
11	9 and 10	1431

Embase 1947-Present, updated daily

1	"Mental Health"/	137116
2	Depression/	363527
3	Anxiety/ or "Anxiety Disorder"/	271435
4	Well-being.ab,ti.	91982
5	Wellbeing.ab,ti.	23716

6	"Quality of Life"/	453495
7	"Life satisfaction".ab,ti.	8751
8	"Psychological distress".ab,ti.	23551
9	((income* or social security or earning* or salar* or wage* or money or financ* or loan* or debt* or payment* or lottery or poverty or monies or wealth or cash or welfare) adj3 (effect* or change* or increas* or decreas* or variation* or alter* or transfer* or shock* or win* or won or award* or transition or additional)).ab,ti.	27743
10	1 or 2 or 3 or 4 or 5 or 6 or 7 or 8	1122531
11	9 and 10	2800

Web of Science Core Collection

# 10	6,843	#9 AND #8<br>*Databases= WOS Timespan=All years*<br>*Search language=Auto*
# 9	1,426,253	#7 OR #6 OR #5 OR #4 OR #3 OR #2 OR #1<br>*Databases= WOS Timespan=All years*<br>*Search language=Auto*
# 8	113,223	TS=((income* OR "social security" OR earning* OR salar* OR wage* OR money OR financ* OR loan* OR debt* OR payment* OR lottery OR poverty OR monies OR wealth OR cash OR welfare) NEAR/3 (effect* OR change* OR increas* OR decreas* OR variation* OR alter* OR transfer* OR shock* OR win* OR won OR award* OR transition OR additional))<br>*Databases= WOS Timespan=All years*<br>*Search language=Auto*
# 7	31,903	TS="Psychological distress"<br>*Databases= WOS Timespan=All years*<br>*Search language=Auto*
# 6	17,537	TS="Life satisfaction"<br>*Databases= WOS Timespan=All years*<br>*Search language=Auto*
# 5	437,327	TS="Quality of Life"<br>*Databases= WOS Timespan=All years*<br>*Search language=Auto*
# 4	142,010	TS=(Wellbeing OR well-being)<br>*Databases= WOS Timespan=All years*<br>*Search language=Auto*
# 3	326,424	TS=(Anxiety OR “Anxiety disorder”)<br>*Databases= WOS Timespan=All years*<br>*Search language=Auto*
# 2	611,768	TS=Depression<br>*Databases= WOS Timespan=All years*<br>*Search language=Auto*
# 1	239,157	TS=”Mental Health”<br>*Databases= WOS Timespan=All years*<br>*Search language=Auto*

PsycINFO

Search Terms	Search Options	
S9	S7 AND S8	3,650
S8	S1 OR S2 OR S3 OR S4 OR S5 OR S6	701,660
S7	TI (((income* OR "social security" OR earning* OR salar* OR wage* OR money OR financ* OR loan* OR debt* OR payment* OR lottery OR poverty OR monies OR wealth OR cash OR welfare) N3 (effect* OR change* OR increas* OR decreas* OR variation* OR alter* OR transfer* OR shock* OR win* OR won OR award* OR transition OR additional))) OR AB (((income* OR "social security" OR earning* OR salar* OR wage* OR money OR financ* OR loan* OR debt* OR payment* OR lottery OR poverty OR monies OR wealth OR cash OR welfare) N3 (effect* OR change* OR increas* OR decreas* OR variation* OR alter* OR transfer* OR shock* OR win* OR won OR award* OR transition OR additional)))	18,125
S6	SU “life satisfaction” OR TI “life satisfaction” OR AB “life satisfaction”	15,841
S5	SU “quality of life” OR TI “quality of life” OR AB “quality of life”	79,719
S4	SU (wellbeing or well-being) OR TI (wellbeing or well-being) OR AB (wellbeing or well-being)	91,776
S3	SU (anxiety or “anxiety disorder”) OR TI (anxiety or “anxiety disorder”) OR AB (anxiety or “anxiety disorder”)	212,887
S2	SU depression OR TI depression OR AB depression	274,589
S1	SU “mental health” OR TI “mental health” OR AB “mental health”	216,366

EconLit

S9	S7 AND S8	6,367
S8	S1 OR S2 OR S3 OR S4 OR S5 OR S6	44,293
S7	TI (((income* OR "social security" OR earning* OR salar* OR wage* OR money OR financ* OR loan* OR debt* OR payment* OR lottery OR poverty OR monies OR wealth OR cash OR welfare) N3 (effect* OR change* OR increas* OR decreas* OR variation* OR alter* OR transfer* OR shock* OR win* OR won OR award* OR transition OR additional))) OR AB (((income* OR "social security" OR earning* OR salar* OR wage* OR money OR financ* OR loan* OR debt* OR payment* OR lottery OR poverty OR monies OR wealth OR cash OR welfare) N3 (effect* OR change* OR increas* OR decreas* OR variation* OR alter* OR transfer* OR shock* OR win* OR won OR award* OR transition OR additional)))	86,310
S6	SU “life satisfaction” OR TI “life satisfaction” OR AB “life satisfaction”	1,408
S5	SU “quality of life” OR TI “quality of life” OR AB “quality of life”	3,362
S4	SU (wellbeing or well-being) OR TI (wellbeing or well-being) OR AB (wellbeing or well-being)	36,430
S3	SU (anxiety or “anxiety disorder”) OR TI (anxiety or “anxiety disorder”) OR AB (anxiety or “anxiety disorder”)	876
S2	SU depression OR TI depression OR AB depression	4,486
S1	SU “mental health” OR TI “mental health” OR AB “mental health”	1,780

Applied Social Sciences Index & Abstracts (ASSIA)

**S9**	S7 AND S8	1,218
**S8**	S1 OR S2 OR S3 OR S4 OR S5 OR S6	179,510

**S7**	ti(((income* OR "social security" OR earning* OR salar* OR wage* OR money OR financ* OR loan* OR debt* OR payment* OR lottery OR poverty OR monies OR wealth OR cash OR welfare) NEAR/3 (effect* OR change* OR increas* OR decreas* OR variation* OR alter* OR transfer* OR shock* OR win* OR won OR award* OR transition OR additional))) OR ab(((income* OR "social security" OR earning* OR salar* OR wage* OR money OR financ* OR loan* OR debt* OR payment* OR lottery OR poverty OR monies OR wealth OR cash OR welfare) NEAR/3 (effect* OR change* OR increas* OR decreas* OR variation* OR alter* OR transfer* OR shock* OR win* OR won OR award* OR transition OR additional)))	9,650
**S6**	su("life satisfaction") OR ti("life satisfaction") OR ab("life satisfaction")	3,838
**S5**	su("quality of life") OR ti("quality of life") OR ab("quality of life")	24,337
**S4**	su(wellbeing OR well-being) OR ti(wellbeing OR well-being) OR ab(wellbeing OR well-being)	31,076
**S3**	su(anxiety OR "anxiety disorder") OR ti(anxiety OR "anxiety disorder") OR ab(anxiety OR "anxiety disorder")	41,243
**S2**	su(depression) OR ti(depression) OR ab(depression)	59,941
**S1**	su("mental health") OR ti("mental health") OR ab("mental health")	71,210

RePEC

("mental health"| depression| anxiety| well-being| wellbeing| "quality of life"| "life satisfaction"| "psychological distress") + (income*| "social security"| earning*| salar*| wage*| money| financ*| loan*| debt*| lottery| poverty| "cash transfer"| welfare) + (change*| alter*| shock*| w?n)

Search within: abstract only. Limit to: [nil]

Articles	711 found; 5 failed URLs (2 duplicates)<br>= 706 importable, 3 manually sourced
Papers	802 found; 9 failed URLs (5 duplicates)<br>= 793 importable, 4 manually sourced

Search within: title only. Limit to: [nil]

Articles	5 found; 0 failed URLs<br>= 5 importable
Papers	2 found; 1 failed URL<br>= 1 importable, 1 manually sourced

Total: 706 + 3 + 793 + 4 + 5 + 2 = **1513**

Appendix C: PRISMA flowchart

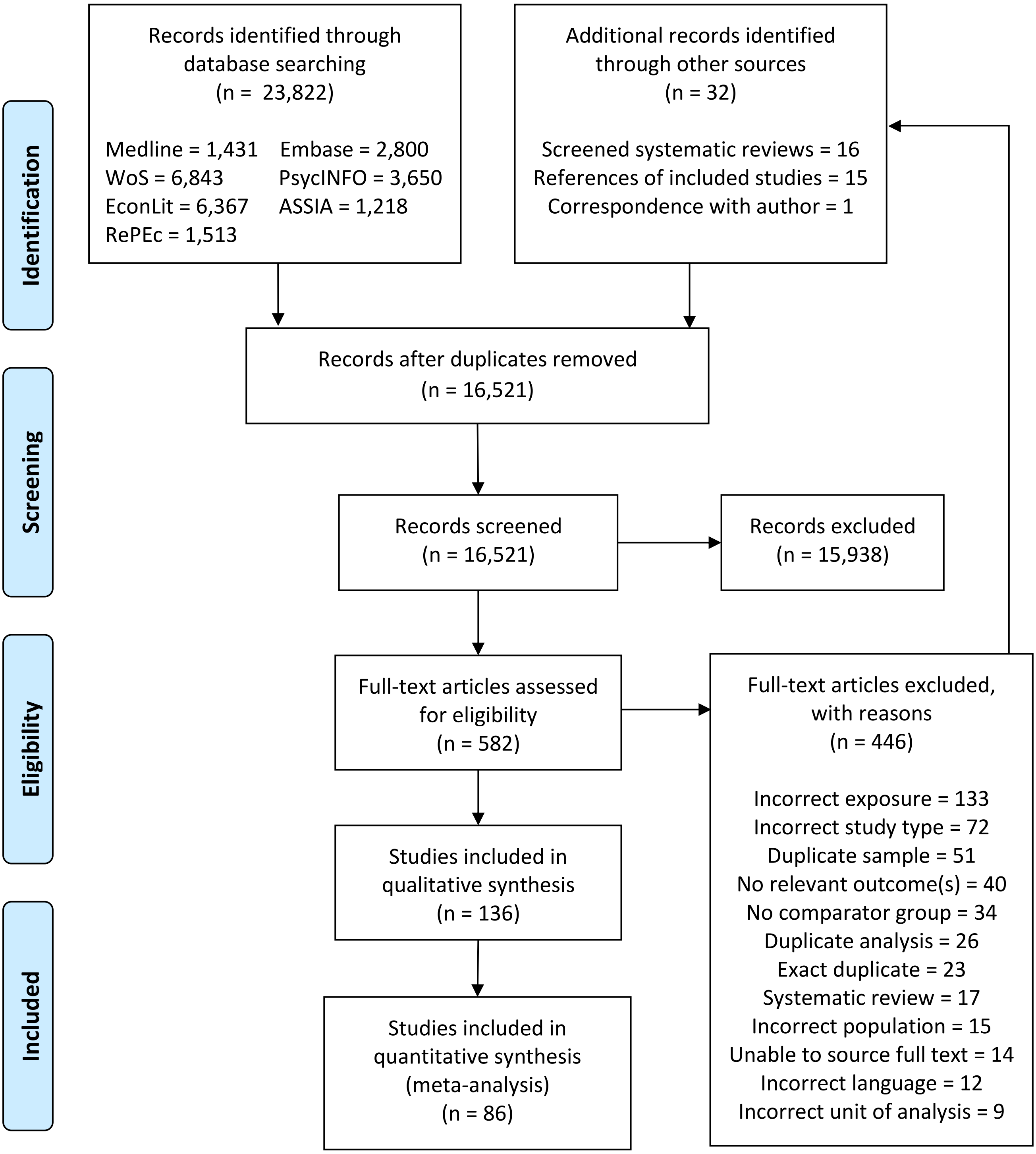

Cohen's Kappa for full text screening was 0.67, indicating substantial agreement.

Note: the 446 articles excluded at full-text stage are listed in Appendix E with reason for exclusion

Appendix D: Details of included studies

List of 136 included studies

ABBOTT, S. & HOBBY, L. 2000. Welfare benefits advice in primary care: evidence of improvements in health. *Public Health,* 114**,** 324-327.

ABBOTT, S., HOBBY, L. & COTTER, S. 2006. What is the impact on individual health of services in general practice settings which offer welfare benefits advice? *Health & Social Care in the Community,* 14**,** 1-8.

ABEL, G. A., ALBELDA, R., KHERA, N., HAHN, T., CORONADO, D. Y. S., ODEJIDE, O. O., BONA, K., TUCKER-SEELEY, R. & SOIFFER, R. 2016. Financial Hardship and Patient-Reported Outcomes after Hematopoietic Cell Transplantation. *Biology of Blood and Marrow Transplantation,* 22**,** 1504-1510.

ADAMS, H., BELL, A. R. & TAMAL, M. E. H. 2019. Temporal dimensions of reported life satisfaction in a low-income, agricultural environment. *Ecology and Society,* 24.

ADEOLA, F. O. 2009. Mental health & psychosocial distress sequelae of Katrina: An empirical study of survivors. *Human Ecology Review***,** 195-210.

ALLOUCHE, M. 2019. *Psychological Well-Being and the Dynamics of Poverty.* Ph.D., University of California, Davis.

AMBREY, C. L. & FLEMING, C. M. 2014. The causal effect of income on life satisfaction and the implications for valuing non-market goods. *Economics Letters,* 123**,** 131-134.

AMERICAN INSTITUTES FOR RESEARCH 2014. 12-Month Impact Report for Zimbabwe's Harmonised Social Cash Transfer Programmes. American Institutes for Research Washington, DC.

APOUEY, B. & CLARK, A. E. 2015. Winning big but feeling no better? The effect of lottery prizes on physical and mental health. *Health Economics,* 24**,** 516-38.

ARATA, C. M., PICOU, J. S., JOHNSON, G. D. & MCNALLY, T. S. 2000. Coping with technological disaster: An application of the conservation of resources model to the Exxon Valdez oil spill. *Journal of Traumatic Stress: Official Publication of The International Society for Traumatic Stress Studies,* 13**,** 23-39.

ARAYA, R., LEWIS, G., ROJAS, G. & FRITSCH, R. 2003. Education and income: Which is more important for mental health? *Journal of epidemiology and community health,* 57**,** 501-505.

ASADULLAH, M. N. & CHAUDHURY, N. 2012. Subjective well-being and relative poverty in rural Bangladesh. *Journal of Economic Psychology,* 33**,** 940-950.

ASSARI, S., PREISER, B. & KELLY, M. 2018. Education and income predict future emotional well-being of whites but not blacks: A ten-year cohort. *Brain Sciences,* 8.

BAIRD, S., DE HOOP, J. & OZLER, B. 2013. Income Shocks and Adolescent Mental Health. *Journal of Human Resources,* 48**,** 370-403.

BARBAGLIA, M. G., TEN HAVE, M., DORSSELAER, S., ALONSO, J. & DE GRAAF, R. 2015. Negative socioeconomic changes and mental disorders: a longitudinal study. *Journal of Epidemiology & Community Health,* 69**,** 55-62.

BECCHETTI, L. & CASTRIOTA, S. 2010. The Effects of a Calamity on Income and Wellbeing of Poor Microfinance Borrowers: The Case of the 2004 Tsunami Shock. *Journal of Development Studies,* 46**,** 211-233.

BEDOYA ARGUELLES, G., COVILLE, A., HAUSHOFER, J., ISAQZADEH, M. R. & SHAPIRO, J. 2019. No Household Left Behind : Afghanistan Targeting the Ultra Poor Impact Evaluation. The World Bank, Policy Research Working Paper Series: 8877.

BENZEVAL, M. & JUDGE, K. 2001. Income and health: the time dimension. *Social science & medicine,* 52**,** 1371-1390.

BIOTTEAU, A. L., BONNET, C. & CAMBOIS, E. 2019. Risk of Major Depressive Episodes After Separation: The Gender-Specific Contribution of the Income and Support Lost Through Union Dissolution. *European Journal of Population-Revue Europeenne De Demographie,* 35**,** 519-542.

BLAND, S. H., O'LEARY, E. S., FARINARO, E., JOSSA, F. & TREVISAN, M. 1996. Long-term psychological effects of natural disasters. *Psychosomatic medicine,* 58**,** 18-24.

BLAZQUEZ CUESTA, M. & BUDRIA, S. 2013. Does income deprivation affect people's mental well-being? : Banco de Espana, Banco de Espana Working Papers: 1312.

BOLDEN, L. & WICKS, M. N. 2010. Predictors of Mental Health, Subjective Burden, and Rewards in Family Caregivers of Patients With Chronic Liver Disease. *Archives of Psychiatric Nursing,* 24**,** 89-103.

BONANNO, G. A., GALEA, S., BUCCIARELLI, A. & VLAHOV, D. 2007. What Predicts Psychological Resilience After Disaster? The Role of Demographics, Resources, and Life Stress. *Journal of Consulting and Clinical Psychology,* 75**,** 671-682.

BOYCE, C. J., DELANEY, L. & WOOD, A. M. 2018. The Great Recession and subjective well-being: How did the life satisfaction of people living in the United Kingdom change following the financial crisis? *PLoS ONE [Electronic Resource],* 13**,** e0201215.

BOYCE, C. J., WOOD, A. M., BANKS, J., CLARK, A. E. & BROWN, G. D. 2013. Money, well-being, and loss aversion: does an income loss have a greater effect on well-being than an equivalent income gain? *Psychological Science,* 24**,** 2557-62.

BOYCE, W., RAJA, S., PATRANABISH, R. G., BEKOE, T., DEME-DER, D. & GALLUPE, O. 2009. Occupation, poverty and mental health improvement in Ghana. *Alter,* 3**,** 233-244.

BOYD-SWAN, C., HERBST, C. M., IFCHER, J. & ZARGHAMEE, H. 2016. The earned income tax credit, mental health, and happiness. *Journal of Economic Behavior & Organization,* 126**,** 18-38.

BRENNER, M. H., ANDREEVA, E., THEORELL, T., GOLDBERG, M., WESTERLUND, H., LEINEWEBER, C., HANSON, L. L. M., IMBERNON, E. & BONNAUD, S. 2014. Organizational downsizing and depressive symptoms in the European recession: the experience of workers in France, Hungary, Sweden and the United Kingdom. *PloS one,* 9**,** e97063.

BRICKMAN, P., COATES, D. & JANOFF-BULMAN, R. 1978. Lottery winners and accident victims: Is happiness relative? *Journal of personality and social psychology,* 36**,** 917.

BROCK, A. M. & O'SULLIVAN, P. 1985. From wife to widow: Role transition in the elderly. *Journal of psychosocial nursing and mental health services,* 23**,** 6-12.

BURKE, R. J. 1986. Reemployment on a poorer job after a plant closing. *Psychological Reports,* 58**,** 559-570.

BURMASTER, K. B., LANDEFELD, J. C., REHKOPF, D. H., LAHIFF, M., SOKAL-GUTIERREZ, K., ADLER-MILSTEIN, S. & FERNALD, L. C. 2015. Impact of a private sector living wage intervention on depressive symptoms among apparel workers in the Dominican Republic: a quasi-experimental study. *BMJ Open,* 5**,** e007336.

BUTTKE, D., VAGI, S., SCHNALL, A., BAYLEYEGN, T., MORRISON, M., ALLEN, M. & WOLKIN, A. 2012. Community Assessment for Public Health Emergency Response (CASPER) one year following the Gulf Coast oil spill: Alabama and Mississippi, 2011. *Prehospital & Disaster Medicine,* 27**,** 496-502.

CAI, S. & PARK, A. 2016. Permanent income and subjective well-being. *Journal of Economic Behavior & Organization,* 130**,** 298-319.

CASEY, P., GOOLSBY, S., BERKOWITZ, C., FRANK, D., COOK, J., CUTTS, D., BLACK, M. M., ZALDIVAR, N., LEVENSON, S., HEEREN, T. & MEYERS, A. 2004. Maternal Depression, Changing Public Assistance, Food Security, and Child Health Status. *Pediatrics,* 113**,** 298-304.

CESARINI, D., LINDQVIST, E., OSTLING, R. & WALLACE, B. 2016. WEALTH, HEALTH, AND CHILD DEVELOPMENT: EVIDENCE FROM ADMINISTRATIVE DATA ON SWEDISH LOTTERY PLAYERS. *Quarterly Journal of Economics,* 131**,** 687-738.

CHEUNG, F. & LUCAS, R. E. 2015. When does money matter most? Examining the association between income and life satisfaction over the life course. *Psychology and aging,* 30**,** 120.

CHIEN, I. C., CHOU, F. H.-C., CHOU, P., HUANG, M.-W., LU, M.-K., OU-YANG, W.-C. & SU, T. T.-P. 2004. Quality of life and related risk factors in a Taiwanese Village population 21 months after an earthquake. *Australian and New Zealand Journal of Psychiatry,* 38**,** 358-364.

CHIN, W. S., GUO, Y. L., LIAO, S. C., WU, H. C., KUO, C. Y., CHEN, C. C. & SHIAO, J. S. 2018. Quality of life at 6 years after occupational injury. *Quality of Life Research,* 27**,** 609-618.

CHROSTEK, P. 2016. An Empirical Investigation into the Determinants and Persistence of Happiness and Life Evaluation. *Journal of Happiness Studies,* 17**,** 413-430.

CLARK, A. E., D'AMBROSIO, C. & GHISLANDI, S. 2016. Adaptation to poverty in long-run panel data. *Review of Economics and Statistics,* 98**,** 591-600.

CLINGINGSMITH, D. 2016. Negative emotions, income, and welfare: Causal estimates from the PSID. *Journal of Economic Behavior & Organization,* 130**,** 1-19.

COLEY, R. L. & LOMBARDI, C. M. 2014. Low-income women's employment experiences and their financial, personal, and family well-being. *Journal of Family Psychology,* 28**,** 88-97.

COSTELLO, E. J., ERKANLI, A., COPELAND, W. & ANGOLD, A. 2010. Association of Family Income Supplements in Adolescence With Development of Psychiatric and Substance Use Disorders in Adulthood Among an American Indian Population. *Jama-Journal of the American Medical Association,* 303**,** 1954-1960.

COURTIN, E., MUENNIG, P., VERMA, N., RICCIO, J. A., LAGARDE, M., VINEIS, P., KAWACHI, I. & AVENDANO, M. 2018. Conditional Cash Transfers And Health Of Low-Income Families In The US: Evaluating The Family Rewards Experiment. *Health Affairs,* 37**,** 438-446.

DALEY, A. 2017. Income and the mental health of Canadian mothers: Evidence from the Universal Child Care Benefit. *Ssm-Population Health,* 3**,** 674-683.

DANG, H.-A. H., LOKSHIN, M. M. & ABANOKOVA, K. 2019. Did the Poor Adapt to Their Circumstances? Evidence from Long-run Russian Panel Data. *Economics Bulletin,* 39**,** 2258-2274.

DEARING, E., TAYLOR, B. A. & MCCARTNEY, K. 2004. Implications of family income dynamics for women's depressive symptoms during the first 3 years after childbirth. *American Journal of Public Health,* 94**,** 1372-7.

DIENER, E., SANDVIK, E., SEIDLITZ, L. & DIENER, M. 1993. THE RELATIONSHIP BETWEEN INCOME AND SUBJECTIVE WELL-BEING - RELATIVE OR ABSOLUTE. *Social Indicators Research,* 28**,** 195-223.

DOLAN, P. & LORDAN, G. 2013. Moving Up and Sliding Down: An Empirical Assessment of the Effect of Social Mobility on Subjective Wellbeing. Centre for Economic Performance, LSE, CEP Discussion Papers.

DORSETT, R. & OSWALD, A. J. 2014. HUMAN WELL-BEING AND IN-WORK BENEFITS: A RANDOMIZED CONTROLLED TRIAL. University of Warwick, Department of Economics, The Warwick Economics Research Paper Series (TWERPS).

ELWELL-SUTTON, T., FOLB, N., CLARK, A., FAIRALL, L. R., LUND, C. & BACHMANN, M. O. 2019. Socioeconomic position and depression in South African adults with long-term health conditions: a longitudinal study of causal pathways. *Epidemiology & Psychiatric Science,* 28**,** 199-209.

ERIXSON, O. 2017. Health responses to a wealth shock: evidence from a Swedish tax reform. *Journal of Population Economics,* 30**,** 1281-1336.

EVANS, S. & HUXLEY, P. 2005. Adaptation, response-shift and quality of life ratings in mentally well and unwell groups. *Quality of Life Research,* 14**,** 1719-32.

EVANS, W. N. & GARTHWAITE, C. L. 2010. Giving Mom a Break: The Impact of Higher EITC Payments on Maternal Health. *National Bureau of Economic Research Working Paper Series,* No. 16296.

FANG, Z. & NIIMI, Y. 2015. Do Losses Bite More than Gains? Evidence from a Panel Quantile Regression Analysis of Subjective Well-being in Japan. Nanyang Technological University, School of Humanities and Social Sciences, Economic Growth Centre, Economic Growth Centre Working Paper Series: 1507.

FEENY, S., MCDONALD, L. & POSSO, A. 2014. Are Poor People Less Happy? Findings from Melanesia. *World Development,* 64**,** 448-459.

FENN, B., NOURA, G., SIBSON, V., DOLAN, C. & SHOHAM, J. 2015. The role of unconditional cash transfers during a nutritional emergency in Maradi region, Niger: a pre-post intervention observational study. *Public Health Nutrition,* 18**,** 343-51.

FERNALD, L. C. & HIDROBO, M. 2011. Effect of Ecuador's cash transfer program (Bono de Desarrollo Humano) on child development in infants and toddlers: a randomized effectiveness trial. *Social science & medicine,* 72**,** 1437-1446.

FRIJTERS, P., JOHNSTON, D. W. & SHIELDS, M. A. 2011. Life Satisfaction Dynamics with Quarterly Life Event Data. *Scandinavian Journal of Economics,* 113**,** 190-211.

FRIJTERS, P., JOHNSTON, D. W. & SHIELDS, M. A. 2012. The Optimality of Tax Transfers: What does Life Satisfaction Data Tell Us? *Journal of Happiness Studies,* 13**,** 821-832.

GALAMA, T. J., MORGAN, R. & SAAVEDRA, J. E. 2017. Wealthier, Happier and More Self-Sufficient: When Anti-Poverty Programs Improve Economic and Subjective Wellbeing at a Reduced Cost to Taxpayers. National Bureau of Economic Research, Inc, NBER Working Papers: 24090.

GASSMAN-PINES, A. & YOSHIKAWA, H. 2006. Five-year effects of an anti-poverty program on marriage among never-married mothers. *Journal of Policy Analysis and Management,* 25**,** 11-30.

GENNETIAN, L. A. & MILLER, C. 2002. Children and welfare reform: A view from an experimental welfare program in Minnesota. *Child development,* 73**,** 601-620.

GRATTAN, L. M., BRUMBACK, B., ROBERTS, S. M., BUCKINGHAM-HOWES, S., TOBEN, A. C. & MORRIS, G. 2017. "Bouncing back" after the Deepwater Horizon oil spill. *Disaster Prevention and Management,* 26**,** 122-133.

GREEN, E. P., BLATTMAN, C., JAMISON, J. & ANNAN, J. 2016. Does poverty alleviation decrease depression symptoms in post-conflict settings? A cluster-randomized trial of microenterprise assistance in Northern Uganda. *Global Mental Health,* 3**,** 9.

GROS, C., BAILEY, M., SCHWAGER, S., HASSAN, A., ZINGG, R., UDDIN, M. M., SHAHJAHAN, M., ISLAM, H., LUX, S., JAIME, C. & DE PEREZ, E. C. 2019. Household-level effects of providing forecast-based cash in anticipation of extreme weather events: Quasi-experimental evidence from humanitarian interventions in the 2017 floods in Bangladesh. *International Journal of Disaster Risk Reduction,* 41.

GULAL, F. & AYAITA, A. 2019. The Impact of Minimum Wages on Well-Being: Evidence from a Quasi-experiment in Germany. *Journal of Happiness Studies*, 24.

HANDA, S., PARK, M., DARKO, R. O., OSEI-AKOTO, I., DAVIS, B. & DAIDONE, S. 2014. Livelihood empowerment against poverty program impact evaluation. *Carolina Population Center, Chapel Hill, NC: University of North Carolina*.

HANLY, P., MAGUIRE, R., HYLAND, P. & SHARP, L. 2015. Examining the role of subjective and objective burden in carer health-related quality of life: the case of colorectal cancer. *Supportive Care in Cancer,* 23, 1941-9.

HASAN, H. 2016. Does Happiness Adapt to Increase in Income? Evidence from Pakistan Socio-economic Survey (1998-2001). *Pakistan Development Review,* 55, 113-122.

HAUSHOFER, J. & SHAPIRO, J. 2016. The Short-term Impact of Unconditional Cash Transfers to the Poor: Experimental Evidence from Kenya. *Quarterly Journal of Economics,* 131, 1973-2042.

HILTON, J. M. & KOPERA-FRYE, K. 2006. Loss and Depression in Cohabiting and Noncohabiting Custodial Single Parents. *The Family Journal,* 14, 28-40.

HJELM, L., HANDA, S., DE HOOP, J. & PALERMO, T. 2017. Poverty and perceived stress: Evidence from two unconditional cash transfer programs in Zambia. *Social Science & Medicine,* 177, 110-117.

HORN, B. P., MACLEAN, J. C. & STRAIN, M. R. 2017. DO MINIMUM WAGE INCREASES INFLUENCE WORKER HEALTH? *Economic Inquiry,* 55, 1986-2007.

HUANG, Y., WONG, H. & TAN, N. T. 2015. Associations between economic loss, financial strain and the psychological status of Wenchuan earthquake survivors. *Disasters,* 39, 795-810.

ISHIGURO, A., INOUE, M., FISHER, J., INOUE, M., MATSUMOTO, S. & YAMAOKA, K. 2019. Gender-Based Risk and Protective Factors for Psychological Distress in the Midterm Recovery Period Following the Great East Japan Earthquake. *Disaster Medicine and Public Health Preparedness,* 13, 487-496.

JALAL, C. S., FRONGILLO, E. A. & WARREN, A. M. 2015. Food Insecurity Mediates the Effect of a Poverty-Alleviation Program on Psychosocial Health among the Ultra-Poor in Bangladesh. *Journal of Nutrition,* 145, 1934-41.

JUNNA, L., MOUSTGAARD, H., TARKIAINEN, L. & MARTIKAINEN, P. 2019. The Association Between Income and Psychotropic Drug Purchases: Individual Fixed Effects Analysis of Annual Longitudinal Data in 2003-2013. *Epidemiology,* 30, 221-229.

KENDALL, G. E., NGUYEN, H. & ONG, R. 2019. The association between income, wealth, economic security perception, and health: a longitudinal Australian study. *Health Sociology Review,* 28, 20-38.

KIERNAN, F. 2018. The Great Recession and Mental Health: the Effect of Income Loss on the Psychological Health of Young Mothers. Geary Institute, University College Dublin, Working Papers: 201821.

KIKUCHI, H., MIFUNE, N., NIINO, M., KIRA, J. I., KOHRIYAMA, T., OTA, K., TANAKA, M., OCHI, H., NAKANE, S. & KIKUCHI, S. 2013. Structural equation modeling of factors contributing to quality of life in Japanese patients with multiple sclerosis. *BMC Neurology,* 13 (no pagination).

KILBURN, K., HANDA, S., ANGELES, G., TSOKA, M. & MVULA, P. 2018. Paying for Happiness: Experimental Results from a Large Cash Transfer Program in Malawi. *Journal of Policy Analysis and Management,* 37, 331-+.

KILBURN, K., THIRUMURTHY, H., HALPERN, C. T., PETTIFOR, A. & HANDA, S. 2016. Effects of a Large-Scale Unconditional Cash Transfer Program on Mental Health Outcomes of Young People in Kenya. *Journal of Adolescent Health,* 58, 223-9.

KLEIN, J., LUDECKE, D., HOFREUTER-GATGENS, K., FISCH, M., GRAEFEN, M. & VON DEM KNESEBECK, O. 2017. Income and health-related quality of life among prostate cancer patients over a one-year period after radical prostatectomy: a linear mixed model analysis. *Quality of Life Research,* 26**,** 2363-2373.

KOLTAI, J., BIERMAN, A. & SCHIEMAN, S. 2018. Financial circumstances, mastery, and mental health: Taking unobserved time-stable influences into account. *Social Science & Medicine,* 202**,** 108-116.

KRAUSS, A. & GRAHAM, C. 2013. Subjective wellbeing in Colombia : some insights on vulnerability, job security, and relative incomes. The World Bank, Policy Research Working Paper Series: 6672.

KUHN, P., KOOREMAN, P., SOETEVENT, A. & KAPTEYN, A. 2011. The effects of lottery prizes on winners and their neighbors: Evidence from the Dutch postcode lottery. *American Economic Review,* 101**,** 2226-47.

LACHOWSKA, M. 2017. The Effect of Income on Subjective Well-Being Evidence from the 2008 Economic Stimulus Tax Rebates. *Journal of Human Resources,* 52**,** 374-417.

LAFAVE, H. G., DE SOUZA, H. R., PRINCE, P. N., ATCHISON, K. E. & GERBER, G. J. 1995. Partnerships for people with serious mental illness who live below the poverty line. *Psychiatric Services*.

LAM, J. A. & ROSENHECK, R. A. 2000. Correlates of improvement in quality of life among homeless persons with serious mental illness. *Psychiatric Services,* 51**,** 116-8.

LATIF, E. 2015. Happiness Adaptation to Income: Evidence from Canada. *Economics Bulletin,* 35**,** 1477-1487.

LEBIHAN, L. & MAO TAKONGMO, C.-O. 2018. The Impact of Universal Child Benefits on Family Health and Behaviours. *Research in Economics,* 72**,** 415-427.

LINDAHL, M. 2005. Estimating the effect of income on health and mortality using lottery prizes as an exogenous source of variation in income. *Journal of Human resources,* 40**,** 144-168.

LINDQVIST, E., OSTLING, R. H. & CESARINI, D. 2018. Long-run Effects of Lottery Wealth on Psychological Well-being. National Bureau of Economic Research, Inc, NBER Working Papers: 24667.

LORANT, V., CROUX, C., WEICH, S., DELIÈGE, D., MACKENBACH, J. & ANSSEAU, M. 2007. Depression and socio-economic risk factors: 7-year longitudinal population study. *The British journal of psychiatry,* 190**,** 293-298.

LORENZ, F. O., ELDER, G. H., BAO, W. N., WICKRAMA, K. A. S. & CONGER, R. D. 2000. After farming: Emotional health trajectories of farm, nonfarm, and displaced farm couples. *Rural Sociology,* 65**,** 50-71.

MACOURS, K., SCHADY, N. & VAKIS, R. 2012. Cash transfers, behavioral changes, and cognitive development in early childhood: evidence from a randomized experiment. *American Economic Journal: Applied Economics,* 4**,** 247-73.

MAEDER, M. 2014. Earnings-related parental leave benefits and subjective well-being of young mothers: evidence from a German parental leave reform. Bavarian Graduate Program in Economics (BGPE).

MARKUSSEN, T. & NGO, Q.-T. 2019. Economic and Non-economic Returns to Communist Party Membership in Vietnam. *World Development,* 122**,** 370-384.

MCCARTHY, B., CARTER, A., JANSSON, M., BENOIT, C. & FINNIGAN, R. 2018. Poverty, Material Hardship, and Mental Health among Workers in Three Front-Line Service Occupations. *Journal of Poverty,* 22**,** 334-354.

MCKENZIE, S. K., GUNASEKARA, F. I., RICHARDSON, K. & CARTER, K. 2014. Do changes in socioeconomic factors lead to changes in mental health? Findings from three waves of a population based panel study. *Journal of epidemiology and community health,* 68**,** 253-260.

MELZER, S. M. & MUFFELS, R. J. 2017. Migrants' pursuit of happiness: An analysis of the effects of adaptation, social comparison and economic integration on subjective well-being on the basis of German panel data for 1990-2014. *Migration Studies,* 5**,** 190-215.

MILLIGAN, K. & STABILE, M. 2008. Do Child Tax Benefits Affect the Wellbeing of Children? Evidence from Canadian Child Benefit Expansions. National Bureau of Economic Research, Inc, NBER Working Papers: 14624.

MOHANTY, M. S. 2014. What Determines Happiness? Income or Attitude: Evidence from the U.S. Longitudinal Data. *Journal of Neuroscience, Psychology, and Economics,* 7**,** 80-102.

NATALI, L., HANDA, S., PETERMAN, A., SEIDENFELD, D. & TEMBO, G. 2018. Does money buy happiness? Evidence from an unconditional cash transfer in Zambia. *SSM - Population Health,* 4**,** 225-235.

NORTH, R. J., HOLAHAN, C. J., MOOS, R. H. & CRONKITE, R. C. 2008. Family support, family income, and happiness: A 10-year perspective. *Journal of Family Psychology,* 22**,** 475-483.

ONG, R., NGUYEN, T. & KENDALL, G. 2018. The impact of intergenerational financial transfers on health and wellbeing outcomes: A longitudinal study. *Social Science & Medicine,* 214**,** 179-186.

OSHIO, T., UMEDA, M. & FUJII, M. 2012. The association between income dynamics and subjective well-being: Evidence from career income records in Japan. Center for Intergenerational Studies, Institute of Economic Research, Hitotsubashi University.

OZER, E. J., FERNALD, L. C. H., WEBER, A., FLYNN, E. P. & VANDERWEELE, T. J. 2011. Does alleviating poverty affect mothers' depressive symptoms? A quasi-experimental investigation of Mexico's Oportunidades programme. *International Journal of Epidemiology,* 40**,** 1565-1576.

PLAGERSON, S., PATEL, V., HARPHAM, T., KIELMANN, K. & MATHEE, A. 2011. Does money matter for mental health? Evidence from the Child Support Grants in Johannesburg, South Africa. *Global Public Health,* 6**,** 760-76.

POWELL-JACKSON, T., PEREIRA, S. K., DUTT, V., TOUGHER, S., HALDAR, K. & KUMAR, P. 2016. Cash transfers, maternal depression and emotional well-being: Quasi-experimental evidence from India's Janani Suraksha Yojana programme. *Social Science & Medicine,* 162**,** 210-8.

PRIEBE, S., YEELES, K., BREMNER, S., LAUBER, C., ELDRIDGE, S., ASHBY, D., DAVID, A. S., O'CONNELL, N., FORREST, A. & BURNS, T. 2013. Effectiveness of financial incentives to improve adherence to maintenance treatment with antipsychotics: Cluster randomised controlled trial. *BMJ (Online),* 347.

PROTO, E. & RUSTICHINI, A. 2015. Life satisfaction, income and personality. *Journal of Economic Psychology,* 48**,** 17-32.

RADEY, M., MCWEY, L. & CUI, M. 2019. Psychological distress among low-income mothers: the role of public and private safety nets. *Women & Health***,** 15.

RASCHKE, C. 2019. Unexpected windfalls, education, and mental health: evidence from lottery winners in Germany. *Applied Economics,* 51**,** 207-218.

REEVE, J., JAMES, F., MCNEILL, R., BROWN, P., CAMERON, L. & MILLS, S. 2011. Functional and psychological outcomes following burn injury: reduced income and hidden emotions are predictors of greater distress. *Journal of Burn Care & Research,* 32**,** 468-74.

REEVES, A., CLAIR, A., MCKEE, M. & STUCKLER, D. 2016. Reductions in the United Kingdom's government housing benefit and symptoms of depression in low-income households. *American journal of epidemiology,* 184**,** 421-429.

REEVES, A., MCKEE, M., MACKENBACH, J., WHITEHEAD, M. & STUCKLER, D. 2017. Introduction of a National Minimum Wage Reduced Depressive Symptoms in Low-Wage Workers: A Quasi-Natural Experiment in the UK. *Health Economics,* 26**,** 639-655.

ROBERT, G., MARTINEZ, J. M., GARCIA, A. M., BENAVIDES, F. G. & RONDA, E. 2014. From the boom to the crisis: changes in employment conditions of immigrants in Spain and their effects on mental health. *European Journal of Public Health,* 24**,** 404-9.

ROGERS, S. J. & DEBOER, D. D. 2001. Changes in wives' income: Effects on marital happiness, psychological well-being, and the risk of divorce. *Journal of Marriage and Family,* 63**,** 458-472.

ROH, Y. H., CHANG, J. Y., KIM, M. U. & NAM, S. K. 2014. The Effects of Income and Skill Utilization on the Underemployed's Self-Esteem, Mental Health, and Life Satisfaction. *Journal of Employment Counseling,* 51**,** 125-141.

ROSENHECK, R. A., DAUSEY, D. J., FRISMAN, L. & KASPROW, W. 2000. Outcomes after initial receipt of social security benefits among homeless veterans with mental illness. *Psychiatric Services,* 51**,** 1549-54.

SAREEN, J., AFIFI, T. O., MCMILLAN, K. A. & ASMUNDSON, G. J. G. 2011. Relationship between household income and mental disorders: Findings from a population-based longitudinal study. *Archives of General Psychiatry,* 68**,** 419-427.

SCHOLLGEN, I., KERSTEN, N. & ROSE, U. 2019. Income trajectories and subjective well-being: Linking administrative records and survey data. *International Journal of Environmental Research and Public Health,* 16.

SCHYNS, P. 2001. Income and satisfaction in Russia. *Journal of Happiness Studies: An Interdisciplinary Forum on Subjective Well-Being,* 2**,** 173-204.

SEKULOVA, F. & VAN DEN BERGH, J. 2013. Climate change, income and happiness: An empirical study for Barcelona. *Global Environmental Change-Human and Policy Dimensions,* 23**,** 1467-1475.

SWIFT, S. L., ELFASSY, T., BAILEY, Z., FLOREZ, H., FEASTER, D. J., CALONICO, S., SIDNEY, S., KIEFE, C. I. & AL HAZZOURI, A. Z. 2020. Association of negative financial shocks during the Great Recession with depressive symptoms and substance use in the USA: the CARDIA study. *J Epidemiol Community Health,* 74**,** 995-1001.

TACHIBANA, T., GOTO, R., SAKURAI, T., RAYAMAJHI, S., ADHIKARI, A. & DOW, W. H. 2019. Do remittances alleviate negative impacts of disaster on mental health? A case of the 2015 Nepal earthquake. *Social Science & Medicine,* 238.

THOITS, P. & HANNAN, M. 1979. Income and psychological distress: The impact of an income-maintenance experiment. *Journal of Health and Social Behavior,* 20**,** 120-138.

THOMPSON, S., HARTEL, G., MANDERSON, L., WOELZ-STIRLING, N. & KELAHER, M. 2002. The mental health status of Filipinas in Queensland. *Australian & New Zealand Journal of Psychiatry,* 36**,** 674-80.

TURVEY, C., STROMQUIST, A., KELLY, K., ZWERLING, C. & MERCHANT, J. 2002. Financial loss and suicidal ideation in a rural community sample. *Acta Psychiatrica Scandinavica,* 106**,** 373-380.

WICKHAM, S., WHITEHEAD, M., TAYLOR-ROBINSON, D. & BARR, B. 2017. The effect of a transition into poverty on child and maternal mental health: a longitudinal analysis of the UK Millennium Cohort Study. *The lancet. Public Health,* 2**,** e141-e148.

WONG, H. & YE, S. 2015. Impact of enforcing a statutory minimum wage on work and quality of life of vulnerable groups in Hong Kong. *International Journal of Social Welfare,* 24**,** 223-235.

YOUNG, H. Y., YOUNG, S. R., IM, O. K., JUNG, S. L., SOO, M. B., WON, S. L., JUN, S. K., SI, Y. K., SANG, W. S. & YOUNG, S. H. 2005. Economic burdens and quality of life of family caregivers of cancer patients. *Oncology,* 68**,** 107-114.

ZIMMERMAN, F. J. & KATON, W. 2005. Socioeconomic status, depression disparities, and financial strain: what lies behind the income-depression relationship? *Health Economics,* 14**,** 1197-215.

Table D1: Included studies and their characteristics. Pov? = studies which consider impact of poverty transitions. Co-Is? = studies which include important co-interventions (for the exposed group only). Time = number of months between start of intervention and outcome measurement (where study included >1 timepoint, time reported is that used in primary analysis). DPs = datapoints. ED = effect direction (beneficial [Ben.], inconsistent [Inc.], or harmful [Har.]). MA? = studies which were included in meta-analysis. Country codes = ISO Alpha-3.

Study	Title	Cou-ntry	Years	Study design	Sampling frame & sample size	Intervention	Pov?	Co-Is?	Time	Outcome	No. DPs	ED	Findings	Risk of bias	MA?
Abbott 2000	Welfare benefits advice in primary care: evidence of improvements in health	GBR	1996-1997	Non-randomised trial of intervention	Welfare benefits advice service users in seven Liverpool GP practices (n=68)	If incomes **increased** 6 months after initial attendance at the Health and Advice Project, a GP practice-based welfare benefits advice service managed by the Citizens Advice Bureau	No	No	12	Short form 36 (SF-36) mental health subscale	2	Ben.	There were significant difference in pre/post intervention scores between those whose incomes increased and those whose incomes didn't following benefits advice service	Critical	Yes
Abbott 2006	What is the impact on individual health of services in general practice settings which offer welfare benefits advice?	GBR	2000-2001	Non-randomised trial of intervention	Welfare benefits advice service users in seven sites: 59 GP practices across England (n=244)	If participants had successfully claimed and appealed, resulting in an income **increase**, 6 months after attendance at a welfare advice service based in their GP practice	No	No	12	SF-36 mental health subscale	2	Inc.	Those whose incomes increased within 6 months of using benefits advice service did not see significant improvement in SF-36 score by 6 months, but did by 12 months	Critical	Yes
Abel 2016	Financial Hardship and Patient-Reported Outcomes after Hematopoietic Cell Transplantation	USA	2014-2015	Basic cross-sectional	All adult patients (18+) surviving 150 days after hematopoietic cell transplant (HCT) at three cancer centres in Boston, Arizona and Buffalo NY (n=325)	Experiencing a **decrease** in monthly household income compared with the period before undergoing HCT	No	No	6	1. Perceived Stress Scale-4 (PSS) 2. Quality of life (QoL) score on a 7-point self assessment scale using 'similar wording as global health status domain of EORTC QLQ-C30'	2	Ben.	After stem cell transplant for haematological cancers, those who experienced income reduction after surviving ~173 days had increased odds of stress and better QoL, though the latter was not significant	Critical	Yes
Adams 2019	Temporal dimensions of reported life satisfaction in a low-income, agricultural environment	BGD	2015-2016	Basic longitudinal	Farmers in two upazilas (subdistricts) in the Rangpur division of northwestern Bangladesh (n=353)	Presence of household income shocks such as death or illness of a main income earner **(decrease)**	No	Yes	0.5	Life satisfaction, 11 point scale (Cantril self-anchoring scale)	2	Inc.	Income shocks in the current period and two week lagged period for Bangladeshi agricultural workers were not significantly associated with life satisfaction	Serious	No

Adeola 2009	Mental Health & Psychosocial Distress Sequelae of Katrina: An Empirical Study of Survivors	USA	2006	Basic cross-sectional	Adults (18+) who survived Hurricane Katrina and requested aid from the Red Cross (n=602)	The impact of Hurricane Katrina on personal finances rated on a 5-point Likert scale **(decrease)**	No	No	0	Composite measure indicating acute psychological distress	1	Ben.	Survivors of Hurricane Katrina had higher levels of psychological distress if they experienced more of an impact of the disaster on their finances	Critical	No
AIR 2014	12-Month Impact Report for Zimbabwe's Harmonised Social Cash Transfer Programmes	ZWE	2013-2014	Non-randomised trial of intervention	Zimbabwean households that were labour constrained and food poor. Included adolescent respondents (aged 14-21 at follow-up) and household head (n=3,430)	Receiving the HSCT cash transfer, ranging from US$10-$25 depending on household size (approx. $5 per person) - represents **increase** of 20% of household consumption.	Yes	No	12	1. Center for Epidemiological Studies Depression scale, 10 item version (CES-D 10) 2. Diener's Satisfaction with Life Scale	3	Inc.	Cash transfer scheme had no significant impact on adolescent depression, but did have an impact on adult life satisfaction	Low	Yes
Allouche 2019	Psychological Well-Being and the Dynamics of Poverty	ZAF	2008-2014	Basic longitudinal	South African population aged 16+ (n=6,314)	Within-person **change** in household income per capita between waves	No	No	24	CES-D 10	3	Ben.	In South African panel data, within-person changes in income resulted in significant changes to CES-D score which were more pronounced for the poorest 20% of the population	Serious	Yes
Ambrey 2014	The causal effect of income on life satisfaction and the implications for valuing non-market goods	AUS	2002-2011	Basic longitudinal	Australian population (n=23,157)	Within-person **changes** in restricted windfall income (e.g. lottery win) and within-person **changes** in household income (latter used for meta-analysis for consistency with other studies)	No	No	12	Life satisfaction, 10 point scale	2	Ben.	Windfall payments were associated with increases in life satisfaction, though these were extremely small compared with the impact of changes in household income	Serious	Yes
Apouey 2015	Winning big but feeling no better? The effect of lottery prizes on physical and mental health	GBR	1996-2008	Other natural experiment	UK population (n=16,645)	Winning the lottery within the last one or two years, categorised as (1) Any win, (2) Big win (>£500) win and (3) Log(prize) **(increase)**	No	No	12	1. General Health Questionnaire (GHQ) 2. Life satisfaction, 7 point scale	12	Ben.	Winning a large lottery prize in the last 2 years was associated with a significant increase in both GHQ score and life satisfaction. The same was true for log(prize) when all prizes were included.	Serious	Yes

												Winning within the last one year was less clearly associated with improvement.		
Arata 2000	Coping with Technological Disaster: An Application of the Conservation of Resources Model to the Exxon Valdez Oil Spill	USA	1995	Basic cross-sectional	Members of the Cordova District Fishermen United in 1989 (n=125)	Experiencing an 'income loss spiral' following the oil spill, i.e. loss of income in 3 or more of the 6 years following the spill **(decrease)**	No	No	72	Symptom Checklist 90 Revised (SCL90-R) - subscale scores for anxiety, depression and PTSD	3	Ben.	Experiencing an income loss spiral following an Alaska oil spill was correlated with anxiety, depression and PTSD symptoms in fishermen	Critical	No
Araya 2003	Education and income: Which is more important for mental health?	CHL	1996-1998	Basic cross-sectional	Adult population living in private households of Santiago, Chile (n=3,870)	Household experienced a significant **decrease** in income over the past six months	No	No	6	Revised Clinical Interview Schedule (CIS-R)	1	Ben.	Experiencing a significant drop in household income in the last six months was associated with two-fold increase in likely common mental disorder	Critical	Yes
Asadullah 2012	Subjective well-being and relative poverty in rural Bangladesh	BGD	2008	Basic cross-sectional	Rural Bangladesh population (n=2,318)	Household did NOT experience adverse economic shock in the last 12 months **(decrease)**	No	No	12	Life satisfaction, 10 point scale	3	Ben.	Experiencing an adverse economic shock in the past 12 months was not associated with life satisfaction in rural Bangladesh	Critical	No
Assari 2018	Education and income predict future emotional well-being of whites but not blacks: A ten-year cohort	USA	1995-2005	Basic longitudinal	Population of continental United States with a telephone number (n=3,731)	**Change** in income compared with income 10 years prior at baseline	No	No	120	Positive and negative affect, measured using Mroczek & Kolarz scale	4	Ben.	Showed significant relationship between income change over 10 year period (2 measurements) and positive/negative affect for white participants in a panel, but not black participants	Serious	No
Baird 2013	Income Shocks and Adolescent Mental Health	MWI	2007-2010	Randomised trial of intervention	Never married females aged 13-22 living in Zomba district. Mixture of school dropouts and attendees (n=2,827)	Cash transfer both to parents and directly to girl, plus payment of secondary school fees direct to school - varied amounts. Payments were conditional on school attendance	Yes	Yes	12	GHQ	18	Inc.	Cash transfers reduced psychological distress among baseline schoolgirls, but not baseline dropouts. The large beneficial effects declined with increases in the transfer amount offered to the parents. Effects measured shortly	Some concerns	Yes

						except in unconditional arm of trial **(increase)**							after transfer ended were non-significant.		
Barbaglia 2015	Negative socioeconomic changes and mental disorders: a longitudinal study	NLD	2007-2012	Basic longitudinal	Dutch population aged 18-64 with at least 12h of paid work and no mental illness at baseline, not retired at follow-up (n=3,175)	Experiencing a substantial reduction in own or partner's income over five year period **(decrease)**	No	No	36	3 year incidence of any mental disorder, mood disorder or anxiety disorder, assessed using Composite International Diagnostic Interview (CIDI), ICD-10 criteria	2	Ben.	3 year incidence of mood disorders and anxiety disorders was higher for those who experienced a substantial income drop, though the latter was non-significant	Moderate	Yes
Becchetti 2010	The Effects of a Calamity on Income and Wellbeing of Poor Microfinance Borrowers: The Case of the 2004 Tsunami Shock	LKA	2007	Basic cross-sectional	Micro finance institution borrowers from Agro Micro Finance scheme living on the southern coast of Sri Lanka prior to 2004 tsunami (n=242)	The change in real income from pre-Tsunami to post-Tsunami before any refinancing was received from MFI **(decrease)**	No	Yes	26	Life satisfaction, 10 point scale	2	Ben.	No significant effect of the income change variable following tsunami on life satisfaction	Critical	No
Bedoya 2019	No Household Left Behind : Afghanistan Targeting the Ultra Poor Impact Evaluation	AFG	2016-2018	Randomised trial of intervention	Ultra-poor households in the poorest villages in four districts of Afghanistan's Balkh province (n=1,147)	TUP intervention: transfer of livestock; monthly cash transfer or 15 US$ per month for 12 months; basic training; health subsidy; fortnightly mentoring visits by social organisers and vets. Cash transfer aimed to be sufficient to support HH with basic food needs **(increase)**	Yes	Yes	12	1. CES-D 7, 2. Cohen's 4-item stress scale, 3. Happiness - 4 point scale, 4. Life satisfaction - 10 point scale	8	Ben.	There were significant effects of the TUP programme on life satisfaction, happiness, depression and stress, which were larger for women than men	Some concerns	Yes
Benzeval 2001	Income and health: the time dimension	GBR	1991-1997	Basic longitudinal	UK population (n=5,281)	1. Difference between income in wave 6 and wave 1. 2. Experiencing >30% income **increase** in same period. 3. >30% income **decrease** in same period	No	No	72	GHQ	2	Ben.	Income change across 6 years was not significantly associated with GHQ, but experiencing a large decrease of >30% had a greater impact	Serious	Yes

Biotteau 2019	Risk of Major Depressive Episodes After Separation: The Gender-Specific Contribution of the Income and Support Lost Through Union Dissolution	FRA	2006-2010	Basic longitudinal	French population aged 20-74 living in private households in relationship at baseline, not students (n=7,321)	Stagnation or **decrease** in adjusted income since 2006	No	No	48	Mini International Neuropsychiatric Interview: screens for major depressive episodes	2	Ben.	Income stagnation or decrease across 4 years was associated with increased odds of depression for women but not men	Serious	Yes
Bland 1996	Long-Term Psychological Effects of Natural Disasters	ITA	1987	Basic cross-sectional	Male employees of Olivetti Factory in Naples (n=174)	Experiencing financial loss associated with damages following the 1983-84 earthquakes **(decrease)**	No	Yes	0	SCL90-R, shortened version (64 items)	2	Ben.	Factory workers in Naples who experienced financial loss after 1983/94 earthquakes had poorer global mental health than those who did not	Critical	No
Blazquez-Cuesta 2013	Does income deprivation affect people's mental well-being?	DEU	2002-2010	Basic longitudinal	German population (n=5,783)	Within-person **changes** in household income	No	No	12	SF-12 mental component summary (MCS)	2	Ben.	Transitory income change was associated a significant change in SF-12 MCS for women, but not men	Serious	Yes
Bolden 2010	Predictors of Mental Health, Subjective Burden, and Rewards in Family Caregivers of Patients With Chronic Liver Disease	USA	Not reported: likely 2009	Basic cross-sectional	Family caregivers of people with chronic liver disease at a University-based hepatology practice in a large south-eastern metropolitan city in USA (n=73)	Income **decreased** since caring for the patient	No	No	72	1. CES-D 20, 2. Hamilton Anxiety Rating scale (HAM-A)	2	Ben.	Income loss after beginning caring for someone with chronic liver disease was correlated with CES-D score but not HAMA score	Critical	Yes
Bonanno 2007	What Predicts Psychological Resilience After Disaster? The Role of Demographics, Resources, and Life Stress	USA	2002	Basic cross-sectional	All adults in New York City and contiguous geographic areas in New York State, New Jersey and Lower Fairfield County in Connecticut (n=2,096)	Income **decrease** as a result of the September 11th terrorist attack	No	No	6	National Women's Study PTSD module (NWS-PTSD)	3	Ben.	Losing income as a result of 9/11 made New Yorkers less likely to be resilient against PTSD or mild/moderate trauma	Serious	Yes
Boyce 2009	Occupation, poverty and	GHA	2006	Basic cross-sectional	People with mental illness or	Income **increased** in last 6 months	No	No	6	Subjective improvement in	1	Ben.	There was no significant improvement in mental	Critical	Yes

	mental health improvement in Ghana				their caregivers who were enrolled in the BasicNeeds programme in Ghana (n=399)					mental health in last 6 months vs decline/no change			health for mentally ill Ghanaians following an income increase		
Boyce 2013	Money, well-being, and loss aversion: does an income loss have a greater effect on well-being than an equivalent income gain?	GBR	1998-2007	Basic longitudinal	UK population (n=20,570)	Within-person changes in household income, and income-loss dummy to differentiate between effects of **increases/decreases**	No	No	12	GHQ	2	Inc.	No significant improvement in GHQ with income change overall, but the relationship with income decreases did appear to be significant	Serious	No
Boyce 2018	The Great Recession and subjective well-being: How did the life satisfaction of people living in the United Kingdom change following the financial crisis?	GBR	2006-2010	Basic longitudinal	UK population (n=8,661)	**Change** in household income following the 2008 recession	No	No	36	Life satisfaction, 7 point scale	1	Ben.	Income change post-recession was associated with a change in life satisfaction	Serious	Yes
Boyd-Swan 2016	The earned income tax credit, mental health, and happiness	USA	1987-1994	Before and after (controlled)	US women aged 16-55 living in households whose primary language is English or Spanish, with education of high school degree or less (n=2,606)	Being affected by changes to Earned Income Tax Credit (EITC) from 1991 to 1994. Resulted in more households receiving EITC and also an **increased** amount being available for families with 2 or more children for the first time.	No	No	0	1. CES-D 11, 2. Happiness, 7 point scale	2	Ben.	Expansion of earned income tax credit was not significantly associated with CES-D score or happiness	Serious	Yes
Brenner 2014	Organizational Downsizing and Depressive Symptoms in the European	FRA HUN SWE GBR	2009-2011	Basic cross-sectional	Workers in small, medium & large companies who experienced recent large downsizing of	If reported **decreased** income and benefits after company downsizing	No	Yes	0	SCL90-R, depression subscale	1	Ben.	Having decreased income and benefits after experiencing a large downsizing events was	Critical	Yes

	Recession: The Experience of Workers in France, Hungary, Sweden and the United Kingdom				at least 10% of workforce in last 2 years, and workers who had not (recruitment varied between countries) (n=641)								significantly associated with depression symptoms		
Brickman 1978	Lottery Winners and Accident Victims: Is Happiness Relative?	USA	Not reported: likely 1977	Basic cross-sectional	"Major" winners of Illinois State Lottery and controls: people living in approximately the same areas of the city as the winners (n=152)	Being a major winner on the Illinois lottery **(increase)**. Among 22 people interviewed, 7 won $1 million, 6 won $400,000, 2 won $300,000, 4 won $100,000, and 3 won $50,000.	No	No	0	Happiness, 6 point scale	1	Har.	No significant effect of lottery wins on happiness	Critical	No
Brock 1985	From wife to widow: Role transition in the elderly	USA	Not reported: likely 1981	Basic cross-sectional	Widows whose husbands had died 11-13 months prior to data collection, registered at 20 funeral homes in large metropolitan city in southwestern USA (n=92)	If income **decreased** by $5,000-$10,000 per year after widowhood or by $15,000-$35,000 per year after widowhood	No	No	12.3	Bradburn Affect Balance Scale	2	Ben.	Income loss after widowhood was associated with poorer scores on the affect balance scale	Critical	No
Burke 1986	Reemployment on a poorer job after a plant closing	CAN	1983	Basic cross-sectional	Former employees at the Canadian Admiral plant who became re-employed (n=44)	Drop in salary between previous job with Admiral and new job **(decrease)**	No	No	16	1. Specific life satisfaction: asked about present life, 10 bipolar item domains combined into single index, 2. Global life satisfaction: two items combined to form an index measure, one on happiness and one on life satisfaction	2	Ben.	After closure of Admiral plant in Canada, re-employment in a job with smaller hourly salary was correlated with reduced specific life satisfaction but not global life satisfaction	Critical	No
Burmaster 2015	Impact of a private sector living wage intervention on depressive	DOM	2011	Other natural experiment	Low-paid, hourly wage workers at the intervention factory and workers at a	Being employed at a factory which operated a living wage on opening in April 2010, which 'early hire'	Yes	Yes	16	CES-D 20	3	Ben.	Working in a factory with a living wage intervention was associated with better CES-D scores	Serious	Yes

	symptoms among apparel workers in the Dominican Republic: a quasi-experimental study				comparison factory in similar geographical area (n=204)	employees did not know in advance of being getting the job. Included other workplace improvements such as right to organise, higher labour standards, work hour restrictions etc. **(increase)**								
Buttke 2012	Community Assessment for Public Health Emergency Response (CASPER) one year following the Gulf Coast oil spill: Alabama and Mississippi, 2011	USA	2011	Repeat cross-sectional	Adult population of Gulf Coast counties of Alabama and Mississippi (n=596)	If the oil spill resulted in an income **decrease**	No	No	18	1. Experiencing 14 or more mentally unhealthy days in last 30 (CDC Healthy Days measure), 2. Patient Health Questionnaire-2 (PHQ-2) for depressive symptoms, 3. Generalized Anxiety Disorder-2 (GAD-2)	9	Ben.	In areas affected by a Gulf Coast oil spill, people whose incomes decreased as a result scored more poorly on three mental health measures than others	Critical	Yes
Cai 2016	Permanent income and subjective well-being	CHN	2006-2009	Basic longitudinal	Adults aged 18-60 in rural households in 64 Chinese villages across four countries (n=960)	Within-person **changes** in household income per capita	No	No	36	Life satisfaction, 5 point scale	1	Ben.	There was no significant effect of income changes on life satisfaction	Serious	Yes
Casey 2004	Maternal Depression, Changing Public Assistance, Food Security, and Child Health Status	USA	2000-2001	Basic cross-sectional	Primary adult caregivers accompanying children 0-3 years presenting at hospital EDs and primary care clinics in Baltimore, Maryland, Boston, Minneapolis, Minnesota and Washington DC (n=845)	Changes in welfare payments during the 12 months preceding the interview: 1) sanctioned: terminated or reduced by full family or partial sanctions for failure to comply with behavioral requirements; 2) **decreased**: benefits decreased administratively due to increased income or decreased expense (eg	No	Yes	12	3-item maternal depression screen developed by Kemper et al used to screen for maternal depression	2	Ben.	Reduction in benefits due to change in circumstances was associated with worsened mental health. The effect of a reduction due to sanctions was smaller and non-significant	Critical	Yes

						from employment or changes in marital status)								
Cesarini 2016	Wealth, Health, And Child Development: Evidence From Administrative Data On Swedish Lottery Players	SWE	1986-2010	Other natural experiment	1. Swedes who held prize-linked savings accounts from 1986 to 2003 and won pre-2006; 2. Subscribers to the Kombi monthly ticket lottery from 1998 to 2010; 3. Participants in the Triss scratch ticket lottery run by Swedish Govt owned gambling operator from 1994 to 2010 (n=57,988)	Uses the amount won in the 3 lotteries as the exposure. Amounts varied between lotteries, from 1,000 to 2 million SEK. **(increase)**	No	No	288	The sum of the defined daily doses of mental health related drugs prescribed to someone between 2006 and 2010	1	Ben.	A lottery win was associated with a small reduction in the consumption of mental health drugs	Low	No
Cheung 2015	When Does Money Matter Most? Examining the Association between Income and Life Satisfaction over the Life Course	CHE	1999-2007	Basic longitudinal	Swiss population aged 14+ (n=13,405)	Within-person **changes** in household income	No	No	12	Life satisfaction, 11 point scale	1	Ben.	Within person income change was significantly associated with life satisfaction in Swiss population	Moderate	Yes
Chien 2004	Quality of life and related risk factors in a Taiwanese Village population 21 months after an earthquake	TWN	2001	Basic cross-sectional	Residents of Tong-Chi village aged 16 or older (n=461)	Experiencing prominent financial loss immediately after the earthquake in September 1999 **(decrease)**	No	No	21	SF-36 mental health subscale	1	Ben.	Those experiencing prominent financial loss after an earthquake in Taiwan had poorer mental health on SF-36 mental health subscale	Critical	Yes
Chin 2018	Quality of life at 6 years after occupational injury	TWN	2009-2015	Basic longitudinal	Taiwanese workers hospitalised for 3 or more days due to occupational injury between 1st Feb and 31st	If current monthly salary (6 years post-accident) is <100% of that before the occupational injury **(decrease)**	No	No	72	Psychological domain of the Taiwanese version of WHOQOL-BREF	1	Ben.	There was a statistically significant worsening of the psychological domain of QoL if the salary received 6 years after an occupational injury was	Serious	Yes

					August 2009 (n=563)								<100% of what it was pre-injury		
Chrostek 2016	An Empirical Investigation into the Determinants and Persistence of Happiness and Life Evaluation	POL	2003-2011	Basic longitudinal	Polish population (n=3,706)	Within-person **changes** in household income	No	No	12	1. Happiness - 4 point scale, 2. Life satisfaction/evaluatio n - 7 point scale	2	Inc.	Changes in household income were associated with happiness but not life satisfaction	Serious	No
Clark 2016	Adaptation to poverty in long-run panel data	DEU	1985-2012	Basic longitudinal	German population (n=53,867)	Within-person **changes** in whether someone is above/below the poverty line, defined as 60% of country-level median equivalent household income. Also, fixed effects of poverty that started within last 0-1 years i.e. recent move into poverty **(decrease)**	Yes	No	12	Life satisfaction, 11 point scale	6	Ben.	Changes in poverty line status was significantly associated with life satisfaction, and experiencing a recent move below the poverty line was associated with larger effects	Serious	Yes
Clingingsmit h 2016	Negative emotions, income, and welfare: Causal estimates from the PSID	USA	2001-2013	Basic longitudinal	US population aged 23+ employed in at least one year of data collection (n=10,156)	Within-person **changes** in total family income	No	No	12	1. Kessler 6 (K6) index of non-specific psychological distress, 2. Life satisfaction, 5 point scale	5	Ben.	Within-person changes in family income was associated with worsening of psychological distress and life satisfaction. Instrumental variable analysis using cross-industry wage variation as an instrument found a larger effect size than self-reported income	Serious	Yes
Coley 2014	Low-income women's employment experiences and their financial, personal, and family well-being	USA	1999-2006	Basic longitudinal	Low-income mothers in moderate- and high-poverty neighbourhoods in Boston, Chicago and San Antonio (n=1,586)	Within-person **changes** in weekly wages	No	No	54	Brief Symptom Inventory (BSI), 18 item version	1	Ben.	No significant. effect of within-person changes in weekly wages on depression for low income mothers	Serious	No
Costello 2010	Association of Family Income Supplements in	USA	1993-2006	Other natural experiment	Children aged 9, 11 and 13 in 1993 living in 11	Receiving an income supplement as a member of the Eastern	No	Yes	36	Child and Adolescent Psychiatric Assessment (CAPA) -	5	Ben.	The likelihood of having any psychiatric disorder reduced in Native	Moderate	Yes

	Adolescence With Development of Psychiatric and Substance Use Disorders in Adulthood Among an American Indian Population				counties in North Carolina (n=1,185)	Band of Cherokees when a casino was opened on their reservation in 1996. Every tribal member receives a % of the profits, paid every 6 months **(increase)**				Presence of any DSM-IV psychiatric disorder, any behavioral disorder (conduct, oppositional, or antisocial personality disorder), any emotional disorder (depressive or anxiety disorders) in early adulthood			American 18 year olds who received cash transfers from casino proceeds. The reduction was largely attributable to changes in substance use related disorders. Larger transfers resulted in greater reductions		
Courtin 2018	Conditional Cash Transfers And Health Of Low-Income Families In The US: Evaluating The Family Rewards Experiment	USA	2007-2009	Randomised trial of intervention	Low income families with children living in six community districts in the Bronx, Brooklyn and Manhattan in 2007 (n=2,043)	Average conditional cash transfer amount was $8,674 over 3 years of the programme, approx. 22% increase in average monthly income. Cash rewards were offered for 22 activities in three core areas: Education incentives, health incentives, workforce incentives **(increase)**	Yes	Yes	18	1. Subjectively experiencing serious psychological distress in past month, 2. Kessler Psychological Distress scale (K10)	2	Ben.	There was no significant effect of the conditional cash transfer on two measures of distress at 18 months	Some concerns	Yes
Daley 2017	Income and the mental health of Canadian mothers: Evidence from the Universal Child Care Benefit	CAN	2003-2008	Before and after (controlled)	Mothers aged 18-59 with children aged <11 in all Canadian provinces except those on Crown land and First Nations reserves (n=26,886)	Exposure to the Universal Child Care Benefit, introduced in 2006, which paid $100/month ($1200 annually) for each child <6 years **(increase)**	No	No	0	1. Self-assessed mental health, 5 point scale, 2. Stress on a daily basis, 5 point scale, 3. Life satisfaction, 5 point scale	3	Inc.	Introduction of a universal child benefit of modest amount ($100/month) had a significant effect on life satisfaction but not on self-assessed mental health or stress	Moderate	No
Dang 2019	Did the Poor Adapt to Their Circumstances? Evidence from Long-run Russian Panel Data	RUS	2001-2017	Basic longitudinal	Russian population (n=38,696)	Within-person **changes** in whether someone is above/below the poverty line, defined as 60% of median per capita income. Also, fixed effects of poverty that started within last	Yes	No	12	Life satisfaction, 5 point scale	4	Ben.	Changes in poverty line status were significantly associated with life satisfaction, slightly more for women than men. A recent move into poverty was associated with a larger effect.	Serious	Yes

						0-1 years i.e. recent move into poverty **(decrease)**								
Dearing 2004	Implications of family income dynamics for women's depressive symptoms during the first 3 years after childbirth	USA	1991-1994	Basic longitudinal	Women 18+ living in or near 10 urban and suburban sites in US who gave birth to full-term, healthy newborns from January to November 1991 (n=1,351)	Within-person **changes** in family income. Also, within-person changes in poverty status (calculated by dividing total family income by poverty threshold for the appropriate family size)	Yes	No	12	CES-D 20	3	Ben.	Income changes and moves across the poverty line in the 3 years after childbirth were significantly associated with odds of depression	Serious	Yes
Diener 1993	The Relationship Between Income And Subjective Well-Being - Relative Or Absolute	USA	1971-1984	Basic longitudinal	US population in 1971 (n=4,942)	**Decrease** OR **increase** in income of >0.5 SDs compared with baseline	No	No	120	General Well-Being Schedule	4	Har.	There was no significant difference in wellbeing scores crossing income change groups (over 10 years) and whether someone is low, medium or high income earner	Critical	No
Dolan 2013	Moving Up and Sliding Down: An Empirical Assessment of the Effect of Social Mobility on Subjective Wellbeing	GBR	1980-2004	Basic longitudinal	Children born between 5th and 11th April in 1970 in England, Scotland, Wales and Northern Ireland, and children born on those days outside the country identified from school registers at later ages (n=5,383)	Difference between household income at age 30 or 34 and household income at age 10, classed as either upwardly mobile **(increase)** or downwardly mobile **(decrease)**	No	No	288	1. Life satisfaction, 11 point scale, 2. Rutter Malaise Inventory, 3. GHQ, 4. Kessler stress scale, but only 4 questions rather than usual 6 or 10	12	Ben.	Upward income trajectories from age 10 to 30/34 were not significantly associated with life satisfaction, distress, common mental disorder or stress, but downward income trajectories were significantly associated across all measures	Serious	No
Dorsett 2014	Human Well-Being and In-Work Benefits: A Randomized Controlled Trial	GBR	2003-2010	Randomised trial of intervention	Out of work single mothers on Income Support OR single mothers working part-time in low-paid jobs that qualified them for Working Tax Credit (living	Employment and Retention Advancement (ERA). Included (1) post-employment job coaching (2) cash incentives for staying and advancing in work and (3) training opportunities, including	No	Yes	33	Life satisfaction, 5 point scale	2	Inc.	Enhanced in-work income support which included cash incentives was successful in improving earnings but resulted in no significant improvement in life satisfaction at Year 2 and a worsening of life satisfaction at Year 5	Some concerns	Yes

					outside of London) (n=2,841)	help with tuition costs and cash rewards for completing training courses while employed **(increase)**									
Elwell-Sutton 2019	Socioeconomic position and depression in South African adults with long-term health conditions: a longitudinal study of causal pathways	ZAF	2011-2012	Basic longitudinal	Adults aged 18+ with hypertension, diabetes, chronic respiratory disease or depression who attended 38 primary care clinics in the Western Cape of South Africa (n=3,904)	Income **change** between baseline and follow-up	No	No	14	CES-D 10	1	Ben.	Change in income was associated with reduction (improvement) in CES-D score in South Africans with chronic illnesses	Serious	No
Erixson 2017	Health responses to a wealth shock: evidence from a Swedish tax reform	SWE	2003-2005	Before and after (controlled)	Heirs and beneficiaries of all deceased Swedes over the period 2003 to 2005 (n=79,801)	Abolition of inheritance tax in December 2004, which resulted in larger than expected inheritances. Classed as affected by reform if inheritance exceeds what had previously been the tax threshold **(increase)**	No	No	0	If hospitalised in given year for conditions categorised in ICD chapter on mental and behavioural disorders	2	Ben.	An inheritance shock related to tax abolition in Sweden was not significantly associated with hospitalisations for mental or behavioural disorders	Low	Yes
Evans 2005	Adaptation, response-shift and quality of life ratings in mentally well and unwell groups	GBR	1994-1996, 1999-2001	Basic longitudinal	1. Patients aged 18-65 with diagnosed psychotic illness of at least 2 years duration at 3 London hospital sites and 1 Manchester site; 2. Adults aged 18-65 living in community residences in a deprived area of South Manchester (n=1,075)	Experiencing an **increase** or **decrease** in monthly income from earnings and/or benefits from baseline to follow-up	No	No	24	Subjective 'general' quality of life measured by Andrews and Withey's seven-point delighted-terrible scale	2	Ben.	An income increase was significantly associated with improved life satisfaction, but income decreases were not	Critical	Yes

Evans 2011	Giving Mom a Break: The Impact of Higher EITC Payments on Maternal Health	USA	1993-2001	Before and after (controlled)	Women aged 21-40 with children eligible to receive Earned Income Tax Credit (n=82,907)	1993 Earned Income Tax Credit (EITC) expansion - first meaningful separation of benefit levels for families based on number of children, and families of two or more children received substantially more in payments **(increase)**	No	No	0	1. Any bad mental health days in past 30 days, 2. Number of bad mental health days in past month	2	Ben.	Expansion of earned income tax credit which included additional monies for larger families for first time was significantly associated with reduction in number of bad mental health days, but not likelihood of having any bad MH days	Serious	Yes
Fang 2017	Does everyone exhibit loss aversion? Evidence from a panel quantile regression analysis of subjective well-being in Japan	JPN	2009-2013	Basic longitudinal	Japanese population aged 20-69 (n=3,695)	Within-person changes in household income. Also: percentage change in income compared with previous year, included all on one scale and as two separate variables, one for positive changes **(increase)** and one for negative changes **(decrease)**	No	No	12	Happiness, 11 point scale	9	Ben.	Income change in Japanese adults was associated with a significant difference in happiness in the following year. When split into increases and decreases, only decreases were significant	Serious	Yes
Feeny 2014	Are Poor People Less Happy? Findings from Melanesia	SLB	2012-2013	Basic cross-sectional	Population of Solomon Islands and Vanuatu (n=619)	If the total amount of money that comes into the house has gone up **(increase)** or down **(decrease)** in the past 2 years, measured on 5 point Likert scale	No	No	24	Happiness, 10 point scale	1	Ben.	There was a significant effect of how income has changed compared with two years ago on happiness	Critical	No
Fenn 2015	The role of unconditional cash transfers during a nutritional emergency in Maradi region, Niger: a pre-post intervention observational study	NER	2012	Before and after (no control)	Households with a non-acutely malnourished child aged 6-36 months in the Aguie district of Maradi, Niger (n=412)	Receiving a cash transfer from Save the Children - amount estimated to meet 80% of the energy requirements for an average-sized household, equivalent to about £32/household/month, rising to about £43 in June when food prices increased. Timed to	Yes	No	5	Self-Reporting Questionnaire (SRQ-19)	1	Ben.	Cash transfer offered to families in Niger during rainy season significantly reduced maternal depression	Critical	No

						cover the lean and rainy seasons when households were unable to farm **(increase)**								
Fernald 2011	Effect of Ecuador's cash transfer program (Bono de Desarrollo Humano) on child development in infants and toddlers: a randomized effectiveness trial	ECU	2003-2006	Randomised trial of intervention	Low-income mothers in bottom two poverty quintiles with children <16 years (n=959)	$15/month cash transfer **(increase)**. Equated to approx. 6-10% of average HH pre-transfer income. No difference according to number of children.	No	No	17	CES-D 20	1	Har.	There was no significant effect of a small Ecuadorian cash transfer scheme on maternal depression	Some concerns	Yes
Frijters 2011	Life Satisfaction Dynamics with Quarterly Life Event Data	AUS	2002-2007	Longitudinal	Australian population aged 16+ (n=13,388)	Self-defined major worsening/**decrease** (e.g., went bankrupt) or improvement/**increase** (e.g., won lottery, received an inheritance) in financial situation within last 12 months.	No	No	12	Life satisfaction, 11 point scale	2	Ben.	Both major improvements and major worsenings of income were significantly associated with life satisfaction, but there was also an element of anticipation and adaptation such that these impacts don't last	Serious	Yes
Frijters 2012	The Optimality of Tax Transfers: What does Life Satisfaction Data Tell Us?	AUS	2001-2007	Basic longitudinal	Australian population aged 25+, not students (n=9,518)	Within-person **change** in household income	No	No	12	Life satisfaction, 11 point scale	1	Ben.	There was a significant effect of household income changes on life satisfaction in the Australian population	Serious	Yes
Galama 2017	Wealthier, Happier and More Self-Sufficient: When Anti-Poverty Programs Improve Economic and Subjective Wellbeing at a	COL	2010	Other natural experiment	Low-income Colombian households with children living in one of the fourteen cities participating in FAU conditional cash transfer programme (n=472)	Being estimated to be eligible for the FAU conditional cash transfer programme. Conditional on young children attending regular medical check-ups and school-age children enrolling and continually attending school. Provided	Yes	Yes	36	1. Life satisfaction, 4 point scale, 2. Happiness (not clearly defined)	1	Ben.	Cash transfer was significantly associated with wellbeing, but the size of the effect was very sensitive to the choice of cut-off used in analysis	Serious	Yes

	Reduced Cost to Taxpayers					nutritional subsidy and educational subsidy **(increase)**								
Gassman-Pines 2006	Five-year effects of an anti-poverty program on marriage among never-married mothers	USA	1994-1997	Randomised trial of intervention	Unmarried women aged 18+ with incomes at or below 150% of the federal poverty line living in two poor neighbourhoods in Milwaukee, Wisconsin (n=295)	New Hope Project: included wage supplement **(increase)** to increase participants' income up to poverty threshold, affordable health insurance, childcare subsidies, and community service jobs for those unable to find private employment. All conditional on working 30 hours/week.	Yes	Yes	24	CES-D 20	1	Ben.	There was no significant impact of the New Hope anti-poverty program on depression in never married mothers	Some concerns	Yes
Gennetian 2002	Children and Welfare Reform: A View from an Experimental Welfare Program in Minnesota	USA	1994-1997	Randomised trial of intervention	Single mothers of children aged 2-9 years who were long-term welfare recipient families in urban counties of Minnesota (n=587)	Minnesota Family Investment Program (MFIP): (1) working families received supplemental benefits **(increase)** while working until income reached approx. 140% of poverty line (2) child-care expenses were paid directly to provider (3) single parents who received benefits for 2 of past 3 years had to participate in employment and training activities unless working >30hrs/wk or had good cause not to	Yes	Yes	36	CES-D 20	2	Ben.	New more generous benefits were not associated with CES-D score in single mothers who were long-term welfare recipients, except a subset of individuals who only received incentives without obligations/conditionality	High	Yes
Grattan 2017	"Bouncing back" after the Deepwater Horizon oil spill	USA	2010-2011	Basic longitudinal	Adults from Franklin County who experienced an indirect impact or exposure to	Experiencing an income loss **(decrease)** due to the oil spill and indicating that the biggest impact of the	No	No	12	Profile of Mood States (POMS)	1	Ben.	Experiencing an income loss due to the Deepwater oil spill was significantly associated with poorer mental health	Critical	No

					Deepwater oil spill (e.g. worked in fishing or tourism industries) AND adults from Baldwin County who lived or worked in a community where spilled oil reached the shoreline i.e. direct impact (n=133)	oil spill on their life was economic								
Green 2016	Does poverty alleviation decrease depression symptoms in post-conflict settings? A cluster-randomized trial of microenterprise assistance in Northern Uganda	UGA	2009-2011	Randomised trial of intervention	The poorest and most vulnerable community members living in villages, transit sites and displacement camps in Gulu and Kitgum districts where population >400 OR at least 80 households were present (n=1,732)	Women's Income Generating Support (WINGS) program, three core components: (i) 5 days of business skills training designed for illiterate populations, (ii) an individual start-up grant of roughly US$150 **(increase)**, and (iii) 3 to 5 visits over approximately 18 months by trained community workers who provided business advice and encouragement to use the grant for business development	Yes	Yes	16	Modified version of the Acholi Psychosocial Assessment Instrument (APAI) depression subscale	3	Ben.	There was no effect of a poverty alleviation program on symptoms of depression in vulnerable people (mostly women) in post-conflict setting	Low	Yes
Gros 2019	Household-level effects of providing forecast-based cash in anticipation of extreme weather events: Quasi-experimental evidence from	BGD	2016-2017	Other natural experiment	Those living in vulnerable households in flood-risk areas of Bangladesh (n=348)	Forecast-based Financing (FbF) project beneficiaries: involved an unconditional cash transfer of BFT 5000 (equivalent of $60), just below the monthly average value of 2016/17 Minimum Expenditure Basket for	Yes	No	1	1. Subjective unhappiness, 3 point scale, 2. Subjective depressive mood or anxiety in past 7 days, 3 point scale	2	Ben.	Groups offered forecast based cash transfers prior to flooding in Bangladesh more likely to be happy and less likely to be depressed after the flood occured	Serious	Yes

	humanitarian interventions in the 2017 floods in Bangladesh					Northwest Bangladesh (BDT 5400). Triggered in the event of a forecast of an extreme weather event - in this study, it was triggered in advance of 2017 floods, varying from 3-7 days in advance of the flood peak **(increase)**								
Gulal 2019	The Impact of Minimum Wages on Well-Being: Evidence from a Quasi-experiment in Germany	DEU	2012-2016	Before and after (controlled)	German population aged 18-65 employed in 2014 or 2015 (n=2,944)	Introduction of a minimum wage of 8.5 euro/hr on Jan 1st 2015 in Germany - prior to this no minimum wage existed **(increase)**	No	No	12	Life satisfaction, 11 point scale	1	Ben.	Introduction of a minimum wage in Germany was associated with increased life satisfaction, and effects were larger in East Germany (which was overall more deprived)	Serious	Yes
Handa 2014	Livelihood Empowerment against Poverty Program Impact Evaluation	GHA	2010-2012	Non-randomised trial of intervention	Families living in poverty in Ghana with a vulnerable household member (orphaned/vulnerable child, elderly poor, unable to work due to disability) (n=1,518)	LEAP program: included both a cash transfer element **(increase)** and enrollment into a National Health Insurance scheme. Median transfer level per HH was GHc 15, only 7% of upper poverty line and 11% of lower poverty line	No	Yes	24	Happiness, binary	3	Ben.	A small cash transfer in Ghana accompanied by a national health insurance scheme was effective overall at increasing happiness, but this was driven by a significant effect for women rather than men	Serious	Yes
Hanly 2015	Examining the role of subjective and objective burden in carer health-related quality of life: the case of colorectal cancer	IRL	2010	Basic cross-sectional	Informal carers of survivors of colorectal cancer in Ireland (n=136)	Experiencing an income **decrease** since commencing caregiving	No	No	36	SF-12 MCS	1	Ben.	Income loss after beginning caring for a person with colon cancer was associated with poorer mental health	Critical	No
Hasan 2016	Does Happiness Adapt to Increase in Income? Evidence from Pakistan Socio-	PAK	1998-2001	Basic longitudinal	Pakistan population (n=6,749)	Within-person **changes** in nominal and real income	No	No	36	Happiness, 3 point scale	2	Ben.	Household income changes were significantly associated with happiness	Serious	No

	economic Survey (1998-2001)														
Haushofer 2016	The Short-term Impact of Unconditional Cash Transfers to the Poor: Experimental Evidence from Kenya	KEN	2011-2012	Randomised trial of intervention	Individuals living in poverty in the poorest areas of Rarieda, Kenya (n=1,474)	Unconditional cash transfer of KES 25,200 ($404) **(increase)**. Three treatment arms: 1. Transfer given to male vs female as head of household; 2. Transfer given as lump-sum or sequence of 9 monthly payments of KES 2,800 ($45); 3. Standard vs larger transfer of additional KES 70,000 ($1,121 PPP), paid in seven monthly installments of KES 10,000 ($160 PPP) each. The total transfer amount received by these households was KES 95,200 ($1,525 PPP, $1,000 nominal)	Yes	No	9	1. CES-D 20, 2. PSS, standardised total from 4 elements, 3. Happiness, 4 point scale, 4. Life satisfaction, 10 point scale	8	Ben.	An unconditional cash transfer in Kenya was associated with improvements to all outcomes including depression, stress, happiness and life satisfaction. There was less clear evidence that a very large top-up to transfer may be better than a basic transfer.	Some concerns	Yes
Hilton 2006	Loss and Depression in Cohabiting and Noncohabiting Custodial Single Parents	USA	1992	Basic cross-sectional	Divorced or separated single parent in USA (n=728)	Experiencing a **change** in finances compared with prior to marital separation, rated on 5-point Likert scale.	No	No	24	Modified version of CES-D	2	Ben.	Having a lower income than pre-divorce was correlated with depression for single parents	Critical	No
Hjelm 2017	Poverty and perceived stress: Evidence from two unconditional cash transfer programs in Zambia	ZMB	2010-2013 (CGP), 2011-2014 (MCP)	Randomised trial of intervention	CGP: Women from 90 communities in Kaputa, Kalabo and Shang'ombo with children <5 years; MCP: Women in 92 communities in Luwingu and Serenje identified as most vulnerable in the society (n=4,763)	Zambia Child Grant Program (CGP) and Zambia Multiple Category Cash Transfer Program (MCP): both unconditional, government run unconditional cash transfer programs **(increase)**, treatment households received amount equivalent to 11 US$/month,	Yes	No	36	PSS	2	Inc.	Two unconditional cash transfer programmes in Zambia were not significantly associated with improvement in perceived stress for female caregivers of children	Some concerns	Yes

						estimated to be sufficent to cover cost of one meal per person per day in an average sized household								
Horn 2017	Do Minimum Wage Increases Influence Worker Health?	USA	1993-2014	Before and after (controlled)	US workers aged 18-54 with no college degree (n=1,361,382)	Fixed effects of area-level minimum wage, with one-year lag **(changes)**	No	No	0	# of bad mental health days in past month	2	Inc.	State-level minimum wage changes were not significantly associated with # of bad mental health days	Serious	Yes
Huang 2015	Associations between economic loss, financial strain and the psychological status of Wenchuan earthquake survivors	CHN	2012	Basic cross-sectional	Adults aged 18+ living in five communities near the epicentre of the Wenchuan earthquake (n=306)	Impact of the earthquake on income, rated on 5-point Likert scale **(decrease)**	No	No	48	1. Diener's 5-item Satisfaction with Life Scale, 2. CES-D 10	4	Ben.	Income loss following an earthquake in China was significantly associated with depression but not life satisfaction	Critical	No
Ishiguro 2019	Gender-Based Risk and Protective Factors for Psychological Distress in the Midterm Recovery Period Following the Great East Japan Earthquake	JPN	2012	Basic cross-sectional	Adults 19-65 who had remained living in their home despite earthquake damage in Ishinomaki City; students excluded (n=1,527)	If experienced changes in family income as a result of the earthquake **(decrease)**	No	No	21	K6	2	Ben.	Change in income due to earthquake was associated with increased odds of psychological distress 14-21 months later, with a larger effect for women than men	Critical	Yes
Jalal 2015	Food Insecurity Mediates the Effect of a Poverty-Alleviation Program on Psychosocial Health among the Ultra-Poor in Bangladesh	BGD	2002-2006	Non-randomised trial of intervention	Women in ultra-poor households in Rangpur, Nilphamari and Kurigram who were eligible to earn income (n=209)	CFPR-TUP: consisted of a subsistence allowance (Tk 10/day [equiv of $0.17] for 12-15 months), transfer of income-earning assets (ranged in value from equiv of $50-$150), training on basic entrepreneurial skills, and health support **(increase)**	Yes	Yes	15	1. Positive and Negative Affect Schedule, 2. World Health Organisation Self-Reporting Questionnaire for psychological distress	2	Ben.	A poverty alleviation program for the ultra poor (which included business skills) had an impact on distress and wellbeing	Serious	No

Junna 2019	The Association Between Income and Psychotropic Drug Purchases: Individual Fixed Effects Analysis of Annual Longitudinal Data in 2003-2013	FIN	2003-2013	Basic longitudinal	Finnish residents aged 30-62 with taxable personal income (n=337,456)	Within-person **change** in individual income	No	No	12	If purchased psychotropic medication within a year (medications for mood, anxiety and other mental disorders). Included antipsychotics, anxioloytics, hypnotics and sedatives, and antidepressants. Excluded anti-dementia drugs and psychostimulants.	2	Har.	There was no significant effect of individual income changes on mental health prescriptions in the Finnish population	Moderate	Yes
Kendall 2019	The association between income, wealth, economic security perception, and health: a longitudinal Australian study	AUS	2002-2010	Basic longitudinal	Australian population 15+ (n=16,682)	Within-person **change** in disposable household income	No	No	48	SF-36 mental health subscale	1	Ben.	Income changes were significantly associated with SF-36 mental health scores	Serious	No
Kiernan 2018	The Great Recession and Mental Health: the Effect of Income Loss on the Psychological Health of Young Mothers	IRL	2008-2013	Basic longitudinal	Female caregivers in Ireland with children aged 9 months at baseline (n=6,821)	Within-person **change** in household income	No	No	24	1. CES-D 8, 2. Self-reported treatment for depression or anxiety	3	Ben.	Income changes in mothers of infants in Ireland were significantly associated with changes in depression	Serious	Yes
Kikuchi 2013	Structural equation modeling of factors contributing to quality of life in Japanese patients with multiple sclerosis	JPN	2007	Basic cross-sectional	Japanese inpatients and outpatients diagnosed with multiple sclerosis (MS) at eight University hospitals (n=163)	If income **increased** OR **decreased** following onset of MS	No	No	124.8	Nottingham Adjustment Scale-Japanese (NAS-J)	1	Ben.	Experiencing an income change after diagnosis of MS was associated with poorer global mental health	Critical	No

Kilburn 2016	Effects of a Large-Scale Unconditional Cash Transfer Program on Mental Health Outcomes of Young People in Kenya	KEN	2007-2011	Randomised trial of intervention	Poor households that have at least one orphan or vulnerable child<18 in seven districts in Kenya; respondents aged 15-24 at follow-up (n=1,960)	Cash Transfer for Orphans and Vulnerable Children (CT-OVC): unconditional cash transfer **(increase)** of approx. $20/month (mean monthly wage per adult in sample at baseline was $22/month)	Yes	No	48	CES-D 10	3	Inc.	A Kenyan cash transfer aimed at orphans and vulnerable children reduced likelihood of depression overall, but the effect was concentrated in men (particularly young men)	Low	Yes
Kilburn 2018	Paying for Happiness: Experimental Results from a Large Cash Transfer Program in Malawi	MWI	2013-2014	Randomised trial of intervention	Ultra-poor and labour-constrained households in Malawi (n=2,919)	SCTP: monthly unconditional cash transfer **(increase)** to eligible households which varies depending upon the number of members in the household. Average value of the transfer is 18 percent of pre-program consumption.	No	No	17	Quality of life, 8 items combined into single index	1	Ben.	A Malawian cash transfer programme for ultra poor/labour constrained households was significantly associated with improved quality of life	Some concerns	Yes
Klein 2017	Income and health-related quality of life among prostate cancer patients over a one-year period after radical prostatectomy: a linear mixed model analysis	DEU	2013-2015	Basic longitudinal	All prostate cancer patients aged <65 in Germany attending a radical prostatectomy in two acute care hospitals in Hamburg (n=201)	Within-person **change** in household income	No	No	6	European Organization for Research and Treatment of Cancer quality of life questionnaire (EORTC QLQ-C30)	1	Ben.	A change in household income after radical prostatectomy was significantly associated with quality of life	Serious	No
Koltai 2018	Financial circumstances, mastery, and mental health: Taking unobserved time-stable influences into account	CAN	2011-2015	Basic longitudinal	Canadian residents aged 18+ working a paid job or operating an income-producing business (n=3,805)	Within-person **change** in whether someone's equivalised household income is below or above the Low-Income Measure Threshold, defined by Statistics Canada	Yes	No	24	K6	1	Ben.	There was no effect of changes in low income status on K6 scores in Canadian working adults with fixed-effects modelling, though random-effects models did find a significant effect	Moderate	Yes

Krauss 2013	Subjective wellbeing in Colombia : some insights on vulnerability, job security, and relative incomes	COL	2010	Basic cross-sectional	Colombian population aged 18-65 (n=2,348)	If experienced an income **increase** in last two years	No	No	24	Life satisfaction, 11 point scale (Cantril self-anchoring scale)	1	Ben.	Reporting an income increase in the last 2 years was significantly associated with life satisfaction	Critical	No
Kuhn 2011	The Effects of Lottery Prizes on Winners and Their Neighbors: Evidence from the Dutch Postcode Lottery	NLD	2003-2006	Basic cross-sectional	Winners: all households in PCL-winning postcodes from six months before September 2003 to six months before July 2006; Controls: neighbouring households which did not win during same period (n=1,879)	Winning the Dutch postcode lottery in last 6 months **(increase)**. Average amount won 18,596 euro (approx. 7 months of post-tax income for typical Dutch household). 11.2% of winners also won a BMW, an additional random possibility, worth approx. 25,000 euro.	No	No	6	Happiness, 10 point scale	1	Har.	There were no significant effects of lottery wins in the Dutch Postcode Lottery on happiness	Critical	No
Lachowska 2017	The Effect of Income on Subjective Well-Being Evidence from the 2008 Economic Stimulus Tax Rebates	USA	2008	Repeat cross-sectional	Adults aged 18+ living in United States with gross monthly income <$6000 (n=17,933)	Receiving a tax rebate **(increase)** as a result of the Economic Stimulus Act of 2008, meant to counter impact of Great Recession. Aimed at low and middle-income families, determined on 2007 tax returns. Ranged from $300-$600 for single filers and $600-$1,200 for joint filers. If dependent child, received extra $300 per child.	No	No	0	1. Life satisfaction, 11 point scale (Cantril self-anchoring scale), 2. Subjective affect	12	Ben.	A 2008 tax rebate to counter the effects of the Great Recession in the US was associated with improved life satisfaction	Serious	Yes
Lafave 1995	Partnerships for people with serious mental illness who live below the poverty line	CAN	Not reported: likely 1994	Randomised trial of intervention	People with severe and persistent mental illness who were heavy service users in two counties of Eastern Ontario and lived	Cash transfer of $160 (Canadian) per month for 11 months of study and forgivable loans totalling $1,800 for the study period **(increase)**. Maintained study	Yes	No	11	1. Lehman Quality of Life Interview (QOLI), 2. Annual number of psychiatric hospital inpatient days	2	Har.	A cash transfer for people with serious mental illness to bring them above the poverty line for 11 months was not significantly associated with QoL or inpatient stays	Some concerns	No

					below the poverty line (n=48)	group above poverty line. Control group received $115 for completing 3 interviews and $25-$35 per month in increasing increments for 11 months to maintain contact with project and sponsors. Did not bring this group above poverty line.									
Lam 2000	Correlates of improvement in quality of life among homeless persons with serious mental illness	USA	1994-1997	Basic longitudinal	People who were homeless, suffered from severe mental illness, and were not involved in ongoing community treatment in 15 US cities (n=4,257)	**Change** in total income from baseline to 12 months	No	No	12	QOLI	1	Ben.	A change in income within the last 12 months was significantly associated with subjective QoL in homeless people with severe mental illness	Serious	No
Latif 2015	Happiness Adaptation to Income: Evidence from Canada	CAN	1994-2009	Basic longitudinal	Adults aged 16-65 in ten Canadian provinces (n=5,127)	Within-person **changes** in household income	No	No	12	Happiness, 5 point scale	1	Ben.	Income changes were significantly associated with happiness in Canadian working-age adults	Serious	Yes
Lebihan 2018	The Impact of Universal Child Benefits on Family Health and Behaviours	CAN	1994-2009	Before and after (controlled)	Coupled/partnered mothers of children aged 1-8 years, not living in Quebec (n=5,824)	Introduction an unconditional child care benefit (UCCB) in July 2006: $100/month i.e. $1200 annually for each child under the age of 6 **(increase)**	No	No	0	Maternal depression, original/unvalidated questionnaire	1	Ben.	The introduction of a universal child benefit in Canada was not significantly associated with improved maternal depression	Moderate	Yes
Lindahl 2005	Estimating the Effect of Income on Health and Mortality Using Lottery Prizes as an Exogenous Source of Variation in Income	SWE	1968-1981	Basic longitudinal	Swedish population who have won the lottery (n=626)	Winning at least 1,000 SEK on the lottery between 1969 and 1981 **(increase)**. No indication of when won, so take amount reported in each survey and divide by number of years since last wave	No	No	72	Number of poor mental health related symptoms reported	1	Ben.	The number of mental health symptoms was significantly smaller in people who won larger lottery prizes, compared with those who won smaller prizes	Moderate	No

						to generate 'annual' win amount								
Lindqvist 2018	Long-run Effects of Lottery Wealth on Psychological Well-being	SWE	1994-2016	Basic longitudinal	1. Successful players aged 75 or less from 3 of 4 lotteries held in administrative records: all winners from 2 Triss lotteries [1994-2011] and all large prize winners from Kombi lottery [1998-2011] (those who won at least 1M SEK); 2. 4:1 matched non-winning player controls for large prize Kombi winners (based on sex, age and number of tickets in month of win) (n=3,331)	Amount of lottery prize won at any time between 1994 and 2011 **(increase)**	No	No	264	1. Happiness, 11 point scale, 2. Life satisfaction, 11 point scale, 3. GHQ	15	Ben.	A lottery win was not associated with changes in happiness or likelihood of common mental disorder in a Swedish population of winners compared with non-winners, or those who won less. There was a significant association with life satisfaction which was slightly larger for those who previously had income above the median, and for women	Moderate AND Low	No
Lorant 2007	Depression and socio-economic risk factors: 7-year longitudinal population study	BEL	1992-1999	Basic longitudinal	Belgian adults aged 16+ (n=11,909)	Within-person changes in poverty status, defined as living in a household with income less than half of population median, differentiating between **decreased** and **increased** poverty. Also, within-person changes in household income.	Yes	No	12	Modified version of global depression scale of Health and Daily Living Form (HDL)	8	Ben.	Changes in poverty line status were significantly associated with depression scores in the Belgian population, but not with likelihood of depression. The effects of moving out of poverty were larger than moving into poverty. Changes in income were not significantly associated with either type of outcome	Serious	Yes
Lorenz 2000	After farming: Emotional health trajectories of farm, nonfarm,	USA	1989-1992	Basic longitudinal	Families in north central Iowa with adolescent children living with both biological	**Change** in per capita family income over a three year period, as modelled in latent growth curves	No	No	36	SCL90-R, depression subscale	2	Inc.	Income change over 3 years was not associated with depression in Iowa families	Serious	No

	and displaced farm couples				parents who had siblings within 4 years of their age (n=700)										
Macours 2012	Cash transfers, behavioral changes, and cognitive development in early childhood: evidence from a randomized experiment	NIC	2005-2009	Randomised trial of intervention	Low income mothers in six municipalities in rural Nicaragua affected by drought in previous year (n=1,151)	Basic group received cash transfer **(increase)** every two months, conditional on health check-ups, and additional education transfer for children aged 7-15, conditional on attendance. This was equivalent to 15% of per capita expenditure for average recipient household. A second Training group received Basic plus a scholarship for one member of the household to get vocational training and skills workshops. A third Lump-sum group received Basic plus a one-off payment to start a small nonagricultural activity, conditional on developing business plan - the value was 11% of average household expenditure, so this group got approx. 26% of annual expenditure in total	Yes	Yes	9	CES-D 20	4	Ben.	A small Nicaraguan cash transfer had no significant effect on depression for mothers	Some concerns	Yes
Maeder 2014	Earnings-related parental leave benefits and subjective well-being of young mothers:	DEU	2008-2011	Before and after (controlled)	Women living in Germany in 2008 born in one of three birth cohorts (1991-93, 1981-83, or 1971-73) who	Elterngeld child welfare payment: a new earnings-related benefit, replacing 67% of prior labour earnings to upper bound of 1800	No	No	14	Life satisfaction, 11 point scale	3	Inc.	A change to the German child benefit system to make it universal and earnings linked (rather than means-tested) did not improve life satisfaction for	Serious	Yes

	evidence from a German parental leave reform				gave birth in first or last quarter of 2003/04 or 2006/07 (n=615)	euro/month and lower bound of 300 euro/month. All parents were eligible for up to 12 months after childhood, or 14 months for single parents OR couples where parents share parental leave. Universal and (for majority, though not some low-income parents) more generous **(increase)** than system it replaced, though available for shorter time.							all mothers or when stratifying by partner's education		
Markussen 2019	Economic and Non-economic Returns to Communist Party Membership in Vietnam	VN M	2012-2014	Basic longitudinal	Rural households in 12 provinces in Vietnam (n=1,029)	Within-person **changes** in household income per capita	No	No	24	Life satisfaction, 4 point scale	1	Ben.	Income changes were significantly associated with life satisfaction in Vietnam adults	Moderate	No
McCarthy 2018	Poverty, Material Hardship, and Mental Health among Workers in Three Front-Line Service Occupations	CAN USA	2003-2008	Basic longitudinal	Service workers (barbers/ stylists, food/beverage servers, sex workers) living in Census Metropolitan Area of Victoria, Canada or in three counties of greater metropolitan area of Sacramento, California (n=443)	Within-person **change** in whether someone was 'income poor', defined as household income below one half of median income of city in which they resided, adjusted for household size and membership	Yes	No	12	1. Self-rated mental health, 2. Beck Depression Inventory	2	Ben.	Changes in whether service workers were below an 'income poor' line were not significantly related to self-rated mental health or depression	Serious	Yes
McKenzie 2014	Do changes in socioeconomic factors lead to changes in mental health?	NZL	2004-2009	Basic longitudinal	Residents of New Zealand living in private dwellings aged 15-60 (n=11,445)	Within-person **changes** in household income	No	No	24	1. SF-36 mental health subscale, 2. K10	2	Har.	There was no significant relationship between household income changes and either SF-36 or K10 scores	Serious	Yes

	Findings from three waves of a population based panel study													
Melzer 2017	Migrants' pursuit of happiness: An analysis of the effects of adaptation, social comparison and economic integration on subjective well-being on the basis of German panel data for 1990-2014	DEU	1990-2014	Basic longitudinal	German adults aged 18-59 who have had at least one job (n=38,615)	Within-person **changes** in individual income	No	No	12	Life satisfaction, 11 point scale	2	Ben.	Changes in individual income were significantly associated with life satisfaction in German working age adults	Serious	Yes
Milligan 2008	Do Child Tax Benefits Affect the Wellbeing of Children? Evidence from Canadian Child Benefit Expansions	CAN	1994-2005	Other natural experiment	Canadian women with children <10 years (n=17,287)	**Changes** in amount of child benefit likely received by families across year, province, and number of children due to changes to National Child Benefit Supplement - exploited between-province variation to determine degree of exposure	No	No	120	Maternal depression score adapted from CES-D	2	Ben.	Child benefit amount in Canada was significantly associated with mother's depression score, and effect sizes were larger for mothers with less education	Serious	No
Mohanty 2014	What Determines Happiness? Income or Attitude: Evidence from the U.S. Longitudinal Data	USA	1980-2006	Basic longitudinal	US population aged 14 to 21 in 1979 (n=4,500)	Within-person **changes** in income	No	No	228	Happiness, 4 point scale	2	Ben.	Income changes were significantly associated with life satisfaction	Serious	No
Natali 2018	Does money buy happiness? Evidence from an unconditional	ZMB	2010-2014	Randomised trial of intervention	Women with at least one child < 5 in Kaputa, Kalano	Cash transfer of 120 Zambian Kwacha (roughly $24) on a bi-monthly basis	Yes	No	48	Happiness, binary	2	Ben.	A Zambian unconditional cash transfer scheme increased happiness for women	Some concerns	Yes

	cash transfer in Zambia				or Shangombo districts (n=2,203)	**(increase)**. The transfer represented an increase by 27% to the household's pre-program monthly expenditure and was calculated as an amount sufficient to purchase food equivalent of one meal monthly per day on average for all household members									
North 2008	Family support, family income, and happiness: A 10-year perspective	USA	1981-1991	Basic longitudinal	Unmarried population of San Francisco Bay in 1981 (n=195)	Within-person **changes** in total family income	No	No	72	Happiness, 5 point scale	1	Ben.	Income changes for married San Franciscans from 1981-1991 were not significantly associated with happiness	Serious	No
Ong 2018	The impact of intergenerationa l financial transfers on health and wellbeing outcomes: A longitudinal study	AUS	2001-2015	Basic longitudinal	Australian population aged 15+ not living with parents (n=3,210)	Receiving an intergenerational financial transfer during the last financial year from (i) inheritances or (ii) surviving parents **(increase)**. Mean/median transfer for inheritance = $78,000/$25,000; for parental transfer = $9,700/$2,500.	No	Yes	12	SF-36 mental health subscale	6	Har.	Intergenerational transfers were not associated with improved mental health in Australian adults	Moderate	Yes
Oshio 2012	The association between income dynamics and subjective well-being: Evidence from career income records in Japan	JPN	2011	Basic longitudinal	Japanese men aged 30-59 in 2009 who registered at an internet survey company, held 2009 Social Security Statements, had no interruption in wage history, and were members of an Employees	The amount of income **increase** or **decrease** since the previous year of wage history	No	No	12	1. Life satisfaction, 6 point scale, 2. K6	4	Ben.	Income increases and decreases in Japanese men were mostly not significantly related to life satisfaction or stress except weakly significant results for income increases and K6	Critical	No

					Pension Insurance scheme (n=1,004)									
Ozer 2011	Does alleviating poverty affect mothers' depressive symptoms? A quasi-experimental investigation of Mexico's Oportunidades programme	MEX	2003	Other natural experiment	Women living in poverty in poor rural communities who met all eligibility criteria for Oportunidaes (n=6,343)	Oportunidades conditional cash transfer programme: income supplementation of ~25% of household income, conditional on accessing pre-natal care, nutrition monitoring and supplementation, well infant care and immunisation, preventive check-ups, and participation in educational workshops **(increase)**	Yes	Yes	60	1. CES-D 20, 2. PSS	3	Ben.	A conditional cash transfer scheme for low income women in Mexico led to a significant improvement in depression and stress	Moderate	Yes
Plagerson 2011	Does money matter for mental health? Evidence from the Child Support Grants in Johannesburg, South Africa	ZAF	2007	Basic cross-sectional	Low-income adults aged 18+ living in 5 disadvantaged areas of Johannesburg with children <14 (n=72)	Means-tested cash transfer (Child Support Grant) equivalent to monthly payment of ZAR 210/approx $28 paid to primary caregiver per child **(increase)**	Yes	No	108	20-item Self-Reporting Questionnaire (SRQ)	2	Ben.	Receiving a Child Support Grant in South Africa was associated with improved mental health	Critical	Yes
Powell-Jackson 2016	Cash transfers, maternal depression and emotional well-being: Quasi-experimental evidence from India's Janani Suraksha Yojana programme	IND	2015	Other natural experiment	Women aged 15-49 living in 6 districits of Uttar Pradesh who gave birth in the previous two years in a government facility (n=1,695)	Janani Suraksha Yojana (JSY) payment: a one-off payment of 1400 INR (rural areas) or 1000 INR (urban areas), conditional on giving birth in a health facility **(increase)**	No	Yes	24	1. K10, 2. Happiness, 5 point scale	4	Ben.	A maternity payment given at birth in India was associated with reduction in psychological distress and moderate depression, but not severe depression or happiness	Moderate	Yes
Priebe 2013	Effectiveness of financial incentives to improve adherence to maintenance	GBR	2010-2012	Randomised trial of intervention	Patients aged 18-65 in England and Wales with psychotic disorder who are prescribed long-acting depot	Intervention group offered £15 for each injection of antipsychotic drug over 12 month period	No	Yes	12	1. Clinical Improvement component of Clinical Global Impression scale, 2. Subjective	2	Ben.	Giving £15 incentives for depot injections in mental health patients resulted in significant impacts on quality of life/life satisfaction, but not a	Some concerns AND High	Yes

	treatment with antipsychotics: Cluster randomised controlled trial				injections of antipsychotics and have poor treatment adherence (n=96)	(intervals ranged from 1-4 weeks) **(increase)**				QoL rated on DIALOG scale			clinical improvement in mental health		
Proto 2015	Life satisfaction, income and personality	GBR	1996-2008	Basic longitudinal	UK population aged 18-65 (n=6,843)	Within-person **changes** in household income	No	No	12	Life satisfaction, 7 point scale	1	Har.	There was no significant effect of income changes on life satisfaction in the UK population	Serious	No
Radey 2019	Psychological distress among low-income mothers: the role of public and private safety nets	USA	1999-2005	Basic longitudinal	Female caregivers of children aged 0-4 or 10-14 in 1999, from Black, Hispanic and White families living in high-poverty neighbourhoods in Boston, Chicago and San Antonio, living below 200% of poverty line (n=1,987)	Within-person **change** in the percentage of poverty in which a mother lived	Yes	No	48	BSI	1	Ben.	Changes in poverty status were significantly associated with distress in mothers of children aged 0-4 in low income neighbourhoods in the US	Serious	No
Raschke 2019	Unexpected windfalls, education, and mental health: evidence from lottery winners in Germany	DEU	2000-2011	Other natural experiment	German population who have won the lottery (n=237)	Receiving a large sum of money as lottery winnings in the last year, worth at least €2,500 **(increase)**	No	No	12	SF-12 MCS	3	Har.	Lottery wins were significantly associated with poorer mental health in the following year in German cohort, particularly for those of lower socioeconomic position	Serious	Yes
Reeve 2011	Functional and psychological outcomes following burn injury: reduced income and hidden emotions are predictors of greater distress	NZL	Not reported: likely 2009	Basic cross-sectional	Adults aged 18+ with burn injuries of >10% total body surface area, who were patients at Middlemore Hospital on North Island (n=50)	Experiencing a reduction in income since the time of the burn **(decrease)**	No	No	61.2	HADS	1	Har.	Experiencing an income loss following a considerable burn injury within the last 10 years was significantly associated with anxiety and depression (NOTE: authors report this as a positive association but actually it appears to be a negative association i.e. income loss associated with less anxiety/depression)	Critical	No

Reeves 2016	Reductions in the United Kingdom's Government Housing Benefit and Symptoms of Depression in Low-Income Households	GBR	2009-2013	Before and after (controlled)	UK population aged 16-69 renting housing in the private sector (n=179,037)	Reduction in Housing Benefit: reduced payment from median value of local market rent to 30th percentile **(decrease)**, and capped amount of money households could receive according to number of bedrooms. Average loss of income per claimant £1,220/year.	No	No	0	Self-reported depression, bad nerves or anxiety	1	Ben.	Reductions in housing benefit in 2011 were significantly associated with self-reported depression in UK population of claimants	Serious	Yes
Reeves 2017	Introduction of a National Minimum Wage Reduced Depressive Symptoms in Low-Wage Workers: A Quasi-Natural Experiment in the UK	GBR	1994-2001	Before and after (controlled)	UK population aged 22-59 employed at least 1h/week in 1998 and 1999, earning <£4/hr at baseline (n=172)	Introduction of national minimum wage (NMW) of £3.60 **(increase)**	No	No	12	1. GHQ, 2. Self-reported anxiety/depression	4	Ben.	The introduction of a minimum wage in the UK was associated with improvements in GHQ scores and self-reported anxiety/depression	Serious	Yes
Robert 2014	From the boom to the crisis: changes in employment conditions of immigrants in Spain and their effects on mental health	ESP	2008-2011	Basic longitudinal	Migrant workers from Colombia, Ecuador, Morocco and Romania (n=182)	Monthly income had **increased** or **decreased** compared with baseline three years prior	No	No	36	GHQ	2	Inc.	Income decreases (but not increases) were significantly associated with likelihood of common mental disorder	Critical	Yes
Rogers 2001	Changes in wives' income: Effects on marital happiness, psychological well-being, and the risk of divorce	USA	1980, 1988	Basic longitudinal	Married men and women aged <55 living in the United States (n=1,047)	**Change** in annual income of married women from 1980 to 1988	No	No	96	Individual psychological wellbeing, measured with 21-item scale	2	Ben.	Changes in wives' incomes were significantly associated with psychological wellbeing for the women but not the men in a marriage	Serious	No

Roh 2014	The Effects of Income and Skill Utilization on the Underemployed's Self-Esteem, Mental Health, and Life Satisfaction	KOR	Not reported: likely 2012	Basic longitudinal	Unemployed adults who visited Korean Employment Service Center to receive unemployment insurance benefits, had at least high school diploma and earned more than minimum wage in last job (n=239)	Being under-employed compared with last employment: current earning of at least 20% less than earnings from previous job **(decrease)**	No	No	18	1. GHQ, 2. Life satisfaction- used 4 items from Satisfaction With Life Scale, each with 7-point Likert	2	Har.	After experiencing unemployment, under-employment in the subsequent job (with wages <20% of previous role) was not significantly associated with either GHQ score or life satisfaction	Critical	No
Rosenheck 2000	Outcomes after initial receipt of social security benefits among homeless veterans with mental illness	USA	1992-1999	Basic longitudinal	Veterans enrolled in SSA-VA Joint Outreach Initiative between 1992 and 1999 in Manhattan, Brooklyn, Dallas or LA (n=173)	Being awarded Social Security Disability Insurance (SSDI) and Supplemental Security Income (SSI) benefits after participation in Joint Outreach Initiative, run by Social Security Administration and Veterans Affairs **(increase)**. Mean amount gained $612.73/month.	Yes	No	3	1. Psychiatric status subscale of Addiction Severity Index (ASI), 2. Lehman's summary measure of subjective QoL	2	Ben.	Veterans receiving increased social security payments after contact with an outreach service had a significant improvement in QoL but not psychiatric problems	Critical	Yes
Sareen 2011	Relationship between household income and mental disorders: Findings from a population-based longitudinal study	USA	2001-2005	Basic longitudinal	US population aged 18+ without mental illness at baseline (n=34,895)	Experiencing a **decrease** or **increase** in household income during the three-year period	No	No	36	Axis I and Axis IIDSM-IV mental disorders diagnosed using the Alcohol Use Disorder and Associated Disabilities Interview Schedule-DSM-IV Version: (1) any incident mood disorder (2) any incident anxiety disorder	4	Inc.	Income decreases were associated with 3 year incidence of mood disorder, but no significant results for income increases, or for either on anxiety disorders	Serious	Yes
Schollgen 2019	Income trajectories and subjective well-being: Linking	DEU	1999-2012	Basic longitudinal	German employees born between 1951 and 1980 who were	Mean **change** in individual income from timepoint to timepoint	No	No	156	1. Life satisfaction, German Satisfaction with Life Scale, 2.	4	Ben.	Income changes were significantly associated with life satisfaction and	Serious	No

	administrative records and survey data				subject to social security contributions and consented to have survey data linked with employment histories (n=1,566)	between 1999 and 2011				Scale of Positive and Negative Experiences			affect in German employees		
Schyns 2001	Income and satisfaction in Russia	RUS	1993-1995	Basic longitudinal	Russian population (n not reported)	**Change** in household income between survey waves	No	No	12	Life satisfaction, 10 point scale	2	Ben.	Income changes were significantly associated with life satisfaction in Russian population	Critical	Yes
Sekulova 2013	Climate change, income and happiness: An empirical study for Barcelona	ESP	2011	Basic cross-sectional	Adult residents of Barcelona (n=807)	If income had **decreased** in 2011 compared with recalled income in 2010, 2009 and 2006	No	No	60	Life satisfaction, 10 point scale	3	Inc.	1 year lagged income changes were significantly associated with life satisfaction in Barcelona residents	Critical	Yes
Swift 2020	Association of negative financial shocks during the Great Recession with depressive symptoms and substance use in the USA: the CARDIA study	USA	1990-2010	Basic longitudinal	Black and white adults aged 18-30 years with stable financial wellbeing up to 2005 at four field centres: Universities of Alabama, Minnesota, Northwestern and Kaiser Permanente (n=1,563)	Experiencing a negative income shock i.e. **decrease** in income bracket from pre-recession (1990-2005) to post-recession (2010). Also sensitivity analysis using 'large income drop' as exposure, defined as a shift of two or more income brackets.	No	No	60	CES-D 20	1	Ben.	Income changes were significantly associated with CES-D score in a US cardiac cohort study	Serious	Yes
Tachibana 2019	Do remittances alleviate negative impacts of disaster on mental health? A case of the 2015 Nepal earthquake	NPL	2014-2016	Basic longitudinal	120 sample households who participated in Kumar and Hotchkiss 1988 study on forest depletion and human welfare in 6 villages in Western Nepal. Aimed to sample original respondents OR their children OR someone in HH immediately	Receiving an **increase** in amount of remittance sent to household or a single individual from family abroad, comparing pre- and post-earthquake	No	No	12	1. Checklist Civilian (PCL-C) questionnaire for PTSD, 2. CES-D 20	8	Ben.	Increased remittances sent to Nepalese households after a 2015 earthquake resulted in small significant improvements in PTSD and depression symptoms, but remittances to individuals did not. Results were non-significant for a binary threshold indicating clinical levels of PTSD or depression	Serious	Yes

					adjacent to original sample (n=335)									
Thoits 1979	Income and psychological distress: The impact of an income-maintenance experiment	USA	1970-1974	Randomised trial of intervention	Families with dependent children where household head(s) were aged 18-58 years in low-income areas in Seattle and Denver, only black, white or Chicano ethnicities (n=988)	Being assigned to income-maintenance treatment group - amounts varied, but least generous plan just sufficient to bring families up to official poverty line and slightly exceed support available from existing welfare programmes **(increase)**	Yes	No	24	Variant of Macmillan Health Opinion Survey index	1	Har.	An income maintenance programme found mixed results by ethnicity: no evidence found for improvement in psychological distress, but some evidence of potential worsening of distress in some subgroups	High	No
Thompson 2002	The mental health status of Filipinas in Queensland	AUS	1996-1998	Basic longitudinal	Filipina women aged 16+ living in Queensland who were involved in 24 community organisations and their social contacts, those involved in church groups, and those working in factories (n=337)	Experiencing a major **decrease** in financial situation in last 12 months	No	No	12	GHQ	1	Ben.	Major worsening of financial situation in last 12 months was significantly associated with GHQ in Filipina women in Australia	Serious AND Critical	Yes
Turvey 2002	Financial loss and suicidal ideation in a rural community sample	USA	1998	Basic cross-sectional	Adults aged 18+ living in a single county in Iowa (n=1,621)	If experienced an income **decrease** in the last 12 months	No	No	12	Self-reported suicidal ideation in previous 12 months	1	Ben.	Substantial income decreases in last 12 months were associated with higher prevalence of suicidal ideation	Critical	No
Wickham 2017	The effect of a transition into poverty on child and maternal mental health: a longitudinal analysis of the UK Millennium Cohort Study	GBR	2003-2012	Basic longitudinal	Mothers of singleton children born in the UK between 1st Sep 2000 and 11th Jan 2002, with no mental health problems or poverty when child was aged 3 (n=6,063)	Transition into poverty when the child was aged 5, 7 or 11 years, defined as household income less than 60% of national median **(decrease)**	Yes	No	96	K6	1	Ben.	Moving below the poverty line was significantly associated with mother's depression	Serious	Yes

Wong 2015	Impact of enforcing a statutory minimum wage on work and quality of life of vulnerable groups in Hong Kong	HKG	2010-2012	Basic longitudinal	Adults belonging to vulnerable groups (defined as welfare recipients, new migrant women and low-paid workers) living in Hong Kong (n=253)	Introduction of statutory minimum wage which set hourly wage rate at 28 HKD **(increase)**	No	No	8	WHOQOL-BREF	1	Ben.	There was no significant effect of a minimum wage change in Hong Kong on QoL in vulnerable groups	Critical	No
Young 2005	Economic burdens and quality of life of family caregivers of cancer patients	KOR	Not report ed: likely 2003	Basic cross-sectional	Primary family caregiver aged 18+ for family member with cancer at one of six University Hospitals or the National Cancer Centre in Korea (n=704)	If the impact of the cancer patient's illness (as perceived by caregiver) had included the loss of a major source of family income **(decrease)**	No	No	12	Caregiver QoL Index-cancer (CQOLC)	1	Ben.	Experiencing the loss of an income source after a relative gets cancer was associated with reduced quality of life	Critical	Yes
Zimmerman 2005	Socioeconomic status, depression disparities, and financial strain: what lies behind the income-depression relationship?	USA	1992-1998	Basic longitudinal	US population in 1979 (n=5,604)	Within-person **changes** in household income	No	No	48	CES-D 20	8	Inc.	There was no significant effect of income changes on CES-D score in a US 1979 cohort	Serious	Yes

Table D2: Risk of bias (RoB) domain scores for included non-randomised studies, measured using ROBINS-I

Study	Confounding	Selection	Classification	Deviations	Missingness	Outcome measurement	Reporting	Overall score
**Abbott 2000**	Serious	Critical	Moderate	Low	Low	Low	Low	Critical
**Abbott 2006**	Critical	No information	Moderate	Low	No information	Low	Low	Critical
**Abel 2016**	Critical	Critical	Moderate	Low	No information	Moderate	Low	Critical
**Adams 2019**	Serious	Serious	Serious	Low	No information	Moderate	Moderate	Serious
**Adeola 2009**	Serious	Critical	Serious	Low	Serious	Low	Low	Critical
**AIR 2014**	Low	Low	Low	Low	Low	Low	Low	Low
**Allouche 2019**	Serious	Low	Low	Low	Serious	Low	Low	Serious
**Ambrey 2014**	Moderate	Low	Moderate	Low	Serious	Moderate	Moderate	Serious

**Apouey 2015**	Serious	Serious	Moderate	Low	Serious	Low for GHQ Moderate for life sat.	Low	Serious
**Arata 2000**	Critical	Critical	Serious	Low	No information	Low	Low	Critical
**Araya 2003**	Critical	Serious	Moderate	Low	Low	Low	Low	Critical
**Asadullah 2012**	Critical	Serious	Serious	Low	Low	Moderate	Low	Critical
**Assari 2018**	Serious	Low	Moderate	Low	No information	Low	Low	Serious
**Barbaglia 2015**	Moderate	Low	Moderate	Low	Low	Low	Low	Moderate
**Becchetti 2010**	Critical	Critical	Serious	Low	No information	Serious	Low	Critical
**Benzeval 2001**	Serious	Low	Moderate	Low	Low	Low	Moderate	Serious
**Biotteau 2019**	Serious	Low	Moderate	Low	Low	Low	Low	Serious
**Bland 1996**	Critical	Critical	Serious	Low	Serious	Low	Moderate	Critical
**BlazquezCuesta 2013**	Serious	Moderate	Moderate	Low	Serious	Low	Low	Serious
**Bolden 2010**	Critical	Critical	Moderate	Low	Serious	Low	Low	Critical
**Bonanno 2007**	Serious	Serious	Moderate	Low	Serious	Low	Low	Serious
**Boyce 2009**	Critical	Critical	Serious	Low	No information	Moderate	Low	Critical
**Boyce 2013**	Moderate	Moderate	Moderate	Low	Serious	Low	Moderate	Serious
**Boyce 2018**	Serious	Moderate	Moderate	Low	Serious	Moderate	Low	Serious
**Boyd-Swan 2016**	Serious	Moderate	Low	Low	Moderate	Low for CES-D Moderate for happiness	Low	Serious
**Brenner 2014**	Critical	Critical	Serious	Low	Serious	Low	Low	Critical
**Brickman 1978**	Critical	Critical	Low	Low	Low	Serious	Low	Critical
**Brock 1985**	Critical	Serious	Moderate	Low	Low	Low	Serious	Critical
**Burke 1986**	Critical	Serious	Moderate	Low	Low	Moderate	Low	Critical
**Burmaster 2015**	Serious	Serious	Low	Low	Low	Low	Low	Serious
**Buttke 2012**	Critical	Serious	Moderate	Low	Low	Low for PHQ-2 Moderate for mentally unhealthy days	Low	Critical
**Cai 2016**	Serious	Low	Low	Low	Moderate	Moderate	Low	Serious
**Casey 2004**	Critical	Critical	Moderate	Low	Low	Low	Low	Critical
**Cesarini 2016**	Low	Low	Low	Low	Low	Low	Low	Low
**Cheung 2015**	Moderate	Moderate	Moderate	Low	Low	Moderate	Low	Moderate

**Chien 2004**	Critical	Serious	Moderate	Low	Low	Low	Serious	Critical
**Chin 2018**	Serious	Low	Moderate	Low	Serious	Low	Low	Serious
**Chrostek 2016**	Moderate	No information	Low	Low	Serious	Moderate	Low	Serious
**Clark 2016**	Serious	Moderate	Moderate	Low	No information	Moderate	Low	Serious
**Clingingsmith 2016**	Serious	Serious	Moderate	Low	Serious	Low for K6<br>Moderate for life sat.	Low	Serious
**Coley 2014**	Serious	Low	Moderate	Low	Low	Low	Low	Serious
**Costello 2010**	Moderate	Low	Low	Low	Low	Low	Low	Moderate
**Daley 2017**	Moderate	Moderate	Low	Low	Low	Moderate	Low	Moderate
**Dang 2019**	Serious	Moderate	Low	Low	Serious	Moderate	Low	Serious
**Dearing 2004**	Serious	Moderate	Moderate	Low	Low	Low	Low	Serious
**Diener 1993**	Critical	Serious	Moderate	Low	Serious	Low	Low	Critical
**Dolan 2013**	Serious	Low	Moderate	Low	Serious	Low for Rutter, GHQ, K4<br>Moderate for life sat.	Low	Serious
**Elwell-Sutton 2019**	Serious	Moderate	Moderate	Low	No information	Low	Low	Serious
**Erixson 2017**	Low	Low	Low	Low	Low	Low	Low	Low
**Evans 2005**	Serious	Critical	Moderate	Low	Critical	Moderate	Low	Critical
**Evans 2011**	Serious	Serious	Moderate	Low	No information	Moderate	Low	Serious
**Fang 2017**	Serious	Low	Moderate	Low	Serious	Moderate	Low	Serious
**Feeny 2014**	Critical	Critical	Serious	Low	No information	Moderate	Low	Critical
**Fenn 2015**	Critical	Low	Low	Low	Serious	Low	Low	Critical
**Frijters 2011**	Moderate	Moderate	Moderate	Low	Serious	Moderate	Low	Serious
**Frijters 2012**	Moderate	Moderate	Moderate	Low	Serious	Moderate	Low	Serious
**Galama 2017**	Serious	Moderate	Low	Low	Serious	Moderate	Low	Serious
**Grattan 2017**	Serious	Critical	Serious	Low	Serious	Low	Low	Critical
**Gros 2019**	Serious	Moderate	Low	Serious	Low	Moderate	Low	Serious
**Gulal 2019**	Serious	Moderate	Low	Low	No information	Moderate	Low	Serious
**Handa 2014**	Serious	Moderate	Low	Serious	Low	Moderate	Low	Serious
**Hanly 2015**	Critical	Critical	Moderate	Low	Low	Low	Low	Critical
**Hasan 2016**	Serious	No information	Low	Low	Serious	Serious	Low	Serious
**Hilton 2006**	Critical	Critical	Moderate	Low	Moderate	Low	Low	Critical
**Horn 2017**	Serious	Moderate	Low	Low	Moderate	Moderate	Low	Serious

**Huang 2015**	Critical	Critical	Moderate	Low	Low	Low	Low	Critical
**Ishiguro 2019**	Critical	Critical	Moderate	Low	Serious	Low	Low	Critical
**Jalal 2015**	Serious	Low	Low	Low	No information	Low	Low	Serious
**Junna 2019**	Moderate	Low	Moderate	Low	Low	Low	Low	Moderate
**Kendall 2019**	Moderate	Moderate	Moderate	Low	Serious	Low	Low	Serious
**Kiernan 2018**	Serious	Moderate	Moderate	Low	Moderate	Low for CES-D<br>Moderate for subj. mental health	Low	Serious
**Kikuchi 2013**	Critical	Critical	Moderate	Low	No information	Low	Moderate	Critical
**Klein 2017**	Serious	Moderate	Moderate	Low	Low	Low	Low	Serious
**Koltai 2018**	Moderate	Moderate	Moderate	Low	Moderate	Low	Low	Moderate
**Krauss 2013**	Critical	Critical	Moderate	Low	No information	Moderate	Low	Critical
**Kuhn 2011**	Critical	Critical	Moderate	Low	No information	Moderate	Low	Critical
**Lachowska 2017**	Serious	Moderate	Low	Low	No information	Moderate	Low	Serious
**Lam 2000**	Moderate	Low	Moderate	Low	Serious	Moderate	Low	Serious
**Latif 2015**	Moderate	Moderate	Moderate	Low	Serious	Moderate	Low	Serious
**Lebihan 2018**	Moderate	Low	Low	Low	Low	Low	Low	Moderate
**Lindahl 2005**	Low	Moderate	Moderate	Low	No information	Moderate	Low	Moderate
**Lindqvist 2018**	Low	Low	Low	Low	Low	Low for GHQ<br>Moderate for life sat., happiness	Low	Low for GHQ<br>Moderate for life sat., happiness
**Lorant 2007**	Serious	Low	Moderate	Low	Low	Low	Moderate	Serious
**Lorenz 2000**	Serious	Moderate	Moderate	Low	Serious	Low	Low	Serious
**Maeder 2014**	Moderate	Moderate	Low	Low	Serious	Moderate	Low	Serious
**Markussen 2019**	Moderate	Low	Low	Low	Low	Moderate	Low	Moderate
**McCarthy 2018**	Serious	Serious	Moderate	Low	Serious	Low for Beck<br>Moderate for subj. mental health	Low	Serious
**McKenzie 2014**	Serious	Moderate	Low	Low	Serious	Moderate	Low	Serious
**Melzer 2017**	Moderate	Moderate	Moderate	Low	Serious	Moderate	Low	Serious
**Milligan 2008**	Serious	Moderate	Low	Low	Serious	Moderate	Low	Serious
**Mohanty 2014**	Moderate	Moderate	Moderate	Low	Serious	Moderate	Low	Serious
**North 2008**	Serious	Moderate	Moderate	Low	Low	Moderate	Low	Serious
**Ong 2018**	Moderate	Moderate	Moderate	Low	Moderate	Low	Low	Moderate
**Oshio 2012**	Serious	Critical	Moderate	Low	Low	Low for K6	Low	Critical

						Moderate for life sat.		
**Ozer 2011**	Moderate	Low	Low	Low	Moderate	Low	Low	Moderate
**Plagerson 2011**	Critical	Critical	Moderate	Low	Low	Low	Low	Critical
**Powell-Jackson 2016**	Low	Moderate	Moderate	Low	Low	Low for K10<br>Moderate for happiness	Low	Moderate
**Proto 2015**	Serious	Moderate	Moderate	Low	Serious	Moderate	Low	Serious
**Radey 2019**	Serious	Low	Moderate	Low	Low	Low	Low	Serious
**Raschke 2019**	Serious	Moderate	Moderate	Low	Serious	Low	Low	Serious
**Reeve 2011**	Critical	Critical	Moderate	Low	Low	Low	Low	Critical
**Reeves 2016**	Serious	Serious	Low	Low	No information	Moderate	Low	Serious
**Reeves 2017**	Serious	Serious	Moderate	Low	Low	Low for GHQ<br>Moderate for subj. mental health	Low	Serious
**Robert 2014**	Serious	Serious	Moderate	Low	Critical	Low	Low	Critical
**Rogers 2001**	Serious	Low	Moderate	Low	Serious	Low	Low	Serious
**Roh 2014**	Critical	Moderate	Moderate	Low	Serious	Low	Low	Critical
**Rosenheck 2000**	Critical	Low	Moderate	Low	Serious	Low	Low	Critical
**Sareen 2011**	Serious	Low	Moderate	Low	Low	Low	Low	Serious
**Schollgen 2019**	Serious	Moderate	Moderate	Low	Moderate	Low	Low	Serious
**Schyns 2001**	Critical	Moderate	Moderate	Low	Serious	Moderate	Low	Critical
**Sekulova 2013**	Critical	Critical	Moderate	Low	No information	Low	Low	Critical
**Swift 2020**	Serious	Moderate	Moderate	Low	Serious	Low	Low	Serious
**Tachibana 2019**	Serious	Low	Moderate for remittances to household<br>Serious for remittances to individuals	Low	Moderate	Low	Low	Serious
**Thompson 2002**	Serious for follow-up<br>Critical for baseline	Serious for follow-up<br>Critical for baseline	Serious	Low	Low for follow-up<br>Serious for baseline	Low	Low	Serious for follow-up<br>Critical for baseline
**Turvey 2002**	Critical	Serious	Serious	Low	Low	Serious	Low	Critical

**Wickham 2017**	Moderate	Low	Moderate	Low	Moderate	Low	Low	Serious
**Wong 2015**	Critical	Low	Low	Low	Serious	Low	Low	Critical
**Young 2005**	Critical	Critical	Serious	Low	Low	Low	Low	Critical
**Zimmerman 2005**	Serious	Low	Moderate	Low	Moderate	Moderate	Low	Serious

Table D3: Risk of bias (RoB) domain scores for included randomised controlled trials, measured using ROB-2

Study	Randomisation	Timing	Deviations	Missingness	Measurement	Reporting	Overall score
**Baird 2013**	Low	Low	Low	Low	Some concerns	Some concerns	Some concerns
**Bedoya 2019**	Low	Low	Low	Low	Some concerns	Low	Some concerns
**Courtin 2018**	Low	n/a	Low	Some concerns	Low for K10<br>Some concerns for subj. distress	Some concerns	Some concerns
**Dorsett 2014**	Low	n/a	Some concerns	Low	Some concerns	Some concerns	Some concerns
**Fernald 2011**	Low	Low	Low	Low	Low	Some concerns	Some concerns
**Gassman-Pines 2006**	Some concerns	n/a	Low	Low	Low	Some concerns	Some concerns
**Gennetian 2002**	Some concerns	n/a	Low	Low	Low	Some concerns	Some concerns
**Green 2016**	Low	Low	Low	Low	Low	Low	Low
**Haushofer 2016**	Low	Low	Some concerns	Low	Low for CES-D, Cohens<br>Some concerns for life sat., happiness	Low	Some concerns
**Hjelm 2017 (CGP)**	Low	Low	Low	Low	Low	Some concerns	Some concerns
**Kilburn 2016**	Low	Low	Low	Low	Low	Low	Low
**Kilburn 2018**	Low	Low	Low	Low	Low	Some concerns	Some concerns
**Lafave 1995**	Some concerns	n/a	Some concerns	Low	Low	Some concerns	Some concerns
**Macours 2012**	Low	Low	Low	Low	Low	Some concerns	Some concerns
**Natali 2018**	Low	Low	Low	Low	Some concerns	Some concerns	Some concerns
**Priebe 2013**	Low	Low	Low	Some concerns for clinical improvement<br>High for QoL	Low	Low	Some concerns for clinical improvement<br>High for QoL
**Thoits 1979**	Some concerns	n/a	Low	Some concerns	Low	Some concerns	High

Appendix E: List of studies excluded at full text

#	Author/Year	Title	Where published	Why excluded
1	Abdoulayi 2015	Malawi Social Cash Transfer Programme: Midline Impact Evaluation Report.	Chapel Hill: Carolina Population Center	Duplicate sample
2	Acierno 2019	Mental Health Correlates of Financial Mistreatment in the National Elder Mistreatment Study Wave II	Journal of Aging and Health	Incorrect population
3	Adams 2006	A systematic review of the health, social and financial impacts of welfare rights advice delivered in healthcare settings	BMC Public Health	Systematic review
4	Adato 2010	Conditional Cash Transfers in Latin America	Johns Hopkins University Press	Incorrect study type
5	Adena 2013	Poverty and transitions in key areas of quality of life	De Gruyter: Active Ageing and Solidarity between Generations in Europe	Incorrect study type
6	Adoho 2014	The impact of an adolescent girls employment program: the EPAG project in Liberia	The World Bank, Policy Research Working Paper Series: 6832	No comparator group
7	Agyapong 2017	Microfinance, Rural Non-farm Activities and Welfare Linkages in Ghana: Assessing Beneficiaries' Perspectives	Global Social Welfare	No relevant outcome(s)
8	Ahmat 2019	Effects of minimum wage policy implementation: Compensation, work behaviors, and quality of life	International Journal of Hospitality Management	Incorrect exposure
9	Ahmed 2019	Human wellbeing outcomes of involvement in industrial crop production: Evidence from sugarcane, oil palm and jatropha sites in Ghana	PLoS ONE	Incorrect exposure
10	Ahn 2010	La felicidad de los espanoles: Factores explicativos. (Some Determinants of Happiness among the Spanish People)	Revista de Economia Aplicada	Incorrect language
11	Ahnquist 2007	Is cumulative exposure to economic hardships more hazardous to women's health than men's? A 16-year follow-up study of the Swedish Survey of Living Conditions	Journal of epidemiology and community health	Incorrect exposure
12	Ahuvia 2008	If money doesn't make us happy, why do we act as if it does?	Journal of Economic Psychology	Incorrect study type
13	AIR 2016	Zambia's Multiple Category Targeting Grant: 36-Month Impact Report.	Washington, DC: American Institutes for Research	Duplicate sample
14	Akande 2003	South African women and unemployment: A social-economic perspective	Psikhologicheskii Zhurnal	Unable to source full text
15	Akay 2016	Remittances and relative concerns in rural China	China Economic Review	Incorrect exposure
16	Akay 2019	Relative concerns and sleep behavior	Economics and Human Biology	Incorrect exposure
17	Aker 2015	Comparing cash and voucher transfers in a humanitarian context: evidence from the Democratic Republic of Congo	World Bank Economic Review	Exact duplicate
18	Aker 2017	Comparing Cash and Voucher Transfers in a Humanitarian Context: Evidence from the Democratic Republic of Congo	World Bank Economic Review	No relevant outcome(s)
19	Aknin 2018	Buying well-being: Spending behavior and happiness	Social and Personality Psychology Compass	Incorrect study type
20	Alcazar 2016	Impacts of the Peruvian Conditional Cash Transfer Program on Women's Empowerment: A Quantitative and Qualitative Approach	PEP-PMMA, Working Papers	No relevant outcome(s)
21	Alderson 2008	Effects of Employment-Based Programs on Families by Prior Levels of Disadvantage	Social Service Review	No comparator group

22	Alem 2015	Consumption Smoothing and the Welfare Cost of Uncertainty	Centre for Economic Performance, LSE, CEP Discussion Papers	Incorrect exposure
23	Ali 1997	Employment transitions and psychological distress: the contrasting experiences of single and married mothers	Journal of Health & Social Behavior	No comparator group
24	Ali 2010	Recovery From Depression Among Clients Transitioning Out of Poverty	American Journal of Orthopsychiatry	Incorrect exposure
25	Ali 2015	Empirical Assessment of the Impact of Microfinance on Quality of Life	Pakistan Business Review	No relevant outcome(s)
26	Allanson 2008	Longitudinal analysis of income-related health inequality	University of Dundee, Economic Studies, Discussion Papers: 214	Incorrect unit of analysis
27	Amell 2007	An examination of mental health and mental health trajectories among African American and white men: The effects of poverty and perceived social supports on psychological distress, self-esteem, and life satisfaction	University of Wisconsin-Madison	Incorrect exposure
28	Amroussia 2017	Explaining mental health inequalities in Northern Sweden: a decomposition analysis	Global health action	Incorrect exposure
29	Anand 2018	Depression and economic status: evidence for non-linear patterns in women from Mexico	Journal of Mental Health	Incorrect exposure
30	Angeles 2011	A Closer Look at the Easterlin Paradox	Journal of Socio-Economics	Incorrect exposure
31	Angeles 2016	The Social Cash Transfer Programme of Malawi: The Role of Evaluation from the Pilot to the Expansion	From Evidence to Action: The Story of Cash Transfers and Impact Evaluation in Sub-Saharan Africa	Incorrect study type
32	Antonides 2007	Income Evaluation and Happiness in the Case of an Income Decline	Kyklos	No comparator group
33	Apouey 2009	Winning Big but Feeling no Better? The Effect of Lottery Prizes on Physical and Mental Health	Fondazione Eni Enrico Mattei, Working Papers: 2009.96	Duplicate analysis
34	Apouey 2010	Winning Big but Feeling No Better? The Effect of Lottery Prizes on Physical and Mental Health	Institute of Labor Economics (IZA) discussion paper	Duplicate analysis
35	Apouey 2013	Winning Big But Feeling No Better? The Effect of Lottery Prizes on Physical and Mental Health	Paris School of Economics - HAL discussion paper	Duplicate analysis
36	Apouey 2014	Winning big but feeling no better? The effect of lottery prizes on physical and mental health	Centre for Economic Performance discussion paper	Duplicate analysis
37	Argyle 1997	L'effet des variables environnementales sur le bonheur = The effect of environmental variables on happiness	Revue Quebecoise de Psychologie	Unable to source full text
38	Ariyabuddhi-phongs 2011	Lottery gambling: A review	Journal of Gambling Studies	Incorrect study type
39	Armstrong 2018	Assessing the financial impact of pulmonary hypertension: a survey by the UK Pulmonary Hypertension Association (PHA-UK)	European Respiratory Journal.	Incorrect study type
40	Arnould 2009	Does Fair Trade Deliver on Its Core Value Proposition? Effects on Income, Educational Attainment, and Health in Three Countries	Journal of Public Policy & Marketing	Incorrect exposure
41	Arriagada 2015	Do Payments Pay Off? Evidence from Participation in Costa Rica's PES Program	PLoS ONE	Incorrect unit of analysis
42	Arthaud-Day 2005	The wealth of nations and the happiness of nations: Why "accounting" matters	Social Indicators Research	Incorrect study type
43	Asadullah 2018	Subjective Well-Being in China, 2005-2010: The Role of Relative Income, Gender, and Location	China Economic Review	Incorrect exposure

44	Asoogo 2018	Experiences of patients diagnosed and living with metastatic breastcancer in Kumasi, Ghana: A lesion to learn from low-middle income country	Journal of Global Oncology	Incorrect study type
45	Attah 2016	Can Social Protection Affect Psychosocial Wellbeing and Why Does This Matter? Lessons from Cash Transfers in Sub-Saharan Africa	Journal of Development Studies	Incorrect study type
46	Awaworyi-Churchill 2019	Religiosity, Income and Wellbeing in Developing Countries	Empirical Economics	Incorrect exposure
47	Ayala 2013	Health-related effects of welfare-to-work policies	Social Science & Medicine	Incorrect exposure
48	Babic 2015	RE-INITIATING PROFESSIONAL WORKING ACTIVITY AFTER MYOCARDIAL INFARCTION IN PRIMARY PERCUTANEOUS CORONARY INTERVENTION NETWORKS ERA	International journal of occupational medicine and environmental health	No comparator group
49	Baird 2011	Income shocks and adolescent mental health	The World Bank, Policy Research Working Paper Series: 5644	Duplicate analysis
50	Baird 2013	Girl Power: Cash Transfers and Adolescent Welfare. Evidence from a Cluster-Randomized Experiment in Malawi	National Bureau of Economic Research, Inc, NBER Working Papers: 19479	No relevant outcome(s)
51	Baird 2015	What Happens Once the Intervention Ends? the Medium-Term Impacts of a Cash Transfer Programme in Malawi, 3IE Grantee Final Report	New Delhi: International Initiative for Impact Evaluation	Duplicate sample
52	Balbinotto 2016	The impact of socioeconomic factors in the prevalence of depression in Brazil between 1998 and 2008	Value in Health	Incorrect study type
53	Ball 2008	Absolute income, relative income, and happiness	Social Indicators Research	Incorrect exposure
54	Barrett 2018	The Economics of Poverty Traps	National Bureau of Economic Research Conference Report series	Incorrect study type
55	Barrientos 2012	Poverty Transitions among Older Households in Brazil and South Africa	European Journal of Development Research	Incorrect population
56	Barrington-Leigh 2018	Informing policy priorities using inference from life satisfaction responses in a large community survey	Applied Research in Quality of Life	Exact duplicate
57	Barrington-Leigh 2019	Informing Policy Priorities using Inference from Life Satisfaction Responses in a Large Community Survey	Applied Research in Quality of Life	Incorrect exposure
58	Bartolini 2010	Predicting the Trend of Well-Being in Germany: How Much Do Comparisons, Adaptation and Sociability Matter?	CEPS Instead Working Paper 2010-07	Duplicate analysis
59	Bartolini 2011	Predicting the Trend of Well-Being in Germany: How Much Do Comparisons, Adaptation and Sociability Matter?	DIW Berlin, The German Socio-Economic Panel working paper	Duplicate analysis
60	Bartolini 2013	Predicting the Trend of Well-Being in Germany: How Much Do Comparisons, Adaptation and Sociability Matter?	Social Indicators Research	Duplicate sample
61	Bartolini 2013	Did the Decline in Social Connections Depress Americans' Happiness?	Social Indicators Research	Incorrect exposure
62	Bartram 2011	Economic Migration and Happiness: Comparing Immigrants' and Natives' Happiness Gains From Income	Social Indicators Research	Incorrect exposure
63	Bartram 2013	Happiness and 'economic migration': A comparison of Eastern European migrants and stayers	Migration Studies	Incorrect exposure
64	Bartram 2015	Inverting the Logic of Economic Migration: Happiness Among Migrants Moving from Wealthier to Poorer Countries in Europe	Journal of Happiness Studies	Incorrect exposure

65	Bayer 2015	Happiness and the Persistence of Income Shocks	American Economic Journal-Macroeconomics	Duplicate sample
66	Becchetti 2008	Does money affect happiness and self-esteem? The poor borrowers' perspective in a quasi-natural experiment	Associazione Italiana per la Cultura della Cooperazione e del Non Profit	Duplicate analysis
67	Becchetti 2008	Easterlin-types and Frustrated Achievers: the Heterogeneous Effects of Income Changes on Life Satisfaction	Faculty of Economics, University of Cambridge, Cambridge Working Papers in Economics	Duplicate analysis
68	Becchetti 2009	When money does not buy happiness: The case of 'frustrated achievers'	The Journal of Socio-Economics	No relevant outcome(s)
69	Becchetti 2010	The Money-Happiness Relationship in Transition Countries: Evidence from Albania	Transition Studies Review	No comparator group
70	Becchetti 2011	The Heterogeneous Effects of Income Changes on Happiness	Social Indicators Research	Duplicate sample
71	Becchetti 2011	Income, Relational Goods and Happiness	Applied Economics	Incorrect exposure
72	Becchetti 2013	Credit access and life satisfaction: evaluating the nonmonetary effects of micro finance	Applied Economics	Incorrect exposure
73	Beggs 2002	Income, subjective well-being and the comparative perspective: An examination of relative income and its possible comparison standards	Saint Louis University	Unable to source full text
74	Behrman 2010	The Economics of Conditional Cash Transfers	Conditional Cash Transfers in Latin America	Incorrect study type
75	Behrman 2011	The Impact of the PROGRESA/Oportunidades Conditional Cash Transfer Program on Health and Related Outcomes for the Aging in Mexico	Penn Institute for Economic Research, Department of Economics, University of Pennsylvania, PIER Working Paper Archive	No relevant outcome(s)
76	Berger 2013	Happy Working Mothers? Investigating the Effect of Maternal Employment on Life Satisfaction	Economica	Duplicate sample
77	Berzins 2018	Relationship of employment status and socio-economic factors with distress levels and counselling outcomes during a recession	Counselling & Psychotherapy Research	Incorrect exposure
78	Beser 2005	Quality of Life in Lymphoma Patients	Clinical Excellence for Nurse Practitioners	Incorrect exposure
79	Bhanot 2018	Workfare, wellbeing and consumption: Evidence from a field experiment with Kenya's urban poor	Journal of Economic Behavior & Organization	Incorrect exposure
80	Bhuiyan 2019	Micro-entrepreneurship and subjective well-being: Evidence from rural Bangladesh	Journal of Business Venturing	Incorrect exposure
81	Bijlsma 2017	Unemployment and subsequent depression: A mediation analysis using the parametric G-formula	Social Science & Medicine	No comparator group
82	Binder 2010	An examination of the dynamics of well-being and life events using vector autoregressions	Journal of Economic Behavior & Organization	Duplicate sample
83	Binder 2013	The Structure of Subjective Well-being: A Vector Autoregressive Approach	Metroeconomica	Duplicate sample
84	Blazquez-Cuesta 2014	Deprivation and Subjective Well-Being: Evidence from Panel Data	Review of Income and Wealth	Duplicate sample
85	Boen 2016	The physiological impacts of wealth shocks in late life: Evidence from the Great Recession	Social Science & Medicine	Incorrect population
86	Boes 2004	Income and Happiness: New Results from Generalized Threshold and Sequential Models	Institute of Labor Economics (IZA)	Incorrect exposure
87	Boes 2010	The Effect of Income on General Life Satisfaction and Dissatisfaction	Social Indicators Research	Duplicate sample

88	Bonsang 2006	The relative impact of income and health on the subjective well-being across generations in Europe	Centre de Recherche en Economie Publique et de la Population (CREPP), University of Liège	Incorrect exposure
89	Borgen 1996	Psychological well-being throughout the transition from adolescence to adulthood	Career Development Quarterly	Incorrect exposure
90	Boyce 2011	Personality and the marginal utility of income: Personality interacts with increases in household income to determine life satisfaction	Journal of Economic Behavior & Organization	Duplicate sample
91	Breslin 2003	Factors influencing the impact of unemployment on mental health among young and older adults in a longitudinal, population-based survey	Scandinavian Journal of Work, Environment & Health	No comparator group
92	Brewer 2014	Partnership dissolution: how does it affect income, employment and well-being?	Institute for Social and Economic Research	No comparator group
93	Brockmann 2012	Das Glück der Migranten: eine Lebenslaufanalyse zum subjektiven Wohlbefinden von Migranten der ersten Generation in Deutschland	DIW Berlin, The German Socio-Economic Panel (SOEP)	Incorrect language
94	Brodeur 2012	Do Higher Childcare Subsidies Improve Parental Well-being? Evidence from QuÃ©bec's Family Policies	Paris School of Economics - HAL discussion paper	Incorrect exposure
95	Brodeur 2013	Do higher child care subsidies improve parental well-being? Evidence from Quebec's family policies	Journal of Economic Behavior & Organization	Incorrect exposure
96	Brown 2020	Consumption Changes, Not Income Changes, Predict Changes in Subjective Well-Being	Social Psychological and Personality Science	Duplicate sample
97	Bruggink 2016	Changes between pre-crisis and crisis period in socioeconomic inequalities in health and stimulant use in Netherlands	European Journal of Public Health	Incorrect exposure
98	Bruggink 2016	Changes between pre-crisis and crisis period in socioeconomic inequalities in health and stimulant use in Netherlands. [Portuguese]	Nederlands Tijdschrift voor Geneeskunde	Incorrect language
99	Brzezinski 2018	Diagnosing unhappiness dynamics: Evidence from poland and russia	Journal of Happiness Studies: An Interdisciplinary Forum on Subjective Well-Being	Exact duplicate
100	Brzezinski 2019	Diagnosing Unhappiness Dynamics: Evidence from Poland and Russia	Journal of Happiness Studies	Incorrect unit of analysis
101	Buchanan 2009	Gimme money, that's what I want	New Scientist	Exact duplicate
102	Buller 2016	The way to a man's heart is through his stomach?: a mixed methods study on causal mechanisms through which cash and in-kind food transfers decreased intimate partner violence	BMC Public Health	Incorrect exposure
103	Buttke 2012	Mental health needs assessment after the gulf coast oil Spilla-Alabama and Mississippi, 2010	Prehospital and Disaster Medicine	No comparator group
104	Buttke 2012	Mental health needs assessment after the Gulf Coast oil spill-Alabama and Mississippi, 2010	Prehospital & Disaster Medicine	Exact duplicate
105	Calvo 2018	Does Money Buy Immigrant Happiness?	Journal of Happiness Studies	Duplicate sample
106	Campbell 2007	Exploring the relationships between provision of welfare benefits advice and the health of elderly people: a longitudinal observational study and discussion of methodological issues	Health & Social Care in the Community	Incorrect population
107	Caner 2015	Happiness, Comparison Effects, and Expectations in Turkey	Journal of Happiness Studies	Incorrect exposure
108	Cao 2014	Household income and subjective well-being after spinal cord injury: A longitudinal study	Topics in Spinal Cord Injury Rehabilitation	Incorrect exposure

109	Cariappa 2019	Universal Basic Income for India: The Way towards Right to Equality--A Review	Indian Journal of Economics and Development	Incorrect study type
110	Carroll 2005	Unemployment and Psychological Well-Being	Centre for Economic Policy Research, Research School of Social Sciences, Australian National University, Discussion Papers: 492	No comparator group
111	Case 2001	Health, Income and Economic Development	Princeton University, Woodrow Wilson School of Public and International Affairs, Research Program in Development Studies., Working Papers: 207	Incorrect exposure
112	Case 2002	Health, income, and economic development	Annual World Bank Conference on Development Economics 2001/2002	Incorrect study type
113	Chan 2011	The Impact of Microloans in Vulnerable Remote Areas: Evidence from Malaysia	Asia Pacific Business Review	No relevant outcome(s)
114	Chang 2013	Climbing up the social ladders: Identity, relative income, and subjective well-being	Social Indicators Research	Incorrect exposure
115	Cheung 2017	Income redistribution and life satisfaction	Michigan State University	Incorrect unit of analysis
116	Cheung 2018	Income redistribution predicts greater life satisfaction across individual, national, and cultural characteristics	Journal of Personality & Social Psychology	Incorrect unit of analysis
117	Chhagan 2008	The socioeconomic impact of antiretroviral treatment on individuals in Soweto, South Africa	Health Sociology Review	No relevant outcome(s)
118	Chin 2010	Income, health, and well-being in rural Malawi	Demographic Research	Incorrect exposure
119	Chin 2012	Essays on health, mortality, and intergenerational transfers in rural Malawi	University of Pennsylvania	Unable to source full text
120	Christian 2019	Income Shocks and Suicides: Causal Evidence From Indonesia	The Review of Economics and Statistics	Incorrect unit of analysis
121	Chu 2014	Winding road toward the Chinese dream: The U-shaped relationship between income and life satisfaction among Chinese migrant workers	Social Indicators Research	Incorrect exposure
122	Clark 2005	Heterogeneity in reported well-being: Evidence from twelve European countries	Economic Journal	Incorrect exposure
123	Cockerham 1990	A test of the relationship between race, socioeconomic status, and psychological distress	Social Science & Medicine	Incorrect exposure
124	Coley 2007	Maternal functioning, time, and money: The world of work and welfare	Children and Youth Services Review	No comparator group
125	Coley 2014	Low-income women's employment experiences and their financial, personal, and family well-being: JFP JFP	Journal of Family Psychology	Exact duplicate
126	Commander 2008	North Birmingham assertive outreach evaluation of service users' quality of life	Journal of Mental Health	No comparator group
127	Coppola 2015	Smoking, Drinking, Never Thinking of Tomorrow: Income and Risky Choices amongst Young Adults in the UK	Institute of Labor Economics (IZA)	No relevant outcome(s)
128	Craig 2009	Household income, economic pressure, and depressive mood among unmarried women in midlife: The moderating effects of locus of control, financial instrumental support received from parents, and race	University of North Carolina	Incorrect exposure
129	Crowe 2016	Financial hardship, mastery and social support: Explaining poor mental health amongst the inadequately employed using data from the HILDA survey	SSM - Population Health	Incorrect exposure

130	Cutler 2014	When Does Education Matter? The Protective Effect of Education for Cohorts Graduating in Bad Times	National Bureau of Economic Research, Inc, NBER Working Papers: 20156	Incorrect exposure
131	Cutler 2015	When does education matter? The protective effect of education for cohorts graduating in bad times	Social Science & Medicine	Incorrect exposure
132	D'Ambrosio 2009	Satisfaction with Life and Economic Well-Being: Evidence from Germany	Schmollers Jahrbuch: Zeitschrift fur Wirtschafts- und Sozialwissenschaften/Journal of Applied Social Science Studies	Incorrect exposure
133	D'Ambrosio 2019	Money and happiness: Income, wealth and subjective well-being	Social Indicators Research	Duplicate sample
134	DeGarmo 1999	Social class as a moderator of income effects on stress and health outcomes across nine years	Annals of the New York Academy of Sciences	Incorrect unit of analysis
135	deGraaf 2013	First-incidence of DSM-IV mood, anxiety and substance use disorders and its determinants: results from the Netherlands Mental Health Survey and Incidence Study-2	Journal of Affective Disorders	Duplicate sample
136	deRuffi 2018	Effects of financial precariousness on mental health	Psychiatria Danubina	Incorrect exposure
137	Diener 1995	Factors predicting the subjective well-being of nations	Journal of personality and social psychology	Incorrect exposure
138	Diener 2002	Will money increase subjective well-being? A literature review and guide to needed research	Social Indicators Research	Incorrect study type
139	Diener 2002	Will money increase subjective well-being?	Social Indicators Research	Exact duplicate
140	Diener 2009	Will Money Increase Subjective Well-Being?: A Literature Review and Guide to Needed Research	Science of Well-Being: The Collected Works of Ed Diener	Incorrect study type
141	Diener 2009	Income's Differential Influence on Judgments of Life Versus Affective Well-Being	Assessing Well-Being: The Collected Works of Ed Diener	Incorrect exposure
142	Diener 2013	Rising income and the subjective well-being of nations	Journal of Personality & Social Psychology	Incorrect exposure
143	Dijkstra-Kersten 2015	Associations of financial strain and income with depressive and anxiety disorders	Journal of Epidemiology & Community Health	Incorrect exposure
144	DiTella 2007	Happiness Adaptation to Income and to Status in an Individual Panel	National Bureau of Economic Research, Inc, NBER Working Papers: 13159	Duplicate analysis
145	DiTella 2008	Gross national happiness as an answer to the Easterlin Paradox?	Journal of Development Economics	Incorrect exposure
146	DiTella 2010	Happiness Adaptation to Income and to Status in an Individual Panel	Journal of Economic Behavior and Organization	Duplicate sample
147	Dolan 2007	'Good luck to them if they can get it': exploring working class men's understandings and experiences of income inequality and material standards	Sociology of Health & Illness	Incorrect study type
148	Dooley 2000	Underemployment and depression: longitudinal relationships	Journal of Health & Social Behavior	Duplicate sample
149	Duncan 1997	Income Effects across the Life Span: Integration and Interpretation	Consequences of growing up poor	Incorrect study type
150	Easterlin 1995	WILL RAISING THE INCOMES OF ALL INCREASE THE HAPPINESS OF ALL	Journal of Economic Behavior & Organization	Incorrect study type
151	Easterlin 2005	Diminishing marginal utility of income? Caveat emptor	Social Indicators Research	Incorrect unit of analysis
152	Easterlin 2005	Diminishing Marginal Utility of Income? A Caveat	Social Indicators Research	Exact duplicate

153	Elmes 2019	Health impacts of a WISE: a longitudinal study	Social Enterprise Journal	No comparator group
154	Emanuel 2000	What do you advice doc? A citizens advice bureau in primary care in the West Midlands	Manchester, Centre for Higher and Adult Education, Faculty of Education, University of Manchester; 2000	Unable to source full text
155	Eren 2017	The Determinants of Happiness in Turkey: Evidence from City-Level Data	Journal of Happiness Studies	Incorrect exposure
156	Eren 2017	The determinants of happiness in Turkey: Evidence from city-level data	Journal of Happiness Studies: An Interdisciplinary Forum on Subjective Well-Being	Exact duplicate
157	Ettner 1996	New evidence on the relationship between income and health	Journal of Health Economics	Incorrect exposure
158	Fang 2015	Do Losses Bite More than Gains? Evidence from a Panel Quantile Regression Analysis of Subjective Well-being in Japan	Nanyang Technological University, School of Humanities and Social Sciences, Economic Growth Centre, Economic Growth Centre Working Paper Series: 1507	Duplicate analysis
159	Feather 1997	Economic deprivation and the psychological impact of unemployment	Australian Psychologist	Incorrect study type
160	Fernald 2009	Poverty-alleviation program participation and salivary cortisol in very low-income children	Social Science and Medicine	No relevant outcome(s)
161	Fernandes 2018	A Randomized Controlled Trial of Financial Incentives for Medicaid Beneficiaries with Diabetes	Permanente Journal	No relevant outcome(s)
162	Flavin 2017	The State of the Minimum Wage: Federalism, Economic Policy, and Workers' Well-Being	Forum-a Journal of Applied Research in Contemporary Politics	Incorrect unit of analysis
163	Forget 2011	The Town with No Poverty: The Health Effects of a Canadian Guaranteed Annual Income Field Experiment	Canadian Public Policy-Analyse de Politiques	No comparator group
164	Frescoln 2018	Work Requirements and Well-Being in Public Housing	Cityscape	No comparator group
165	Frese 1987	Alleviating depression in the unemployed: adequate financial support, hope and early retirement	Social Science & Medicine	Incorrect exposure
166	Frey 1999	Measuring Preferences by Subjective Well-Being	Journal of Institutional and Theoretical Economics (JITE)	Incorrect exposure
167	Frijters 2001	The Value of Reunification in Germany: An Analysis of Changes in Life Satisfaction	The University of Melbourne	Duplicate analysis
168	Frijters 2002	The Value of Reunification in Germany: An Analysis of Changes in Life Satisfaction	Institute of Labor Economics (IZA)	Duplicate analysis
169	Frijters 2003	The Value of Reunification in Germany: An Analysis of Changes in Life Satisfaction	Royal Economics Society Working Paper	Duplicate analysis
170	Frijters 2003	Estimating The Causal Effect of Income on Health: Evidence from Post Reunification East Germany	Centre for Economic Policy Research, Research School of Social Sciences, Australian National University, Discussion Papers: 465	No relevant outcome(s)
171	Frijters 2004a	The value of reunification in Germany; An analysis of changes in life satisfaction?	School of Economics and Finance, Queensland University of Technology	Duplicate analysis
172	Frijters 2004b	Income and Life Satisfaction in Post-transition Russia: A New Empirical Methodology for Panel Data	School of Economics and Finance, Queensland	Duplicate analysis

			University of Technology, School of Economics and Finance Discussion Papers and Working Papers Series: 186c	
173	Frijters 2004c	Investigating the patterns and determinants of life satisfaction in Germany following reunification	Journal of Human Resources	Duplicate sample
174	Frijters 2004d	Money does matter! Evidence from increasing real income and life satisfaction in East Germany following reunification	American Economic Review	Duplicate sample
175	Frijters 2005	The causal effect of income on health: Evidence from German reunification	Journal of Health Economics	No relevant outcome(s)
176	Frijters 2005	The Causal Effect of the Income on Health: Evidence from German Reunification	School of Economics and Finance, Queensland University of Technology, School of Economics and Finance Discussion Papers and Working Papers Series: 205b	No relevant outcome(s)
177	Frijters 2006	Can the large swings in russian life satisfaction be explained by ups and downs in real incomes ?	Scandinavian Journal of Economics	Duplicate sample
178	Gaarder 2010	Conditional cash transfers and health: unpacking the causal chain	Journal of Development Effectiveness	Systematic review
179	Gardner 2006	Money and Mental Wellbeing : A Longitudinal Study of Medium-Sized Lottery Wins	University of Warwick, Department of Economics, The Warwick Economics Research Paper Series (TWERPS)	Duplicate analysis
180	Gardner 2007	Money and mental wellbeing: a longitudinal study of medium-sized lottery wins	Journal of Health Economics	Duplicate sample
181	Gariepy 2017	Early-life family income and subjective well-being in adolescents	PLoS ONE [Electronic Resource]	Incorrect population
182	Gartaula 2012	Socio-Cultural Dispositions and Wellbeing of the Women Left Behind: A Case of Migrant Households in Nepal	Social Indicators Research	Incorrect study type
183	Gassman-Pines 2013	How Social Safety Net Programs Affect Family Economic Well-Being, Family Functioning, and Children's Development	Child Development Perspectives	Incorrect study type
184	Gatina 2016	Does money buy happiness? Financial and general well-being of immigrants in Australia	Journal of Behavioral and Experimental Economics (formerly The Journal of Socio-Economics)	Duplicate sample
185	Gerber 2013	Remittances in the Republic of Georgia: Correlates, Economic Impact, and Social Capital Formation	Demography	No relevant outcome(s)
186	Gibson 2017	Welfare-to-work interventions and their effects on the mental and physical health of lone parents and their children	Cochrane Database of Systematic Reviews	Systematic review
187	Gibson 2018	Welfare-to-work interventions and their effects on the mental and physical health of lone parents and their children	Cochrane Database of Systematic Reviews	Exact duplicate
188	Gillen 2017	Do Financial Constraints Affect Depressive Symptomatology Among Mid-Aged and Older Adults?	International Journal of Aging & Human Development	Incorrect population
189	Glenn 1998	Effect of a transient, geographically localised economic recovery on community health and income studied with longitudinal household cohort interview method	Journal of Epidemiology and Community Health	No comparator group
190	Gomez 2013	Social determinants of health outcomes in older Mexican American cancer survivors	Augusta University	Unable to source full text

191	Gorgievski-Duijvesteijn 2005	Finances and well-being: a dynamic equilibrium model of resources	Journal of Occupational Health Psychology	Incorrect exposure
192	Gottlieb 2011	Depressive Symptoms and Their Social Contexts: a Qualitative Systematic Literature Review of Contextual Interventions	International Journal of Social Psychiatry	Systematic review
193	Gould 2003	Essays on Health, Work, Poverty, and Income Inequality	University of Wisconsin	Unable to source full text
194	Gregg 2007	Welfare Reform and Lone Parents in the UK	Department of Economics, University of Bristol, UK, The Centre for Market and Public Organisation	Duplicate sample
195	Greyling 2019	Access to micro- and informal loans: Evaluating the impact on the quality of life of poor females in South Africa	South African Journal of Economic and Management Sciences	Incorrect exposure
196	Groot 2000	Life-satisfaction and preference drift	Social Indicators Research	Incorrect exposure
197	Grun 2010	Is Any Job Better than No Job? Life Satisfaction and Re-employment	Journal of Labor Research	Incorrect exposure
198	Gudmundsdottir 2013	The Impact of Economic Crisis on Happiness	Social Indicators Research	Incorrect exposure
199	Gumber 2017	Effects of Out-of-Pocket (OOP) payments and financial distress on Quality of Life (QoL) of People with Parkinson's (PwP) and their Carers	Health and Quality of Life Outcomes. Conference: Patient Reported Outcome Measure's, PROMs Conference: Advances in Patient Reported Outcomes Research. United Kingdom	Incorrect study type
200	Gupta 2009	Depressive Symptoms and Economic Outcomes of Low-Income Women: A Review of the Social Causation, Social Selection, and Interactionist Hypotheses	Social Issues and Policy Review	Incorrect study type
201	Hagen-Zanker 2018	What are the effects of cash transfers for refugees in the context of protracted displacement? Findings from Jordan	International Social Security Review	Incorrect study type
202	Halvorsen 2018	Self-employment in later life: Implications for financial, physical, and mental well-being	Washington University, St Louis	Incorrect population
203	Hansson 2008	Impact of changes in life circumstances on subjective well-being in an adult population over a 3-year period	Public Health	Incorrect exposure
204	Hashmi 2020	Socioeconomic inequalities in mental health in Australia: Explaining life shock exposure	Health Policy	Incorrect exposure
205	Haushofer 2013	Household Response to Income Changes: Evidence from an Unconditional Cash Transfer Program in Kenya.	Princeton University	Duplicate sample
206	Hazell 2019	Financial Toxicity in Lung Cancer: An Assessment of Magnitude, Perception, and Impact on Quality of Life	International Journal of Radiation Oncology Biology Physics	Incorrect study type
207	Hazell 2020	Financial toxicity in lung cancer: an assessment of magnitude, perception, and impact on quality of life	Annals of Oncology	Incorrect exposure
208	Headey 2004	The effects of wealth and income on subjective well-being and ill-being	Economic Record	Incorrect exposure
209	Headey 2004	Money Doesn't Buy Happiness â€¦ or Does It? A Reconsideration Based on the Combined Effects of Wealth, Income and Consumption	Melbourne Institute of Applied Economic and Social Research, The University of Melbourne	No relevant outcome(s)
210	Headey 2005	Money and Happiness: The Combined Effects of Wealth, Income and Consumption	Schmollers Jahrbuch: Zeitschrift fur Wirtschafts-	Incorrect exposure

			und Sozialwissenschaften/Journal of Applied Social Science Studies	
211	Headey 2005	The Importance of Wealth for Subjective Well-Being	Journal of Financial Transformation	Incorrect exposure
212	Headey 2008	Money does not buy happiness: Or does it? A reassessment based on the combined effects of wealth, income and consumption	Social Indicators Research	No relevant outcome(s)
213	Hellevik 2003	Economy, values and happiness in Norway	Journal of Happiness Studies: An Interdisciplinary Forum on Subjective Well-Being	Incorrect exposure
214	Hentschel 2017	The Influence of Major Life Events and Personality Traits on the Stability of Affective Well-Being	Journal of Happiness Studies	Duplicate sample
215	Hill 1999	Changes in roles following divorce: Comparison of factors contributing to depression in custodial single mothers and custodial single fathers	Journal of Divorce & Remarriage	No comparator group
216	Hlouskova 2019	The Consumption-Investment Decision of a Prospect Theory Household: A Two-Period Model with an Endogenous Second Period Reference Level	Journal of Mathematical Economics	Incorrect study type
217	Horiuchi 2006	The quality of life among persons with severe mental illness enrolled in an assertive community treatment program in Japan: 1-year follow-up and analyses	Clinical Practice and Epidemiology in Mental Health	No comparator group
218	Hsiao 2015	The reductions in monetary cost and gains in productivity with methadone maintenance treatment: one year follow-up	Psychiatry Research	No comparator group
219	Huber 2015	Financial incentives reduce smoking and improve wellbeing in pregnant women	Psychoneuroendocrinology	Incorrect study type
220	Hunn 2011	Path analysis of welfare use: Depression as a mediating factor	Journal of Family and Economic Issues	Incorrect exposure
221	Hutchinson 2007	Evaluation of a combined supported computer education and employment training program for persons with psychiatric disabilities	Psychiatric Rehabilitation Journal	No comparator group
222	Ifcher 2011	The Happiness of Single Mothers after Welfare Reform	B E Journal of Economic Analysis & Policy	Incorrect exposure
223	Jalal 2009	Effects of BRAC's poverty reduction program targeting the ultra-poor in rural Bangladesh	Cornell University	Incorrect exposure
224	Johnston 1984	Effects of labelling on income, work and social function among hypertensive employees	Journal of Chronic Diseases	No relevant outcome(s)
225	Johnston 1984	EFFECTS OF LABELING ON INCOME, WORK AND SOCIAL FUNCTION AMONG HYPERTENSIVE EMPLOYEES	Journal of Chronic Diseases	Exact duplicate
226	Jones 2008	Health, Income and Relative Deprivation: Evidence from the BHPS	Journal of Health Economics	Duplicate sample
227	Juarez 2019	Effects of non-health-targeted policies on migrant health: a systematic review and meta-analysis	The Lancet Global Health	Systematic review
228	Kafle 2017	Impact of Coordinated Asset Transfers and Asset Ownership on Poverty Reduction, Women's Empowerment, and Child Education: Evidence from Zambia, and Tanzania	University of Illinois	No relevant outcome(s)
229	Kaplan 2008	Socioeconomic determinants of psychological well-being: the role of income, income change, and income sources during the course of 29 years	Annals of Epidemiology	No relevant outcome(s)

230	Kapteyn 1985	The Impact of Changes in Income and Family Composition on Subjective Measures of Well-Being	Horizontal Equity, Uncertainty, and Economic Well-Being	Incorrect study type
231	Karanikolos 2016	Effects of the Global Financial Crisis on Health in High-Income Oecd Countries: A Narrative Review	International Journal of Health Services	Systematic review
232	Kaur 2017	The role of socio-economic factors on the onset of comorbidity in primary care consulters with osteoarthritis: A prospective cohort study	Rheumatology (United Kingdom)	Incorrect study type
233	Kelly 2009	Financial implications for patients and their families having treatment for head and neck cancer : Another aspect of quality of life	Supportive Care in Cancer	Incorrect study type
234	Kenny 2009	THERE'S MORE TO LIFE THAN MONEY: EXPLORING THE LEVELS/GROWTH PARADOX IN INCOME AND HEALTH	Journal of International Development	No relevant outcome(s)
235	Keuschnigg 2012	Rich and satisfied? Theoretical considerations and empirical results on the association between wealth and life satisfaction	Berliner Journal Fur Soziologie	Incorrect language
236	Kikuchi 2011	Impact and characteristics of quality of life in Japanese patients with multiple sclerosis	Quality of Life Research	Duplicate sample
237	Kikuchi 2011	Structural equation modelling of contributing factors to quality of life in Japanese patients with multiple sclerosis	Multiple Sclerosis	Incorrect study type
238	Kikuchi 2013	Structural equation modeling of factors contributing to quality of life in Japanese patients with multiple sclerosis	BMC Neurology	Exact duplicate
239	Kilburn 2016	Effects of a Large-Scale Unconditional Cash Transfer Program on Mental Health Outcomes of Young People in Kenya	Journal of Adolescent Health	Duplicate analysis
240	Kim 2003	Depressive symptoms across adolescence and young adulthood in men: Predictions from parental and contextual risk factors	Development and Psychopathology	Incorrect exposure
241	Kim 2007	Socioeconomic status and age trajectories of health	Social Science & Medicine	Incorrect exposure
242	Kim 2016	The effect of childhood and current economic status on depressive symptoms in South Korean individuals: a longitudinal study	International Journal for Equity in Health	Incorrect exposure
243	Kincaid 1995	MARITAL SEPARATION - CAUSES, COPING, AND CONSEQUENCES	Journal of Divorce & Remarriage	No comparator group
244	King 2004	Moving to Opportunity and Tranquility: Neighborhood Effects on Adult Economic Self-Sufficiency and Health from a Randomized Housing Voucher Experiment	Harvard University, John F. Kennedy School of Government, Working Paper Series	Incorrect exposure
245	Klaas 2011	Change in socio-economic status related to psychosocial factors among youth with spinal cord injury	Topics in Spinal Cord Injury Rehabilitation	Incorrect study type
246	Knab 2008	The Effects of Welfare and Child Support Policies on Maternal Health and Well-Being	Making Americans Healthier: Social and Economic Policy as Health Policy	Incorrect exposure
247	Knabe 2011	Quantifying the psychological costs of unemployment: the role of permanent income	Applied Economics	Duplicate sample
248	Koltai 2018	Socioeconomic status, stress exposure, and psychological well-being: Complexities in the stress process	University of Toronto	Duplicate analysis
249	Kongsakon 2009	Quality of life among the navies at six months after the tsunami in phang-nga naval base, phangnga province, Thailand	European Psychiatry	Incorrect study type
250	Kongsakon 2012	Quality of life among the navies and their spouses survivors, six months after the tsunami disaster in Phang-Nga Naval Base, Thailand	Journal of the Medical Association of Thailand	Unable to source full text

251	Kopasker 2018	Economic insecurity: A socioeconomic determinant of mental health	Ssm-Population Health	Duplicate sample
252	Kotsis 2018	Sustained income reductions during economic recession predict depression onset in people with chronic illnesses: A 6-month prospective study	Journal of Psychosomatic Research	Incorrect study type
253	Kramer 2018	Relative Earnings and Depressive Symptoms among Working Parents: Gender Differences in the Effect of Relative Income on Depressive Symptoms	Sex Roles	Incorrect exposure
254	Kronenberg 2017	The impact of the UK National Minimum Wage on mental health	SSM - Population Health	Duplicate sample
255	Kuroki 2018	Subjective well-being and minimum wages: Evidence from U.S. states	Health Economics	Incorrect exposure
256	Kuruvilla 2007	Poverty, social stress & mental health	Indian Journal of Medical Research	Incorrect study type
257	Lachowska 2015	The Effect of Income on Subjective Well-Being: Evidence from the 2008 Economic Stimulus Tax Rebates	W.E. Upjohn Institute for Employment Research, Upjohn Working Papers and Journal Articles: 15-238	Duplicate analysis
258	Lakshmanasamy 2010	Are You Satisfied with Your Income? The Economics of Happiness in India	Journal of Quantitative Economics, New Series	Unable to source full text
259	Layard 2007	The Marginal Utility of Income	Centre for Economic Performance, LSE, CEP Discussion Papers	Incorrect exposure
260	Layard 2008	The marginal utility of income	Journal of Public Economics	Incorrect exposure
261	Le 2012	Analysis of medical expenditure and socio-economic status in patients with ocular chemical burns in East China: a retrospective study	BMC Public Health	No comparator group
262	Lee 2011	Happiness, Adaptation, and Decreasing Marginal Utility of Income	Journal of Private Enterprise	Incorrect study type
263	Leigh 2019	Minimum wages and public health: A literature review	Preventive Medicine	Systematic review
264	Leszko 2019	The Effectiveness of Psychoeducational and Financial Intervention to Support Caregivers of Individuals With Alzheimer's Disease in Poland	Innovation in Aging	Incorrect population
265	Li 2016	Why Economic Growth Did Not Translate into Increased Happiness: Preliminary Results of a Multilevel Modeling of Happiness in China	Social Indicators Research	Incorrect exposure
266	Liker 1982	Wage and status effects of employment on affective well-being among ex-felons	American Sociological Review	No comparator group
267	Littrell 2011	The influence of orphan care and other household shocks on health status over time: a longitudinal study of children's caregivers in rural Malawi	Aids Care-Psychological and Socio-Medical Aspects of Aids/Hiv	Incorrect exposure
268	Liu 2012	Individual Well-Being in Urban China: The Role of Income Expectations	China Economic Review	Incorrect exposure
269	Ludbrook 2004	Do Interventions to Increase Income Improve the Health of the Poor in Developed Economies and Are Such Policies Cost Effective?	Applied Health Economics and Health Policy	Systematic review
270	Luhmann 2011	Stability and variability in the relationship between subjective well-being and income	Journal of Research in Personality	No comparator group
271	Lund 2011	Poverty and mental disorders: Breaking the cycle in low-income and middle-income countries	The Lancet	Systematic review

272	Lund 2011	Global Mental Health 1 Poverty and mental disorders: breaking the cycle in low-income and middle-income countries	Lancet	Exact duplicate
273	Luo 2017	Income growth and happiness growth in China	Economic and Political Studies-Eps	Incorrect exposure
274	Máté 2007	Anyagi helyzet és boldogság: Kapcsolat individuális és nemzetközi szinten = Happiness and financial status: Connection on individual and national level	Mentálhigiéné és Pszichoszomatika	Incorrect language
275	Macours 2008	Cash transfers, behavioral changes, and cognitive development in early childhood: evidence from a randomized experiment	The World Bank Policy Research Working Paper	Duplicate analysis
276	Madden 2009	Mental stress in Ireland, 1994-2000: a stochastic dominance approach	Health Economics	Incorrect exposure
277	Madden 2011	The Impact of an Economic Boom on the Level and Distribution of Subjective Well-Being: Ireland, 1994-2001	Journal of Happiness Studies	No relevant outcome(s)
278	Madhani 2015	Participation in Micro-Finance Programmes and Women's Mental Health in South Asia: A Modified Systematic Review	Journal of Development Studies	Systematic review
279	Mahadea 2008	Economic Growth, Income and Happiness: An Exploratory Study	South African Journal of Economics	Incorrect exposure
280	Mahadea 2013	ON THE ECONOMICS OF HAPPINESS: THE INFLUENCE OF INCOME AND NON-INCOME FACTORS ON HAPPINESS	South African Journal of Economic and Management Sciences	Incorrect exposure
281	Mahadea 2014	On the Economics of Happiness: The Influence of Income and Non-income Factors on Happiness	South African Journal of Economic and Management Sciences, N.S.	Exact duplicate
282	Mahmutefendic 2013	Ekonomija srece. (Economics of Happiness. With English summary.)	Socioeconomica	Incorrect language
283	Mammen 2009	Life Satisfaction among Rural Low-income Mothers: The Influence of Health, Human, Personal, and Social Capital	University of Massachusetts Amherst, Department of Resource Economics, Working Papers: 2009-2	Incorrect exposure
284	Marinescu 2018	No Strings Attached: The Behavioral Effects of U.S. Unconditional Cash Transfer Programs	National Bureau of Economic Research, Inc, NBER Working Papers: 24337	Incorrect study type
285	Marks 1999	Influences and consequences of well-being among Australian young people: 1980-1995	Social Indicators Research	No comparator group
286	Markussen 2018	The happy farmer: Self-employment and subjective well-being in rural Vietnam	Journal of Happiness Studies: An Interdisciplinary Forum on Subjective Well-Being	Incorrect exposure
287	Martikainen 2003	Effects of income and wealth on GHQ depression and poor self rated health in white collar women and men in the Whitehall II study	Journal of epidemiology and community health	Incorrect exposure
288	Martinez 2018	The Impacts of Cash Transfers on Subjective Wellbeing and Poverty: The Case of Colombia	Journal of Family and Economic Issues	No relevant outcome(s)
289	McAllister 2018	How do macro-level structural determinants affect inequalities in mental health? - a systematic review of the literature	International Journal for Equity in Health	Systematic review
290	McBride 2007	Money, Happiness, and Aspirations: An Experimental Study	University of California-Irvine, Department of Economics, Working Papers: 060721	Incorrect exposure
291	McBride 2010	Money, happiness, and aspirations: An experimental study	Journal of Economic Behavior & Organization	Incorrect exposure

292	McCloud 2019	Financial stress and mental health among higher education students in the UK up to 2018: rapid review of evidence	Journal of epidemiology and community health	Systematic review
293	McCormick 2016	High Financial Strain, but Not Poverty or Lower Education, Increases the Risk of Incident Depression in Systemic Lupus Erythematosus (SLE)	Arthritis & Rheumatology	Incorrect study type
294	McGarrigle 2012	The impact of being the intermediate caring generation on self-reported health of older women in Ireland: Analysis of the irish longitudinal study of ageing (TILDA 2010)	Irish Journal of Medical Science	Incorrect study type
295	McGarrigle 2013	The impact of being the intermediate caring generation on self-reported health of older women in Ireland. Analysis of the IRISH longitudinal study on ageing (TILDA 2010)	Age and Ageing	Incorrect study type
296	McInerney 2013	Recession depression: Physical and mental health effects of the 2008 stock market crash	Journal of Mental Health Policy and Economics	Incorrect study type
297	McInerney 2013	Recession depression: mental health effects of the 2008 stock market crash	Journal of Health Economics	Incorrect population
298	Mehlis 2018	Financial toxicity in patients with colorectal and neuroendocrine tumors: Impact of a chronic disease on patients' economic situation	Oncology Research and Treatment	Incorrect study type
299	Melzer 2012	Migrant's Pursuit of Happiness: The Impact of Adaption, Social Comparison and Relative Deprivation; Evidence from a 'Natural' Experiment	DIW Berlin, The German Socio-Economic Panel (SOEP)	Duplicate analysis
300	Mendolia 2011	The impact of husband's job loss on partners' mental health	University Library of Munich	Duplicate analysis
301	Mendolia 2014	The impact of husband's job loss on partners' mental health	Review of Economics of the Household	Duplicate sample
302	Mentzakis 2009	The poor, the rich and the happy: Exploring the link between income and subjective well-being	The Journal of Socio-Economics	Duplicate sample
303	Meraya 2018	Heterogeneous Relationships between Labor Income and Health by Race/Ethnicity	Health Services Research	Duplicate sample
304	Mervin 2014	Is shared misery double misery?	Social Science and Medicine	Duplicate sample
305	Mesch 1999	The effects of hospital closure on mental health workers: An overview of employment, mental and physical health, and attitudinal outcomes	Journal of Behavioral Health Services & Research	No comparator group
306	Meyer 2008	Changes in the Consumption, Income, and Well-Being of Single Mother Headed Families	American Economic Review	No relevant outcome(s)
307	Michaud 2008	Health and wealth of elderly couples: Causality tests using dynamic panel data models	Journal of Health Economics	Incorrect population
308	Minkin 2017	Income and Wellbeing in a Society on the Verge to Market Integration: The Case of the Tsimane' in the Bolivian Amazon	Journal of Happiness Studies	Incorrect exposure
309	Miret 2016	Effect of economic crises on suicide rates	Understanding suicide: From diagnosis to personalized treatment.	Incorrect study type
310	Missen 2012	The financial and psychological impacts on mothers of children with chronic fatigue syndrome (CFS/ME): [1]	Child Care, Health and Development	No comparator group
311	Missen 2012	The financial and psychological impacts on mothers of children with chronic fatigue syndrome (CFS/ME)	Child: Care, Health & Development	Exact duplicate
312	Mitrut 2011	Do private and public transfers received affect life satisfaction? Evidence from Romania	Journal of Economic Psychology	Incorrect exposure

313	Mohamadzadeh 2013	The Effect of Income on Labor Force Happiness in Iran. (In Farsi. With English summary.)	Tahghighat-e-Eghtesadi	Incorrect language
314	Mohindra 2008	On poverty and health: An interventionist perspective; a study of women microcredit groups in Kerala, India	Universite de Montreal	Incorrect exposure
315	Mok 2018	Family income inequalities and trajectories through childhood and self-harm and violence in young adults: a population-based, nested case-control study	The Lancet Public Health	Incorrect exposure
316	Moorhead 2003	Child and adolescent predictors for eating disorders in a community population of young adult women	International Journal of Eating Disorders	Incorrect exposure
317	Moren-Cross 2004	The effects of life course poverty on depression	Gerontologist	Incorrect study type
318	Morente 2016	DETERMINANTS AND IMPACT OF ECONOMIC CRISIS ON THE MENTAL HEALTH OF POPULATION OF SPAIN	Revista Rol De Enfermeria	Unable to source full text
319	Morris 2010	Conditional Cash Transfer Programs and Health	Conditional Cash Transfers in Latin America	Incorrect study type
320	Mossakowski 2008	Dissecting the Influence of Race, Ethnicity, and Socioeconomic Status on Mental Health in Young Adulthood	Research on Aging	Incorrect exposure
321	Mota-Pereira 2012	Unemployment and income lowering increase risk of depression relapse	European Psychiatry. Conference: 20th European Congress of Psychiatry, EPA	Incorrect study type
322	MotaPereira 2015	Financial crisis increases the risk of depression relapse	Journal of Psychiatric Research	Incorrect exposure
323	Muller 2003	Money or time? Comparing the effects of time structure and financial deprivation on the psychological distress of unemployed adults	Australian Journal of Psychology	Incorrect exposure
324	Muskin 2000	Earnings changes	The American journal of psychiatry	Incorrect study type
325	Nam 2016	Financial Difficulty Effects on Depressive Symptoms Among Dementia Patient Caregivers	Community Mental Health Journal	Incorrect exposure
326	Nars 2007	Modern science of happiness and role of money	Ekonomiska Samfundets Tidskrift	Incorrect study type
327	Nars 2007	Den moderna lyckoforskningen och pengarnas roll. (Modern Happiness Science and Money. With English summary.)	Ekonomiska Samfundets Tidskrift	Exact duplicate
328	Navarro 2018	INCOME AND SUBJECTIVE WELL-BEING: THE SOCIAL COMPARISONS EFFECT	Revista de Economia Mundial	Incorrect language
329	Navarro 2018	Ingreso y bienestar subjetivo: El efecto de las comparaciones sociales. (Income and Subjective Well-Being: The Social Comparisons Effect. With English summary.)	Revista de Economia Mundial	Exact duplicate
330	Nelson 2015	Prevalence and predictors of anxiety and depression among allogeneic hematopoietic cell transplant patients	Psycho-Oncology	Incorrect study type
331	Ngo 2016	Community engagement compared with technical assistance to disseminate depression care among low-income, minority women: A randomized controlled effectiveness study	American Journal of Public Health	Incorrect exposure
332	Nikolova 2015	In transit: The well-being of migrants from transition and post-transition countries	Journal of Economic Behavior & Organization	No comparator group
333	Nikolova 2019	Switching to self-employment can be good for your health	Journal of Business Venturing	Duplicate sample
334	Nissle 2002	Winning the jackpot and depression: Money cannot buy happiness	International Journal of Psychiatry in Clinical Practice	Incorrect study type

335	Noble 2012	"The day the welfare rights lassie rang, I felt this whole weight had been lifted" how welfare rights advice can help with the psychological and social impacts of cancer	Psycho-Oncology	Incorrect study type
336	Noel-Miller 2010	DEPRESSION AMONG ADULT-CHILD CAREGIVERS: THE EFFECT OF CARE-RECEIVING PARENT'S FINANCIAL TRANSFER	Gerontologist	Incorrect study type
337	O'Brien 2011	Poverty, Inequality and Subjective Quality of Life in Rural Russia during the Transition to a Market Economy: 1991-2006	Poverty and Public Policy	Incorrect exposure
338	O'Campo 1998	Welfare reform and women's health: review of the literature and implications for state policy	Journal of Public Health Policy	Incorrect study type
339	O'Connor 2017	Who Suffered Most from the Great Recession? Happiness in the United States	Rsf-the Russell Sage Journal of the Social Sciences	Incorrect exposure
340	Okada 1997	Health and earned income: Recursive and non-recursive models	Columbia University	Unable to source full text
341	Ong 2019	Reducing debt improves psychological functioning and changes decision-making in the poor	Proceedings of the National Academy of Sciences of the United States of America	Incorrect exposure
342	Oshio 2013	The association of life satisfaction and self-rated health with income dynamics among male employees in Japan	Japan and the World Economy	Duplicate analysis
343	Owusu-Addo 2018	The impact of cash transfers on social determinants of health and health inequalities in sub-Saharan Africa: a systematic review	Health Policy and Planning	Systematic review
344	Ozdamar 2017	The Causal Effects of Income Support and Housing Benefits on Mental Well-Being: An Application of a Bayesian Network	Metroeconomica	Duplicate sample
345	Parker 2016	What Effect Did the Global Financial Crisis Have Upon Youth Wellbeing? Evidence From Four Australian Cohorts	Developmental Psychology	Incorrect exposure
346	Paul 2013	Income-happiness paradox in Australia: Testing the theories of adaptation and social comparison	Economic Modelling	Duplicate sample
347	Paxson 2007	Does money matter? The effects of cash transfers on child health and development in rural Ecuador.	The World Bank Policy Research Working Paper Series: Policy Research Working Paper; No. 4226	Duplicate sample
348	Paxson 2010	Does money matter? The effects of cash transfers on child development in rural Ecuador	Economic Development & Cultural Change	Duplicate sample
349	Payne 2019	Private Intergenerational Transfers, Family Structure, and Health in a subâ€Saharan African Context	Population and Development Review	No relevant outcome(s)
350	Pedersen 2009	Happiness in Europe: Cross-Country Differences in the Determinants of Subjective Well-Being	Institute of Labor Economics (IZA)	No relevant outcome(s)
351	Pedersen 2011	Happiness in Europe: Cross-country differences in the determinants of satisfaction with main activity	The Journal of Socio-Economics	No relevant outcome(s)
352	Pedersen 2011	Happiness in Europe	Journal of Socio-Economics	No relevant outcome(s)
353	Pega 2013	In-work tax credits for families and their impact on health status in adults	Cochrane Database of Systematic Reviews	Systematic review
354	Pega 2015	Unconditional cash transfers for assistance in humanitarian disasters: Effect on use of health services and health outcomes in low- and middle-income countries	Cochrane Database of Systematic Reviews	Systematic review
355	Pega 2017	Unconditional cash transfers for reducing poverty and vulnerabilities: effect on use of health services and health outcomes in low- and middle-income countries	Cochrane Database of Systematic Reviews	Systematic review
356	Perova 2010	Three Essays on Intended and Not Intended Impacts of Conditional Cash Transfers	University of California, Berkeley	No relevant outcome(s)

357	Pettifor 2016	The impact of a cash transfer on young South African women's on mental health: HPTN 068	Journal of the International AIDS Society	Incorrect study type
358	Pettifor 2016	The impact of a cash transfer on young South African women's on mental health: HPTN 068	Journal of the International AIDS Society	Exact duplicate
359	Pollmann-Schult 2014	Parenthood and Life Satisfaction: Why Don't Children Make People Happy?	Journal of Marriage and Family	Duplicate sample
360	Pool 2015	NEGATIVE WEALTH SHOCK AND THE ASSOCIATIONS WITH DEPRESSION AND MEDICATION NON-ADHERENCE: SUPPORT FOR A LIFE COURSE SENSITIVE PERIOD	Gerontologist	Incorrect study type
361	Pool 2017	Negative wealth shock and short-term changes in depressive symptoms and medication adherence among late middle-aged adults	Journal of Epidemiology & Community Health	Incorrect exposure
362	Poresky 2001	Two-year comparison of income, education, and depression among parents participating in regular Head Start or supplementary Family Service Center Services	Psychological Reports	No comparator group
363	Pouwels 2008	Income, Working Hours, and Happiness	Economics Letters	Incorrect exposure
364	Powdthavee 2010	How much does money really matter? Estimating the causal effects of income on happiness	Empirical Economics	Duplicate sample
365	Powdthavee 2014	Economic Approaches to Understanding Change in Happiness	Institute of Labor Economics (IZA)	Incorrect study type
366	Prause 2009	Income Volatility and Psychological Depression	American Journal of Community Psychology	Duplicate sample
367	Proto 2012	Life Satisfaction, Household Income and Personality Traits	University of Warwick, Department of Economics, The Warwick Economics Research Paper Series (TWERPS)	Duplicate analysis
368	Pymont 2017	Within-person analysis of welfare transitions in a longitudinal panel survey reveals change in mental health service use	Journal of Public Health	No comparator group
369	Quadir 2019	How are the surviving workers of the Rana Plaza factory collapse in Bangladesh doing: quality of life, participation restriction, income and occupation	Disability and rehabilitation	No comparator group
370	Raphael 2005	Researching income and income distribution as determinants of health in Canada: gaps between theoretical knowledge, research practice, and policy implementation	Health Policy	Incorrect study type
371	Raschke 2013	Essays on the Impact of Income on Family and Child Well-Being	Louisiana State University	No relevant outcome(s)
372	Rebeira 2017	Essays on the economics of longevity	University of Toronto	Incorrect exposure
373	Reich 2005	Living wage policies at the San Francisco Airport: Impacts on workers and businesses	Industrial Relations	No relevant outcome(s)
374	RobertRoca 2013	Changes in employment conditions and mental health during the economic crisis in migrant workers in Spain	Occupational and Environmental Medicine. Conference: 23rd Conference on Epidemiology in Occupational Health, EPICOH	Incorrect study type
375	Rodgers 2001	Income, health, and the National Lottery: BMJ BMJ	British Medical Journal	Incorrect study type
376	Rohde 2014	The Effect of Economic Insecurity on Mental Health: Recent Evidence from Australian Panel Data	Griffith University, Department of Accounting, Finance and Economics	Duplicate analysis

377	Rohde 2016	The effect of economic insecurity on mental health: Recent evidence from Australian panel data	Social Science & Medicine	Duplicate sample
378	Rokhim 2016	Does microcredit improve wellbeing? Evidence from Indonesia	Humanomics	No relevant outcome(s)
379	Roll 2010	Intergenerational financial transfers and mental health: an analysis using SHARE-Israel data	Aging & Mental Health	Incorrect exposure
380	Rotermann 2007	Marital breakdown and subsequent depression	Health Reports	Incorrect exposure
381	Sabarwal 2010	How do women weather economic shocks ? a review of the evidence	The World Bank, Policy Research Working Paper Series: 5496	Incorrect study type
382	Santalahti 2012	Children of the recession study I: Are there long-term effects of economic recession on offspring mental health and integration to society?	Neuropsychiatrie de l'Enfance et de l'Adolescence	Incorrect study type
383	Sato 2017	The Impact of Job Loss on Health. (In Japanese. With English summary.)	Keizai Bunseki	Incorrect language
384	Schlegel 2012	Predictors of depressive symptoms among breast cancer patients during the first year post diagnosis	Psychology & Health	Incorrect exposure
385	Schneider 2009	Impact of supported employment on service costs and income of people with mental health needs	Journal of Mental Health	No relevant outcome(s)
386	Schnittker 2008	Diagnosing our national disease: Trends in income and happiness, 1973 to 2004	Social Psychology Quarterly	Incorrect exposure
387	Schofield 2018	Family Health and Income: A Two-Sample Replication	Journal of Family Psychology	Exact duplicate
388	Schurer 2016	HAPPINESS, INCOME AND HETEROGENEITY	Singapore Economic Review	Duplicate sample
389	Schwandt 2018	Wealth Shocks and Health Outcomes: Evidence from Stock Market Fluctuations	American Economic Journal-Applied Economics	Incorrect population
390	Schyns 1999	Income, Change in Income and Life-Satisfaction: A Study over Time in West Germany and the Russian Federation	Vierteljahrshefte zur Wirtschaftsforschung / Quarterly Journal of Economic Research	Duplicate sample
391	Schyns 2000	The relationship between income, changes in income and life-satisfaction in West Germany and the Russian Federation: relative, absolute, or a combination of both?	Advances in Quality of Life Theory and Research	Incorrect study type
392	Scoppa 2008	An empirical study of happiness in Italy	B E Journal of Economic Analysis & Policy	Incorrect exposure
393	Sherman 2016	Impact of socioeconomic disparities on the 12-item short-form health survey (SF-12) quality of life instrument among osteoarthritis patients	Value in Health	Incorrect study type
394	Silverstein 2006	Intergenerational transfers and living arrangements of older people in rural China: consequences for psychological well-being	Journals of Gerontology Series B-Psychological Sciences & Social Sciences	Incorrect population
395	Sipe 2015	Effects of mental health benefits legislation: A community guide systematic review	American Journal of Preventive Medicine	Systematic review
396	Soeda 2011	Organ donation in Japan: A longitudinal study of quality of life of living liver donors	Transplant International	Incorrect study type
397	Song 2009	Psychological health in residents participating in clean-up works of Hebei spirit oil spill. [Korean]	Journal of Preventive Medicine and Public Health	Incorrect language
398	Song 2009	[Psychological health in residents participating in clean-up works of Hebei Spirit oil spill]	Journal of Preventive Medicine & Public Health / Yebang Uihakhoe Chi	Exact duplicate
399	Song 2016	HEALTH CONSEQUENCES OF RURAL-TO-URBAN MIGRATION: EVIDENCE FROM PANEL DATA IN CHINA	Health Economics	No relevant outcome(s)

400	Soto 2013	Who Can Buy Happiness? Personality Traits Moderate the Effects of Stable Income Differences and Income Fluctuations on Life Satisfaction	Social Psychological and Personality Science	Duplicate sample
401	Stillman 2012	Miserable Migrants? Natural Experiment Evidence on International Migration and Objective and Subjective Well-Being	Institute of Labor Economics (IZA)	Incorrect exposure
402	Strandh 2001	State intervention and mental well-being among the unemployed	Journal of Social Policy	Incorrect exposure
403	Strauss 2004	Indonesian living standards: Before and after the financial crisis	RAND Corporation Monograph Series. Santa Monica, Calif.: RAND Corporation; Singapore: Institute of Southeast Asian Studies	Incorrect study type
404	Stuckler 2017	Austerity and health: the impact in the UK and Europe	European Journal of Public Health	Incorrect study type
405	Swift 2019	Causal inference approaches for understanding the social determinants of substance abuse and depression in the 21st century: The opioid epidemic and the great recession	University of Miami	Unable to source full text
406	Tainaka 1998	Workers' stress after Hanshin-Awaji earthquake in 1995--symptoms related to stress after 18 months. [Japanese]	Sangyo eiseigaku zasshi = Journal of occupational health	Incorrect exposure
407	Tainaka 1998	[Workers' stress after Hanshin-Awaji earthquake in 1995--symptoms related to stress after 18 months]	Sangyo Eiseigaku Zasshi	Exact duplicate
408	Tamayofonseca 2018	Contribution of the economic crisis to the risk increase of poor mental health in a region of spain	International Journal of Environmental Research and Public Health	Incorrect exposure
409	Thiede 1997	Mutual influences of health and poverty. Evidence from German panel data	Social Science and Medicine	No relevant outcome(s)
410	Thomas 2007	Psychological distress after employment transitions: the role of subjective financial position as a mediator	Journal of Epidemiology & Community Health	Incorrect exposure
411	Tiwari 2019	Long-Term Effects of Temporary Income Shocks on Food Consumption and Subjective Well-Being	Journal of Development Studies	No relevant outcome(s)
412	Todd 2007	Three Essays on the Indirect Impacts of Conditional Cash Transfer Programs in Mexico and Nicaragua	American University	No relevant outcome(s)
413	Tsai 2016	Associations of various perceived-stress situations with depressive symptoms in >=50-year old Taiwanese men and women: Results from the Taiwan Longitudinal Study on Aging	Archives of Gerontology & Geriatrics	Incorrect exposure
414	Tseng 2012	Handling the endogeneity of income to health using a field experiment in Taiwan	University of Dundee, Economic Studies, Discussion Papers: 263	Incorrect population
415	Tsui 2014	What Affects Happiness: Absolute Income, Relative Income or Expected Income?	Journal of Policy Modeling	Incorrect exposure
416	Ullah 1990	The association between income, financial strain and psychological well-being among unemployed youths	Journal of Occupational Psychology	Incorrect exposure
417	Unger 2018	Age and gender differences in the impact of labour-market transitions on subjective health in Germany	Scandinavian Journal of Public Health	Incorrect exposure
418	Urbanos-Garrido 2013	Desempleo y salud: Un analisis de la repercusion de la crisis economica sobre la salud de los espanoles. (Unemployment and Health: An Analysis of the Impact of the Economic Crisis on the Health of the Spanish Population. With English summary.)	Estudios de Economia Aplicada	Incorrect language
419	VandenBroeck 2017	Does Off-Farm Wage Employment Make Women in Rural Senegal Happy?	Feminist Economics	Incorrect exposure

420	vanderVeen 2000	Basic income on the agenda: Policy objectives and political chances	Amsterdam: Amsterdam University Press; distributed by University of Michigan Press, Ann Arbor	Incorrect study type
421	Varner 2009	Marital Transitions and Changes in African American Mothers' Depressive Symptoms: The Buffering Role of Financial Resources	Journal of Family Psychology	Incorrect exposure
422	Varner 2009	Marital Transitions and Changes in African American Mothers' Depressive Symptoms: The Buffering Role of Financial Resources: JFP JFP	Journal of Family Psychology	Exact duplicate
423	Vemuri 2011	A Tale of Two Scales: Evaluating the Relationship Among Life Satisfaction, Social Capital, Income, and the Natural Environment at Individual and Neighborhood Levels in Metropolitan Baltimore	Environment and Behavior	Incorrect exposure
424	Vendrik 2013	Adaptation, anticipation and social interaction in happiness: An integrated error-correction approach	Journal of Public Economics	Duplicate sample
425	Vietch 1995	Prescribing citizens advice: an evaluation of the work of the citizens advice bureau with Health and Social Services in Birmingham	Birmingham, District Citizens Advice Bureau	Unable to source full text
426	Vilches 2014	POST-CATASTROPHE SOCIAL-ENVIRONMENTAL EFFECTS IN VULNERABLE COASTAL AREAS AFFECTED BY THE TSUNAMI OF 02/27/2010 IN CHILE	Interciencia	Incorrect language
427	Vinokur 1996	Hard times and hurtful partners: How financial strain affects depression and relationship satisfaction of unemployed persons and their spouses	Journal of personality and social psychology	Incorrect exposure
428	Vitturi 2018	Social consequences and quality of life of patients with myasthenia gravis	Journal of Neuromuscular Diseases	Incorrect study type
429	Voss 2017	Honest Labor Bears a Lovely Face: Will Late-Life Unemployment Impact Health and Satisfaction in Retirement?	Journal of Occupational & Environmental Medicine	Incorrect exposure
430	Wahlbeck 2017	Interventions to mitigate the effects of poverty and inequality on mental health	Social Psychiatry & Psychiatric Epidemiology	Incorrect study type
431	Wang 2019	The short-term impact of unconditional cash transfers: a replication study of a randomized controlled trial in Kenya	Journal of Development Effectiveness	Duplicate sample
432	Wang 2019	Gains and losses: Does farmland acquisition harm farmers' welfare?	Land Use Policy	Incorrect exposure
433	Waqas 2018	Access to better health? The impact of the Benazir Income Support Programme in Pakistan	Asia Pacific Journal of Public Administration	No relevant outcome(s)
434	Wedcliffe 2001	The psychological effects of traumatic brain injury on the quality of life of a group of spouses/partners	South African Journal of Communication Disorders - die Suid-Afrikaanse Tydskrif vir Kommunikasieafwykings	No relevant outcome(s)
435	Weich 1998	Poverty, unemployment, and common mental disorders: population based cohort study	Bmj-British Medical Journal	Incorrect exposure
436	White 2018	Two Year Sustainability of the Effect of a Financial Education Program on the Health and Wellbeing of Single, Low-Income Women	Journal of Financial Counseling and Planning	No comparator group
437	Wickham 2016	Impact of moving into poverty on maternal and child mental health: Longitudinal analysis of the Millennium Cohort Study	The Lancet	Exact duplicate
438	Wilkinson 2016	Financial Strain and Mental Health Among Older Adults During the Great Recession	Journals of Gerontology Series B-Psychological Sciences & Social Sciences	Incorrect population

439	Wu 1996	Marital disruption and change in mental health	University of Nebraska	Unable to source full text
440	Wu 2018	Financial transfers from adult children and depressive symptoms among mid-aged and elderly residents in China - evidence from the China health and retirement longitudinal study	BMC Public Health	Incorrect exposure
441	Yoon 2003	Factors associated with family caregivers' burden and depression in Korea	International Journal of Aging & Human Development	Incorrect population
442	Young 2012	Losing a Job: The Nonpecuniary Cost of Unemployment in the United States	Social Forces	Duplicate sample
443	Zavisca 2005	Does Money Buy Happiness in Unhappy Russia?	Institute of Slavic, East European, and Eurasian Studies, UC Berkeley	Duplicate sample
444	Zhang 2007	Wealth and health: What's the magnitude of effect of permanent income and transitory income on mental health?	Journal of Mental Health Policy and Economics	Incorrect study type
445	Ziersch 2014	A longitudinal study of the mental health impacts of job loss: the role of socioeconomic, sociodemographic, and social capital factors	Journal of Occupational & Environmental Medicine	Incorrect exposure
446	Zvorsky 2018	Effects of Financial Incentives for Smoking Cessation on Mood and Anxiety Symptoms Among Pregnant and Newly Postpartum Women	Nicotine & Tobacco Research	No comparator group

Appendix F: Additional tables

Table F1: Results of meta-regression for all main analyses. *Individual and fully adjusted models shown, results with p<0.05 in bold. Reference categories: Income source = unknown; Direction = no distinction; If earned = unearned; Conditionality = Yes; Size = includes both; SEP = mixed; Setting = High income country; Outcome measure = admin data; Poverty transition = no; Outcome type = life satisfaction*

Model	Exposure variable	Coefficient (95% CI)	p-value
Income increases and mental health outcomes (Figure 3A)			
Income source	Lottery win	-0.08 (-0.28, 0.11)	0.410
Income source	Tax/wage policy	0.08 (-0.08, 0.23)	0.353
Income source	Cash transfer (not welfare policy)	-0.01 (-0.17, 0.15)	0.931
Income source	Welfare policy (cash transfer only)	0.03 (-0.10, 0.16)	0.620
Income source	**Other welfare policy**	**0.18 (0.02, 0.34)**	**0.025**
Direction	Increase only	0.02 (-0.14, 0.18)	0.804
If earned	Unearned income	-0.02 (-0.13, 0.10)	0.779
Conditionality	Yes	0.04 (-0.08, 0.16)	0.500
Size	Small change	0.02 (-0.13, 0.17)	0.787
Size	Large change	0.03 (-0.09, 0.15)	0.614
SEP	Low	0.08 (-0.01, 0.17)	0.080
SEP	High	-0.01 (-0.39, 0.38)	0.973
Setting	Low/middle income	0.07 (-0.02, 0.15)	0.146
Outcome measure	Clinical interview	0.09 (-0.25, 0.43)	0.614
Outcome measure	Self report - survey Q	0.07 (-0.22, 0.36)	0.630
Outcome measure	Self report - validated tool	0.08 (-0.17, 0.33)	0.543
**Poverty transition**	**Yes**	**0.11 (0.03, 0.19)**	**0.005**
Adjusted	**Lottery win**	**0.56 (0.04, 1.08)**	**0.036**
Adjusted	**Tax/wage policy**	**0.58 (0.12, 1.04)**	**0.013**
Adjusted	**Cash transfer (not welfare policy)**	**0.54 (0.08, 0.99)**	**0.020**
Adjusted	Welfare policy (cash transfer only)	0.40 (-0.07, 0.87)	0.098
Adjusted	**Other welfare policy**	**0.50 (0.02, 0.98)**	**0.042**
Adjusted	Increase only	-0.08 (-0.34, 0.18)	0.532
Adjusted	Unearned income	-0.22 (-0.45, 0.01)	0.061
Adjusted	Conditionality	-0.01 (-0.18, 0.15)	0.872
Adjusted	Small change	0.21 (-0.05, 0.48)	0.110
Adjusted	Large change	0.03 (-0.18, 0.24)	0.787
Adjusted	Low SEP	-0.13 (-0.30, 0.03)	0.112
Adjusted	High SEP	-0.01 (-0.44, 0.41)	0.949
Adjusted	Clinical interview	0.36 (-0.05, 0.77)	0.082
Adjusted	Self report - survey Q	0.05 (-0.25, 0.34)	0.752
Adjusted	Self report - validated tool	0.01 (-0.31, 0.33)	0.946
Adjusted	**Yes (poverty transition)**	**0.40 (0.14, 0.67)**	**0.003**
Income decreases and mental health outcomes (Figure 3B)			
Income source	**Disaster**	**-0.48 (-0.88, -0.07)**	**0.023**
Income source	Salary change	0.10 (-0.16, 0.36)	0.451

Income source	Welfare policy (cash transfer only)	0.05 (-0.20, 0.31)	0.693
If earned	Unearned income	0.07 (-0.23, 0.37)	0.633
Size	Large change	-0.08 (-0.27, 0.12)	0.439
Setting	Low/middle income	0.13 (-0.17, 0.42)	0.403
Outcome measure	Self report - survey Q	0.17 (-0.18, 0.52)	0.328
Outcome measure	Self report - validated tool	0.13 (-0.12, 0.38)	0.309
Poverty transition	Yes	0.14 (-0.06, 0.33)	0.166
Adjusted	**Disaster**	**-0.51 (-0.93, -0.09)**	**0.018**
Adjusted	Salary change	0.07 (-0.21, 0.34)	0.643
Adjusted	Welfare policy (cash transfer only)	0.24 (-0.08, 0.55)	0.138
Adjusted	Large change	-0.11 (-0.35, 0.12)	0.335
Adjusted	Self report - validated tool	0.11 (-0.13, 0.34)	0.384
Adjusted	Yes (poverty transition)	0.07 (-0.16, 0.29)	0.568
Income increases and wellbeing outcomes (Figure 4A)			
Income source	Lottery win	-0.04 (-0.61, 0.53)	0.897
Income source	Tax/wage policy	-0.02 (-0.45, 0.41)	0.941
Income source	Cash transfer (not welfare policy)	0.60 (-0.12, 1.33)	0.100
Income source	Welfare policy (cash transfer only)	0.33 (-0.01, 0.68)	0.059
Income source	Other welfare policy	0.09 (-0.32, 0.51)	0.655
Direction	Increase only	0.23 (-0.17, 0.63)	0.255
If earned	Unearned income	0.24 (-0.05, 0.53)	0.110
Conditionality	Yes	0.28 (-0.05, 0.61)	0.101
Size	Small change	0.11 (-0.26, 0.47)	0.557
Size	Large change	0.24 (-0.11, 0.58)	0.179
SEP	Low	0.25 (-0.03, 0.52)	0.082
SEP	High	0.25 (-0.36, 0.86)	0.422
Setting	Low/middle income	0.20 (-0.05, 0.44)	0.111
Outcome measure	Self report - validated tool	0.17 (-0.29, 0.63)	0.465
Poverty transition	Yes	0.15 (-0.12, 0.41)	0.279
Adjusted	Lottery win	-1.05 (-3.73, 1.64)	0.444
Adjusted	Tax/wage policy	-0.60 (-2.88, 1.68)	0.607
Adjusted	Cash transfer (not welfare policy)	-0.85 (-4.38, 2.69)	0.638
Adjusted	Welfare policy (cash transfer only)	-0.84 (-3.39, 1.71)	0.517
Adjusted	Other welfare policy	-1.06 (-3.63, 1.51)	0.420
Adjusted	Increase only	0.30 (-1.52, 2.12)	0.748
Adjusted	Unearned income	0.94 (-1.51, 3.40)	0.452
Adjusted	Conditionality	0.53 (-0.27, 1.32)	0.194
Adjusted	Small change	-0.70 (-2.60, 1.21)	0.473
Adjusted	Large change	-0.20 (-1.76, 1.37)	0.803
Adjusted	Low SEP	0.29 (-1.11, 1.68)	0.687
Adjusted	High SEP	0.07 (-1.97, 2.12)	0.944
Adjusted	Happiness measure	-0.18 (-0.99, 0.63)	0.663
Adjusted	Quality of life measure	0.43 (-1.12, 1.99)	0.584
Adjusted	Self report - validated tool	-0.31 (-1.32, 0.70)	0.551

Table F2: GRADE summary of findings and certainty of evidence for each outcome domain

**Income changes and mental health/wellbeing outcomes**						
**Population/setting:** General population in low, middle and high-income settings<br>**Intervention:** An income change from any source<br>**Comparison:** Not receiving an equivalent income change						
Outcomes	Risk with no intervention	Risk with intervention (95% CI)	Effect estimate (95% CI)	No. of participants (studies)	Certainty of the evidence (GRADE)	Comments
**1. Income changes and grouped mental health outcomes e.g. CES-D (range 0-60)**						
EFFECT DIRECTION						
a) Any income change	88.9% of studies reported a beneficial effect of income changes on mental health (95% CI 77.4%, 95.8%) i.e., an increase was associated with improvement and/or a decrease was associated with worsening			922,428 (54 studies)	⊕⊕⊖⊖ **Low**[a,b]	Downgraded for inconsistency and risk of bias<br><br>Income has a beneficial effect on mental health
EFFECT SIZE						
b) 10% income increase	Mean CES-D score 16.01[c]	Mean **0.03 pts higher** (0.0003 to 0.005 pts)[d]	Std. β 0.003 (0.0003, 0.005)	1,510,666 (9 studies)	⊕⊕⊖⊖ **Low**[a,e]	Downgraded for inconsistency and risk of bias<br><br>10% income increases result in a very small improvement in mental health
c) Crossing poverty/ subsistence threshold	Mean CES-D score 16.01[c]	Mean **1.26 pts higher** (0.68 to 1.93 pts)[d]	SMD 0.13 (0.07, 0.20)	42,128 (18 studies)	⊕⊕⊖⊖ **Low**[a,f,g]	Downgraded for inconsistency and indirectness<br><br>Moving individuals out of poverty results in a small improvement in mental health
d) Income decrease (mixed amounts)	Mean CES-D score 16.01[c]	Mean **2.03 pts lower** (1.26 to 2.90 pts)[d]	SMD -0.21 (-0.30, -0.13)	227,804 (11 studies)	⊕⊖⊖⊖ **Very low**[a,h,i]	Downgraded for inconsistency, risk of bias and publication bias<br><br>Income decreases may result in a small worsening in mental health

**2. Income changes and grouped wellbeing outcomes e.g. life satisfaction (range 0-10)**						
EFFECT DIRECTION						
a) Any income change	95.0% of studies reported a beneficial effect of income changes on wellbeing (95% CI 83.1%, 99.4%) i.e., an increase was associated with improvement and/or a decrease was associated with worsening			311,219<br>(40 studies)	⊕⊕⊖⊖<br>**Low**[a,b]	Downgraded for inconsistency and risk of bias<br><br>Income has a beneficial effect on wellbeing
EFFECT SIZE						
b) 10% income increase	Mean life satisfaction score 7.36[j]	Mean **0.005 pts higher** (0.003 to 0.009 pts)[k]	Std. β 0.003 (0.002, 0.005)	105,326<br>(9 studies)	⊕⊖⊖⊖<br>**Very low**[a,e,i]	Downgraded for inconsistency, risk of bias and publication bias<br><br>10% income increases may result in a very small improvement in wellbeing
c) Crossing poverty/ subsistence threshold	Mean life satisfaction score 7.36[j]	Mean **0.65 pts higher** (0.15 to 1.14 pts)[k]	SMD 0.38 (0.09, 0.66)	101,350<br>(8 studies)	⊕⊕⊖⊖<br>**Low**[a,l,m]	Downgraded for inconsistency and risk of bias<br><br>Moving individuals out of poverty results in a moderate improvement in wellbeing
d) Income decrease (mixed amounts)						Insufficient evidence
**3. Changes in unearned income and mental health/wellbeing outcomes**						
Any						Insufficient evidence[n]

GRADE Working Group grades of evidence
High certainty ⊕⊕⊕⊕**:** We are very confident that the true effect lies close to that of the estimate of the effect
Moderate certainty ⊕⊕⊕⊖**:** We are moderately confident in the effect estimate: The true effect is likely to be close to the estimate of the effect, but there is a possibility that it is substantially different
Low certainty ⊕⊕⊖⊖**:** Our confidence in the effect estimate is limited: The true effect may be substantially different from the estimate of the effect
Very low certainty ⊕⊖⊖⊖**:** We have very little confidence in the effect estimate: The true effect is likely to be substantially different from the estimate of effect

Explanations

a) Downgraded for serious inconsistency as heterogeneity of included studies was considerable (I^2 >75% in meta-analyses)
b) Downgraded for RoB as though critical RoB studies were excluded, most included studies were of serious RoB
c) Based on n=7 included studies which reported mean CES-D scores in control groups (the most commonly reported mental health outcome)
d) Based on n=6 included studies which reported SD of CES-D scores (9.67)
e) Downgraded for RoB as all but one included studies were rated serious RoB
f) Downgraded for serious indirectness: half of the 18 studies (n=9) have either conditionality attached to the cash transfers or an important co-intervention, where it is therefore unclear to what degree the associated factors (e.g. school attendance, engagement with healthcare or training) contributed to the change in outcome
g) **Not** downgraded for RoB as most included studies were RCTs, majority of studies were rated low/moderate RoB, and no difference in effect size in serious RoB studies on stratification
h) Downgraded for RoB given most studies were rated serious RoB and stratified analyses indicate differing effect sizes for serious vs low/moderate RoB studies
i) Downgraded for suspected publication bias given impression from funnel plot
j) Based on n=4 included studies which reported mean self-rated life satisfaction on 11 point scale in control groups (the most commonly reported wellbeing outcome)
k) Based on n=6 included studies which reported SD of self-rated life satisfaction on 11 point scale (1.72)
l) Downgraded for RoB as though majority of included studies are low/moderate RoB, the two contributing serious RoB studies report notably larger effect sizes
m) **Not** downgraded for indirectness as only a quarter of the 8 included studies (n=2) have an important co-intervention or conditionality
n) Robust stratified analyses directly comparing earned and unearned income sources were not possible, as individual meta-analyses included either almost exclusively unearned income changes or a combination of earned and unknown sources; readers should note that the vast majority of studies contributing to outcomes 1c) and 2c) were interventions on unearned income

Appendix G: Additional graphs

Effect direction plots

Figure G1: Effect direction plot for all studies reporting the relationship between income changes and mental health outcomes, stratified by risk of bias (n=90)

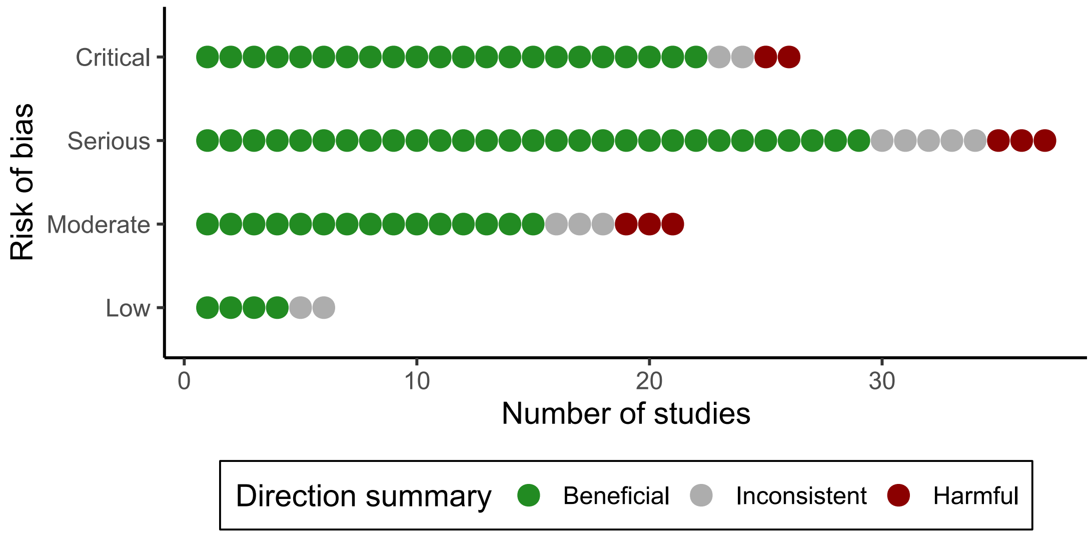

A beneficial effect direction indicates either that an income increase was associated with an improvement in outcome or that an income decrease was associated with a worsening in outcome. A harmful effect direction indicates the opposite relationship. An inconsistent rating indicates that <70% of extracted datapoints for the study reported a consistent effect direction.

Figure G2: Effect direction plot for all studies reporting the relationship between income changes and wellbeing outcomes, stratified by risk of bias (n=64)

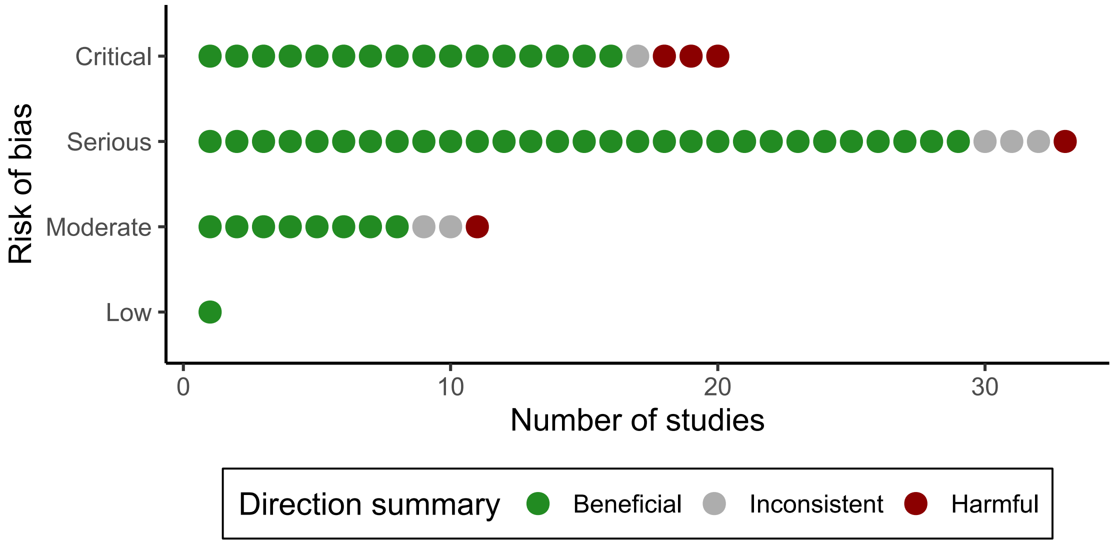

A beneficial effect direction indicates either that an income increase was associated with an improvement in outcome or that an income decrease was associated with a worsening in outcome. A harmful effect direction indicates the opposite relationship. An inconsistent rating indicates that <70% of extracted datapoints for the study reported a consistent effect direction.

Stratified forest plots for main subgroup analysis

Figure G3: Forest plot for meta-analysis of studies reporting the effect of a binary income increase on a mental health outcome, stratified by sex

Source	RCT	Inc. source	Outcome	Country	RoB	SMD	95% CI	Weight
**Sex: Both**								
Priebe	+	Other transfer	CGI scale	GBR	Some conc.	0.554	[-0.245; 1.352]	0.3%
Haushofer	+	Welf. policy (£)	CES-D	KEN	Some conc.	0.125	[0.032; 0.217]	3.5%
Courtin	+	Welf. policy (£)	Kessler	USA	Some conc.	0.106	[-0.085; 0.298]	2.4%
Green	+	Welf. policy (£+)	APAI	UGA	Low	0.057	[-0.054; 0.168]	3.3%
Apouey	.	Lottery win	GHQ	GBR	Serious	0.079	[0.025; 0.132]	3.9%
Raschke (high)	.	Lottery win	SF-MCS	DEU	Serious	-0.012	[-0.326; 0.303]	1.2%
Raschke (low)	.	Lottery win	SF-MCS	DEU	Serious	-0.264	[-0.467; -0.062]	2.1%
Erixson	.	Tax/wage policy	MH hosp.	SWE	Low	0.011	[-0.006; 0.028]	4.1%
Burmaster	.	Tax/wage policy	CES-D	DOM	Serious	0.466	[0.198; 0.733]	1.7%
Reeves (2017)	.	Tax/wage policy	GHQ	GBR	Serious	0.534	[0.178; 0.889]	1.2%
Koltai	.	Unknown	Kessler	CAN	Moderate	0.017	[-0.049; 0.083]	3.8%
McCarthy	.	Unknown	Beck	CAN/USA	Serious	0.018	[-0.098; 0.135]	3.3%
Lorant	.	Unknown	HDL	BEL	Serious	0.055	[0.020; 0.090]	4.0%
Sareen	.	Unknown	Inc. mood dis.	USA	Serious	-0.042	[-0.183; 0.098]	3.0%
Costello	.	Other transfer	CAPA	USA	Moderate	0.361	[-0.059; 0.780]	0.9%
Tachibana	.	Other transfer	CES-D	NPL	Serious	0.018	[0.003; 0.033]	4.1%
AIR	.	Welf. policy (£)	CES-D	ZWE	Low	-0.135	[-0.408; 0.138]	1.7%
Gros	.	Welf. policy (£)	Subj. MH	BGD	Serious	0.332	[0.088; 0.577]	1.9%
**Random effects model**						0.054	[0.014; 0.094]	46.4%
Heterogeneity: I^2 = 69%								
**Sex: Male**								
Kilburn (2016, m)	+	Welf. policy (£)	CES-D	KEN	Low	0.282	[0.120; 0.444]	2.3%
Bedoya (m)	+	Welf. policy (£+)	CES-D	AFG	Some conc.	0.198	[0.045; 0.351]	2.2%
Ong (m)	.	Other transfer	SF-MCS	AUS	Moderate	-0.003	[-0.023; 0.017]	3.1%
**Random effects model**						0.143	[-0.033; 0.319]	7.6%
Heterogeneity: I^2 = 89%								
**Sex: Female**								
Kilburn (2016, f)	+	Welf. policy (£)	CES-D	KEN	Low	-0.037	[-0.236; 0.161]	1.9%
Fernald	+	Welf. policy (£)	CES-D	ECU	Some conc.	-0.065	[-0.207; 0.077]	3.0%
Baird (a)	+	Welf. policy (£)	GHQ	MWI	Some conc.	-0.009	[-0.168; 0.150]	2.1%
Baird (b)	+	Welf. policy (£)	GHQ	MWI	Some conc.	0.154	[0.049; 0.260]	2.8%
Hjelm (CGP)	+	Welf. policy (£)	PSS	ZMB	Some conc.	-0.017	[-0.172; 0.139]	2.3%
Hjelm (MCP)	+	Welf. policy (£)	PSS	ZMB	Some conc.	0.089	[-0.060; 0.238]	2.3%
Bedoya (f)	+	Welf. policy (£+)	CES-D	AFG	Some conc.	0.504	[0.388; 0.620]	2.7%
Gassman-Pines	+	Welf. policy (£+)	CES-D	USA	Some conc.	0.208	[-0.027; 0.444]	2.0%
Gennetian	+	Welf. policy (£+)	CES-D	USA	Some conc.	0.129	[-0.033; 0.290]	2.7%
Macours	+	Welf. policy (£+)	CES-D	NIC	Some conc.	0.605	[-0.863; 2.073]	0.1%
Boyd-Swan	.	Tax/wage policy	CES-D	USA	Serious	0.077	[-0.035; 0.189]	3.3%
Evans (2010)	.	Tax/wage policy	Subj. MH	USA	Serious	0.009	[0.001; 0.017]	4.1%
Dearing	.	Unknown	CES-D	USA	Serious	0.216	[0.008; 0.424]	2.2%
Ong (f)	.	Other transfer	SF-MCS	AUS	Moderate	-0.011	[-0.031; 0.009]	3.1%
Ozer	.	Welf. policy (£)	CES-D	MEX	Moderate	0.168	[0.094; 0.241]	3.7%
Powell-Jackson	.	Welf. policy (£)	Kessler	IND	Moderate	0.015	[0.003; 0.026]	4.1%
Lebihan	.	Welf. policy (£)	Mat. dep	CAN	Moderate	0.025	[-0.057; 0.107]	3.6%
**Random effects model**						0.088	[0.018; 0.158]	46.0%
Heterogeneity: I^2 = 86%								
**Random effects model**						**0.084**	**[0.038; 0.130]**	**100.0%**

Heterogeneity: I^2 = 80%
Test for overall effect: p < 0.001
Test for SG diffs: χ^2_2 = 1.48, df = 2 (p = 0.48)

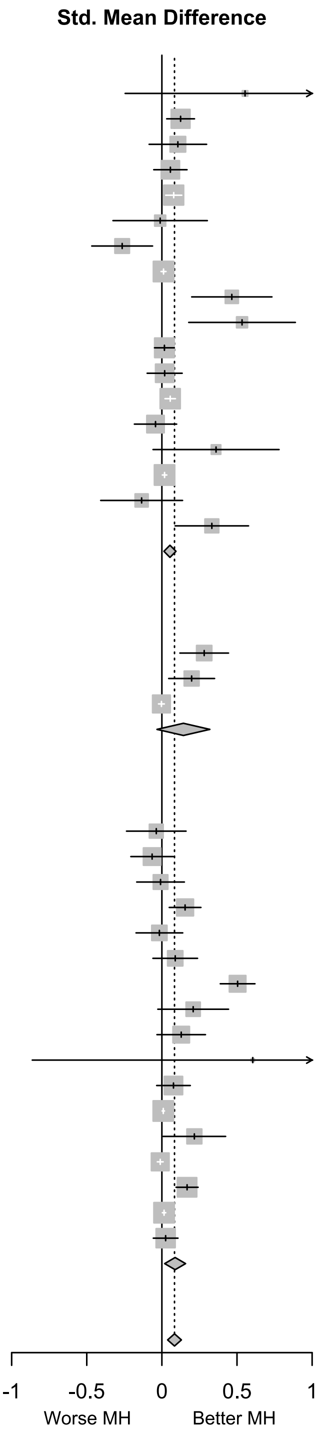

Number of people = 258,637; number of observations = 1,756,078. Studies sorted by RCT/NRS status → income source → outcome → RoB. + indicates study was a randomised controlled trial. (£) indicates a welfare policy which only affected income; (£+) indicates a welfare policy which also influenced other factors. (f) and (m) indicate studies which stratified results by sex; (high) and (low) indicate Raschke et al. stratified results by educational attainment. Baird (a) reported on a cash transfer to school dropouts versus Baird (b) which reported on a cash transfer to girls still enrolled in school; Hjelm (CGP) reported on the Zambia Child Grant Program versus Hjelm (MCP) which reported on the Zambia Multiple Category Cash Transfer Program. RoB = Risk of Bias score; SG = subgroup. APAI = Acholi Psychosocial Assessment Instrument. Beck = Beck depression inventory. CAPA = Child and Adolescent Psychiatric Assessment. CES-D = Center for Epidemiological Studies-Depression scale. CGI = Clinical Global Impression scale. GHQ = General Health Questionnaire. HDL = Health and Daily Living

Form. Inc. mood dis. = Incident mood disorder (assessed at clinical interview). Kessler = Kessler Psychological Distress scale. Mat. dep = measure of maternal depression. MH hosp. = mental health hospitalisations. PSS = Perceived Stress Scale. SF-MCS = Mental Component Summary of Short Form Survey. Subj. MH = measure of subjective/self-assessed mental health.

Figure G4: Forest plot for meta-analysis of studies reporting the effect of a binary income increase on a mental health outcome, stratified by study design

Source	RCT	Inc. source	Outcome	Country	RoB	SMD	95% CI	Weight
**Study Design: NRS**								
Apouey	.	Lottery win	GHQ	GBR	Serious	0.079	[0.025; 0.132]	3.9%
Raschke (high)	.	Lottery win	SF-MCS	DEU	Serious	-0.012	[-0.326; 0.303]	1.2%
Raschke (low)	.	Lottery win	SF-MCS	DEU	Serious	-0.264	[-0.467; -0.062]	2.1%
Erixson	.	Tax/wage policy	MH hosp.	SWE	Low	0.011	[-0.006; 0.028]	4.1%
Boyd-Swan	.	Tax/wage policy	CES-D	USA	Serious	0.077	[-0.035; 0.189]	3.3%
Burmaster	.	Tax/wage policy	CES-D	DOM	Serious	0.466	[0.198; 0.733]	1.7%
Reeves (2017)	.	Tax/wage policy	GHQ	GBR	Serious	0.534	[0.178; 0.889]	1.2%
Evans (2010)	.	Tax/wage policy	Subj. MH	USA	Serious	0.009	[0.001; 0.017]	4.1%
Koltai	.	Unknown	Kessler	CAN	Moderate	0.017	[-0.049; 0.083]	3.8%
McCarthy	.	Unknown	Beck	CAN/USA	Serious	0.018	[-0.098; 0.135]	3.3%
Dearing	.	Unknown	CES-D	USA	Serious	0.216	[0.008; 0.424]	2.2%
Lorant	.	Unknown	HDL	BEL	Serious	0.055	[0.020; 0.090]	4.0%
Sareen	.	Unknown	Inc. mood dis.	USA	Serious	-0.042	[-0.183; 0.098]	3.0%
Costello	.	Other transfer	CAPA	USA	Moderate	0.361	[-0.059; 0.780]	0.9%
Ong (f)	.	Other transfer	SF-MCS	AUS	Moderate	-0.011	[-0.031; 0.009]	3.1%
Ong (m)	.	Other transfer	SF-MCS	AUS	Moderate	-0.003	[-0.023; 0.017]	3.1%
Tachibana	.	Other transfer	CES-D	NPL	Serious	0.018	[0.003; 0.033]	4.1%
AIR	.	Welf. policy (£)	CES-D	ZWE	Low	-0.135	[-0.408; 0.138]	1.7%
Ozer	.	Welf. policy (£)	CES-D	MEX	Moderate	0.168	[0.094; 0.241]	3.7%
Powell-Jackson	.	Welf. policy (£)	Kessler	IND	Moderate	0.015	[0.003; 0.026]	4.1%
Lebihan	.	Welf. policy (£)	Mat. dep	CAN	Moderate	0.025	[-0.057; 0.107]	3.6%
Gros	.	Welf. policy (£)	Subj. MH	BGD	Serious	0.332	[0.088; 0.577]	1.9%
**Random effects model**						0.043	[0.012; 0.074]	64.1%
Heterogeneity: $I^2 = 74\%$								
**Study Design: RCT**								
Priebe	+	Other transfer	CGI scale	GBR	Some conc.	0.554	[-0.245; 1.352]	0.3%
Kilburn (2016, f)	+	Welf. policy (£)	CES-D	KEN	Low	-0.037	[-0.236; 0.161]	1.9%
Kilburn (2016, m)	+	Welf. policy (£)	CES-D	KEN	Low	0.282	[0.120; 0.444]	2.3%
Fernald	+	Welf. policy (£)	CES-D	ECU	Some conc.	-0.065	[-0.207; 0.077]	3.0%
Haushofer	+	Welf. policy (£)	CES-D	KEN	Some conc.	0.125	[0.032; 0.217]	3.5%
Baird (a)	+	Welf. policy (£)	GHQ	MWI	Some conc.	-0.009	[-0.168; 0.150]	2.1%
Baird (b)	+	Welf. policy (£)	GHQ	MWI	Some conc.	0.154	[0.049; 0.260]	2.8%
Courtin	+	Welf. policy (£)	Kessler	USA	Some conc.	0.106	[-0.085; 0.298]	2.4%
Hjelm (CGP)	+	Welf. policy (£)	PSS	ZMB	Some conc.	-0.017	[-0.172; 0.139]	2.3%
Hjelm (MCP)	+	Welf. policy (£)	PSS	ZMB	Some conc.	0.089	[-0.060; 0.238]	2.3%
Green	+	Welf. policy (£+)	APAI	UGA	Low	0.057	[-0.054; 0.168]	3.3%
Bedoya (f)	+	Welf. policy (£+)	CES-D	AFG	Some conc.	0.504	[0.388; 0.620]	2.7%
Bedoya (m)	+	Welf. policy (£+)	CES-D	AFG	Some conc.	0.198	[0.045; 0.351]	2.2%
Gassman-Pines	+	Welf. policy (£+)	CES-D	USA	Some conc.	0.208	[-0.027; 0.444]	2.0%
Gennetian	+	Welf. policy (£+)	CES-D	USA	Some conc.	0.129	[-0.033; 0.290]	2.7%
Macours	+	Welf. policy (£+)	CES-D	NIC	Some conc.	0.605	[-0.863; 2.073]	0.1%
**Random effects model**						0.130	[0.047; 0.213]	35.9%
Heterogeneity: $I^2 = 77\%$								
**Random effects model**						**0.084**	**[0.038; 0.130]**	**100.0%**

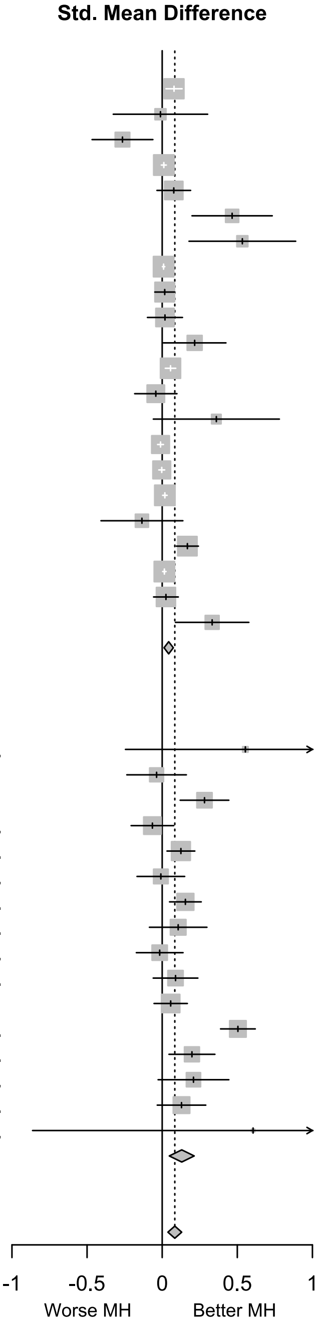

Heterogeneity: $I^2 = 80\%$
Test for overall effect: $p < 0.001$
Test for SG diffs: $\chi^2_1 = 3.70$, df = 1 ($p = 0.05$)

Number of people = 258,637; number of observations = 1,756,078. Studies sorted by RCT/NRS status → income source → outcome → RoB. + indicates study was a randomised controlled trial. (£) indicates a welfare policy which only affected income; (£+) indicates a welfare policy which also influenced other factors. (f) and (m) indicate studies which stratified results by sex; (high) and (low) indicate Raschke et al. stratified results by educational attainment. Baird (a) reported on a cash transfer to school dropouts versus Baird (b) which reported on a cash transfer to girls still enrolled in school; Hjelm (CGP) reported on the Zambia Child Grant Program versus Hjelm (MCP) which reported on the Zambia Multiple Category Cash Transfer Program. RoB = Risk of Bias score; SG = subgroup. APAI = Acholi Psychosocial Assessment Instrument. Beck = Beck depression inventory. CAPA = Child and Adolescent Psychiatric Assessment. CES-D = Center for Epidemiological Studies-Depression scale. CGI = Clinical Global Impression scale. GHQ = General Health Questionnaire. HDL = Health and Daily Living Form. Inc. mood dis. = Incident mood disorder (assessed at clinical interview). Kessler = Kessler Psychological Distress scale.

Mat. dep = measure of maternal depression. MH hosp. = mental health hospitalisations. PSS = Perceived Stress Scale. SF-MCS = Mental Component Summary of Short Form Survey. Subj. MH = measure of subjective/self-assessed mental health.

Figure G5: Forest plot for meta-analysis of studies reporting the effect of a binary income increase on a mental health outcome, stratified by risk of bias

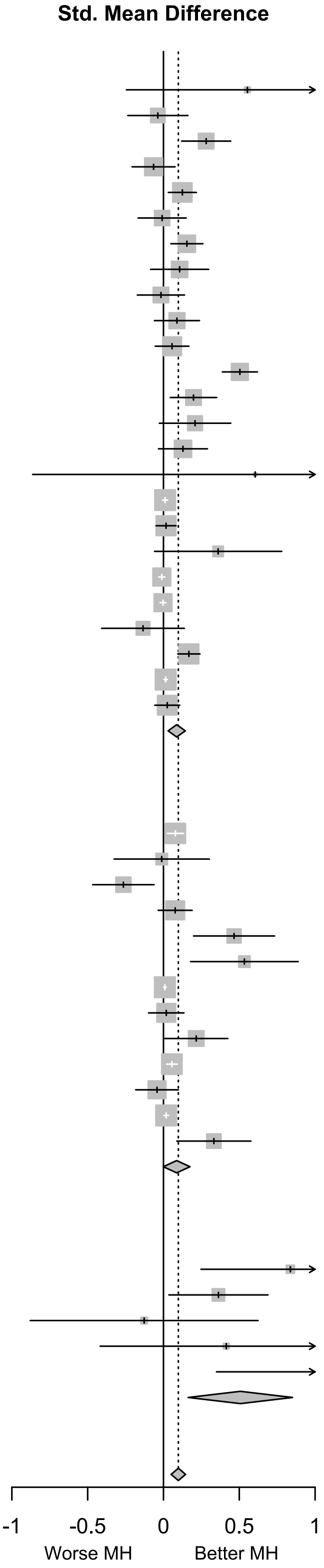

Source	RCT	Inc. source	Outcome	Country	RoB	SMD	95% CI	Weight
**Risk of Bias: Low/Moderate**								
Priebe	+	Other transfer	CGI scale	GBR	Some conc.	0.554	[-0.245; 1.352]	0.3%
Kilburn (2016, f)	+	Welf. policy (£)	CES-D	KEN	Low	-0.037	[-0.236; 0.161]	1.9%
Kilburn (2016, m)	+	Welf. policy (£)	CES-D	KEN	Low	0.282	[0.120; 0.444]	2.2%
Fernald	+	Welf. policy (£)	CES-D	ECU	Some conc.	-0.065	[-0.207; 0.077]	2.9%
Haushofer	+	Welf. policy (£)	CES-D	KEN	Some conc.	0.125	[0.032; 0.217]	3.4%
Baird (a)	+	Welf. policy (£)	GHQ	MWI	Some conc.	-0.009	[-0.168; 0.150]	2.1%
Baird (b)	+	Welf. policy (£)	GHQ	MWI	Some conc.	0.154	[0.049; 0.260]	2.7%
Courtin	+	Welf. policy (£)	Kessler	USA	Some conc.	0.106	[-0.085; 0.298]	2.4%
Hjelm (CGP)	+	Welf. policy (£)	PSS	ZMB	Some conc.	-0.017	[-0.172; 0.139]	2.2%
Hjelm (MCP)	+	Welf. policy (£)	PSS	ZMB	Some conc.	0.089	[-0.060; 0.238]	2.3%
Green	+	Welf. policy (£+)	APAI	UGA	Low	0.057	[-0.054; 0.168]	3.2%
Bedoya (f)	+	Welf. policy (£+)	CES-D	AFG	Some conc.	0.504	[0.388; 0.620]	2.6%
Bedoya (m)	+	Welf. policy (£+)	CES-D	AFG	Some conc.	0.198	[0.045; 0.351]	2.2%
Gassman-Pines	+	Welf. policy (£+)	CES-D	USA	Some conc.	0.208	[-0.027; 0.444]	2.0%
Gennetian	+	Welf. policy (£+)	CES-D	USA	Some conc.	0.129	[-0.033; 0.290]	2.7%
Macours	+	Welf. policy (£+)	CES-D	NIC	Some conc.	0.605	[-0.863; 2.073]	0.1%
Erixson	.	Tax/wage policy	MH hosp.	SWE	Low	0.011	[-0.006; 0.028]	3.9%
Koltai	.	Unknown	Kessler	CAN	Moderate	0.017	[-0.049; 0.083]	3.6%
Costello	.	Other transfer	CAPA	USA	Moderate	0.361	[-0.059; 0.780]	1.0%
Ong (f)	.	Other transfer	SF-MCS	AUS	Moderate	-0.011	[-0.031; 0.009]	2.9%
Ong (m)	.	Other transfer	SF-MCS	AUS	Moderate	-0.003	[-0.023; 0.017]	2.9%
AIR	.	Welf. policy (£)	CES-D	ZWE	Low	-0.135	[-0.408; 0.138]	1.7%
Ozer	.	Welf. policy (£)	CES-D	MEX	Moderate	0.168	[0.094; 0.241]	3.6%
Powell-Jackson	.	Welf. policy (£)	Kessler	IND	Moderate	0.015	[0.003; 0.026]	3.9%
Lebihan	.	Welf. policy (£)	Mat. dep	CAN	Moderate	0.025	[-0.057; 0.107]	3.5%
**Random effects model**						**0.087**	**[0.030; 0.144]**	**62.0%**
Heterogeneity: $I^2 = 83\%$								
**Risk of Bias: Serious**								
Apouey	.	Lottery win	GHQ	GBR	Serious	0.079	[0.025; 0.132]	3.7%
Raschke (high)	.	Lottery win	SF-MCS	DEU	Serious	-0.012	[-0.326; 0.303]	1.2%
Raschke (low)	.	Lottery win	SF-MCS	DEU	Serious	-0.264	[-0.467; -0.062]	2.1%
Boyd-Swan	.	Tax/wage policy	CES-D	USA	Serious	0.077	[-0.035; 0.189]	3.2%
Burmaster	.	Tax/wage policy	CES-D	DOM	Serious	0.466	[0.198; 0.733]	1.8%
Reeves (2017)	.	Tax/wage policy	GHQ	GBR	Serious	0.534	[0.178; 0.889]	1.2%
Evans (2010)	.	Tax/wage policy	Subj. MH	USA	Serious	0.009	[0.001; 0.017]	3.9%
McCarthy	.	Unknown	Beck	CAN/USA	Serious	0.018	[-0.098; 0.135]	3.2%
Dearing	.	Unknown	CES-D	USA	Serious	0.216	[0.008; 0.424]	2.2%
Lorant	.	Unknown	HDL	BEL	Serious	0.055	[0.020; 0.090]	3.8%
Sareen	.	Unknown	Inc. mood dis.	USA	Serious	-0.042	[-0.183; 0.098]	2.9%
Tachibana	.	Other transfer	CES-D	NPL	Serious	0.018	[0.003; 0.033]	3.9%
Gros	.	Welf. policy (£)	Subj. MH	BGD	Serious	0.332	[0.088; 0.577]	1.9%
**Random effects model**						**0.087**	**[-0.001; 0.175]**	**35.0%**
Heterogeneity: $I^2 = 76\%$								
**Risk of Bias: Critical**								
Abbott (2000)	.	Benefits advice	SF-MCS	GBR	Critical	0.837	[0.247; 1.427]	0.6%
Abbott (2006)	.	Benefits advice	SF-MCS	GBR	Critical	0.362	[0.036; 0.689]	1.4%
Robert	.	Unknown	GHQ	ESP	Critical	-0.127	[-0.879; 0.624]	0.4%
Boyce (2009)	.	Unknown	Subj. MH	GHA	Critical	0.414	[-0.418; 1.246]	0.3%
Plagerson	.	Welf. policy (£)	SRQ	ZAF	Critical	1.010	[0.349; 1.671]	0.5%
**Random effects model**						**0.507**	**[0.163; 0.851]**	**3.1%**
Heterogeneity: $I^2 = 42\%$								
**Random effects model**						**0.098**	**[0.050; 0.146]**	**100.0%**
Heterogeneity: $I^2 = 80\%$								
Test for overall effect: $p < 0.001$								
Test for SG diffs: $\chi^2_2 = 5.60$, df = 2 ($p = 0.06$)								

Number of people = 259,412; number of observations = 1,756,853. Studies sorted by RCT/NRS status → income source → outcome → RoB. + indicates study was a randomised controlled trial. (£) indicates a welfare policy which only affected income; (£+) indicates a welfare policy which also influenced other factors. (f) and (m) indicate studies which stratified

results by sex; (high) and (low) indicate Raschke et al. stratified results by educational attainment. Baird (a) reported on a cash transfer to school dropouts versus Baird (b) which reported on a cash transfer to girls still enrolled in school; Hjelm (CGP) reported on the Zambia Child Grant Program versus Hjelm (MCP) which reported on the Zambia Multiple Category Cash Transfer Program. RoB = Risk of Bias score; SG = subgroup. APAI = Acholi Psychosocial Assessment Instrument. Beck = Beck depression inventory. CAPA = Child and Adolescent Psychiatric Assessment. CES-D = Center for Epidemiological Studies-Depression scale. CGI = Clinical Global Impression scale. GHQ = General Health Questionnaire. HDL = Health and Daily Living Form. Inc. mood dis. = Incident mood disorder (assessed at clinical interview). Kessler = Kessler Psychological Distress scale. Mat. dep = measure of maternal depression. MH hosp. = mental health hospitalisations. PSS = Perceived Stress Scale. SF-MCS = Mental Component Summary of Short Form Survey. SRQ = Self-Reporting Questionnaire. Subj. MH = measure of subjective/self-assessed mental health.

Figure G6: Forest plot for meta-analysis of studies reporting the effect of a binary income increase on a mental health outcome, stratified by whether increase likely to move individual out of poverty

Source	RCT	Inc. source	Outcome	Country	RoB	SMD	95% CI	Weight
**Poverty transition: No**								
Priebe	+	Other transfer	CGI scale	GBR	Some conc.	0.554	[-0.245; 1.352]	0.3%
Fernald	+	Welf. policy (£)	CES-D	ECU	Some conc.	-0.065	[-0.207; 0.077]	3.0%
Apouey	.	Lottery win	GHQ	GBR	Serious	0.079	[0.025; 0.132]	3.9%
Raschke (high)	.	Lottery win	SF-MCS	DEU	Serious	-0.012	[-0.326; 0.303]	1.2%
Raschke (low)	.	Lottery win	SF-MCS	DEU	Serious	-0.264	[-0.467; -0.062]	2.1%
Erixson	.	Tax/wage policy	MH hosp.	SWE	Low	0.011	[-0.006; 0.028]	4.1%
Boyd-Swan	.	Tax/wage policy	CES-D	USA	Serious	0.077	[-0.035; 0.189]	3.3%
Reeves (2017)	.	Tax/wage policy	GHQ	GBR	Serious	0.534	[0.178; 0.889]	1.2%
Evans (2010)	.	Tax/wage policy	Subj. MH	USA	Serious	0.009	[0.001; 0.017]	4.1%
Sareen	.	Unknown	Inc. mood dis.	USA	Serious	-0.042	[-0.183; 0.098]	3.0%
Costello	.	Other transfer	CAPA	USA	Moderate	0.361	[-0.059; 0.780]	0.9%
Ong (f)	.	Other transfer	SF-MCS	AUS	Moderate	-0.011	[-0.031; 0.009]	3.1%
Ong (m)	.	Other transfer	SF-MCS	AUS	Moderate	-0.003	[-0.023; 0.017]	3.1%
Tachibana	.	Other transfer	CES-D	NPL	Serious	0.018	[0.003; 0.033]	4.1%
Powell-Jackson	.	Welf. policy (£)	Kessler	IND	Moderate	0.015	[0.003; 0.026]	4.1%
Lebihan	.	Welf. policy (£)	Mat. dep	CAN	Moderate	0.025	[-0.057; 0.107]	3.6%
**Random effects model**						0.011	[0.002; 0.019]	45.0%
Heterogeneity: $I^2 = 60\%$								
**Poverty transition: Yes**								
Kilburn (2016, f)	+	Welf. policy (£)	CES-D	KEN	Low	-0.037	[-0.236; 0.161]	1.9%
Kilburn (2016, m)	+	Welf. policy (£)	CES-D	KEN	Low	0.282	[0.120; 0.444]	2.3%
Haushofer	+	Welf. policy (£)	CES-D	KEN	Some conc.	0.125	[0.032; 0.217]	3.5%
Baird (a)	+	Welf. policy (£)	GHQ	MWI	Some conc.	-0.009	[-0.168; 0.150]	2.1%
Baird (b)	+	Welf. policy (£)	GHQ	MWI	Some conc.	0.154	[0.049; 0.260]	2.8%
Courtin	+	Welf. policy (£)	Kessler	USA	Some conc.	0.106	[-0.085; 0.298]	2.4%
Hjelm (CGP)	+	Welf. policy (£)	PSS	ZMB	Some conc.	-0.017	[-0.172; 0.139]	2.3%
Hjelm (MCP)	+	Welf. policy (£)	PSS	ZMB	Some conc.	0.089	[-0.060; 0.238]	2.3%
Green	+	Welf. policy (£+)	APAI	UGA	Low	0.057	[-0.054; 0.168]	3.3%
Bedoya (f)	+	Welf. policy (£+)	CES-D	AFG	Some conc.	0.504	[0.388; 0.620]	2.7%
Bedoya (m)	+	Welf. policy (£+)	CES-D	AFG	Some conc.	0.198	[0.045; 0.351]	2.2%
Gassman-Pines	+	Welf. policy (£+)	CES-D	USA	Some conc.	0.208	[-0.027; 0.444]	2.0%
Gennetian	+	Welf. policy (£+)	CES-D	USA	Some conc.	0.129	[-0.033; 0.290]	2.7%
Macours	+	Welf. policy (£+)	CES-D	NIC	Some conc.	0.605	[-0.863; 2.073]	0.1%
Burmaster	.	Tax/wage policy	CES-D	DOM	Serious	0.466	[0.198; 0.733]	1.7%
Koltai	.	Unknown	Kessler	CAN	Moderate	0.017	[-0.049; 0.083]	3.8%
McCarthy	.	Unknown	Beck	CAN/USA	Serious	0.018	[-0.098; 0.135]	3.3%
Dearing	.	Unknown	CES-D	USA	Serious	0.216	[0.008; 0.424]	2.2%
Lorant	.	Unknown	HDL	BEL	Serious	0.055	[0.020; 0.090]	4.0%
AIR	.	Welf. policy (£)	CES-D	ZWE	Low	-0.135	[-0.408; 0.138]	1.7%
Ozer	.	Welf. policy (£)	CES-D	MEX	Moderate	0.168	[0.094; 0.241]	3.7%
Gros	.	Welf. policy (£)	Subj. MH	BGD	Serious	0.332	[0.088; 0.577]	1.9%
**Random effects model**						0.134	[0.070; 0.198]	55.0%
Heterogeneity: $I^2 = 78\%$								
**Random effects model**						**0.084**	**[0.038; 0.130]**	**100.0%**

Heterogeneity: $I^2 = 80\%$
Test for overall effect: $p < 0.001$
Test for SG diffs: $\chi^2_1 = 14.14$, df = 1 ($p < 0.01$)

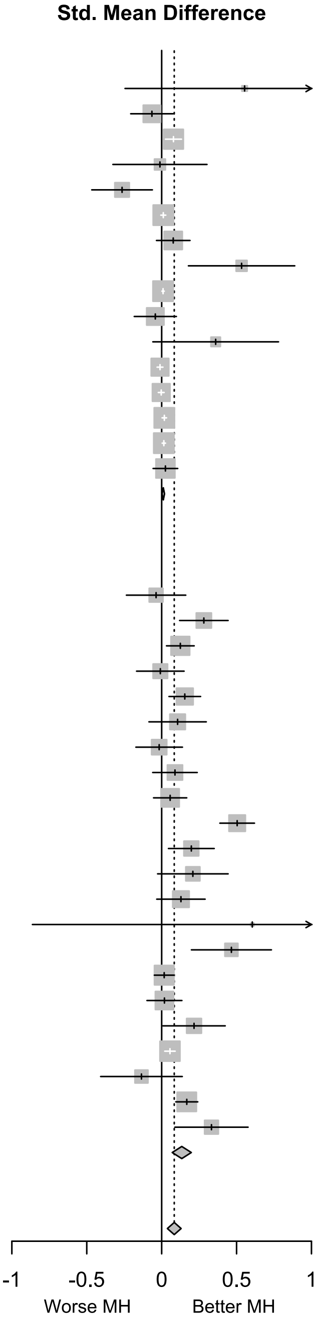

Number of people = 258,637; number of observations = 1,756,078. Studies sorted by RCT/NRS status → income source → outcome → RoB. + indicates study was a randomised controlled trial. (£) indicates a welfare policy which only affected income; (£+) indicates a welfare policy which also influenced other factors. (f) and (m) indicate studies which stratified results by sex; (high) and (low) indicate Raschke et al. stratified results by educational attainment. Baird (a) reported on a cash transfer to school dropouts versus Baird (b) which reported on a cash transfer to girls still enrolled in school; Hjelm (CGP) reported on the Zambia Child Grant Program versus Hjelm (MCP) which reported on the Zambia Multiple Category Cash Transfer Program. RoB = Risk of Bias score; SG = subgroup. APAI = Acholi Psychosocial Assessment Instrument. Beck = Beck depression inventory. CAPA = Child and Adolescent Psychiatric Assessment. CES-D = Center for Epidemiological Studies-Depression scale. CGI = Clinical Global Impression scale. GHQ = General Health Questionnaire. HDL = Health and Daily Living Form. Inc. mood dis. = Incident mood disorder (assessed at clinical interview). Kessler = Kessler Psychological Distress scale. Mat. dep = measure of maternal depression. MH hosp. = mental health hospitalisations. PSS = Perceived Stress Scale. SF-MCS = Mental Component Summary of Short Form Survey. Subj. MH = measure of subjective/self-assessed mental health.

Figure G7: Forest plot for meta-analysis of studies reporting the effect of a binary income increase on a mental health outcome, stratified by socioeconomic position

Source	RCT	Inc. source	Outcome	Country	RoB	SMD	95% CI	Weight
**SEP: Mixed**								
Apouey	.	Lottery win	GHQ	GBR	Serious	0.079	[0.025; 0.132]	3.9%
Erixson	.	Tax/wage policy	MH hosp.	SWE	Low	0.011	[-0.006; 0.028]	4.1%
Evans (2010)	.	Tax/wage policy	Subj. MH	USA	Serious	0.009	[0.001; 0.017]	4.1%
McCarthy	.	Unknown	Beck	CAN/USA	Serious	0.018	[-0.098; 0.135]	3.3%
Dearing	.	Unknown	CES-D	USA	Serious	0.216	[0.008; 0.424]	2.2%
Lorant	.	Unknown	HDL	BEL	Serious	0.055	[0.020; 0.090]	4.0%
Sareen	.	Unknown	Inc. mood dis.	USA	Serious	-0.042	[-0.183; 0.098]	3.0%
Costello	.	Other transfer	CAPA	USA	Moderate	0.361	[-0.059; 0.780]	0.9%
Ong (f)	.	Other transfer	SF-MCS	AUS	Moderate	-0.011	[-0.031; 0.009]	3.1%
Ong (m)	.	Other transfer	SF-MCS	AUS	Moderate	-0.003	[-0.023; 0.017]	3.1%
Powell-Jackson	.	Welf. policy (£)	Kessler	IND	Moderate	0.015	[0.003; 0.026]	4.1%
Lebihan	.	Welf. policy (£)	Mat. dep	CAN	Moderate	0.025	[-0.057; 0.107]	3.6%
**Random effects model**						**0.020**	**[0.002; 0.037]**	**39.4%**
Heterogeneity: I^2 = 59%								
**SEP: Low**								
Priebe	+	Other transfer	CGI scale	GBR	Some conc.	0.554	[-0.245; 1.352]	0.3%
Kilburn (2016, f)	+	Welf. policy (£)	CES-D	KEN	Low	-0.037	[-0.236; 0.161]	1.9%
Kilburn (2016, m)	+	Welf. policy (£)	CES-D	KEN	Low	0.282	[0.120; 0.444]	2.3%
Fernald	+	Welf. policy (£)	CES-D	ECU	Some conc.	-0.065	[-0.207; 0.077]	3.0%
Haushofer	+	Welf. policy (£)	CES-D	KEN	Some conc.	0.125	[0.032; 0.217]	3.5%
Baird (a)	+	Welf. policy (£)	GHQ	MWI	Some conc.	-0.009	[-0.168; 0.150]	2.1%
Baird (b)	+	Welf. policy (£)	GHQ	MWI	Some conc.	0.154	[0.049; 0.260]	2.8%
Courtin	+	Welf. policy (£)	Kessler	USA	Some conc.	0.106	[-0.085; 0.298]	2.4%
Hjelm (CGP)	+	Welf. policy (£)	PSS	ZMB	Some conc.	-0.017	[-0.172; 0.139]	2.3%
Hjelm (MCP)	+	Welf. policy (£)	PSS	ZMB	Some conc.	0.089	[-0.060; 0.238]	2.3%
Green	+	Welf. policy (£+)	APAI	UGA	Low	0.057	[-0.054; 0.168]	3.3%
Bedoya (f)	+	Welf. policy (£+)	CES-D	AFG	Some conc.	0.504	[0.388; 0.620]	2.7%
Bedoya (m)	+	Welf. policy (£+)	CES-D	AFG	Some conc.	0.198	[0.045; 0.351]	2.2%
Gassman-Pines	+	Welf. policy (£+)	CES-D	USA	Some conc.	0.208	[-0.027; 0.444]	2.0%
Gennetian	+	Welf. policy (£+)	CES-D	USA	Some conc.	0.129	[-0.033; 0.290]	2.7%
Macours	+	Welf. policy (£+)	CES-D	NIC	Some conc.	0.605	[-0.863; 2.073]	0.1%
Raschke (low)	.	Lottery win	SF-MCS	DEU	Serious	-0.264	[-0.467; -0.062]	2.1%
Boyd-Swan	.	Tax/wage policy	CES-D	USA	Serious	0.077	[-0.035; 0.189]	3.3%
Burmaster	.	Tax/wage policy	CES-D	DOM	Serious	0.466	[0.198; 0.733]	1.7%
Reeves (2017)	.	Tax/wage policy	GHQ	GBR	Serious	0.534	[0.178; 0.889]	1.2%
Koltai	.	Unknown	Kessler	CAN	Moderate	0.017	[-0.049; 0.083]	3.8%
Tachibana	.	Other transfer	CES-D	NPL	Serious	0.018	[0.003; 0.033]	4.1%
AIR	.	Welf. policy (£)	CES-D	ZWE	Low	-0.135	[-0.408; 0.138]	1.7%
Ozer	.	Welf. policy (£)	CES-D	MEX	Moderate	0.168	[0.094; 0.241]	3.7%
Gros	.	Welf. policy (£)	Subj. MH	BGD	Serious	0.332	[0.088; 0.577]	1.9%
**Random effects model**						**0.120**	**[0.049; 0.192]**	**59.4%**
Heterogeneity: I^2 = 84%								
**SEP: High**								
Raschke (high)	.	Lottery win	SF-MCS	DEU	Serious	-0.012	[-0.326; 0.303]	1.2%
**Random effects model**						**-0.012**	**[-0.326; 0.303]**	**1.2%**
Heterogeneity: not applicable								
**Random effects model**						**0.084**	**[0.038; 0.130]**	**100.0%**
Heterogeneity: I^2 = 80%								
Test for overall effect: $p < 0.001$								
Test for SG diffs: $\chi^2_2 = 7.23$, df = 2 ($p = 0.03$)								

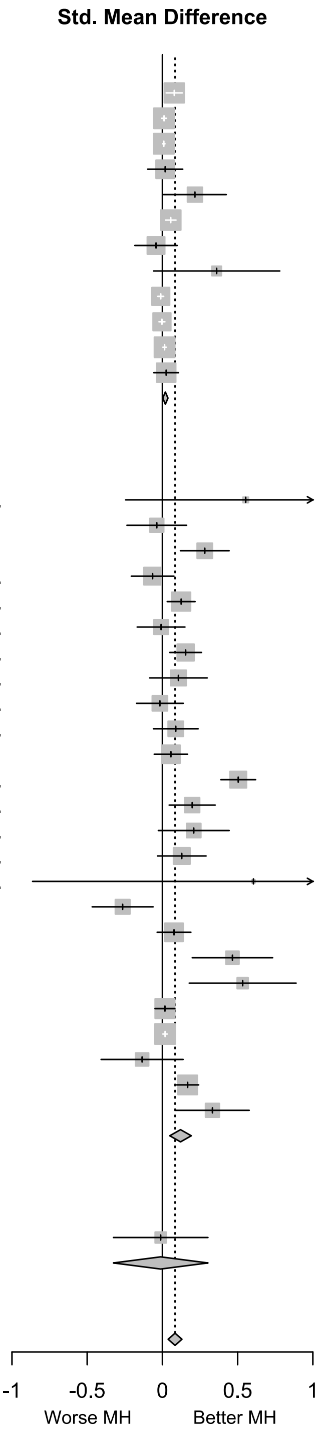

Number of people = 258,637; number of observations = 1,756,078. Studies sorted by RCT/NRS status → income source → outcome → RoB. + indicates study was a randomised controlled trial. (£) indicates a welfare policy which only affected income; (£+) indicates a welfare policy which also influenced other factors. (f) and (m) indicate studies which stratified results by sex; (high) and (low) indicate Raschke et al. stratified results by educational attainment. Baird (a) reported on a cash transfer to school dropouts versus Baird (b) which reported on a cash transfer to girls still enrolled in school; Hjelm (CGP) reported on the Zambia Child Grant Program versus Hjelm (MCP) which reported on the Zambia Multiple Category Cash Transfer Program. RoB = Risk of Bias score; SG = subgroup. APAI = Acholi Psychosocial Assessment Instrument. Beck = Beck depression inventory. CAPA = Child and Adolescent Psychiatric Assessment. CES-D = Center for Epidemiological Studies-Depression scale. CGI = Clinical Global Impression scale. GHQ = General Health Questionnaire. HDL = Health and Daily Living Form. Inc. mood dis. = Incident mood disorder (assessed at clinical interview). Kessler = Kessler Psychological Distress scale.

Mat. dep = measure of maternal depression. MH hosp. = mental health hospitalisations. PSS = Perceived Stress Scale. SF-MCS = Mental Component Summary of Short Form Survey. Subj. MH = measure of subjective/self-assessed mental health.

Figure G8: Forest plot for meta-analysis of studies reporting the effect of a binary income increase on a mental health outcome, stratified by setting

Source	RCT	Inc. source	Outcome	Country	RoB	SMD	95% CI	Weight
**Setting: High income**								
Priebe	+	Other transfer	CGI scale	GBR	Some conc.	0.554	[-0.245; 1.352]	0.3%
Courtin	+	Welf. policy (£)	Kessler	USA	Some conc.	0.106	[-0.085; 0.298]	2.4%
Gassman-Pines	+	Welf. policy (£+)	CES-D	USA	Some conc.	0.208	[-0.027; 0.444]	2.0%
Gennetian	+	Welf. policy (£+)	CES-D	USA	Some conc.	0.129	[-0.033; 0.290]	2.7%
Apouey	.	Lottery win	GHQ	GBR	Serious	0.079	[0.025; 0.132]	3.9%
Raschke (high)	.	Lottery win	SF-MCS	DEU	Serious	-0.012	[-0.326; 0.303]	1.2%
Raschke (low)	.	Lottery win	SF-MCS	DEU	Serious	-0.264	[-0.467; -0.062]	2.1%
Erixson	.	Tax/wage policy	MH hosp.	SWE	Low	0.011	[-0.006; 0.028]	4.1%
Boyd-Swan	.	Tax/wage policy	CES-D	USA	Serious	0.077	[-0.035; 0.189]	3.3%
Reeves (2017)	.	Tax/wage policy	GHQ	GBR	Serious	0.534	[0.178; 0.889]	1.2%
Evans (2010)	.	Tax/wage policy	Subj. MH	USA	Serious	0.009	[0.001; 0.017]	4.1%
Koltai	.	Unknown	Kessler	CAN	Moderate	0.017	[-0.049; 0.083]	3.8%
McCarthy	.	Unknown	Beck	CAN/USA	Serious	0.018	[-0.098; 0.135]	3.3%
Dearing	.	Unknown	CES-D	USA	Serious	0.216	[0.008; 0.424]	2.2%
Lorant	.	Unknown	HDL	BEL	Serious	0.055	[0.020; 0.090]	4.0%
Sareen	.	Unknown	Inc. mood dis.	USA	Serious	-0.042	[-0.183; 0.098]	3.0%
Costello	.	Other transfer	CAPA	USA	Moderate	0.361	[-0.059; 0.780]	0.9%
Ong (f)	.	Other transfer	SF-MCS	AUS	Moderate	-0.011	[-0.031; 0.009]	3.1%
Ong (m)	.	Other transfer	SF-MCS	AUS	Moderate	-0.003	[-0.023; 0.017]	3.1%
Lebihan	.	Welf. policy (£)	Mat. dep	CAN	Moderate	0.025	[-0.057; 0.107]	3.6%
**Random effects model**						**0.030**	**[0.007; 0.053]**	**54.3%**
Heterogeneity: $I^2 = 62\%$								
**Setting: Low/middle income**								
Kilburn (2016, f)	+	Welf. policy (£)	CES-D	KEN	Low	-0.037	[-0.236; 0.161]	1.9%
Kilburn (2016, m)	+	Welf. policy (£)	CES-D	KEN	Low	0.282	[0.120; 0.444]	2.3%
Fernald	+	Welf. policy (£)	CES-D	ECU	Some conc.	-0.065	[-0.207; 0.077]	3.0%
Haushofer	+	Welf. policy (£)	CES-D	KEN	Some conc.	0.125	[0.032; 0.217]	3.5%
Baird (a)	+	Welf. policy (£)	GHQ	MWI	Some conc.	-0.009	[-0.168; 0.150]	2.1%
Baird (b)	+	Welf. policy (£)	GHQ	MWI	Some conc.	0.154	[0.049; 0.260]	2.8%
Hjelm (CGP)	+	Welf. policy (£)	PSS	ZMB	Some conc.	-0.017	[-0.172; 0.139]	2.3%
Hjelm (MCP)	+	Welf. policy (£)	PSS	ZMB	Some conc.	0.089	[-0.060; 0.238]	2.3%
Green	+	Welf. policy (£+)	APAI	UGA	Low	0.057	[-0.054; 0.168]	3.3%
Bedoya (f)	+	Welf. policy (£+)	CES-D	AFG	Some conc.	0.504	[0.388; 0.620]	2.7%
Bedoya (m)	+	Welf. policy (£+)	CES-D	AFG	Some conc.	0.198	[0.045; 0.351]	2.2%
Macours	+	Welf. policy (£+)	CES-D	NIC	Some conc.	0.605	[-0.863; 2.073]	0.1%
Burmaster	.	Tax/wage policy	CES-D	DOM	Serious	0.466	[0.198; 0.733]	1.7%
Tachibana	.	Other transfer	CES-D	NPL	Serious	0.018	[0.003; 0.033]	4.1%
AIR	.	Welf. policy (£)	CES-D	ZWE	Low	-0.135	[-0.408; 0.138]	1.7%
Ozer	.	Welf. policy (£)	CES-D	MEX	Moderate	0.168	[0.094; 0.241]	3.7%
Powell-Jackson	.	Welf. policy (£)	Kessler	IND	Moderate	0.015	[0.003; 0.026]	4.1%
Gros	.	Welf. policy (£)	Subj. MH	BGD	Serious	0.332	[0.088; 0.577]	1.9%
**Random effects model**						**0.122**	**[0.041; 0.203]**	**45.7%**
Heterogeneity: $I^2 = 87\%$								
**Random effects model**						**0.084**	**[0.038; 0.130]**	**100.0%**

Heterogeneity: $I^2 = 80\%$
Test for overall effect: $p < 0.001$
Test for SG diffs: $\chi^2_1 = 4.56$, df = 1 ($p = 0.03$)

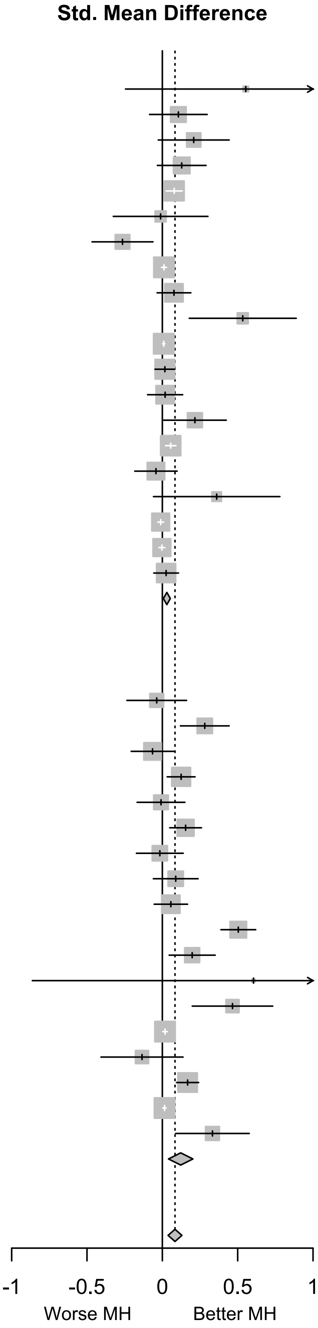

Number of people = 258,637; number of observations = 1,756,078. Studies sorted by RCT/NRS status → income source → outcome → RoB. + indicates study was a randomised controlled trial. (£) indicates a welfare policy which only affected income; (£+) indicates a welfare policy which also influenced other factors. (f) and (m) indicate studies which stratified results by sex; (high) and (low) indicate Raschke et al. stratified results by educational attainment. Baird (a) reported on a cash transfer to school dropouts versus Baird (b) which reported on a cash transfer to girls still enrolled in school; Hjelm (CGP) reported on the Zambia Child Grant Program versus Hjelm (MCP) which reported on the Zambia Multiple Category Cash Transfer Program. RoB = Risk of Bias score; SG = subgroup. APAI = Acholi Psychosocial Assessment Instrument. Beck = Beck depression inventory. CAPA = Child and Adolescent Psychiatric Assessment. CES-D = Center for Epidemiological Studies-Depression scale. CGI = Clinical Global Impression scale. GHQ = General Health Questionnaire. HDL = Health and Daily Living Form. Inc. mood dis. = Incident mood disorder (assessed at clinical interview). Kessler = Kessler Psychological Distress scale.

Mat. dep = measure of maternal depression. MH hosp. = mental health hospitalisations. PSS = Perceived Stress Scale. SF-MCS = Mental Component Summary of Short Form Survey. Subj. MH = measure of subjective/self-assessed mental health.

Figure G9: Forest plot for meta-analysis of studies reporting the effect of a binary income increase on a mental health outcome, stratified by age

Source	RCT	Inc. source	Outcome	Country	RoB	SMD	95% CI	Weight
**Age: Working age only**								
Priebe	+	Other transfer	CGI scale	GBR	Some conc.	0.554	[-0.245; 1.352]	0.3%
Fernald	+	Welf. policy (£)	CES-D	ECU	Some conc.	-0.065	[-0.207; 0.077]	3.0%
Haushofer	+	Welf. policy (£)	CES-D	KEN	Some conc.	0.125	[0.032; 0.217]	3.5%
Courtin	+	Welf. policy (£)	Kessler	USA	Some conc.	0.106	[-0.085; 0.298]	2.4%
Hjelm (CGP)	+	Welf. policy (£)	PSS	ZMB	Some conc.	-0.017	[-0.172; 0.139]	2.3%
Green	+	Welf. policy (£+)	APAI	UGA	Low	0.057	[-0.054; 0.168]	3.3%
Bedoya (f)	+	Welf. policy (£+)	CES-D	AFG	Some conc.	0.504	[0.388; 0.620]	2.7%
Bedoya (m)	+	Welf. policy (£+)	CES-D	AFG	Some conc.	0.198	[0.045; 0.351]	2.2%
Gassman-Pines	+	Welf. policy (£+)	CES-D	USA	Some conc.	0.208	[-0.027; 0.444]	2.0%
Gennetian	+	Welf. policy (£+)	CES-D	USA	Some conc.	0.129	[-0.033; 0.290]	2.7%
Macours	+	Welf. policy (£+)	CES-D	NIC	Some conc.	0.605	[-0.863; 2.073]	0.1%
Boyd-Swan	.	Tax/wage policy	CES-D	USA	Serious	0.077	[-0.035; 0.189]	3.3%
Burmaster	.	Tax/wage policy	CES-D	DOM	Serious	0.466	[0.198; 0.733]	1.7%
Reeves (2017)	.	Tax/wage policy	GHQ	GBR	Serious	0.534	[0.178; 0.889]	1.2%
Evans (2010)	.	Tax/wage policy	Subj. MH	USA	Serious	0.009	[0.001; 0.017]	4.1%
Koltai	.	Unknown	Kessler	CAN	Moderate	0.017	[-0.049; 0.083]	3.8%
McCarthy	.	Unknown	Beck	CAN/USA	Serious	0.018	[-0.098; 0.135]	3.3%
Dearing	.	Unknown	CES-D	USA	Serious	0.216	[0.008; 0.424]	2.2%
Costello	.	Other transfer	CAPA	USA	Moderate	0.361	[-0.059; 0.780]	0.9%
Ong (f)	.	Other transfer	SF-MCS	AUS	Moderate	-0.011	[-0.031; 0.009]	3.1%
Ong (m)	.	Other transfer	SF-MCS	AUS	Moderate	-0.003	[-0.023; 0.017]	3.1%
Ozer	.	Welf. policy (£)	CES-D	MEX	Moderate	0.168	[0.094; 0.241]	3.7%
Powell-Jackson	.	Welf. policy (£)	Kessler	IND	Moderate	0.015	[0.003; 0.026]	4.1%
Lebihan	.	Welf. policy (£)	Mat. dep	CAN	Moderate	0.025	[-0.057; 0.107]	3.6%
Gros	.	Welf. policy (£)	Subj. MH	BGD	Serious	0.332	[0.088; 0.577]	1.9%
**Random effects model**						**0.118**	**[0.056; 0.181]**	**64.5%**
Heterogeneity: I^2 = 84%								
**Age: Includes <16s or >64s**								
Kilburn (2016, f)	+	Welf. policy (£)	CES-D	KEN	Low	-0.037	[-0.236; 0.161]	1.9%
Kilburn (2016, m)	+	Welf. policy (£)	CES-D	KEN	Low	0.282	[0.120; 0.444]	2.3%
Baird (a)	+	Welf. policy (£)	GHQ	MWI	Some conc.	-0.009	[-0.168; 0.150]	2.1%
Baird (b)	+	Welf. policy (£)	GHQ	MWI	Some conc.	0.154	[0.049; 0.260]	2.8%
Hjelm (MCP)	+	Welf. policy (£)	PSS	ZMB	Some conc.	0.089	[-0.060; 0.238]	2.3%
Apouey	.	Lottery win	GHQ	GBR	Serious	0.079	[0.025; 0.132]	3.9%
Raschke (high)	.	Lottery win	SF-MCS	DEU	Serious	-0.012	[-0.326; 0.303]	1.2%
Raschke (low)	.	Lottery win	SF-MCS	DEU	Serious	-0.264	[-0.467; -0.062]	2.1%
Erixson	.	Tax/wage policy	MH hosp.	SWE	Low	0.011	[-0.006; 0.028]	4.1%
Lorant	.	Unknown	HDL	BEL	Serious	0.055	[0.020; 0.090]	4.0%
Sareen	.	Unknown	Inc. mood dis.	USA	Serious	-0.042	[-0.183; 0.098]	3.0%
Tachibana	.	Other transfer	CES-D	NPL	Serious	0.018	[0.003; 0.033]	4.1%
AIR	.	Welf. policy (£)	CES-D	ZWE	Low	-0.135	[-0.408; 0.138]	1.7%
**Random effects model**						**0.035**	**[-0.016; 0.087]**	**35.5%**
Heterogeneity: I^2 = 67%								
**Random effects model**						**0.084**	**[0.038; 0.130]**	**100.0%**
Heterogeneity: I^2 = 80%								
Test for overall effect: p < 0.001								
Test for SG diffs: χ^2_1 = 4.05, df = 1 (p = 0.04)								

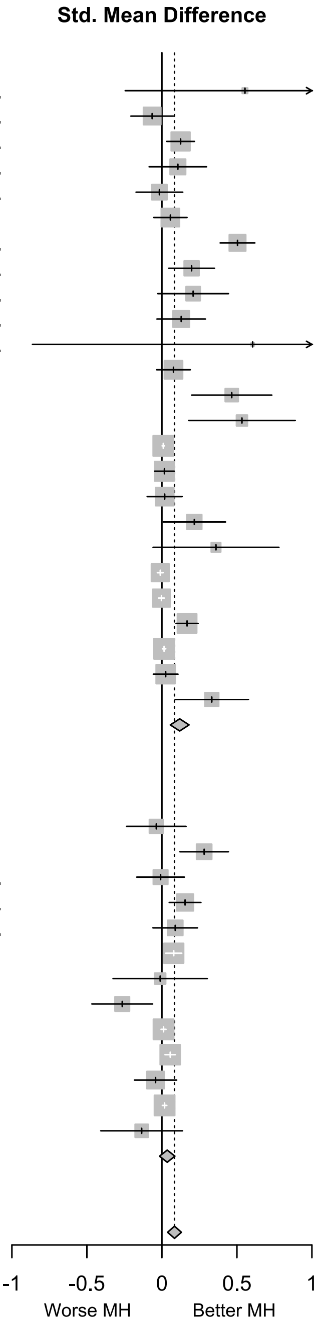

Number of people = 258,637; number of observations = 1,756,078. Studies sorted by RCT/NRS status → income source → outcome → RoB. + indicates study was a randomised controlled trial. (£) indicates a welfare policy which only affected income; (£+) indicates a welfare policy which also influenced other factors. (f) and (m) indicate studies which stratified results by sex; (high) and (low) indicate Raschke et al. stratified results by educational attainment. Baird (a) reported on a cash transfer to school dropouts versus Baird (b) which reported on a cash transfer to girls still enrolled in school; Hjelm (CGP) reported on the Zambia Child Grant Program versus Hjelm (MCP) which reported on the Zambia Multiple Category Cash Transfer Program. RoB = Risk of Bias score; SG = subgroup. APAI = Acholi Psychosocial Assessment Instrument. Beck = Beck depression inventory. CAPA = Child and Adolescent Psychiatric Assessment. CES-D = Center for Epidemiological Studies-Depression scale. CGI = Clinical Global Impression scale. GHQ = General Health Questionnaire. HDL = Health and Daily Living Form. Inc. mood dis. = Incident mood disorder (assessed at clinical interview). Kessler = Kessler Psychological Distress scale.

Mat. dep = measure of maternal depression. MH hosp. = mental health hospitalisations. PSS = Perceived Stress Scale. SF-MCS = Mental Component Summary of Short Form Survey. Subj. MH = measure of subjective/self-assessed mental health.

Figure G10: Forest plot for meta-analysis of studies reporting the effect of a binary income decrease on a mental health outcome, stratified by sex

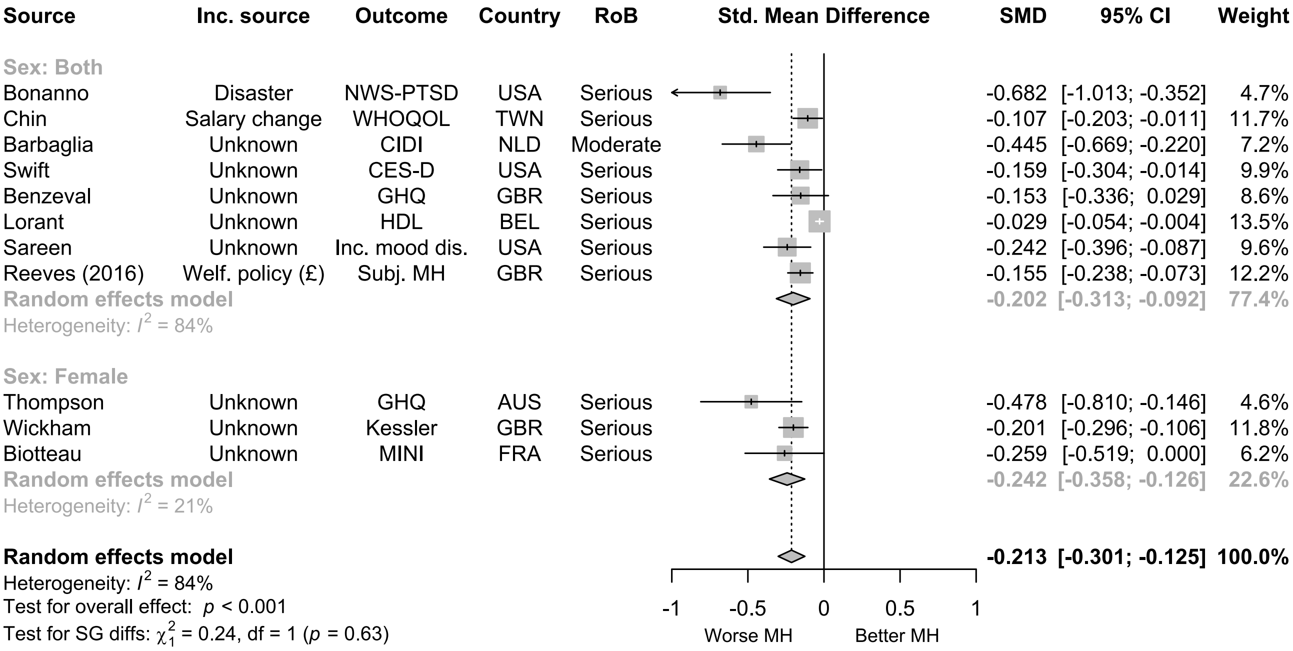

Number of people = 227,804; number of observations = 281,728. Studies sorted by income source → outcome → RoB. (£) indicates a welfare policy which only affected income. RoB = Risk of Bias score; SG = subgroup. CES-D = Center for Epidemiological Studies-Depression scale. CIDI = Composite International Diagnostic Interview. GHQ = General Health Questionnaire. HDL = Health and Daily Living Form. Inc. mood dis. = Incident mood disorder (assessed at clinical interview). Kessler = Kessler Psychological Distress scale. MINI = Mini International Neuropsychiatric Interview. NWS-PTSD = National Women's Study PTSD module. Subj. MH = measure of subjective/self-assessed mental health. WHOQOL = Psychological domain of abbreviated World Health Organisation Quality of Life tool

Figure G11: Forest plot for meta-analysis of studies reporting the effect of a binary income decrease on a mental health outcome, stratified by risk of bias

Source	Inc. source	Outcome	Country	RoB	SMD	95% CI	Weight
**Risk of Bias: Low/Moderate**							
Barbaglia	Unknown	CIDI	NLD	Moderate	-0.445	[-0.669; -0.220]	4.2%
**Random effects model**					**-0.445**	**[-0.669; -0.220]**	**4.2%**
Heterogeneity: not applicable							
**Risk of Bias: Serious**							
Bonanno	Disaster	NWS-PTSD	USA	Serious	-0.682	[-1.013; -0.352]	2.9%
Chin	Salary change	WHOQOL	TWN	Serious	-0.107	[-0.203; -0.011]	6.3%
Swift	Unknown	CES-D	USA	Serious	-0.159	[-0.304; -0.014]	5.5%
Benzeval	Unknown	GHQ	GBR	Serious	-0.153	[-0.336; 0.029]	4.9%
Thompson	Unknown	GHQ	AUS	Serious	-0.478	[-0.810; -0.146]	2.9%
Lorant	Unknown	HDL	BEL	Serious	-0.029	[-0.054; -0.004]	7.0%
Sareen	Unknown	Inc. mood dis.	USA	Serious	-0.242	[-0.396; -0.087]	5.4%
Wickham	Unknown	Kessler	GBR	Serious	-0.201	[-0.296; -0.106]	6.3%
Biotteau	Unknown	MINI	FRA	Serious	-0.259	[-0.519; 0.000]	3.7%
Reeves (2016)	Welf. policy (£)	Subj. MH	GBR	Serious	-0.155	[-0.238; -0.073]	6.5%
**Random effects model**					**-0.185**	**[-0.264; -0.107]**	**51.3%**
Heterogeneity: $I^2 = 82\%$							
**Risk of Bias: Critical**							
Ishiguro (f)	Disaster	Kessler	JPN	Critical	-0.382	[-0.573; -0.191]	4.7%
Ishiguro (m)	Disaster	Kessler	JPN	Critical	-0.293	[-0.566; -0.019]	3.4%
Buttke (B)	Disaster	PHQ-2	USA	Critical	-0.113	[-0.412; 0.186]	3.1%
Buttke (Mi)	Disaster	PHQ-2	USA	Critical	-0.589	[-0.897; -0.281]	3.0%
Buttke (Mo)	Disaster	PHQ-2	USA	Critical	-0.607	[-0.894; -0.320]	3.2%
Chien	Disaster	SF-MCS	TWN	Critical	-0.109	[-0.218; 0.000]	6.1%
Abel	Illness/caring	PSS	USA	Critical	-0.401	[-0.666; -0.136]	3.7%
Brenner	Salary change	SCL dep.	FRA/HUN/SWE/GBR	Critical	-0.305	[-0.549; -0.062]	4.0%
Bolden	Unknown	CES-D	USA	Critical	-0.649	[-1.185; -0.113]	1.5%
Araya	Unknown	CIS-R	CHL	Critical	-0.419	[-0.547; -0.292]	5.8%
Robert	Unknown	GHQ	ESP	Critical	-0.558	[-1.073; -0.043]	1.6%
Casey	Welf. policy (£)	Mat. dep	USA	Critical	-0.231	[-0.446; -0.015]	4.4%
**Random effects model**					**-0.346**	**[-0.449; -0.244]**	**44.4%**
Heterogeneity: $I^2 = 62\%$							
**Random effects model**					**-0.278**	**[-0.351; -0.205]**	**100.0%**

Heterogeneity: $I^2 = 84\%$
Test for overall effect: $p < 0.001$
Test for SG diffs: $\chi^2_2 = 8.81$, df = 2 ($p = 0.01$)

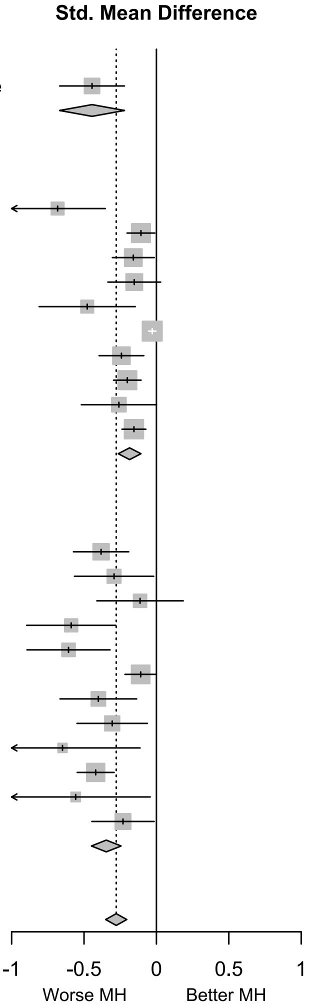

Number of people = 235,154; number of observations = 289,078. Studies sorted by income source → outcome → RoB. (£) indicates a welfare policy which only affected income. RoB = Risk of Bias score; SG = subgroup. CES-D = Center for Epidemiological Studies-Depression scale. CIDI = Composite International Diagnostic Interview. CIS-R = Revised Clinical Interview Schedule. GHQ = General Health Questionnaire. HDL = Health and Daily Living Form. Inc. mood dis. = Incident mood disorder (assessed at clinical interview). Kessler = Kessler Psychological Distress scale. Mat. dep = measure of maternal depression. MINI = Mini International Neuropsychiatric Interview. NWS-PTSD = National Women's Study PTSD module. PHQ-2 = Patient Health Questionnaire-2. PSS = Perceived Stress Scale. SCL dep. = Symptom Checklist 90-Revised, depression subscale. SF-MCS = Mental Component Summary of Short Form Survey. Subj. MH = measure of subjective/self-assessed mental health. WHOQOL = Psychological domain of abbreviated World Health Organisation Quality of Life tool

Figure G12: Forest plot for meta-analysis of studies reporting the effect of a binary income decrease on a mental health outcome, stratified by age

Source	Inc. source	Outcome	Country	RoB	SMD	95% CI	Weight
**Age: Working age only**							
Barbaglia	Unknown	CIDI	NLD	Moderate	-0.445	[-0.669; -0.220]	7.2%
Swift	Unknown	CES-D	USA	Serious	-0.159	[-0.304; -0.014]	9.9%
Wickham	Unknown	Kessler	GBR	Serious	-0.201	[-0.296; -0.106]	11.8%
**Random effects model**					**-0.241**	**[-0.378; -0.104]**	**28.9%**
Heterogeneity: $I^2 = 57\%$							
**Age: Includes <16s or >64s**							
Bonanno	Disaster	NWS-PTSD	USA	Serious	-0.682	[-1.013; -0.352]	4.7%
Chin	Salary change	WHOQOL	TWN	Serious	-0.107	[-0.203; -0.011]	11.7%
Benzeval	Unknown	GHQ	GBR	Serious	-0.153	[-0.336; 0.029]	8.6%
Thompson	Unknown	GHQ	AUS	Serious	-0.478	[-0.810; -0.146]	4.6%
Lorant	Unknown	HDL	BEL	Serious	-0.029	[-0.054; -0.004]	13.5%
Sareen	Unknown	Inc. mood dis.	USA	Serious	-0.242	[-0.396; -0.087]	9.6%
Biotteau	Unknown	MINI	FRA	Serious	-0.259	[-0.519; 0.000]	6.2%
Reeves (2016)	Welf. policy (£)	Subj. MH	GBR	Serious	-0.155	[-0.238; -0.073]	12.2%
**Random effects model**					**-0.208**	**[-0.324; -0.092]**	**71.1%**
Heterogeneity: $I^2 = 83\%$							
**Random effects model**					**-0.213**	**[-0.301; -0.125]**	**100.0%**
Heterogeneity: $I^2 = 84\%$							
Test for overall effect: $p < 0.001$							
Test for SG diffs: $\chi^2_1 = 0.13$, df = 1 ($p = 0.72$)							

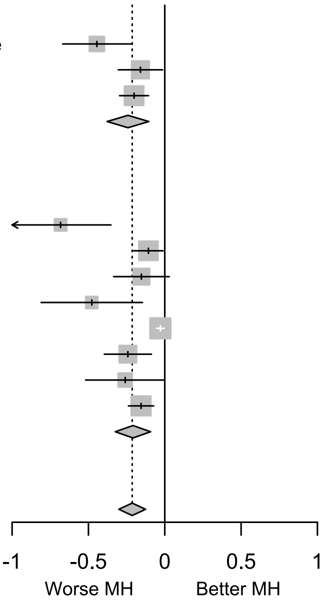

Number of people = 227,804; number of observations = 281,728. Studies sorted by income source → outcome → RoB. (£) indicates a welfare policy which only affected income. RoB = Risk of Bias score; SG = subgroup. CES-D = Center for Epidemiological Studies-Depression scale. CIDI = Composite International Diagnostic Interview. GHQ = General Health Questionnaire. HDL = Health and Daily Living Form. Inc. mood dis. = Incident mood disorder (assessed at clinical interview). Kessler = Kessler Psychological Distress scale. MINI = Mini International Neuropsychiatric Interview. NWS-PTSD = National Women's Study PTSD module. Subj. MH = measure of subjective/self-assessed mental health. WHOQOL = Psychological domain of abbreviated World Health Organisation Quality of Life tool

Figure G13: Forest plot for meta-analysis of studies reporting the effect of a continuous log(income) change on a mental health outcome, stratified by sex

Source	Inc. source	Outcome	Country	RoB	Std. ß	95% CI	Weight
**Sex: Both**							
Apouey	Lottery win	GHQ	GBR	Serious	0.013	[0.002; 0.023]	15.2%
Erixson	Tax/wage policy	MH hosp.	SWE	Low	0.001	[-0.001; 0.002]	15.6%
Allouche	Unknown	CES-D	ZAF	Serious	0.428	[0.098; 0.758]	0.5%
Lorant	Unknown	HDL	BEL	Serious	0.006	[-0.013; 0.025]	14.2%
Clingingsmith	Unknown	Kessler	USA	Serious	0.038	[0.018; 0.057]	14.2%
McKenzie	Unknown	Kessler	NZL	Serious	-0.013	[-0.025; 0.000]	15.0%
**Random effects model**					**0.009**	**[-0.007; 0.024]**	**74.7%**
Heterogeneity: $I^2 = 83\%$, $p < 0.01$							
**Sex: Male**							
Horn (m)	Tax/wage policy	Subj. MH	USA	Serious	-0.006	[-0.121; 0.109]	2.6%
BlazquezCuesta (m)	Unknown	SF-MCS	DEU	Serious	0.080	[-0.002; 0.161]	4.3%
**Random effects model**					**0.047**	**[-0.035; 0.129]**	**6.9%**
Heterogeneity: $I^2 = 30\%$, $p = 0.23$							
**Sex: Female**							
Horn (f)	Tax/wage policy	Subj. MH	USA	Serious	0.088	[-0.001; 0.176]	4.3%
Kiernan	Unknown	CES-D	IRL	Serious	0.054	[0.008; 0.099]	10.2%
BlazquezCuesta (f)	Unknown	SF-MCS	DEU	Serious	0.153	[0.068; 0.238]	4.0%
**Random effects model**					**0.090**	**[0.031; 0.149]**	**18.4%**
Heterogeneity: $I^2 = 52\%$, $p = 0.12$							
**Random effects model**					**0.027**	**[0.003; 0.052]**	**100.0%**
Heterogeneity: $I^2 = 81\%$, $p < 0.01$							
Test for overall effect: $p = 0.030$							
Test for SG diffs: $\chi^2_2 = 7.43$, df = 2 ($p = 0.02$)							

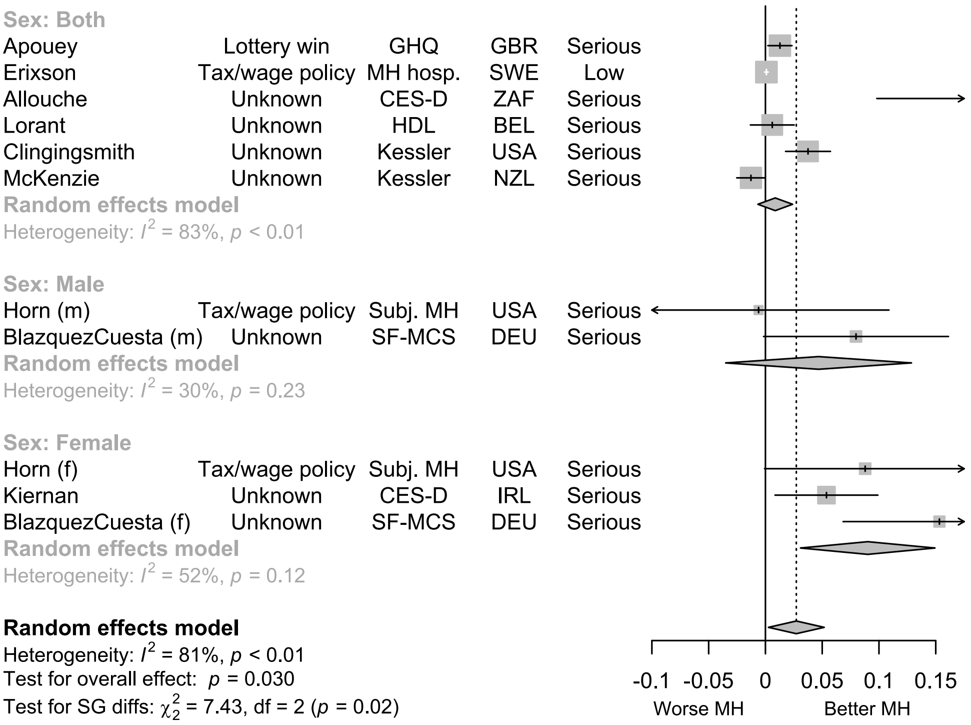

Number of people = 1,510,221; number of observations = 3,036,715. Studies sorted by income source → outcome → RoB. (f) and (m) indicate studies stratified results by sex. RoB = Risk of Bias score; SG = subgroup. CES-D = Center for Epidemiological Studies-Depression scale. GHQ = General Health Questionnaire. HDL = Health and Daily Living Form. Kessler = Kessler Psychological Distress scale. MH hosp. = mental health hospitalisations. Subj. MH = measure of subjective/self-assessed mental health. SF-MCS = Mental Component Summary of Short Form Survey.

Figure G14: Forest plot for meta-analysis of studies reporting the effect of a continuous log(income) change on a mental health outcome, stratified by socioeconomic position

Source	Inc. source	Outcome	Country	RoB	Std. ß	95% CI	Weight
**SEP: Mixed**							
Apouey	Lottery win	GHQ	GBR	Serious	0.013	[0.002; 0.023]	15.3%
Erixson	Tax/wage policy	MH hosp.	SWE	Low	0.001	[-0.001; 0.002]	15.8%
Kiernan	Unknown	CES-D	IRL	Serious	0.054	[0.008; 0.099]	10.1%
Lorant	Unknown	HDL	BEL	Serious	0.006	[-0.013; 0.025]	14.4%
Clingingsmith	Unknown	Kessler	USA	Serious	0.038	[0.018; 0.057]	14.3%
McKenzie	Unknown	Kessler	NZL	Serious	-0.013	[-0.025; 0.000]	15.2%
BlazquezCuesta (f)	Unknown	SF-MCS	DEU	Serious	0.153	[0.068; 0.238]	3.9%
BlazquezCuesta (m)	Unknown	SF-MCS	DEU	Serious	0.080	[-0.002; 0.161]	4.2%
**Random effects model**					**0.023**	**[-0.002; 0.048]**	**93.2%**
Heterogeneity: $I^2 = 84\%$, $p < 0.01$							
**SEP: Low**							
Horn (f)	Tax/wage policy	Subj. MH	USA	Serious	0.088	[-0.001; 0.176]	4.2%
Horn (m)	Tax/wage policy	Subj. MH	USA	Serious	-0.006	[-0.121; 0.109]	2.5%
Allouche (low)	Unknown	CES-D	ZAF	Serious	0.901	[0.223; 1.580]	0.1%
**Random effects model**					**0.405**	**[-0.420; 1.230]**	**6.8%**
Heterogeneity: $I^2 = 74\%$, $p = 0.02$							
**Random effects model**					**0.026**	**[0.002; 0.050]**	**100.0%**

Heterogeneity: $I^2 = 82\%$, $p < 0.01$
Test for overall effect: $p = 0.035$
Test for SG diffs: $\chi^2_1 = 0.82$, df = 1 ($p = 0.36$)

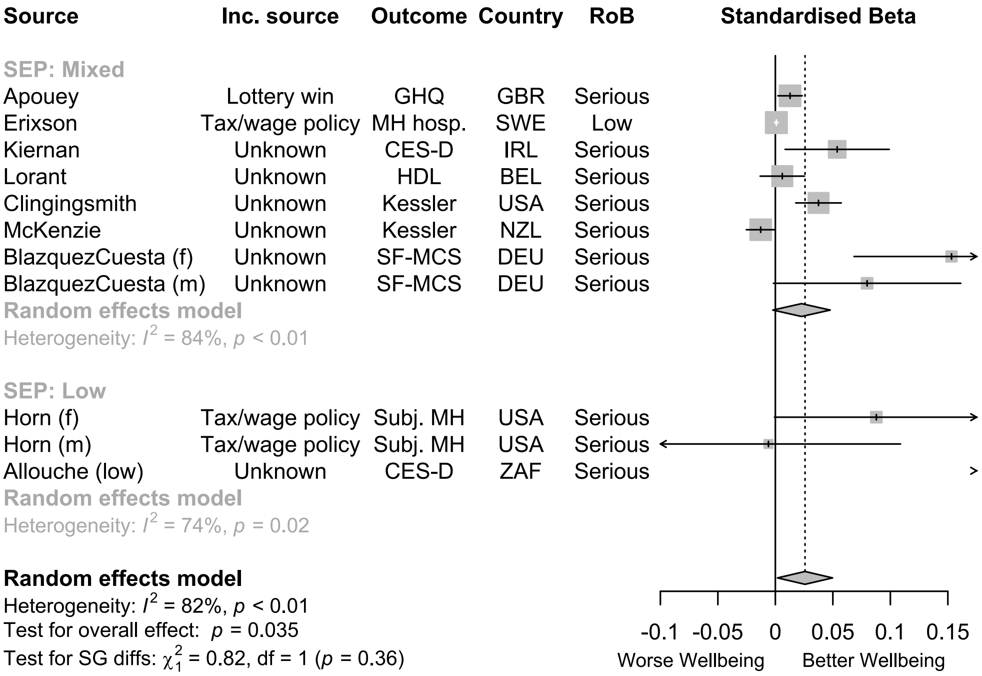

Number of people = 1,510,221; number of observations = 3,036,715. Studies sorted by income source → outcome → RoB. (f) and (m) indicate studies stratified results by sex. RoB = Risk of Bias score; SG = subgroup. CES-D = Center for Epidemiological Studies-Depression scale. GHQ = General Health Questionnaire. HDL = Health and Daily Living Form. Kessler = Kessler Psychological Distress scale. MH hosp. = mental health hospitalisations. Subj. MH = measure of subjective/self-assessed mental health. SF-MCS = Mental Component Summary of Short Form Survey.

Figure G15: Forest plot for meta-analysis of studies reporting the effect of a continuous log(income) change on a mental health outcome, stratified by age

Source	Inc. source	Outcome	Country	RoB	Std. ß	95% CI	Weight
**Age: Working age only**							
Horn (f)	Tax/wage policy	Subj. MH	USA	Serious	0.088	[-0.001; 0.176]	4.3%
Horn (m)	Tax/wage policy	Subj. MH	USA	Serious	-0.006	[-0.121; 0.109]	2.6%
Kiernan	Unknown	CES-D	IRL	Serious	0.054	[0.008; 0.099]	10.2%
Clingingsmith	Unknown	Kessler	USA	Serious	0.038	[0.018; 0.057]	14.2%
McKenzie	Unknown	Kessler	NZL	Serious	-0.013	[-0.025; 0.000]	15.0%
**Random effects model**					**0.027**	**[-0.008; 0.063]**	**46.2%**
Heterogeneity: $I^2 = 85\%$, $p < 0.01$							
**Age: Includes <16s or >64s**							
Apouey	Lottery win	GHQ	GBR	Serious	0.013	[0.002; 0.023]	15.2%
Erixson	Tax/wage policy	MH hosp.	SWE	Low	0.001	[-0.001; 0.002]	15.6%
Allouche	Unknown	CES-D	ZAF	Serious	0.428	[0.098; 0.758]	0.5%
Lorant	Unknown	HDL	BEL	Serious	0.006	[-0.013; 0.025]	14.2%
BlazquezCuesta (f)	Unknown	SF-MCS	DEU	Serious	0.153	[0.068; 0.238]	4.0%
BlazquezCuesta (m)	Unknown	SF-MCS	DEU	Serious	0.080	[-0.002; 0.161]	4.3%
**Random effects model**					**0.038**	**[-0.014; 0.089]**	**53.8%**
Heterogeneity: $I^2 = 82\%$, $p < 0.01$							
**Random effects model**					**0.027**	**[0.003; 0.052]**	**100.0%**

Heterogeneity: $I^2 = 81\%$, $p < 0.01$
Test for overall effect: $p = 0.030$
Test for SG diffs: $\chi^2_1 = 0.10$, df = 1 ($p = 0.75$)

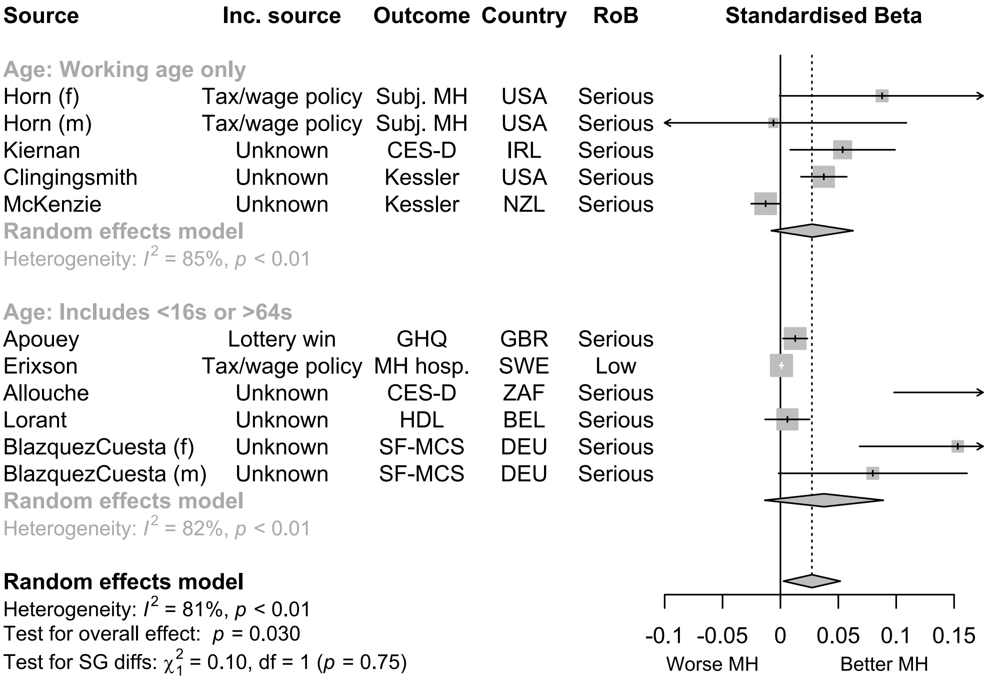

Number of people = 1,510,221; number of observations = 3,036,715. Studies sorted by income source → outcome → RoB. (f) and (m) indicate studies stratified results by sex. RoB = Risk of Bias score; SG = subgroup. CES-D = Center for Epidemiological Studies-Depression scale. GHQ = General Health Questionnaire. HDL = Health and Daily Living Form. Kessler = Kessler Psychological Distress scale. MH hosp. = mental health hospitalisations. Subj. MH = measure of subjective/self-assessed mental health. SF-MCS = Mental Component Summary of Short Form Survey.

Figure G16: Forest plot for meta-analysis of studies reporting the effect of a binary income increase on a wellbeing outcome, stratified by sex

Source	RCT	Inc. source	Outcome	Country	RoB	SMD	95% CI	Weight
**Sex: Both**								
Priebe	+	Other transfer	DIALOG QoL	GBR	High	0.710	[0.265; 1.155]	3.6%
Haushofer	+	Welf. policy (£)	Subj. LS	KEN	Some conc.	0.170	[0.072; 0.268]	6.0%
Kilburn (2018)	+	Welf. policy (£)	Subj. QoL	MWI	Some conc.	0.497	[0.252; 0.742]	5.1%
Apouey	.	Lottery win	Subj. LS	GBR	Serious	0.068	[0.027; 0.109]	6.2%
Gulal	.	Tax/wage policy	Subj. LS	DEU	Serious	0.061	[0.013; 0.108]	6.2%
Frijters (2011)	.	Unknown	Subj. LS	AUS	Serious	0.173	[0.110; 0.236]	6.1%
AIR	.	Welf. policy (£)	SWLS	ZWE	Low	0.253	[0.121; 0.384]	5.9%
Gros	.	Welf. policy (£)	Subj. hap	BGD	Serious	0.475	[0.238; 0.712]	5.2%
Galama	.	Welf. policy (£)	Subj. LS	COL	Serious	1.497	[1.116; 1.879]	4.1%
**Random effects model**						**0.402**	**[0.128; 0.676]**	**48.5%**
Heterogeneity: $I^2 = 92\%$								
**Sex: Male**								
Bedoya (m)	+	Welf. policy (£+)	Subj. LS	AFG	Some conc.	0.176	[0.023; 0.329]	2.1%
Lachowska (m)	.	Tax/wage policy	Subj. LS	USA	Serious	-0.114	[-1.221; 0.993]	0.9%
Clark (m)	.	Unknown	Subj. LS	DEU	Serious	0.067	[0.045; 0.088]	2.8%
Dang (m)	.	Unknown	Subj. LS	RUS	Serious	0.059	[0.042; 0.077]	3.1%
Handa (m)	.	Welf. policy (£+)	Subj. LS	GHA	Serious	0.093	[-0.252; 0.439]	3.4%
**Random effects model**						**0.063**	**[0.050; 0.077]**	**12.3%**
Heterogeneity: $I^2 = 0\%$								
**Sex: Female**								
Natali	+	Welf. policy (£)	Subj. hap	ZMB	Some conc.	0.347	[0.210; 0.483]	5.8%
Bedoya (f)	+	Welf. policy (£+)	Subj. LS	AFG	Some conc.	0.435	[0.323; 0.547]	3.9%
Dorsett	+	Welf. policy (£+)	Subj. LS	GBR	Some conc.	0.038	[-0.037; 0.114]	6.1%
Boyd-Swan	.	Tax/wage policy	Subj. hap	USA	Serious	0.071	[-0.060; 0.201]	5.9%
Lachowska (f)	.	Tax/wage policy	Subj. LS	USA	Serious	0.611	[-0.302; 1.524]	1.3%
Clark (f)	.	Unknown	Subj. LS	DEU	Serious	0.084	[0.066; 0.103]	3.4%
Dang (f)	.	Unknown	Subj. LS	RUS	Serious	0.077	[0.060; 0.095]	3.1%
Powell-Jackson	.	Welf. policy (£)	Subj. hap	IND	Moderate	0.120	[-0.092; 0.332]	5.4%
Maeder	.	Welf. policy (£)	Subj. LS	DEU	Serious	0.545	[-0.039; 1.129]	2.8%
Handa (f)	.	Welf. policy (£+)	Subj. LS	GHA	Serious	0.525	[-0.003; 1.053]	1.4%
**Random effects model**						**0.195**	**[0.080; 0.309]**	**39.2%**
Heterogeneity: $I^2 = 85\%$								
**Random effects model**						**0.277**	**[0.145; 0.408]**	**100.0%**

Heterogeneity: $I^2 = 87\%$
Test for overall effect: $p < 0.001$
Test for SG diffs: $\chi^2_2 = 10.78$, df = 2 ($p < 0.01$)

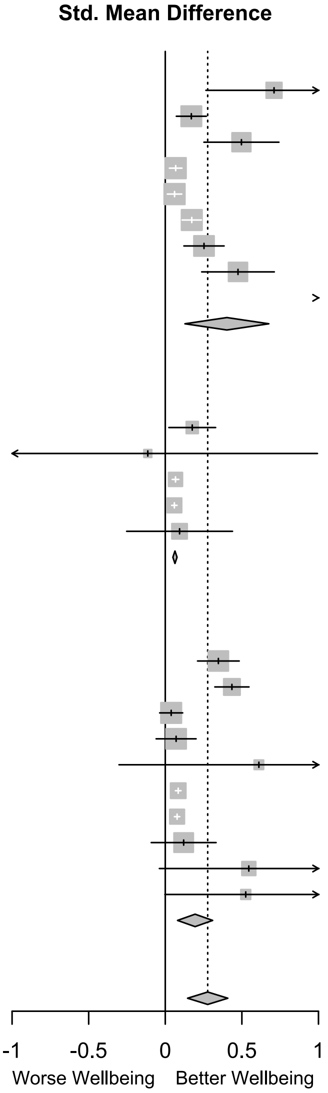

Number of people = 163,969; number of observations = 885,981. Studies sorted by RCT/NRS status → income source → outcome → RoB. + indicates study was a randomised controlled trial. (£) indicates a welfare policy which only affected income; (£+) indicates a welfare policy which also influenced other factors. (f) and (m) indicate studies which stratified results by sex. RoB = Risk of Bias score; SG = subgroup. AIR = American Institutes for Research. DIALOG QoL = DIALOG quality of life scale. Subj. hap = measure of subjective/self-assessed happiness. Subj. LS = measure of subjective/self-assessed life satisfaction. Subj. QoL = measure of subjective/self-assessed quality of life. SWLS = Diener's Satisfaction with Life Scale.

Figure G17: Forest plot for meta-analysis of studies reporting the effect of a binary income increase on a wellbeing outcome, stratified by study design

Source	RCT	Inc. source	Outcome	Country	RoB	SMD	95% CI	Weight
**Study Design: NRS**								
Apouey	.	Lottery win	Subj. LS	GBR	Serious	0.068	[0.027; 0.109]	6.2%
Boyd-Swan	.	Tax/wage policy	Subj. hap	USA	Serious	0.071	[-0.060; 0.201]	5.9%
Gulal	.	Tax/wage policy	Subj. LS	DEU	Serious	0.061	[0.013; 0.108]	6.2%
Lachowska (high)	.	Tax/wage policy	Subj. LS	USA	Serious	0.147	[-0.523; 0.817]	2.2%
Lachowska (low)	.	Tax/wage policy	Subj. LS	USA	Serious	0.667	[-1.152; 2.486]	0.3%
Clark (f)	.	Unknown	Subj. LS	DEU	Serious	0.084	[0.066; 0.103]	3.4%
Clark (m)	.	Unknown	Subj. LS	DEU	Serious	0.067	[0.045; 0.088]	2.8%
Dang (f)	.	Unknown	Subj. LS	RUS	Serious	0.077	[0.060; 0.095]	3.1%
Dang (m)	.	Unknown	Subj. LS	RUS	Serious	0.059	[0.042; 0.077]	3.1%
Frijters (2011)	.	Unknown	Subj. LS	AUS	Serious	0.173	[0.110; 0.236]	6.1%
AIR	.	Welf. policy (£)	SWLS	ZWE	Low	0.253	[0.121; 0.384]	5.9%
Powell-Jackson	.	Welf. policy (£)	Subj. hap	IND	Moderate	0.120	[-0.092; 0.332]	5.3%
Gros	.	Welf. policy (£)	Subj. hap	BGD	Serious	0.475	[0.238; 0.712]	5.2%
Galama	.	Welf. policy (£)	Subj. LS	COL	Serious	1.497	[1.116; 1.879]	4.1%
Maeder	.	Welf. policy (£)	Subj. LS	DEU	Serious	0.545	[-0.039; 1.129]	2.8%
Handa (f)	.	Welf. policy (£+)	Subj. LS	GHA	Serious	0.525	[-0.003; 1.053]	1.4%
Handa (m)	.	Welf. policy (£+)	Subj. LS	GHA	Serious	0.093	[-0.252; 0.439]	3.3%
**Random effects model**						**0.263**	**[0.074; 0.452]**	**67.3%**
Heterogeneity: I^2 = 83%								
**Study Design: RCT**								
Priebe	+	Other transfer	DIALOG QoL	GBR	High	0.710	[0.265; 1.155]	3.6%
Natali	+	Welf. policy (£)	Subj. hap	ZMB	Some conc.	0.347	[0.210; 0.483]	5.8%
Haushofer	+	Welf. policy (£)	Subj. LS	KEN	Some conc.	0.170	[0.072; 0.268]	6.0%
Kilburn (2018)	+	Welf. policy (£)	Subj. QoL	MWI	Some conc.	0.497	[0.252; 0.742]	5.1%
Bedoya (f)	+	Welf. policy (£+)	Subj. LS	AFG	Some conc.	0.435	[0.323; 0.547]	3.9%
Bedoya (m)	+	Welf. policy (£+)	Subj. LS	AFG	Some conc.	0.176	[0.023; 0.329]	2.1%
Dorsett	+	Welf. policy (£+)	Subj. LS	GBR	Some conc.	0.038	[-0.037; 0.114]	6.1%
**Random effects model**						**0.295**	**[0.149; 0.441]**	**32.7%**
Heterogeneity: I^2 = 88%								
**Random effects model**						**0.274**	**[0.143; 0.405]**	**100.0%**

Heterogeneity: I^2 = 87%
Test for overall effect: $p < 0.001$
Test for SG diffs: $\chi^2_1 = 0.07$, df = 1 (p = 0.79)

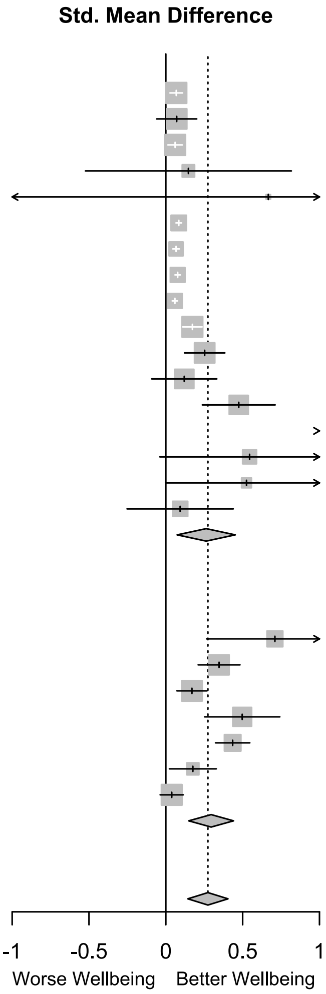

Number of people = 163,969; number of observations = 885,981. Studies sorted by RCT/NRS status → income source → outcome → RoB. + indicates study was a randomised controlled trial. (£) indicates a welfare policy which only affected income; (£+) indicates a welfare policy which also influenced other factors. (f) and (m) indicate studies which stratified results by sex. RoB = Risk of Bias score; SG = subgroup. AIR = American Institutes for Research. DIALOG QoL = DIALOG quality of life scale. Subj. hap = measure of subjective/self-assessed happiness. Subj. LS = measure of subjective/self-assessed life satisfaction. Subj. QoL = measure of subjective/self-assessed quality of life. SWLS = Diener's Satisfaction with Life Scale.

Figure G18: Forest plot for meta-analysis of studies reporting the effect of a binary income increase on a wellbeing outcome, stratified by risk of bias

Source	RCT	Inc. source	Outcome	Country	RoB	SMD	95% CI	Weight
**Risk of Bias: Low/Moderate**								
Natali	+	Welf. policy (£)	Subj. hap	ZMB	Some conc.	0.347	[0.210; 0.483]	5.4%
Haushofer	+	Welf. policy (£)	Subj. LS	KEN	Some conc.	0.170	[0.072; 0.268]	5.6%
Kilburn (2018)	+	Welf. policy (£)	Subj. QoL	MWI	Some conc.	0.497	[0.252; 0.742]	4.6%
Bedoya (f)	+	Welf. policy (£+)	Subj. LS	AFG	Some conc.	0.435	[0.323; 0.547]	3.6%
Bedoya (m)	+	Welf. policy (£+)	Subj. LS	AFG	Some conc.	0.176	[0.023; 0.329]	2.0%
Dorsett	+	Welf. policy (£+)	Subj. LS	GBR	Some conc.	0.038	[-0.037; 0.114]	5.7%
AIR	.	Welf. policy (£)	SWLS	ZWE	Low	0.253	[0.121; 0.384]	5.4%
Powell-Jackson	.	Welf. policy (£)	Subj. hap	IND	Moderate	0.120	[-0.092; 0.332]	4.9%
**Random effects model**						**0.245**	**[0.137; 0.353]**	**37.1%**
Heterogeneity: I^2 = 85%								
**Risk of Bias: Serious**								
Priebe	+	Other transfer	DIALOG QoL	GBR	High	0.710	[0.265; 1.155]	3.1%
Apouey	.	Lottery win	Subj. LS	GBR	Serious	0.068	[0.027; 0.109]	5.8%
Boyd-Swan	.	Tax/wage policy	Subj. hap	USA	Serious	0.071	[-0.060; 0.201]	5.4%
Gulal	.	Tax/wage policy	Subj. LS	DEU	Serious	0.061	[0.013; 0.108]	5.7%
Lachowska (high)	.	Tax/wage policy	Subj. LS	USA	Serious	0.147	[-0.523; 0.817]	1.9%
Lachowska (low)	.	Tax/wage policy	Subj. LS	USA	Serious	0.667	[-1.152; 2.486]	0.3%
Clark (f)	.	Unknown	Subj. LS	DEU	Serious	0.084	[0.066; 0.103]	3.2%
Clark (m)	.	Unknown	Subj. LS	DEU	Serious	0.067	[0.045; 0.088]	2.6%
Dang (f)	.	Unknown	Subj. LS	RUS	Serious	0.077	[0.060; 0.095]	2.9%
Dang (m)	.	Unknown	Subj. LS	RUS	Serious	0.059	[0.042; 0.077]	2.9%
Frijters (2011)	.	Unknown	Subj. LS	AUS	Serious	0.173	[0.110; 0.236]	5.7%
Gros	.	Welf. policy (£)	Subj. hap	BGD	Serious	0.475	[0.238; 0.712]	4.7%
Galama	.	Welf. policy (£)	Subj. LS	COL	Serious	1.497	[1.116; 1.879]	3.6%
Maeder	.	Welf. policy (£)	Subj. LS	DEU	Serious	0.545	[-0.039; 1.129]	2.3%
Handa (f)	.	Welf. policy (£+)	Subj. LS	GHA	Serious	0.525	[-0.003; 1.053]	1.3%
Handa (m)	.	Welf. policy (£+)	Subj. LS	GHA	Serious	0.093	[-0.252; 0.439]	3.0%
**Random effects model**						**0.315**	**[0.089; 0.540]**	**54.3%**
Heterogeneity: I^2 = 84%								
**Risk of Bias: Critical**								
Rosenheck	.	Benefits advice	Subj. QoL	USA	Critical	0.226	[-0.104; 0.555]	3.9%
Evans (2005)	.	Unknown	Subj. QoL	GBR	Critical	0.233	[0.000; 0.466]	4.7%
**Random effects model**						**0.230**	**[0.040; 0.420]**	**8.6%**
Heterogeneity: I^2 = 0%								
**Random effects model**						**0.266**	**[0.150; 0.381]**	**100.0%**
Heterogeneity: I^2 = 86%								
Test for overall effect: $p < 0.001$								
Test for SG diffs: $\chi^2_2 = 0.36$, df = 2 ($p = 0.83$)								

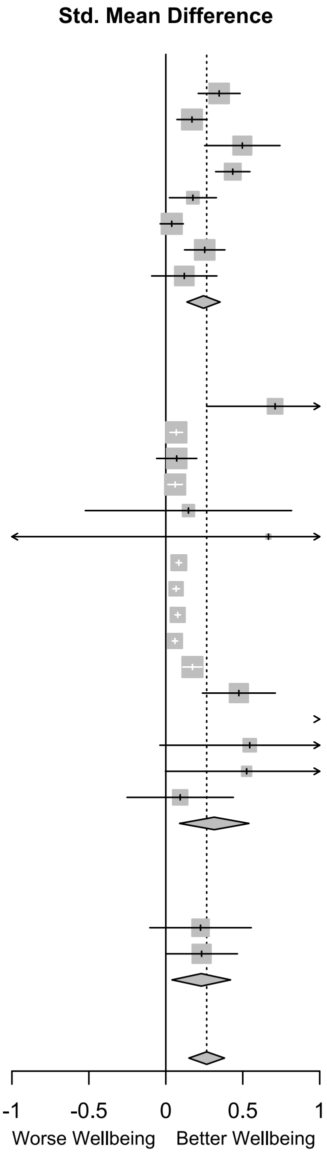

Number of people = 165,217; number of observations = 888,304. Studies sorted by RCT/NRS status → income source → outcome → RoB. + indicates study was a randomised controlled trial. (£) indicates a welfare policy which only affected income; (£+) indicates a welfare policy which also influenced other factors. (f) and (m) indicate studies which stratified results by sex. RoB = Risk of Bias score; SG = subgroup. AIR = American Institutes for Research. DIALOG QoL = DIALOG quality of life scale. Subj. hap = measure of subjective/self-assessed happiness. Subj. LS = measure of subjective/self-assessed life satisfaction. Subj. QoL = measure of subjective/self-assessed quality of life. SWLS = Diener's Satisfaction with Life Scale.

Figure G19: Forest plot for meta-analysis of studies reporting the effect of a binary income increase on a wellbeing outcome, stratified by whether increase likely to move individual out of poverty

Source	RCT	Inc. source	Outcome	Country	RoB	SMD	95% CI	Weight
**Poverty transition: No**								
Priebe	+	Other transfer	DIALOG QoL	GBR	High	0.710	[0.265; 1.155]	3.6%
Kilburn (2018)	+	Welf. policy (£)	Subj. QoL	MWI	Some conc.	0.497	[0.252; 0.742]	5.1%
Dorsett	+	Welf. policy (£+)	Subj. LS	GBR	Some conc.	0.038	[-0.037; 0.114]	6.1%
Apouey	.	Lottery win	Subj. LS	GBR	Serious	0.068	[0.027; 0.109]	6.2%
Boyd-Swan	.	Tax/wage policy	Subj. hap	USA	Serious	0.071	[-0.060; 0.201]	5.9%
Gulal	.	Tax/wage policy	Subj. LS	DEU	Serious	0.061	[0.013; 0.108]	6.2%
Lachowska (high)	.	Tax/wage policy	Subj. LS	USA	Serious	0.147	[-0.523; 0.817]	2.2%
Lachowska (low)	.	Tax/wage policy	Subj. LS	USA	Serious	0.667	[-1.152; 2.486]	0.3%
Frijters (2011)	.	Unknown	Subj. LS	AUS	Serious	0.173	[0.110; 0.236]	6.1%
Powell-Jackson	.	Welf. policy (£)	Subj. hap	IND	Moderate	0.120	[-0.092; 0.332]	5.3%
Maeder	.	Welf. policy (£)	Subj. LS	DEU	Serious	0.545	[-0.039; 1.129]	2.8%
Handa (f)	.	Welf. policy (£+)	Subj. LS	GHA	Serious	0.525	[-0.003; 1.053]	1.4%
Handa (m)	.	Welf. policy (£+)	Subj. LS	GHA	Serious	0.093	[-0.252; 0.439]	3.3%
**Random effects model**						**0.160**	**[0.069; 0.252]**	**54.6%**
Heterogeneity: $I^2 = 65\%$								
**Poverty transition: Yes**								
Natali	+	Welf. policy (£)	Subj. hap	ZMB	Some conc.	0.347	[0.210; 0.483]	5.8%
Haushofer	+	Welf. policy (£)	Subj. LS	KEN	Some conc.	0.170	[0.072; 0.268]	6.0%
Bedoya (f)	+	Welf. policy (£+)	Subj. LS	AFG	Some conc.	0.435	[0.323; 0.547]	3.9%
Bedoya (m)	+	Welf. policy (£+)	Subj. LS	AFG	Some conc.	0.176	[0.023; 0.329]	2.1%
Clark (f)	.	Unknown	Subj. LS	DEU	Serious	0.084	[0.066; 0.103]	3.4%
Clark (m)	.	Unknown	Subj. LS	DEU	Serious	0.067	[0.045; 0.088]	2.8%
Dang (f)	.	Unknown	Subj. LS	RUS	Serious	0.077	[0.060; 0.095]	3.1%
Dang (m)	.	Unknown	Subj. LS	RUS	Serious	0.059	[0.042; 0.077]	3.1%
AIR	.	Welf. policy (£)	SWLS	ZWE	Low	0.253	[0.121; 0.384]	5.9%
Gros	.	Welf. policy (£)	Subj. hap	BGD	Serious	0.475	[0.238; 0.712]	5.2%
Galama	.	Welf. policy (£)	Subj. LS	COL	Serious	> 1.497	[1.116; 1.879]	4.1%
**Random effects model**						**0.377**	**[0.093; 0.661]**	**45.4%**
Heterogeneity: $I^2 = 93\%$								
**Random effects model**						**0.274**	**[0.143; 0.405]**	**100.0%**

Heterogeneity: $I^2 = 87\%$
Test for overall effect: $p < 0.001$
Test for SG diffs: $\chi^2_1 = 2.02$, df = 1 ($p = 0.16$)

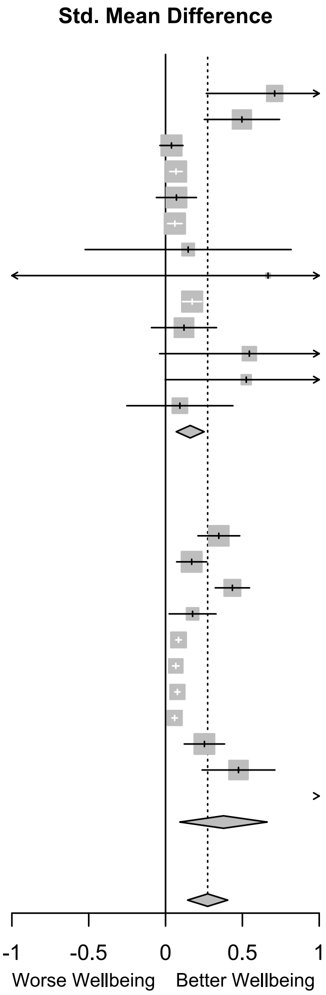

Number of people = 163,969; number of observations = 885,981. Studies sorted by RCT/NRS status → income source → outcome → RoB. + indicates study was a randomised controlled trial. (£) indicates a welfare policy which only affected income; (£+) indicates a welfare policy which also influenced other factors. (f) and (m) indicate studies which stratified results by sex. RoB = Risk of Bias score; SG = subgroup. AIR = American Institutes for Research. DIALOG QoL = DIALOG quality of life scale. Subj. hap = measure of subjective/self-assessed happiness. Subj. LS = measure of subjective/self-assessed life satisfaction. Subj. QoL = measure of subjective/self-assessed quality of life. SWLS = Diener's Satisfaction with Life Scale.

Figure G20: Forest plot for meta-analysis of studies reporting the effect of a binary income increase on a wellbeing outcome, stratified by socioeconomic position

Source	RCT	Inc. source	Outcome	Country	RoB	SMD	95% CI	Weight
**SEP: Mixed**								
Apouey	.	Lottery win	Subj. LS	GBR	Serious	0.068	[0.027; 0.109]	6.2%
Clark (f)	.	Unknown	Subj. LS	DEU	Serious	0.084	[0.066; 0.103]	3.4%
Clark (m)	.	Unknown	Subj. LS	DEU	Serious	0.067	[0.045; 0.088]	2.8%
Dang (f)	.	Unknown	Subj. LS	RUS	Serious	0.077	[0.060; 0.095]	3.1%
Dang (m)	.	Unknown	Subj. LS	RUS	Serious	0.059	[0.042; 0.077]	3.1%
Frijters (2011)	.	Unknown	Subj. LS	AUS	Serious	0.173	[0.110; 0.236]	6.1%
Powell-Jackson	.	Welf. policy (£)	Subj. hap	IND	Moderate	0.120	[-0.092; 0.332]	5.3%
**Random effects model**						**0.088**	**[0.053; 0.123]**	**30.1%**
Heterogeneity: I^2 = 58%								
**SEP: Low**								
Priebe	+	Other transfer	DIALOG QoL	GBR	High	0.710	[0.265; 1.155]	3.6%
Natali	+	Welf. policy (£)	Subj. hap	ZMB	Some conc.	0.347	[0.210; 0.483]	5.8%
Haushofer	+	Welf. policy (£)	Subj. LS	KEN	Some conc.	0.170	[0.072; 0.268]	6.0%
Kilburn (2018)	+	Welf. policy (£)	Subj. QoL	MWI	Some conc.	0.497	[0.252; 0.742]	5.1%
Bedoya (f)	+	Welf. policy (£+)	Subj. LS	AFG	Some conc.	0.435	[0.323; 0.547]	3.9%
Bedoya (m)	+	Welf. policy (£+)	Subj. LS	AFG	Some conc.	0.176	[0.023; 0.329]	2.1%
Dorsett	+	Welf. policy (£+)	Subj. LS	GBR	Some conc.	0.038	[-0.037; 0.114]	6.1%
Boyd-Swan	.	Tax/wage policy	Subj. hap	USA	Serious	0.071	[-0.060; 0.201]	5.9%
Gulal	.	Tax/wage policy	Subj. LS	DEU	Serious	0.061	[0.013; 0.108]	6.2%
Lachowska (low)	.	Tax/wage policy	Subj. LS	USA	Serious	0.667	[-1.152; 2.486]	0.3%
AIR	.	Welf. policy (£)	SWLS	ZWE	Low	0.253	[0.121; 0.384]	5.9%
Gros	.	Welf. policy (£)	Subj. hap	BGD	Serious	0.475	[0.238; 0.712]	5.2%
Galama	.	Welf. policy (£)	Subj. LS	COL	Serious	1.497	[1.116; 1.879]	4.1%
Handa (f)	.	Welf. policy (£+)	Subj. LS	GHA	Serious	0.525	[-0.003; 1.053]	1.4%
Handa (m)	.	Welf. policy (£+)	Subj. LS	GHA	Serious	0.093	[-0.252; 0.439]	3.3%
**Random effects model**						**0.353**	**[0.175; 0.530]**	**64.9%**
Heterogeneity: I^2 = 89%								
**SEP: High**								
Lachowska (high)	.	Tax/wage policy	Subj. LS	USA	Serious	0.147	[-0.523; 0.817]	2.2%
Maeder	.	Welf. policy (£)	Subj. LS	DEU	Serious	0.545	[-0.039; 1.129]	2.8%
**Random effects model**						**0.373**	**[-0.067; 0.814]**	**5.0%**
Heterogeneity: I^2 = 0%								
**Random effects model**						**0.274**	**[0.143; 0.405]**	**100.0%**

Heterogeneity: I^2 = 87%
Test for overall effect: p < 0.001
Test for SG diffs: χ^2_2 = 9.72, df = 2 (p < 0.01)

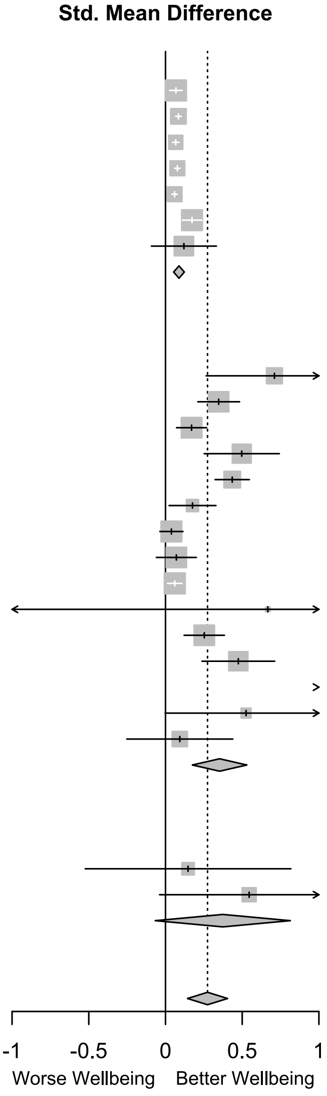

Number of people = 163,969; number of observations = 885,981. Studies sorted by RCT/NRS status → income source → outcome → RoB. + indicates study was a randomised controlled trial. (£) indicates a welfare policy which only affected income; (£+) indicates a welfare policy which also influenced other factors. (f) and (m) indicate studies which stratified results by sex. RoB = Risk of Bias score; SG = subgroup. AIR = American Institutes for Research. DIALOG QoL = DIALOG quality of life scale. Subj. hap = measure of subjective/self-assessed happiness. Subj. LS = measure of subjective/self-assessed life satisfaction. Subj. QoL = measure of subjective/self-assessed quality of life. SWLS = Diener's Satisfaction with Life Scale.

Figure G21: Forest plot for meta-analysis of studies reporting the effect of a binary income increase on a wellbeing outcome, stratified by setting

Source	RCT	Inc. source	Outcome	Country	RoB	SMD	95% CI	Weight
**Setting: High income**								
Priebe	+	Other transfer	DIALOG QoL	GBR	High	0.710	[0.265; 1.155]	3.6%
Dorsett	+	Welf. policy (£+)	Subj. LS	GBR	Some conc.	0.038	[-0.037; 0.114]	6.1%
Apouey	.	Lottery win	Subj. LS	GBR	Serious	0.068	[0.027; 0.109]	6.2%
Boyd-Swan	.	Tax/wage policy	Subj. hap	USA	Serious	0.071	[-0.060; 0.201]	5.9%
Gulal	.	Tax/wage policy	Subj. LS	DEU	Serious	0.061	[0.013; 0.108]	6.2%
Lachowska (high)	.	Tax/wage policy	Subj. LS	USA	Serious	0.147	[-0.523; 0.817]	2.2%
Lachowska (low)	.	Tax/wage policy	Subj. LS	USA	Serious	0.667	[-1.152; 2.486]	0.3%
Clark (f)	.	Unknown	Subj. LS	DEU	Serious	0.084	[0.066; 0.103]	3.4%
Clark (m)	.	Unknown	Subj. LS	DEU	Serious	0.067	[0.045; 0.088]	2.8%
Frijters (2011)	.	Unknown	Subj. LS	AUS	Serious	0.173	[0.110; 0.236]	6.1%
Maeder	.	Welf. policy (£)	Subj. LS	DEU	Serious	0.545	[-0.039; 1.129]	2.8%
**Random effects model**						**0.087**	**[0.052; 0.122]**	**45.6%**
Heterogeneity: $I^2 = 56\%$								
**Setting: Low/middle income**								
Natali	+	Welf. policy (£)	Subj. hap	ZMB	Some conc.	0.347	[0.210; 0.483]	5.8%
Haushofer	+	Welf. policy (£)	Subj. LS	KEN	Some conc.	0.170	[0.072; 0.268]	6.0%
Kilburn (2018)	+	Welf. policy (£)	Subj. QoL	MWI	Some conc.	0.497	[0.252; 0.742]	5.1%
Bedoya (f)	+	Welf. policy (£+)	Subj. LS	AFG	Some conc.	0.435	[0.323; 0.547]	3.9%
Bedoya (m)	+	Welf. policy (£+)	Subj. LS	AFG	Some conc.	0.176	[0.023; 0.329]	2.1%
Dang (f)	.	Unknown	Subj. LS	RUS	Serious	0.077	[0.060; 0.095]	3.1%
Dang (m)	.	Unknown	Subj. LS	RUS	Serious	0.059	[0.042; 0.077]	3.1%
AIR	.	Welf. policy (£)	SWLS	ZWE	Low	0.253	[0.121; 0.384]	5.9%
Powell-Jackson	.	Welf. policy (£)	Subj. hap	IND	Moderate	0.120	[-0.092; 0.332]	5.3%
Gros	.	Welf. policy (£)	Subj. hap	BGD	Serious	0.475	[0.238; 0.712]	5.2%
Galama	.	Welf. policy (£)	Subj. LS	COL	Serious	1.497	[1.116; 1.879]	4.1%
Handa (f)	.	Welf. policy (£+)	Subj. LS	GHA	Serious	0.525	[-0.003; 1.053]	1.4%
Handa (m)	.	Welf. policy (£+)	Subj. LS	GHA	Serious	0.093	[-0.252; 0.439]	3.3%
**Random effects model**						**0.371**	**[0.153; 0.589]**	**54.4%**
Heterogeneity: $I^2 = 92\%$								
**Random effects model**						**0.274**	**[0.143; 0.405]**	**100.0%**

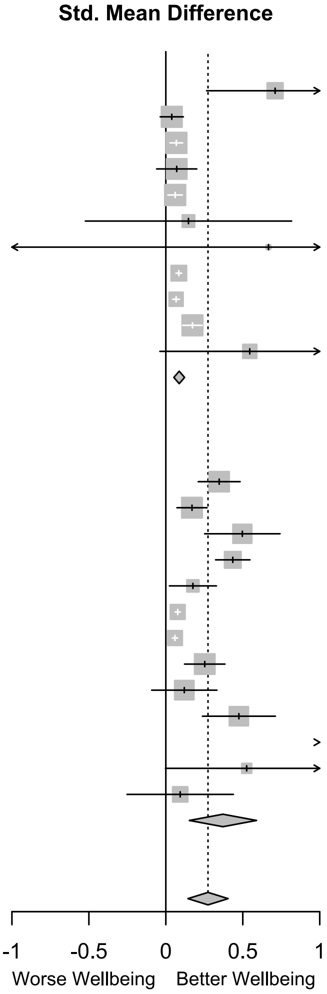

Heterogeneity: $I^2 = 87\%$
Test for overall effect: $p < 0.001$
Test for SG diffs: $\chi^2_1 = 6.34$, df = 1 ($p = 0.01$)

Number of people = 163,969; number of observations = 885,981. Studies sorted by RCT/NRS status → income source → outcome → RoB. + indicates study was a randomised controlled trial. (£) indicates a welfare policy which only affected income; (£+) indicates a welfare policy which also influenced other factors. (f) and (m) indicate studies which stratified results by sex. RoB = Risk of Bias score; SG = subgroup. AIR = American Institutes for Research. DIALOG QoL = DIALOG quality of life scale. Subj. hap = measure of subjective/self-assessed happiness. Subj. LS = measure of subjective/self-assessed life satisfaction. Subj. QoL = measure of subjective/self-assessed quality of life. SWLS = Diener's Satisfaction with Life Scale.

Figure G22: Forest plot for meta-analysis of studies reporting the effect of a binary income increase on a wellbeing outcome, stratified by age

Source	RCT	Inc. source	Outcome	Country	RoB	SMD	95% CI	Weight
**Age: Working age only**								
Priebe	+	Other transfer	DIALOG QoL	GBR	High	0.710	[0.265; 1.155]	3.6%
Natali	+	Welf. policy (£)	Subj. hap	ZMB	Some conc.	0.347	[0.210; 0.483]	5.8%
Haushofer	+	Welf. policy (£)	Subj. LS	KEN	Some conc.	0.170	[0.072; 0.268]	6.0%
Bedoya (f)	+	Welf. policy (£+)	Subj. LS	AFG	Some conc.	0.435	[0.323; 0.547]	3.9%
Bedoya (m)	+	Welf. policy (£+)	Subj. LS	AFG	Some conc.	0.176	[0.023; 0.329]	2.1%
Dorsett	+	Welf. policy (£+)	Subj. LS	GBR	Some conc.	0.038	[-0.037; 0.114]	6.1%
Boyd-Swan	.	Tax/wage policy	Subj. hap	USA	Serious	0.071	[-0.060; 0.201]	5.9%
Gulal	.	Tax/wage policy	Subj. LS	DEU	Serious	0.061	[0.013; 0.108]	6.2%
Powell-Jackson	.	Welf. policy (£)	Subj. hap	IND	Moderate	0.120	[-0.092; 0.332]	5.3%
Gros	.	Welf. policy (£)	Subj. hap	BGD	Serious	0.475	[0.238; 0.712]	5.2%
Maeder	.	Welf. policy (£)	Subj. LS	DEU	Serious	0.545	[-0.039; 1.129]	2.8%
**Random effects model**						**0.231**	**[0.121; 0.342]**	**52.9%**
Heterogeneity: I^2 = 86%								
**Age: Includes <16s or >64s**								
Kilburn (2018)	+	Welf. policy (£)	Subj. QoL	MWI	Some conc.	0.497	[0.252; 0.742]	5.1%
Apouey	.	Lottery win	Subj. LS	GBR	Serious	0.068	[0.027; 0.109]	6.2%
Lachowska (high)	.	Tax/wage policy	Subj. LS	USA	Serious	0.147	[-0.523; 0.817]	2.2%
Lachowska (low)	.	Tax/wage policy	Subj. LS	USA	Serious	0.667	[-1.152; 2.486]	0.3%
Clark (f)	.	Unknown	Subj. LS	DEU	Serious	0.084	[0.066; 0.103]	3.4%
Clark (m)	.	Unknown	Subj. LS	DEU	Serious	0.067	[0.045; 0.088]	2.8%
Dang (f)	.	Unknown	Subj. LS	RUS	Serious	0.077	[0.060; 0.095]	3.1%
Dang (m)	.	Unknown	Subj. LS	RUS	Serious	0.059	[0.042; 0.077]	3.1%
Frijters (2011)	.	Unknown	Subj. LS	AUS	Serious	0.173	[0.110; 0.236]	6.1%
AIR	.	Welf. policy (£)	SWLS	ZWE	Low	0.253	[0.121; 0.384]	5.9%
Galama	.	Welf. policy (£)	Subj. LS	COL	Serious	1.497	[1.116; 1.879]	4.1%
Handa (f)	.	Welf. policy (£+)	Subj. LS	GHA	Serious	0.525	[-0.003; 1.053]	1.4%
Handa (m)	.	Welf. policy (£+)	Subj. LS	GHA	Serious	0.093	[-0.252; 0.439]	3.3%
**Random effects model**						**0.320**	**[0.047; 0.594]**	**47.1%**
Heterogeneity: I^2 = 87%								
**Random effects model**						**0.274**	**[0.143; 0.405]**	**100.0%**

Heterogeneity: I^2 = 87%
Test for overall effect: $p < 0.001$
Test for SG diffs: $\chi^2_1 = 0.35$, df = 1 ($p = 0.55$)

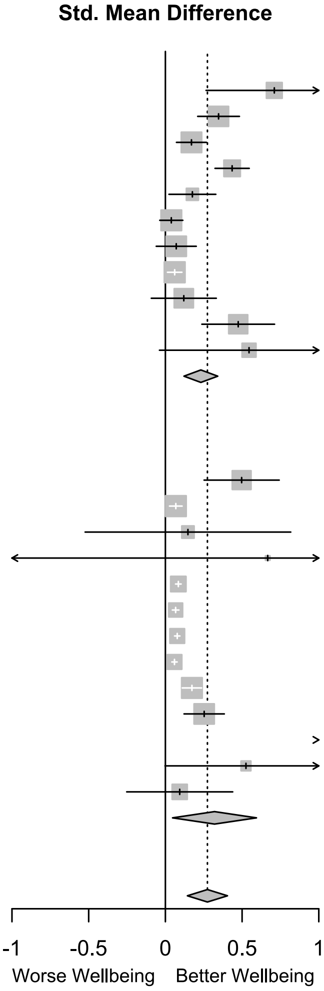

Number of people = 163,969; number of observations = 885,981. Studies sorted by RCT/NRS status → income source → outcome → RoB. + indicates study was a randomised controlled trial. (£) indicates a welfare policy which only affected income; (£+) indicates a welfare policy which also influenced other factors. (f) and (m) indicate studies which stratified results by sex. RoB = Risk of Bias score; SG = subgroup. AIR = American Institutes for Research. DIALOG QoL = DIALOG quality of life scale. Subj. hap = measure of subjective/self-assessed happiness. Subj. LS = measure of subjective/self-assessed life satisfaction. Subj. QoL = measure of subjective/self-assessed quality of life. SWLS = Diener's Satisfaction with Life Scale.

Figure G23: Forest plot for meta-analysis of studies reporting the effect of a binary income decrease on a wellbeing outcome, stratified by risk of bias

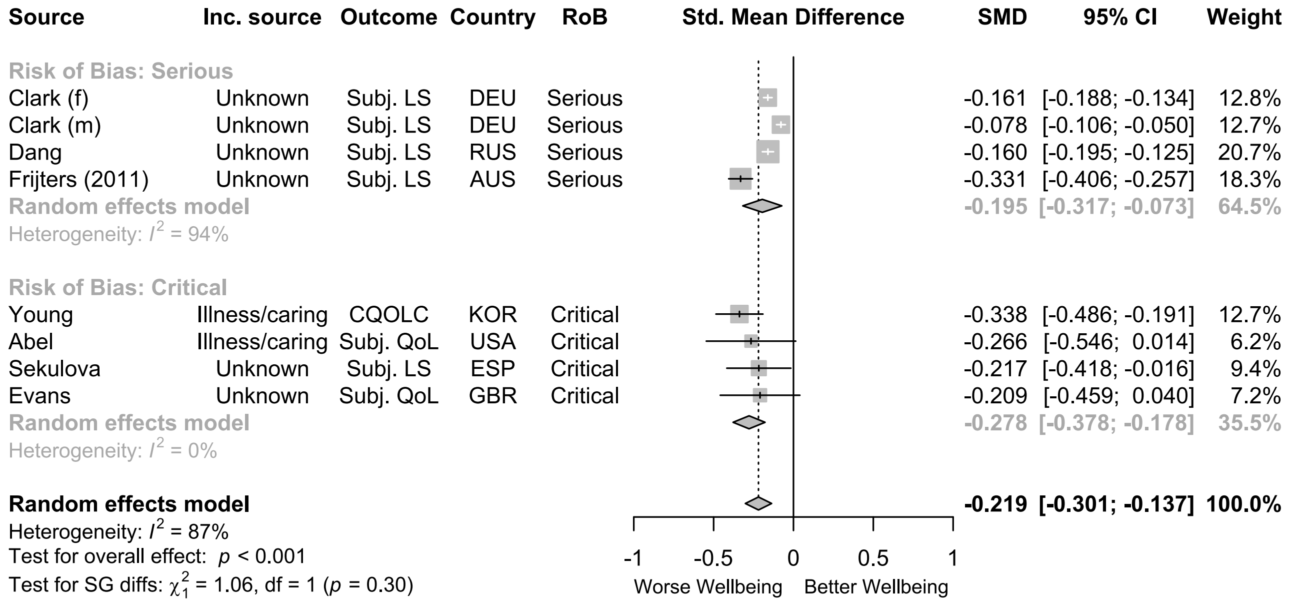

Number of people = 65,456; number of observations = 472,754. Studies sorted by RCT/NRS status → income source → outcome → RoB. (f) and (m) indicate studies which stratified results by sex. RoB = Risk of Bias score; SG = subgroup. CWOLC = Caregiver Quality of Life Index-Cancer. Subj. LS = measure of subjective/self-assessed life satisfaction. Subj. QoL = measure of subjective/self-assessed quality of life.

Figure G24: Forest plot for meta-analysis of studies reporting the effect of a continuous log(income) change on a wellbeing outcome, stratified by sex

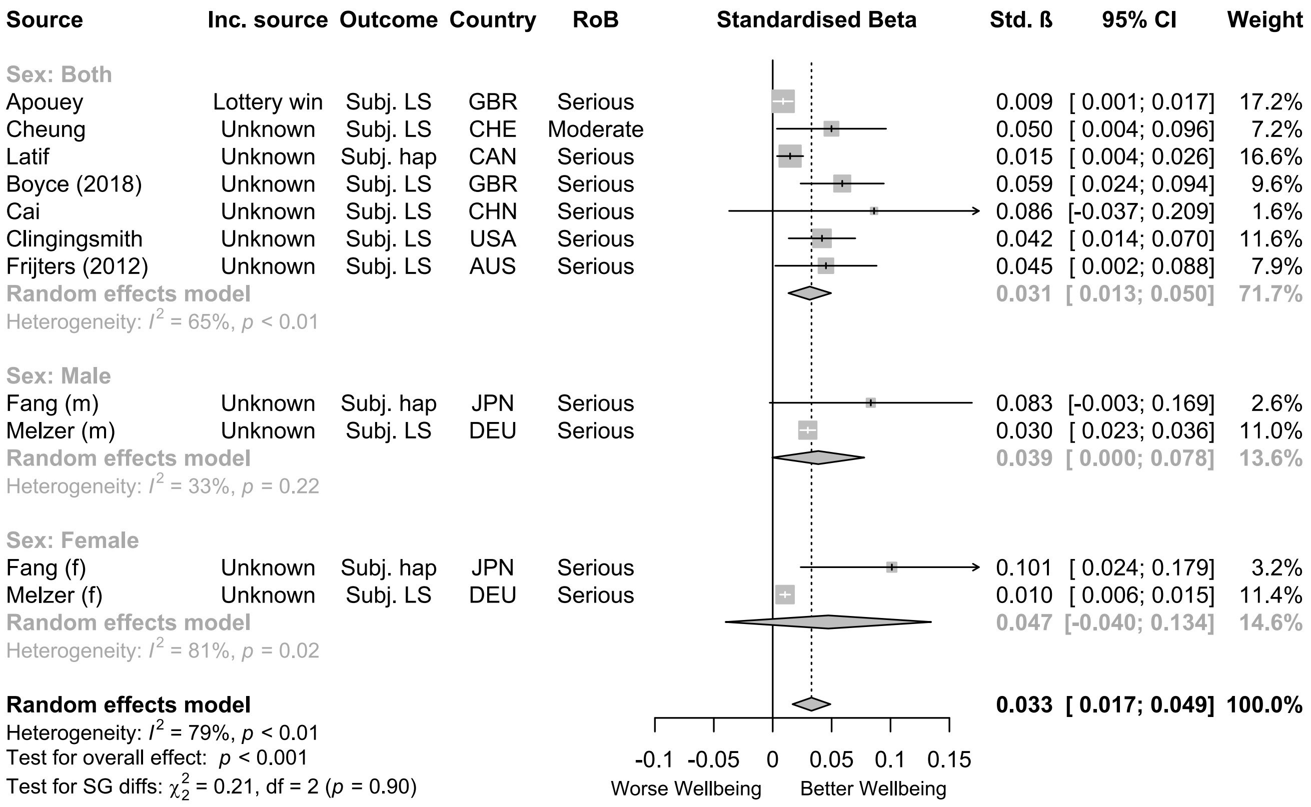

Number of people = 105,326; number of observations = 567,356. Studies sorted by RCT/NRS status → income source → outcome → RoB. (f) and (m) indicate studies stratified results by sex. RoB = Risk of Bias score; SG = subgroup. Subj. hap = measure of subjective/self-assessed happiness. Subj. LS = measure of subjective/self-assessed life satisfaction.

Figure G25: Forest plot for meta-analysis of studies reporting the effect of a continuous log(income) change on a wellbeing outcome, stratified by age

Source	Inc. source	Outcome	Country	RoB	Std. ß	95% CI	Weight
**Age: Working age only**							
Latif	Unknown	Subj. hap	CAN	Serious	0.015	[0.004; 0.026]	16.6%
Cai	Unknown	Subj. LS	CHN	Serious	0.086	[-0.037; 0.209]	1.6%
Clingingsmith	Unknown	Subj. LS	USA	Serious	0.042	[0.014; 0.070]	11.6%
Melzer (f)	Unknown	Subj. LS	DEU	Serious	0.010	[0.006; 0.015]	11.4%
Melzer (m)	Unknown	Subj. LS	DEU	Serious	0.030	[0.023; 0.036]	11.0%
**Random effects model**					**0.022**	**[0.010; 0.034]**	**52.2%**
Heterogeneity: $I^2 = 86\%$, $p < 0.01$							
**Age: Includes <16s or >64s**							
Apouey	Lottery win	Subj. LS	GBR	Serious	0.009	[0.001; 0.017]	17.2%
Cheung	Unknown	Subj. LS	CHE	Moderate	0.050	[0.004; 0.096]	7.2%
Fang (f)	Unknown	Subj. hap	JPN	Serious	0.101	[0.024; 0.179]	3.2%
Fang (m)	Unknown	Subj. hap	JPN	Serious	0.083	[-0.003; 0.169]	2.6%
Boyce (2018)	Unknown	Subj. LS	GBR	Serious	0.059	[0.024; 0.094]	9.6%
Frijters (2012)	Unknown	Subj. LS	AUS	Serious	0.045	[0.002; 0.088]	7.9%
**Random effects model**					**0.045**	**[0.016; 0.073]**	**47.8%**
Heterogeneity: $I^2 = 74\%$, $p < 0.01$							
**Random effects model**					**0.033**	**[0.017; 0.049]**	**100.0%**

Heterogeneity: $I^2 = 79\%$, $p < 0.01$
Test for overall effect: $p < 0.001$
Test for SG diffs: $\chi^2_1 = 2.06$, df = 1 ($p = 0.15$)

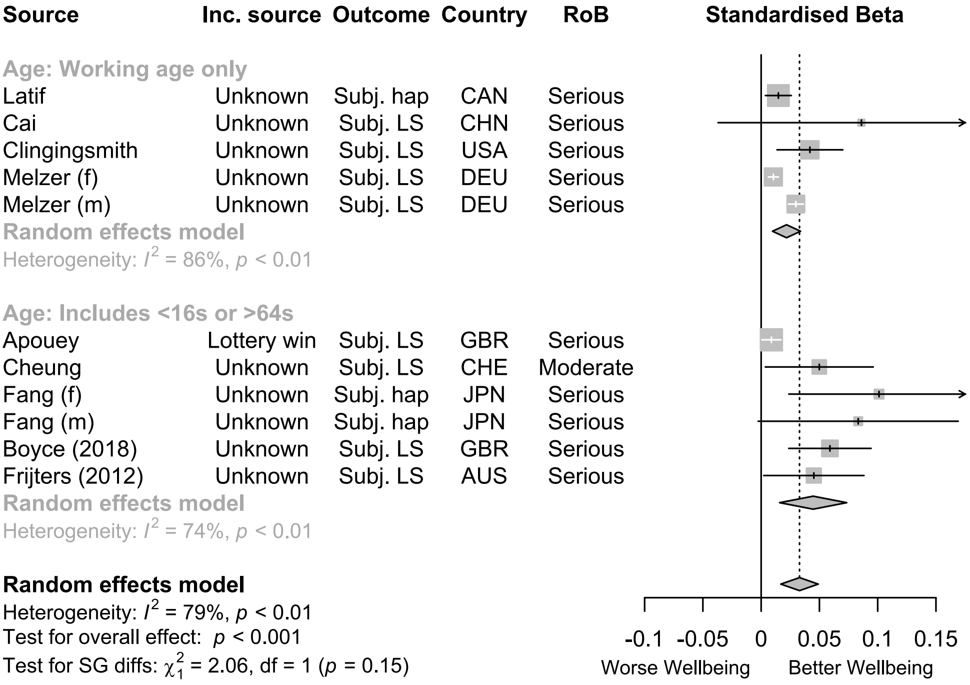

Number of people = 105,326; number of observations = 567,356. Studies sorted by RCT/NRS status → income source → outcome → RoB. (f) and (m) indicate studies stratified results by sex. RoB = Risk of Bias score; SG = subgroup. Subj. hap = measure of subjective/self-assessed happiness. Subj. LS = measure of subjective/self-assessed life satisfaction.

Additional analysis using binary outcomes

Figure G26: Forest plot for meta-analysis of studies reporting the effect of a continuous log(income) change on a binary mental health outcome

Source	Inc. source	Outcome	Country	RoB	OR	95% CI	Weight
Junna	Unknown	MH prescr.	FIN	Moderate	1.000	[0.981; 1.020]	39.3%
Kiernan	Unknown	CES-D	IRL	Serious	0.784	[0.630; 0.977]	4.6%
Zimmerman (f, high)	Unknown	CES-D	USA	Serious	1.099	[0.867; 1.392]	2.9%
Zimmerman (f, low)	Unknown	CES-D	USA	Serious	0.982	[0.816; 1.183]	4.7%
Zimmerman (m, high)	Unknown	CES-D	USA	Serious	0.915	[0.731; 1.145]	3.2%
Zimmerman (m, low)	Unknown	CES-D	USA	Serious	1.129	[0.922; 1.381]	4.0%
Lorant	Unknown	HDL	BEL	Serious	0.950	[0.829; 1.089]	10.2%
Clingingsmith	Unknown	Kessler	USA	Serious	0.934	[0.892; 0.978]	31.0%
**Random effects model**					**0.967**	**[0.919; 1.016]**	**100.0%**

Heterogeneity: $I^2 = 53\%$
Test for overall effect: $p = 0.186$

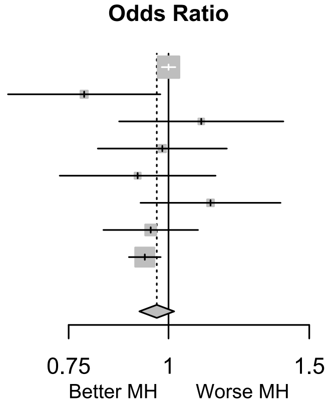

Number of people = 368,714; number of observations = 2,943,756. Studies sorted by income source → outcome → RoB. (f) and (m) indicate studies stratified results by sex. (low) and (high) indicate studies stratified results by socioeconomic position (for Zimmerman, measured by whether household income was below or above study median). RoB = Risk of Bias score; SG = subgroup. CES-D = Center for Epidemiological Studies-Depression scale. HDL = Health and Daily Living Form. Kessler = Kessler Psychological Distress scale. MH prescr. = measure of mental health prescriptions.

Figure G27: Forest plot for meta-analysis of studies reporting the effect of a continuous log(income) change on a binary mental health outcome, stratified by sex

Source	Inc. source	Outcome	Country	RoB	OR	95% CI	Weight
**Sex: Both**							
Junna	Unknown	MH prescr.	FIN	Moderate	1.000	[0.981; 1.020]	39.3%
Lorant	Unknown	HDL	BEL	Serious	0.950	[0.829; 1.089]	10.2%
Clingingsmith	Unknown	Kessler	USA	Serious	0.934	[0.892; 0.978]	31.0%
**Random effects model**					**0.968**	**[0.917; 1.022]**	**80.5%**
Heterogeneity: $I^2 = 74\%$							
**Sex: Male**							
Zimmerman (m, high)	Unknown	CES-D	USA	Serious	0.915	[0.731; 1.145]	3.2%
Zimmerman (m, low)	Unknown	CES-D	USA	Serious	1.129	[0.922; 1.381]	4.0%
**Random effects model**					**1.022**	**[0.832; 1.255]**	**7.2%**
Heterogeneity: $I^2 = 46\%$							
**Sex: Female**							
Kiernan	Unknown	CES-D	IRL	Serious	0.784	[0.630; 0.977]	4.6%
Zimmerman (f, high)	Unknown	CES-D	USA	Serious	1.099	[0.867; 1.392]	2.9%
Zimmerman (f, low)	Unknown	CES-D	USA	Serious	0.982	[0.816; 1.183]	4.7%
**Random effects model**					**0.908**	**[0.700; 1.180]**	**12.2%**
Heterogeneity: $I^2 = 56\%$							
**Random effects model**					**0.967**	**[0.919; 1.016]**	**100.0%**

Heterogeneity: $I^2 = 53\%$
Test for overall effect: $p = 0.186$
Test for SG diffs: $\chi^2_2 = 0.50$, df = 2 ($p = 0.78$)

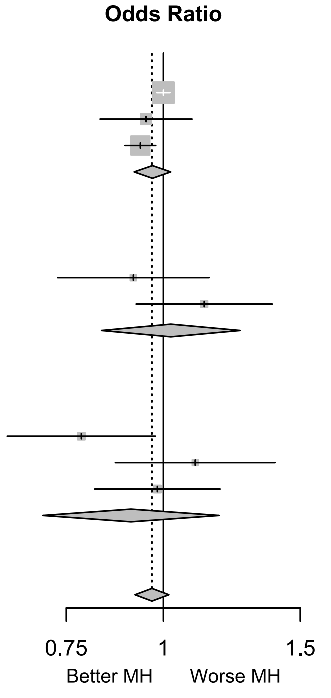

Number of people = 368,714; number of observations = 2,943,756. Studies sorted by income source → outcome → RoB. (f) and (m) indicate studies stratified results by sex. (low) and (high) indicate studies stratified results by socioeconomic position (for Zimmerman, measured by whether household income was below or above study median). RoB = Risk of Bias score; SG = subgroup. CES-D = Center for Epidemiological Studies-Depression scale. HDL = Health and Daily Living Form. Kessler = Kessler Psychological Distress scale. MH prescr. = measure of mental health prescriptions.

Sensitivity analysis excluding calculations which used an external standard deviation

Figure G28: Forest plot for meta-analysis of studies reporting the effect of a binary income increase on a mental health outcome, excluding calculations using an external standard deviation

Source	RCT	Inc. source	Outcome	Country	RoB	SMD	95% CI	Weight
Priebe	+	Other transfer	CGI scale	GBR	Some conc.	0.554	[-0.245; 1.352]	0.4%
Kilburn (2016, f)	+	Welf. policy (£)	CES-D	KEN	Low	-0.037	[-0.236; 0.161]	2.3%
Kilburn (2016, m)	+	Welf. policy (£)	CES-D	KEN	Low	0.282	[0.120; 0.444]	2.7%
Fernald	+	Welf. policy (£)	CES-D	ECU	Some conc.	-0.065	[-0.207; 0.077]	3.5%
Haushofer	+	Welf. policy (£)	CES-D	KEN	Some conc.	0.125	[0.032; 0.217]	4.1%
Baird (a)	+	Welf. policy (£)	GHQ	MWI	Some conc.	-0.009	[-0.168; 0.150]	2.5%
Baird (b)	+	Welf. policy (£)	GHQ	MWI	Some conc.	0.154	[0.049; 0.260]	3.2%
Hjelm (CGP)	+	Welf. policy (£)	PSS	ZMB	Some conc.	-0.017	[-0.172; 0.139]	2.7%
Hjelm (MCP)	+	Welf. policy (£)	PSS	ZMB	Some conc.	0.089	[-0.060; 0.238]	2.7%
Green	+	Welf. policy (£+)	APAI	UGA	Low	0.057	[-0.054; 0.168]	3.9%
Bedoya (f)	+	Welf. policy (£+)	CES-D	AFG	Some conc.	0.504	[0.388; 0.620]	3.1%
Bedoya (m)	+	Welf. policy (£+)	CES-D	AFG	Some conc.	0.198	[0.045; 0.351]	2.6%
Gennetian	+	Welf. policy (£+)	CES-D	USA	Some conc.	0.129	[-0.033; 0.290]	3.3%
Macours	+	Welf. policy (£+)	CES-D	NIC	Some conc.	0.605	[-0.863; 2.073]	0.1%
Raschke (high)		Lottery win	SF-MCS	DEU	Serious	-0.012	[-0.326; 0.303]	1.4%
Raschke (low)		Lottery win	SF-MCS	DEU	Serious	-0.264	[-0.467; -0.062]	2.5%
Erixson		Tax/wage policy	MH hosp.	SWE	Low	0.011	[-0.006; 0.028]	4.6%
Boyd-Swan		Tax/wage policy	CES-D	USA	Serious	0.077	[-0.035; 0.189]	3.8%
Burmaster		Tax/wage policy	CES-D	DOM	Serious	0.466	[0.198; 0.733]	2.1%
Reeves (2017)		Tax/wage policy	GHQ	GBR	Serious	0.534	[0.178; 0.889]	1.5%
Koltai		Unknown	Kessler	CAN	Moderate	0.017	[-0.049; 0.083]	4.3%
McCarthy		Unknown	Beck	CAN/USA	Serious	0.018	[-0.098; 0.135]	3.8%
Dearing		Unknown	CES-D	USA	Serious	0.216	[0.008; 0.424]	2.7%
Lorant		Unknown	HDL	BEL	Serious	0.055	[0.020; 0.090]	4.6%
Sareen		Unknown	Inc. mood dis.	USA	Serious	-0.042	[-0.183; 0.098]	3.5%
Costello		Other transfer	CAPA	USA	Moderate	0.361	[-0.059; 0.780]	1.2%
Ong (f)		Other transfer	SF-MCS	AUS	Moderate	-0.011	[-0.031; 0.009]	3.4%
Ong (m)		Other transfer	SF-MCS	AUS	Moderate	-0.003	[-0.023; 0.017]	3.4%
Tachibana		Other transfer	CES-D	NPL	Serious	0.018	[0.003; 0.033]	4.6%
Ozer		Welf. policy (£)	CES-D	MEX	Moderate	0.168	[0.094; 0.241]	4.3%
Powell-Jackson		Welf. policy (£)	Kessler	IND	Moderate	0.015	[0.003; 0.026]	4.6%
Lebihan		Welf. policy (£)	Mat. dep	CAN	Moderate	0.025	[-0.057; 0.107]	4.2%
Gros		Welf. policy (£)	Subj. MH	BGD	Serious	0.332	[0.088; 0.577]	2.3%
**Random effects model**						**0.091**	**[0.038; 0.144]**	**100.0%**

Heterogeneity: I^2 = 82%
Test for overall effect: $p < 0.001$

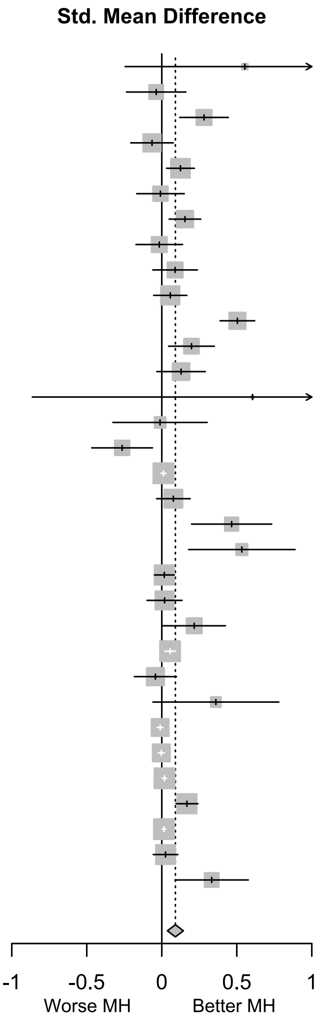

Number of people = 155,940; number of observations = 1,581,150. Studies sorted by RCT/NRS status → income source → outcome → RoB. + indicates study was a randomised controlled trial. (£) indicates a welfare policy which only affected income; (£+) indicates a welfare policy which also influenced other factors. (f) and (m) indicate studies which stratified results by sex; (high) and (low) indicate Raschke et al. stratified results by educational attainment. Baird (a) reported on a cash transfer to school dropouts versus Baird (b) which reported on a cash transfer to girls still enrolled in school; Hjelm (CGP) reported on the Zambia Child Grant Program versus Hjelm (MCP) which reported on the Zambia Multiple Category Cash Transfer Program. RoB = Risk of Bias score; SG = subgroup. APAI = Acholi Psychosocial Assessment Instrument. Beck = Beck depression inventory. CAPA = Child and Adolescent Psychiatric Assessment. CES-D = Center for Epidemiological Studies-Depression scale. CGI = Clinical Global Impression scale. GHQ = General Health Questionnaire. HDL = Health and Daily Living Form. Inc. mood dis. = Incident mood disorder (assessed at clinical interview). Kessler = Kessler Psychological Distress scale. Mat. dep = measure of maternal depression. MH hosp. = mental health hospitalisations. PSS = Perceived Stress Scale. SF-MCS = Mental Component Summary of Short Form Survey. Subj. MH = measure of subjective/self-assessed mental health.

Figure G29: Forest plot for meta-analysis of studies reporting the effect of a binary income decrease on a mental health outcome, excluding calculations using an external standard deviation

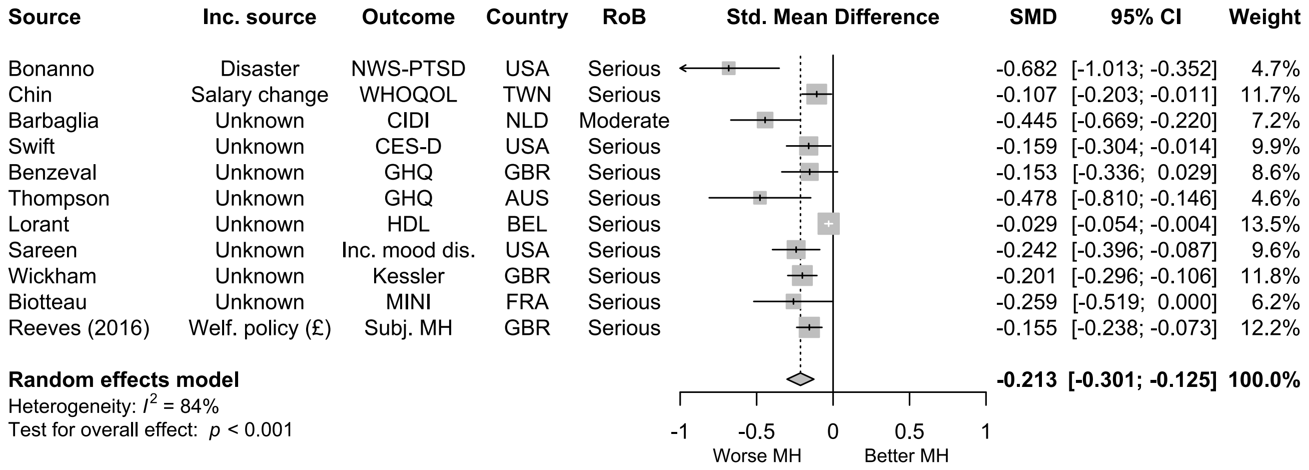

Number of people = 227,804; number of observations = 281,728. Studies sorted by income source → outcome → RoB. (£) indicates a welfare policy which only affected income. RoB = Risk of Bias score; SG = subgroup. CES-D = Center for Epidemiological Studies-Depression scale. CIDI = Composite International Diagnostic Interview. GHQ = General Health Questionnaire. HDL = Health and Daily Living Form. Inc. mood dis. = Incident mood disorder (assessed at clinical interview). Kessler = Kessler Psychological Distress scale. MINI = Mini International Neuropsychiatric Interview. NWS-PTSD = National Women's Study PTSD module. Subj. MH = measure of subjective/self-assessed mental health. WHOQOL = Psychological domain of abbreviated World Health Organisation Quality of Life tool

Figure G30: Forest plot for meta-analysis of studies reporting the effect of a continuous log(income) change on a mental health outcome, excluding calculations using an external

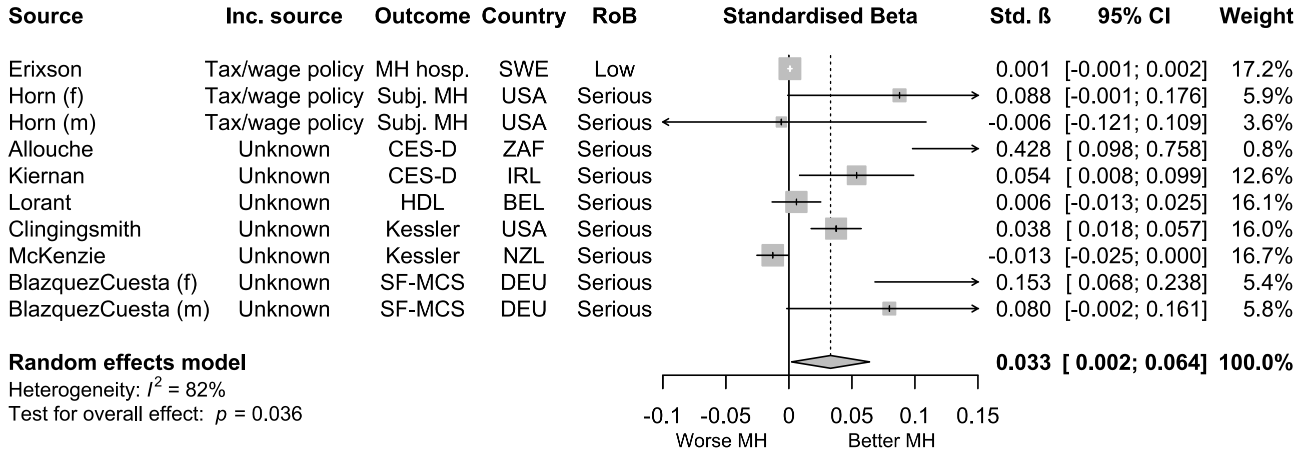

standard deviation

Number of people = 1,494,021; number of observations = 2,948,637. Studies sorted by income source → outcome → RoB. (f) and (m) indicate studies stratified results by sex. RoB = Risk of Bias score; SG = subgroup. CES-D = Center for Epidemiological Studies-Depression scale. GHQ = General Health Questionnaire. HDL = Health and Daily Living Form. Kessler = Kessler Psychological Distress scale. MH hosp. = mental health hospitalisations. Subj. MH = measure of subjective/self-assessed mental health. SF-MCS = Mental Component Summary of Short Form Survey.

Figure G31: Forest plot for meta-analysis of studies reporting the effect of a continuous log(income) change on a binary mental health outcome, excluding calculations using an external standard deviation

Source	Inc. source	Outcome	Country	RoB	OR	95% CI	Weight
Junna	Unknown	MH prescr.	FIN	Moderate	1.000	[0.981; 1.020]	39.3%
Kiernan	Unknown	CES-D	IRL	Serious	0.784	[0.630; 0.977]	4.6%
Zimmerman (f, high)	Unknown	CES-D	USA	Serious	1.099	[0.867; 1.392]	2.9%
Zimmerman (f, low)	Unknown	CES-D	USA	Serious	0.982	[0.816; 1.183]	4.7%
Zimmerman (m, high)	Unknown	CES-D	USA	Serious	0.915	[0.731; 1.145]	3.2%
Zimmerman (m, low)	Unknown	CES-D	USA	Serious	1.129	[0.922; 1.381]	4.0%
Lorant	Unknown	HDL	BEL	Serious	0.950	[0.829; 1.089]	10.2%
Clingingsmith	Unknown	Kessler	USA	Serious	0.934	[0.892; 0.978]	31.0%
**Random effects model**					**0.967**	**[0.919; 1.016]**	**100.0%**

Heterogeneity: $I^2 = 53\%$
Test for overall effect: $p = 0.186$

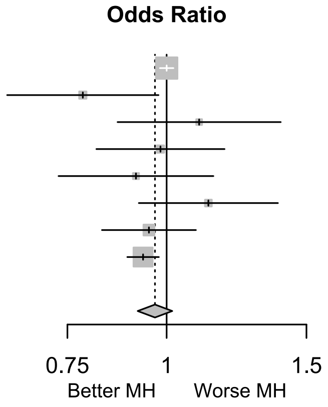

Number of people = 368,714; number of observations = 2,943,756. Studies sorted by income source → outcome → RoB. (f) and (m) indicate studies stratified results by sex. (low) and (high) indicate studies stratified results by socioeconomic position (for Zimmerman, measured by whether household income was below or above study median). RoB = Risk of Bias score; SG = subgroup. CES-D = Center for Epidemiological Studies-Depression scale. HDL = Health and Daily Living Form. Kessler = Kessler Psychological Distress scale. MH prescr. = measure of mental health prescriptions.

Figure G32: Forest plot for meta-analysis of studies reporting the effect of a binary income increase on a wellbeing outcome, excluding calculations using an external standard deviation

Source	RCT	Inc. source	Outcome	Country	RoB	SMD	95% CI	Weight
Priebe	+	Other transfer	DIALOG QoL	GBR	High	0.710	[0.265; 1.155]	4.8%
Natali	+	Welf. policy (£)	Subj. hap	ZMB	Some conc.	0.347	[0.210; 0.483]	7.0%
Haushofer	+	Welf. policy (£)	Subj. LS	KEN	Some conc.	0.170	[0.072; 0.268]	7.2%
Kilburn (2018)	+	Welf. policy (£)	Subj. QoL	MWI	Some conc.	0.497	[0.252; 0.742]	6.3%
Bedoya (f)	+	Welf. policy (£+)	Subj. LS	AFG	Some conc.	0.435	[0.323; 0.547]	4.7%
Bedoya (m)	+	Welf. policy (£+)	Subj. LS	AFG	Some conc.	0.176	[0.023; 0.329]	2.5%
Dorsett	+	Welf. policy (£+)	Subj. LS	GBR	Some conc.	0.038	[-0.037; 0.114]	7.3%
Boyd-Swan		Tax/wage policy	Subj. hap	USA	Serious	0.071	[-0.060; 0.201]	7.0%
Gulal		Tax/wage policy	Subj. LS	DEU	Serious	0.061	[0.013; 0.108]	7.3%
Lachowska (f)		Tax/wage policy	Subj. LS	USA	Serious	0.611	[-0.302; 1.524]	1.9%
Lachowska (m)		Tax/wage policy	Subj. LS	USA	Serious	-0.114	[-1.221; 0.993]	1.3%
Clark (f)		Unknown	Subj. LS	DEU	Serious	0.084	[0.066; 0.103]	4.0%
Clark (m)		Unknown	Subj. LS	DEU	Serious	0.067	[0.045; 0.088]	3.4%
Dang (f)		Unknown	Subj. LS	RUS	Serious	0.077	[0.060; 0.095]	3.7%
Dang (m)		Unknown	Subj. LS	RUS	Serious	0.059	[0.042; 0.077]	3.7%
Powell-Jackson		Welf. policy (£)	Subj. hap	IND	Moderate	0.120	[-0.092; 0.332]	6.6%
Gros		Welf. policy (£)	Subj. hap	BGD	Serious	0.475	[0.238; 0.712]	6.4%
Galama		Welf. policy (£)	Subj. LS	COL	Serious	1.497	[1.116; 1.879]	5.3%
Maeder		Welf. policy (£)	Subj. LS	DEU	Serious	0.545	[-0.039; 1.129]	3.8%
Handa (f)		Welf. policy (£+)	Subj. LS	GHA	Serious	0.525	[-0.003; 1.053]	1.8%
Handa (m)		Welf. policy (£+)	Subj. LS	GHA	Serious	0.093	[-0.252; 0.439]	4.2%
**Random effects model**						**0.311**	**[0.147; 0.474]**	**100.0%**

Heterogeneity: $I^2 = 87\%$
Test for overall effect: $p < 0.001$

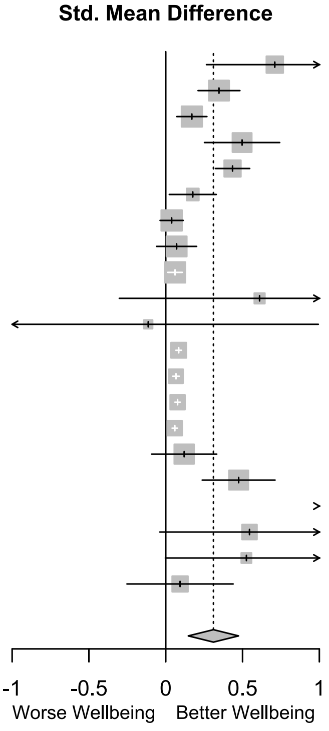

Number of people = 134,232; number of observations = 702,110. Studies sorted by RCT/NRS status → income source → outcome → RoB. + indicates study was a randomised controlled trial. (£) indicates a welfare policy which only affected income; (£+) indicates a welfare policy which also influenced other factors. (f) and (m) indicate studies which stratified results by sex. RoB = Risk of Bias score; SG = subgroup. DIALOG QoL = DIALOG quality of life scale. Subj. hap = measure of subjective/self-assessed happiness. Subj. LS = measure of subjective/self-assessed life satisfaction. Subj. QoL = measure of subjective/self-assessed quality of life.

Figure G33: Forest plot for meta-analysis of studies reporting the effect of a continuous log(income) change on a wellbeing outcome, excluding calculations using an external standard

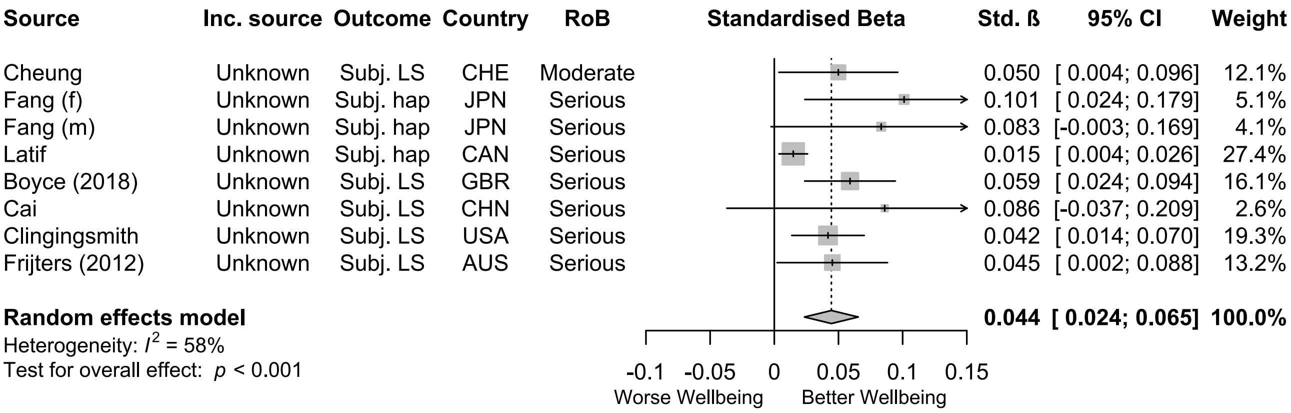

deviation

Number of people = 50,066; number of observations = 223,207. Studies sorted by RCT/NRS status → income source → outcome → RoB. (f) and (m) indicate studies stratified results by sex. RoB = Risk of Bias score; SG = subgroup. Subj. hap = measure of subjective/self-assessed happiness. Subj. LS = measure of subjective/self-assessed life satisfaction.

Funnel plots and Egger's test investigating publication bias/small study effect

Figure G34: Funnel plot for meta-analysis of studies reporting the effect of a binary income increase on a mental health outcome (Fig. 3A)

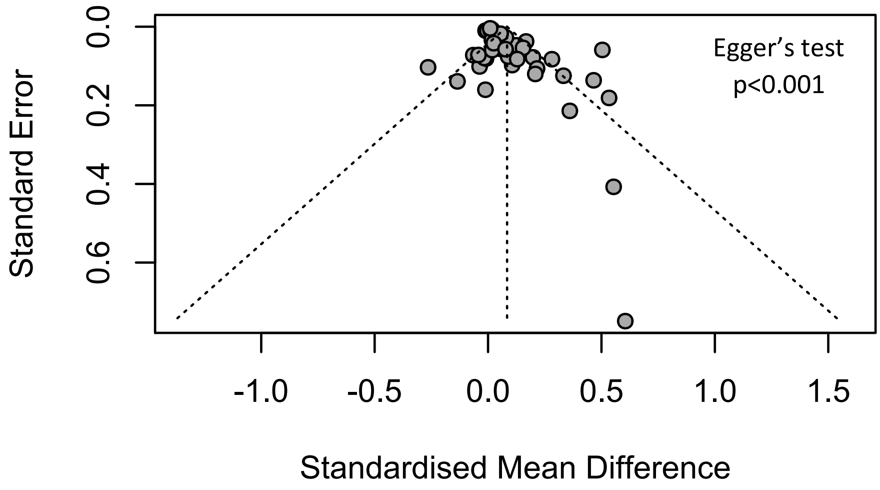

Figure G35: Funnel plot for meta-analysis of studies reporting the effect of a binary income decrease on a mental health outcome (Fig. 3B)

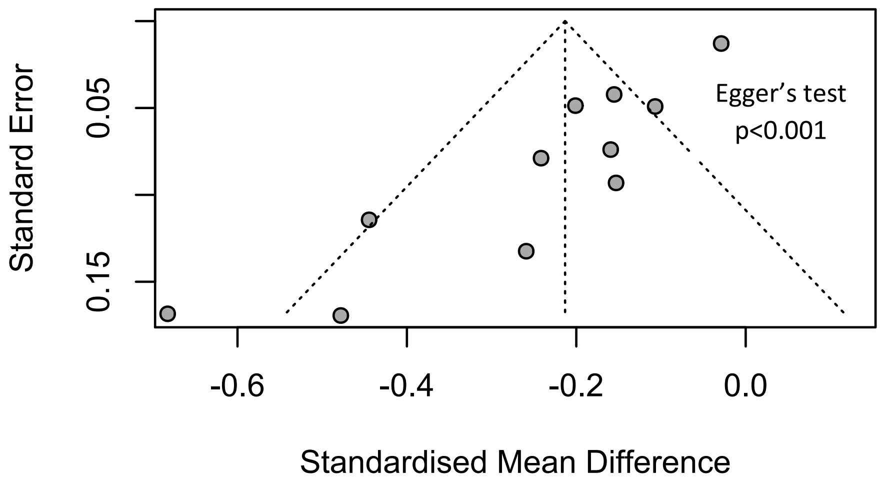

Figure G36: Funnel plot for meta-analysis of studies reporting the effect of a continuous log(income) change on a mental health outcome (Fig. 3C)

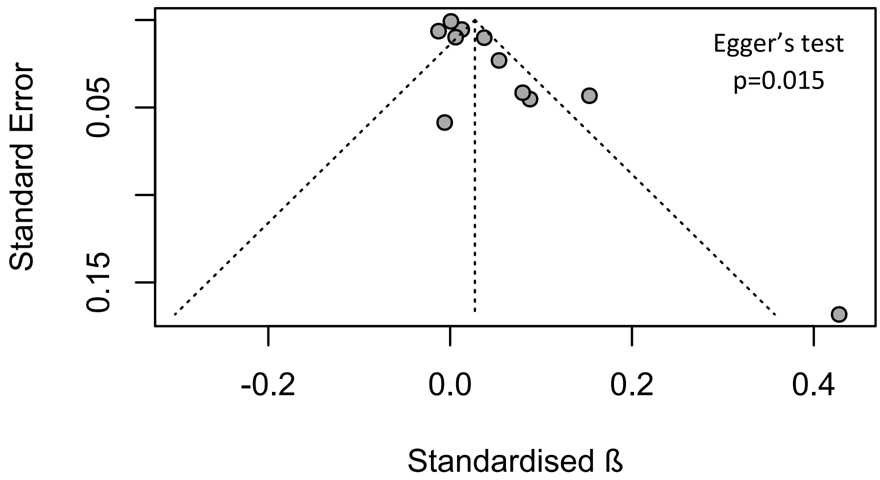

Figure G37: Funnel plot for meta-analysis of studies reporting the effect of a binary income increase on a wellbeing outcome (Fig. 4A)

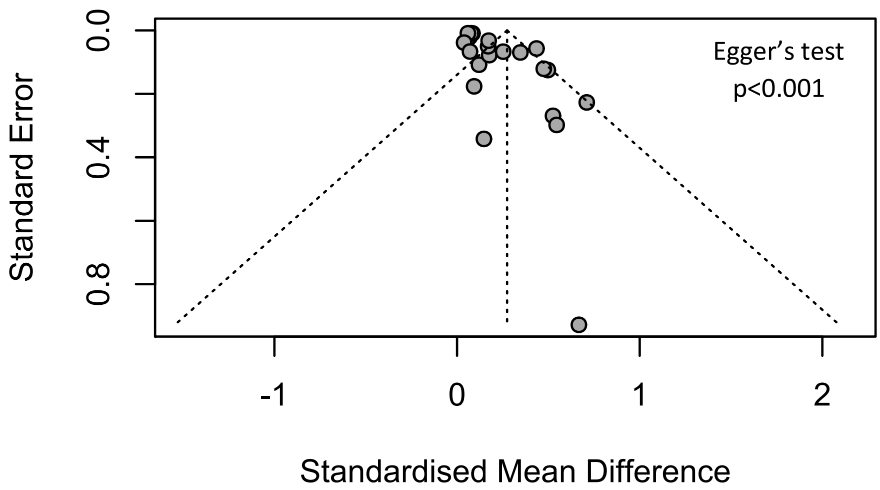

Figure G38: Funnel plot for meta-analysis of studies reporting the effect of a continuous log(income) change on a wellbeing outcome (Fig. 4B)

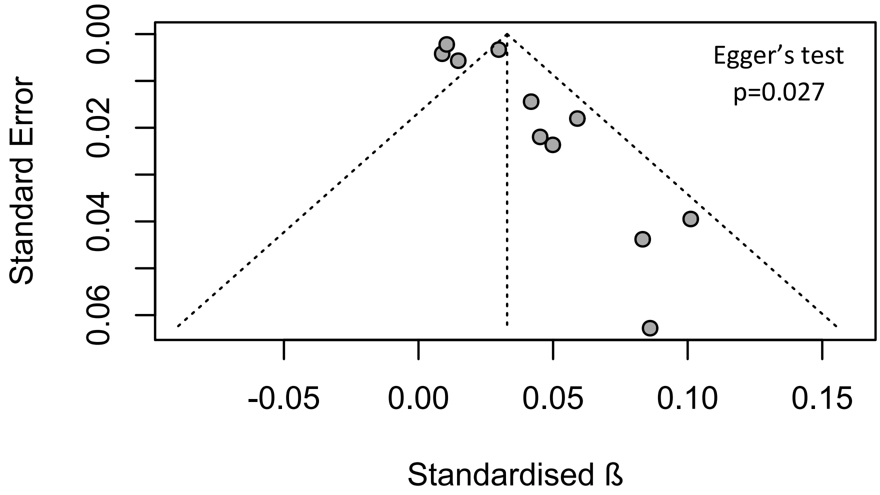

Supplement: Online supplement [file EMS182208-supplement-Online_supplement.pdf]
